# Development of machine learning classifiers to predict compound activity on prostate cancer cell lines

**DOI:** 10.1186/s13321-022-00647-y

**Published:** 2022-11-08

**Authors:** Davide Bonanni, Luca Pinzi, Giulio Rastelli

**Affiliations:** Department of Life Sciences, University of Modena and Reggio Emilia, Via Campi 103, 41125 Modena, Italy

**Keywords:** Machine learning, Prostate cancer, PC-3, DU-145, Drug discovery

## Abstract

Prostate cancer is the most common type of cancer in men. The disease presents good survival rates if treated at the early stages. However, the evolution of the disease in its most aggressive variant remains without effective therapeutic answers. Therefore, the identification of novel effective therapeutics is urgently needed. On these premises, we developed a series of machine learning models, based on compounds with reported highly homogeneous cell-based antiproliferative assay data, able to predict the activity of ligands towards the PC-3 and DU-145 prostate cancer cell lines. The data employed in the development of the computational models was finely-tuned according to a series of thresholds for the classification of active/inactive compounds, to the number of features to be implemented, and by using 10 different machine learning algorithms. Models’ evaluation allowed us to identify the best combination of activity thresholds and ML algorithms for the classification of active compounds, achieving prediction performances with MCC values above 0.60 for PC-3 and DU-145 cells. Moreover, in silico models based on the combination of PC-3 and DU-145 data were also developed, demonstrating excellent precision performances. Finally, an analysis of the activity annotations reported for the ligands in the curated datasets were conducted, suggesting associations between cellular activity and biological targets that might be explored in the future for the design of more effective prostate cancer antiproliferative agents.

**Supplementary Information:**

The online version contains supplementary material available at 10.1186/s13321-022-00647-y.

## Introduction

Prostate cancer (PCa) accounts for around the 14.0% of all new cancer cases in men and caused 31,636 deaths in the United States in 2019, according to the last released data provided by the official federal cancer statistics [1]. Although PCa presents a high degree of survival rate at 5-years, especially if detected at the early stages, the disease can easily evolve from localised to locally advanced PCa or distant metastatic and invade extra-prostatic tissues of organs, such as bone, liver and lungs [2–4]. Androgen deprivation therapy (ADT) is the commonly used therapy for the treatment of PCa at all stages, as testosterone is required for the growth of the prostate cancer cells [5, 6]. Moreover, the ADT treatment is often implemented with luteinizing hormone-releasing hormone (LHRH) agonists or antagonists, anti-androgens drugs (e.g*.* Bicalutamide, Flutamide, Nilutamide), or surgically (i.e., orchiectomy) [5, 7]. Eventually, 10–20% of prostate cancer can evolve into castrate-resistant prostate cancer (CRPC) as a consequence of pharmaceutical castration by ADT [8, 9]. CRPC is defined as the progression of the disease despite the depletion of serum testosterone levels [10, 11]. Indeed, several studies have demonstrated that the androgen receptor (AR) remains active in CRPC, playing an important role in tumor function and growth [12, 13]. Thus, frontiers of research are engaged in the development of new therapies for the treatment of PCA. Current approaches for drug development can be mainly divided into target-based and phenotypic-based [14]. Target-based screening requires prior identification and validation of druggable biological targets. In the last decades, a number of studies based on target-based approaches have been performed in search of drug candidates acting against PCa, their identification and development being facilitated also by the availability of innovative computational and big data tools [15–17]. However, limitations in target-based drug discovery (TBDD) have also been reported, one concern being that target activity often does not translate into cellular activity and clinical efficacy [18]. Besides, phenotypic-based approaches allow the identification of drug candidates by directly assaying them on disease models, such as cells, isolated tissues or animal models, and their mechanism of action (MoA) could also be determined at a later stage [19]. Although phenotypic-based screening can help to address the complexity of a disease, its application is often associated with disadvantages, such as high costs, low throughputs, and difficulties in pursuing further optimization of the emerging hit compounds [20].

In the last years, the integration of computational tools with phenotypic-based screening has increased, demonstrating to help boosting discovery processes while reducing costs. For example, several applications of in silico target-fishing methods (e.g*.*, those based on similarity search, reverse docking and pharmacophore screening) have been actively used to investigate the target space of bioactive compounds from phenotypic screening [21]. Nevertheless, the application of predictive computational models at the early stages of the drug discovery process has also expanded, in trend with the increasing amount of phenotypic-based data available [22, 23]. Indeed, machine learning (ML) and deep learning models have been widely applied to exploit phenotypic-based data, often in tandem with molecular structures and/or “MultiOmics” knowledge, to provide an even more accurate characterization of modern translational precision medicine information. For example, Wang et al.[24] combined cancer genomics, compounds’ chemical properties and biological target information into a single model called Predict Drug Responses in Cancer Cells (PDRCC) to discover novel sensitive associations between cancer cells and anticancer drugs. Moreover, He et al.[25] used molecular descriptors, fingerprints, and molecular graphs data to develop a series of predictive models on 13 different breast cancer cell lines.

In the current study, we performed extensive training on data extracted from ChEMBL (https://www.ebi.ac.uk/chembl/) [26, 27], with the aim of obtaining a series of machine learning models able to predict the antiproliferative cellular activity of compounds against the more aggressive PC-3 and DU-145 prostate cancer cell lines. PC-3 cells are considered highly relevant cellular models for the study of advanced prostate cancer, as they lack AR expression and androgen-independent proliferation [28]. Moreover, PC-3 cells present also a high metastatic potential compared to other PCa cell lines, such as DU-145 and LNCaP. Besides, DU-145 is also AR-negative [29], and is often assayed in tandem with PC-3 to better assess the response of prostate cancer to chemotherapeutic agents. In this study, our attention was firstly focused on the generation of highly homogeneous datasets of compounds for the developments of a series of ML models. To this aim, cellular assay data was classified according to the experimental protocol, in order to retrieve antiproliferative-related records with consistent experimental outcomes. Then, nine different activity thresholds discriminating active and inactive compounds were selected and evaluated against 10 machine learning algorithms, each of them being trained using molecular descriptors as input features. The models were evaluated according to multiple metrics, in order to achieve optimal and robust classification performances. Moreover, we also retrieved and discussed the biological targets associated with the antiproliferative effects of the molecules in the curated PC-3 and DU-145 datasets. Finally, PC-3 and DU-145 datasets were combined to build and evaluate a ML classifier for the two cell lines. Overall, the generated models demonstrated to provide robust results, thus representing a valuable tool for fast in silico phenotypic screening against PC-3 and DU-145 prostate cancer cell lines. The datasets and python scripts implemented in this study are publicly available at https://ligadvisor.unimore.it/downloads.

## Result and discussion

### Data collection and preparation

The quality of data reflects drastically on that of the models [30]. Therefore, data preparation represents a pivotal phase. Several studies have highlighted the significance of harmonized assay and experimental protocols to avoid the generation of inconsistent datasets [31, 32]. This aspect becomes even more relevant in cell-based assays, especially considering the high variety of available experimental methodologies to evaluate the antiproliferative activity. The preparation of a homogeneous set of cellular activity data demands a precise characterization of the data origin. Consistently, activity annotations on PCa cells were collected and filtered from the ChEMBL database, as described in the Methods section. In particular, we focused our attention exclusively on antiproliferative activity data provided by MTT and SRB-based colorimetric assays. MTT assay is a popular method for the determination of metabolic activity of living cells [33, 34]; we collected a total of 498 and 214 records from MTT assays for PC-3 and DU-145 in ChEMBL, respectively. Moreover, the annotations from SRB assays were included in the analysis due to the excellent correlation shown with MTT assays in different studies [35, 36]. With regards to the colorimetric cell-based assays, an important aspect associated with the experimental protocol is the treatment time (i.e*.*, 24 h, 48 h or 72 h), which may lead to different cellular responses for the same drug. In this study, we investigated the experiments with an incubation time of 48 and 72 h, which account for most of the reported activity data from the MTT and SRB assays. The full list of selected ChEMBL ID assays, with their related experimental classification are provided as Additional files 1 and 2 for PC-3 and DU-145, respectively. Table 1 shows the number of compounds in common for the different experimental protocols, on PC-3 and DU-145 cells.

**Table 1:** Number of compounds in common between different experimental protocols for PC-3 and DU-145

PC-3	SRB 48 h	SRB 72 h	MTT 48 h	MTT 72 h	DU-145	SRB 48 h	SRB 72 h	MTT 48 h	MTT 72 h
SRB 48 h	941 (1.0)				SRB 48 h	786 (1.0)			
SRB 72 h	1 (/)	240 (1.0)			SRB 72 h	4 (0.75)	241 (1.0)		
MTT 48 h	6 (0.88)	4 (0.86)	1791 (1.0)		MTT 48 h	10 (0.96)	2 (1.0)	1181 (1.0)	
MTT 72 h	10 (0.96)	10 (0.57)	45 (0.61)	2343 (1.0)	MTT 72 h	9 (0.95)	0 (/)	6 (0.50)	809 (1.0)

The Pearson correlation coefficient is shown in brackets

**Fig. 1:**
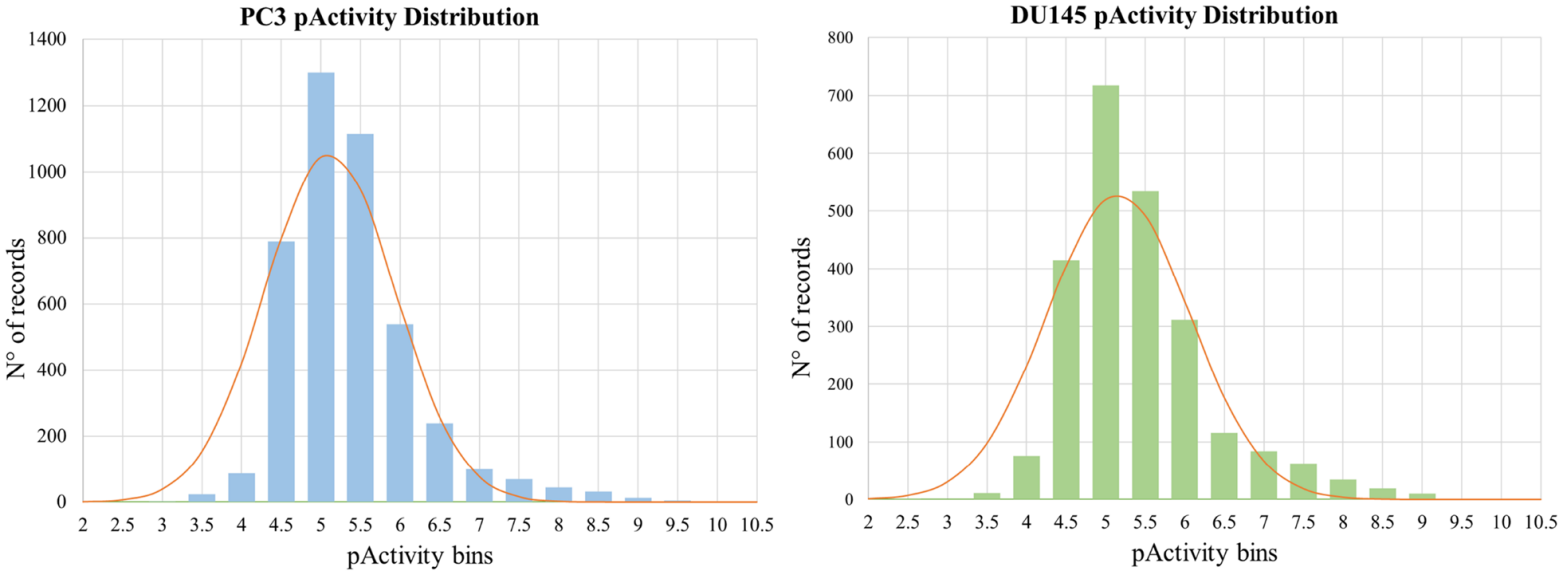
Activity distributions of PC-3 and DU-145 datasets

As can be observed, the majority of the collected molecules have been tested through the MTT (assay time: 72 h) and SRB (assay time: 48 h) protocols, the number of ligands assayed by means of multiple experimental conditions being low. An analysis of the Pearson's correlation coefficients (PCC) on the activity annotations deriving from different MTT and SRB experimental protocols on the same compounds showed moderate to good correlation. For example, annotations from SRB assays (assay time: 48 h) showed a correlation coefficient higher than 0.75, with respect to the other protocols. The inclusion of data deriving from the selected experimental protocols allowed to cover a significantly large chemical space to be explored by the models, while maintaining a high quality of information. Activity records of the collected compounds allowed to cover around 4 logarithmic units of pActivity values, this being a key advantage with respect to the purposes of this study. Duplicated activity records deriving from multiple experiments on the same compounds were filtered, as described in the Methods section. Data related to ligands deriving from the selected antiproliferative assays are reported in the Additional files 3 and 4. Figure 1 shows the distribution of pActivity values for PC-3 and DU-145 datasets. The data on the two tumor cell lines showed similar distributions, the average pActivity values being 5.12 and 5.16 for PC-3 and DU-145, respectively. For both datasets more than 85% of data values ranges between the 4.5 and 6.5. Overall, the adopted approach allowed to collect unique 4353 and 2393 molecules for PC-3 and DU-145, respectively. Interestingly, 587 of the collected compounds present a pActivity value on both the PC-3 and DU-145 PC cells, with an excellent PCC of 0.93 (Additional file 11: Figure S1).

### Activity profiling

**Fig. 2:**
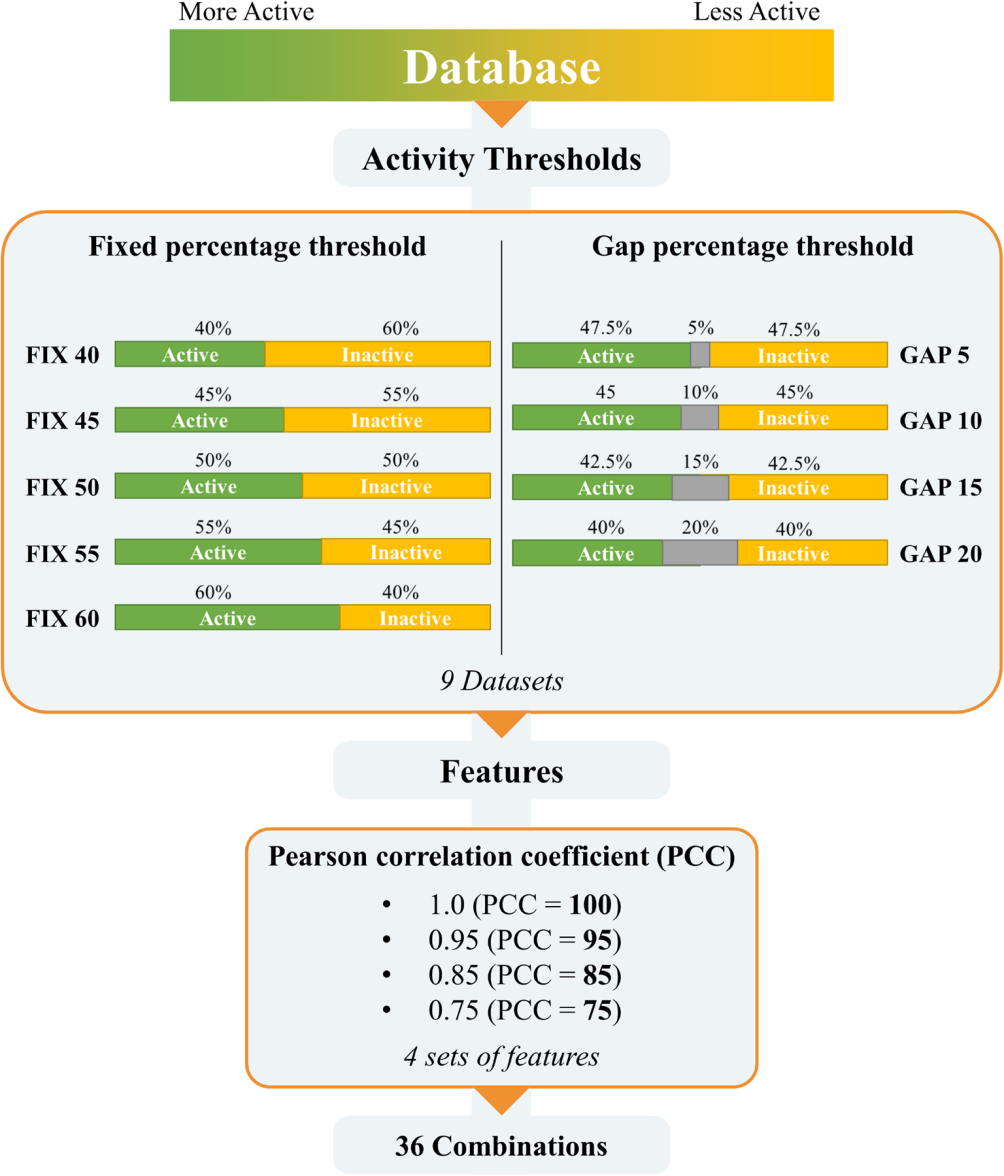
Workflow used to generate the datasets combinations on the two PC-3 and DU-145 cell lines

The first step in the development of machine learning models for the binary classification of active and inactive molecules is labelling the baseline database. In this phase, the compounds are usually classified as active or inactive according to a defined activity threshold, or by using an intermediate window of activity values to better separate the two classes. The selection of the activity thresholds is arbitrarily adapted to the purpose of the model, and on the available activity data. Indeed, the selection of the most suitable activity thresholds is pivotal for the development of highly performant models, as they could reflect in the removal of different number of compounds in the two classes, and thus in the collection of heavily unbalanced training, validation and test datasets. Herein, we explored two different methods for selecting activity thresholds based on activities distribution (Fig. 2). Initially, activity data in the curated PC-3 and DU-145 datasets was sorted by descending pActivity values. In the first method, binary classification was performed by labelling a fixed percentage of the best ranking compounds as active, while considering only the 40–60% of the molecules to avoid the generation of highly unbalanced datasets. The sampling of the databases was performed in incremental steps of 5 percentage points, obtaining 5 different datasets for each cancer cell line. The second approach involved the exclusion of a defined percentage of compounds with intermediate activity data. We gradually excluded the 5–20 percent of the intermediate activity values, by sampling every 5 percentage points. In this case, 4 additional balanced datasets were generated. Table 2 summarize the datasets composition reporting maximum and minimum activities values with the size of sampled classes. Noteworthy, the closest ratio reports the difference in terms of micromolar activity between the last active and the first inactive compounds. As shown, the maximum closest ratio reached is 2.14 for PC-3 and 2.32 for DU-145 database with the GAP 20 method. Since the activities distribution for both databases is highly concentrated around the average, the 20% of intermediate compounds presents a narrow window of activities between 6 and 16 µM. Noteworthy, several studies report anti-cancer compounds with activity against PC-3 and DU-145 in the range of 1–20 µM [37–39], in agreement with the ranges identified for the classification of active and inactive compounds. Of note, the dataset of compounds showed an overall low degree of similarity, despite most of the activity data were distributed in a restricted range of values. In particular, we evaluated the degree of similarity between active and inactive molecules for all the generated datasets according to the Tanimoto coefficent (Tc) [40, 41], which did not indicate any relevant variations for the different datasets (Table 2). Altogether these results suggest that the methods used to select activity thresholds leave the structural similarity between the active and inactive compounds close to an even split on the distribution of activities values (FIX 50 dataset). From the baseline databases, 9 subsets of data were generated by the sampling of activities thresholds. Initially, 123 molecular descriptors were generated for compounds in databases (PCC = 100), as described in the Methods section. Then, to investigate the influence of correlated features on the models’ performance, 3 additional datasets were created by filtering the molecular descriptors with PCC <  = 95, 85 and 75 (Fig. 2). The number of descriptors present in each dataset is shown in Table 2. A total of 36 combinations of activity thresholds and sets of features were obtained for each cancer cell lines and subjected independently to the machine learning workflow described below.

**Table 2:** Number of compounds and activity ranges for PC-3 and DU-145 datasets

		PC-3									DU-145								
Activity Threshold	PCC	N descriptors	Total compounds	*N* active	*N* inactive	*N* Excluded	Max active (µM)	Min inactive (µM)	Closest ratio	Active/inactive mean Tc	N descriptors	Total compounds	N active	N inactive	*N* Excluded	Max active (µM)	Min inactive (µM)	Closest ratio	Active/inactive mean Tc
GAP 5	100	123	4133	2067	2066	220	9.16	11.23	1.23	0.240 ± 0.096	123	2269	1136	1133	124	9.12	11.64	1.28	0.246 ± 0.097
	95	101									102								
	85	82									87								
	75	69									67								
GAP 10	100	123	3919	1962	1957	434	8.40	12.44	1.48	0.240 ± 0.096	123	2151	1076	1075	242	8.33	12.88	1.55	0.246 ± 0.098
	95	101									102								
	85	83									87								
	75	69									67								
GAP 15	100	123	3700	1851	1849	653	7.67	13.53	1.76	0.240 ± 0.096	123	2033	1017	1016	360	7.64	14.09	1.84	0.246 ± 0.098
	95	101									102								
	85	84									86								
	75	68									67								
GAP 20	100	123	3482	1742	1740	871	6.99	14.96	2.14	0.240 ± 0.096	123	1913	957	956	480	6.74	15.64	2.32	0.246 ± 0.098
	95	101									102								
	85	84									86								
	75	68									67								
FIX 40	100	123	4353	1742	2611	–	6.99	7.00	1.00	0.240 ± 0.096	123	2393	957	1436	–	6.74	6.76	1.00	0.246 ± 0.098
	95	101									102								
	85	82									86								
	75	69									67								
FIX 45	100	123	4353	1962	2391	–	8.40	8.41	1.00	0.240 ± 0.096	123	2393	1076	1317	–	8.33	8.35	1.00	0.246 ± 0.098
	95	101									102								
	85	82									86								
	75	69									67								
FIX 50	100	123	4353	2181	2172	–	10.10	10.11	1.00	0.240 ± 0.096	123	2393	1199	1194	–	10.20	10.23	1.00	0.247 ± 0.097
	95	101									102								
	85	82									86								
	75	69									67								
FIX 55	100	123	4353	2395	1958	–	12.40	12.42	1.00	0.239 ± 0.094	123	2393	1316	1077	–	12.81	12.82	1.00	0.247 ± 0.097
	95	101									102								
	85	82									86								
	75	69									67								
FIX 60	100	123	4353	2612	1741	–	14.90	14.92	1.00	0.238 ± 0.094	123	2393	1437	956	–	15.60	15.64	1.00	0.246 ± 0.097
	**95**	101									102								
	**85**	82									86								
	**75**	69									67								

### Machine learning models evaluation

The various generated datasets were subjected to ML workflows with the 10 selected classification algorithms shown in Table 3 (See supplementary information for details).

**Table 3:** Classification algorithms used in this study

ML Algorithm	
1	Logistic Regression (LR)
2	Linear Discriminant Analysis (LDA)
3	K-Nearest Neighbor (KNN)
4	Decision Tree (CART)
5	Naïve Bayes (NB)
6	Support Vector Machine (SVM)
7	Ada Boost (AB)
8	Gradient Boosting (GBM)
9	Random Forest (RF)
10	Extra Trees (ET)

Name abbreviations are shown in brackets

**Fig. 3:**
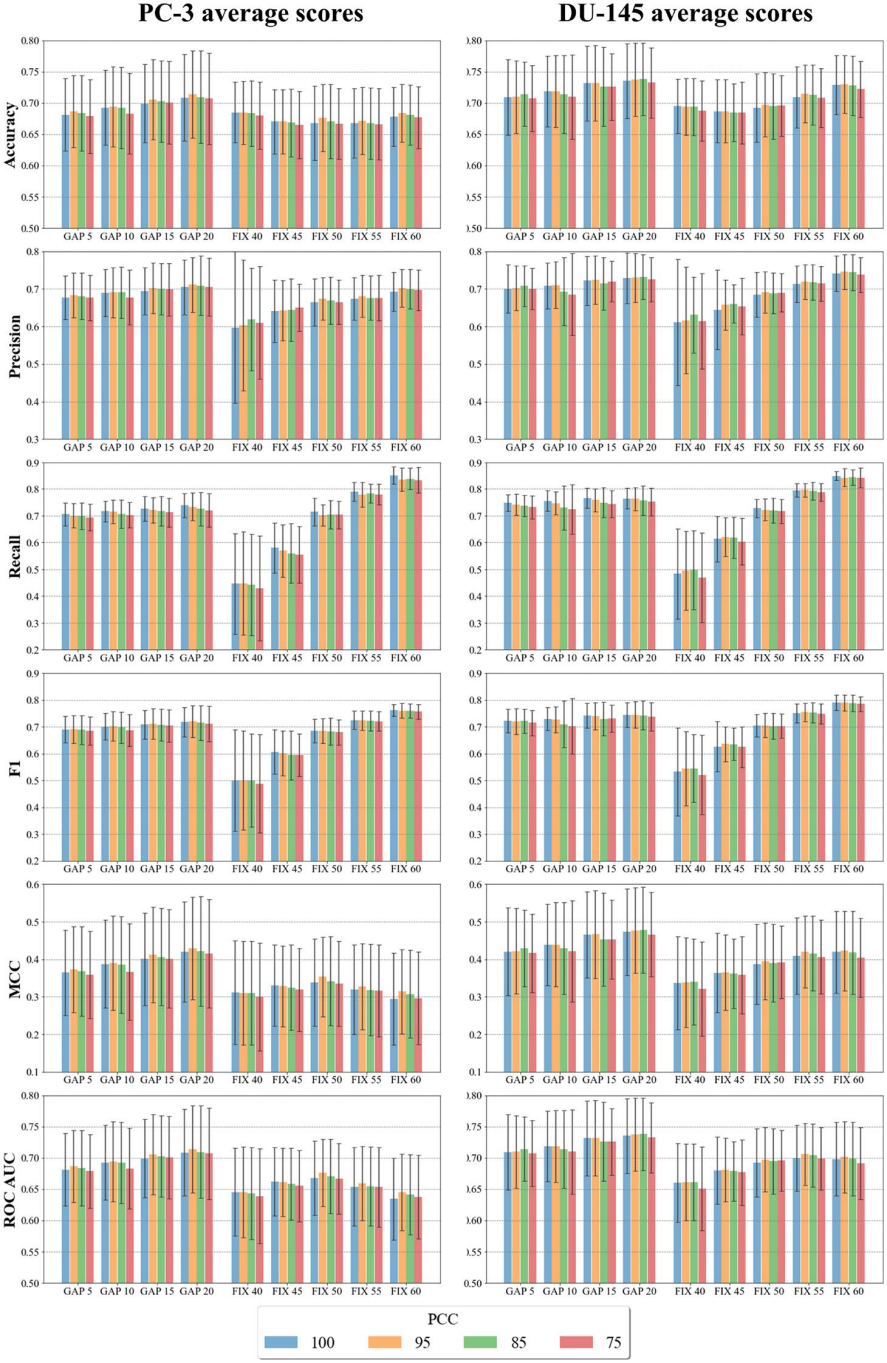
Bar plots of the average scores obtained for the validation set on the 10 ML algorithms. The error bars represent one standard deviation of uncertainty at each data point

Firstly, the molecular similarity of the training and test sets for the 9 datasets were measured according to the ECFP4 molecular fingerprints, scored according to the Tanimoto coefficient. The mean Tc presented a value of 0.23 (Additional file 11: Table S1) for all datasets. The 80th percentile remains below the 0.3 threshold throughout the table, while positive skewness indicates that most of the data are distributed below the mean. The predicted performances of the classifiers are detailed in Additional file 11: Tables S2 and S3 for PC-3 and DU-145, respectively. First, the overall influence of activity thresholds and features selection was evaluated on the mean performance of the 10 ML algorithms on the validation set. The bar plots in Fig. 3 highlight the averaged results of the models according to 6 scoring functions (see Methods for details). Overall, PC-3 and DU-145 showed similar results in terms of prediction performances. Interestingly, the features selection did not affect the average prediction performances of the models, which remained similar despite of the different number of input molecular descriptors. Conversely, the use of different activity thresholds significant impacted on the prediction performances of the models. Indeed, the bar plots highlight trends of varying intensity for the 6 scoring functions (Fig. 3). As expected, the mean precision, recall and F1 scores increased with the fixed percentage of compounds classified as active. These results may be due to the imbalance of positive classes in the datasets, which facilitates the identification of TPs affecting the precision, recall, and consequently F1 scores (See formulas 2, 3 and 4 in Methods). Noteworthy, a trend can also be observed in the validation scores of models from the datasets generated with the GAP threshold method. Interestingly, the introduction of a stepwise gap between activity thresholds provided a small increase in the average precision, recall and F1 score values, while maintaining balanced the classes, when compared with the FIX 50 dataset. A consistent difference between the results from the use of FIX and GAP activity threshold methods can be observed when the MCC score is considered. Indeed, the GAP datasets achieved better averaged predictions with respect to the FIX datasets. Although MCC values showed larger standard deviations, compared to the other scoring functions, the GAP 20 and FIX 50 datasets with PCC = 100 demonstrated statistical differences in their scores, according to the 0.16 and 0.10 *p*-values for PC-3 and DU-145, respectively. The averaged accuracy and ROC AUC did not reveal significant changes for the different activity thresholds and range around 0.75 for both PC-3 and DU-145 datasets. Altogether, the comparison of the results from the developed models showed that the GAP method can provide advantages in the prediction performances compared to the use of a single threshold. Noteworthy, similar conclusions were also reported in previous studies focused on target activity data (*e.g.*, [42]).With respect to the different classifiers, we analyzed the performance of individual ML algorithms to identify the best predictive models. The performances of the models were evaluated on the validation set.

**Fig. 4:**
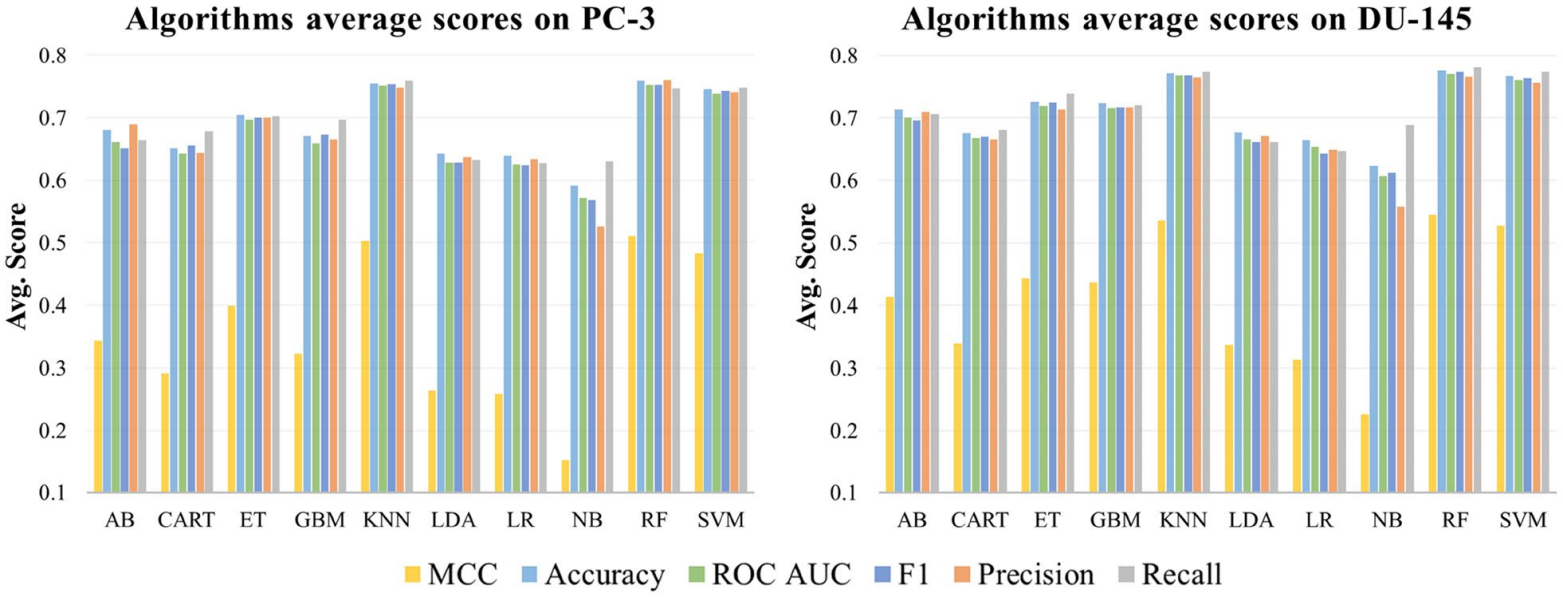
Average prediction performances of the 10 algorithms on PC-3 and DU-145 datasets. The average of the six scores was calculated over the 36 combinations of the 9 datasets with the different number of features

Figure 4 shows the mean validation set results obtained on the different datasets for the 10 classification algorithms. Overall, the KNN, SVM and RF algorithms outperformed the others in both PC-3 and DU-145 data sets. Indeed, the KNN algorithm applied to the GAP 20 dataset demonstrated the highest MCC and accuracy values on both cell lines. In particular, KNN achieved a MCC score higher than 0.6 for the GAP 20 dataset with PCC = 95. Moreover, MCC values above 0.56 were also obtained for SVM and RF on the same datasets. On average, the ML models generated on the GAP 20 dataset led to the highest prediction performances, as shown in Fig. 3. Moreover, the results demonstrated consistent F1 values on training, validation and test sets, confirming a suitable fitting of the datasets on all analyzed models. Based on these results, the GAP 20 method was identified as the best approach to classify active and inactive compounds of the initial dataset.

As a further validation of the protocol, we simulated real case studies by iteratively using a sample of compounds deriving by the same ChEMBL assay as test set. In particular, to achieve acceptable statistical significance, we tested the model on PC-3 and DU-145 cell assays containing 30 or more activity records. Then, the compounds related to the ChEMBL Documents IDs in Table 4 were iteratively extracted from the initial datasets and in turn were randomly split into training and validation sets with a ratio of 75:25. The combination of RF algorithm, GAP 20 activity threshold and PCC = 95 was found to be the most suitable for the models development, given the excellent performances obtained in previous tests. A total of six studies were tested for PC-3 and three for DU-145. For the models so developed on both cancer cell lines, we obtained an accuracy below 0.6 (Table 4) in only one cases, with those deriving from CHEMBL4155048- and CHEMBL4686002-related assays demonstrating excellent performances in the identification of almost all active or inactive compounds. On average, the models provided relevant predictions for the identification of active compounds. Based on the composition of the training set and the limited amount of information provided by the molecular descriptors, it is possible that some classes of compounds may be challenging for the model to classify.

**Table 4:** Prediction results on isolated ChEMBL assays

Assay ChEMBL ID	N compounds	N active	N inactive	Accuracy	TN	FP	FN	TP	TNR	FPR	FNR	TPR
PC3												
CHEMBL4155048 [43]	33	33	0	0.94	0	0	2	31	–	–	0.06	0.94
CHEMBL1840268 [44]	34	28	6	0.71	1	5	5	23	0.17	0.83	0.18	0.82
CHEMBL3097567 [45]	38	0	38	0.63	24	14	0	0	0.63	0.37	–	–
CHEMBL2040207 [46]	44	31	13	0.41	9	4	22	9	0.69	0.31	0.71	0.29
CHEMBL3062059 [47]	47	24	23	0.77	20	3	8	16	0.87	0.13	0.33	0.67
CHEMBL3788954 [48]	64	23	41	0.63	33	8	16	7	0.80	0.20	0.70	0.30
DU145												
CHEMBL4686002 [49]	30	0	30	0.90	27	3	0	0	0.90	0.10	–	–
CHEMBL4158478 [50]	38	26	12	0.69	6	6	6	20	0.50	0.50	0.23	0.77
CHEMBL2406593 [51]	50	0	50	0.28	14	36	0	0	0.28	0.72	–	–

RF classifiers (GAP 20, PCC = 95) results on the external test set consisting of the compounds from a single ChEMBL assay for PC3 and DU145. Reference articles of each assay are added in brackets

### Models developed on combined activity data

Interestingly, several compounds of the curated datasets have reported activity data for both PC-3 and DU-145 cancer cell lines (*i.e*., 587 molecules), showing excellent correlation (PCC = 0.93, Additional file 11: Figure S1). Therefore, we evaluated weather a machine learning model developed on the combination of data reported for PC-3 and DU-145 would achieve higher prediction performances with respect to the models previously derived on the two cancer cell lines. To this aim, the 587 compounds with known pActivity values on PC-3 and DU-145 were used as test set, while the remaining 5572 compounds from the two datasets were used for the training and validation of the models (see the Methods section for details). These analyses were conducted by using SVM, KNN, and RF algorithms in combination with the GAP 20 activity thresholds, which showed the best predictions performances (Additional file 11: Tables S2 and S3), and FIX 50 as comparison set. Additionally, only the features with PCC values below 0.95 (*i.e.*, PCC = 95) were retained in the datasets. Table 4 shows the results obtained from the combined PC-3/DU-145 models on the test set. Interestingly, the resulting performance of prediction resulted similar for the two cancer cell lines in the test dataset, albeit the training set significantly biased towards PC-3 compounds (3766 ligands for PC-3 *vs* 1806 for DU-145).

The models developed on a training dataset generated with the GAP 20 activity threshold overall achieved better classification performances with respect to those obtained with FIX 50 (Table 5). In particular, the MCC score values are consistently higher using the GAP 20 method for the three ML algorithms, even though all classifiers demonstrate good precision. The SVM GAP 20 model achieved a precision equal to 0.84 and 0.83 on PC-3 and DU-145, respectively, presenting a low ratio of false positives (FPR), compared to the true positives ratio (TPR). However, all the newly generated models showed false negative ratio (FNR) of around 0.4, which negatively affected their overall prediction performances (see MCC and F1 scores in Table 4). Overall, the models developed on the combination of PC-3 and DU-145 data produced classification models with high predictivity towards TPs. In order to compare the performance of the combined models with the classifiers developed on the individual cell line datasets, six additional classification models were trained and tested on the 587 compounds presenting PC-3 and DU-145 activity values. The training and validation of the specific models was carried out on the individual datasets of PC-3 and DU-145 using the same procedure used for the combined models. The results showed minor differences between the combined and specific models (Additional file 11: Figure S2). In particular, the combined models showed generally higher MCC and precision values while the remaining scores were comparable across the different datasets.

**Table 5:** Results on the test set for the ML models trained and validated on the combined PC-3/DU-145 dataset (PCC = 95)

Cell line	PC-3						DU-145					
Algorithm	SVM		RF		KNN		SVM		RF		KNN	
Activity threshold method	GAP 20	FIX 50	GAP 20	FIX 50	GAP 20	FIX 50	GAP 20	FIX 50	GAP 20	FIX 50	GAP 20	FIX 50
*N* compounds	461	587	461	587	461	587	485	586	485	586	485	586
*N* active	286	351	286	351	286	351	286	336	286	336	286	336
*N* inactive	175	236	175	236	175	236	199	250	199	250	199	250
Accuracy	0.65	0.65	0.67	0.65	0.64	0.63	0.66	0.66	0.67	0.65	0.64	0.63
Precision	0.85	0.82	0.81	0.74	0.80	0.73	0.83	0.79	0.78	0.72	0.74	0.71
Recall	0.54	0.53	0.61	0.63	0.57	0.58	0.54	0.54	0.61	0.63	0.58	0.59
F1	0.66	0.64	0.69	0.68	0.67	0.65	0.65	0.64	0.69	0.67	0.65	0.65
MCC	0.38	0.36	0.36	0.30	0.33	0.27	0.38	0.36	0.36	0.29	0.29	0.27
ROCAUC	0.69	0.68	0.68	0.65	0.67	0.64	0.69	0.68	0.68	0.65	0.65	0.64
TNR	0.84	0.82	0.76	0.68	0.76	0.69	0.84	0.81	0.75	0.66	0.71	0.68
FPR	0.16	0.18	0.24	0.32	0.24	0.31	0.16	0.19	0.25	0.34	0.29	0.32
FNR	0.46	0.47	0.39	0.37	0.43	0.42	0.46	0.46	0.39	0.37	0.42	0.41
TPR	0.54	0.53	0.61	0.63	0.57	0.58	0.54	0.54	0.61	0.63	0.58	0.59

Overall, the combination of the two databases appears to marginally contribute to the ability of the models to generalize predictions. These results demonstrate that the development of ML models based on combined data of the cancer cells is feasible and could provide good classification performances of the active compounds on the dataset of ligands with activity on PC-3 and DU-145.

### Biological targets analysis

**Fig. 5:**
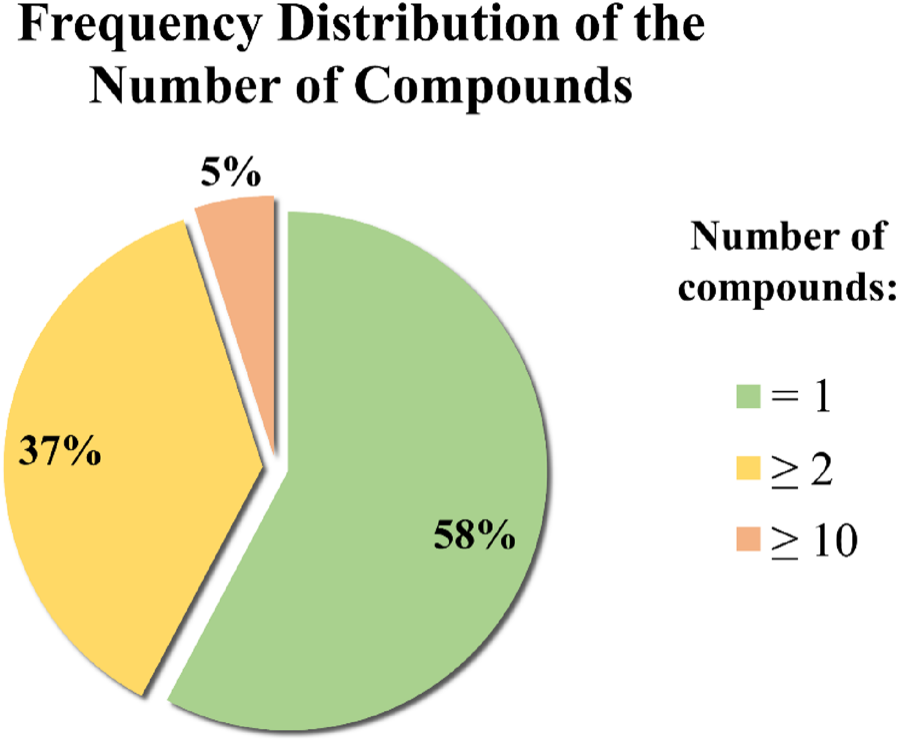
Pie chart showing the relative frequency of the number of compounds associated with biological targets. The frequencies were calculated using the data in Additional file 11: Table S4 considering PC-3 and DU-145 separately

The generated models were able to classify PCa cellular activities without taking into consideration information related to biological targets. In our datasets, the compounds rarely resulted to be investigated on more than 3 biological targets. Indeed, the compounds present an average of 2.4 activity annotations on a total of 169 targets for the PC-3 data set, while we found an average of 3 records *per* molecule on 83 different proteins for DU-145. From the viewpoint of the targets, the relative frequency of the number of active compounds is shown in Fig. 5. More than half of the targets present a single associated active compound, and 5% of targets are associated with at least 10 different active molecules. The high variability in terms of type and number of targets with reported activity annotations for the compounds make this information not easy to be integrated into the ML models. However, the investigation of targets associated to molecules active on PCa cells can provide further insights into the models’ application. Indeed, the information collected on biological targets (Additional file 11: Table S4) could provide useful clues for the search of active compounds. For example, molecules with activity on known PCa-related biological targets stand a greater chance of exhibiting antiproliferative effects against prostate cancer cell lines. Moreover, such information could also be of help to clarify the mechanism of action of compounds with antiproliferative PCa activity. An analysis of the activity records from the curated sets of compounds showed that 94 and 44 of the 213 identified targets are exclusively tested on ligands of the PC-3 and DU-145 datasets, respectively. Moreover, only 47 of the identified targets have a known association with prostate cancer (Additional file 11: Table S4), according to information reported in the UniProt [52] (https://www.uniprot.org/) and Therapeutic Target Database (TTD, http://db.idrblab.net/ttd/) [53] databases. The information related to these targets could be of particular interest while designing compounds with PCa antiproliferative activity. Several compounds with antiproliferative activity against PC-3 cell line present target annotations in common with DU-145, albeit the number of ligands with target annotations and activity towards the latter cell line is significantly lower, this probably deriving also by the different size of the curated datasets. Among them we found Histone deacetylases (HDACs). In particular, 71 compounds with activity towards PC-3 showed HDAC1 inhibition in the nanomolar range. Indeed, HDACs represent established biological targets for PCa, with four HDACs inhibitors being approved for the treatment of different types of cancer. Moreover, Heat shock protein 90 (Hsp90) also emerged as one of the targets with the highest number of associated activity records on DU-145. Hsp90 is a chaperone whose activity is associated with the correct function of several, fundamental processes in cells, including proliferation, survival, differentiation and apoptosis. Moreover, several studies have also extensively discussed its involvement in prostate cancer and other types of tumors [54–56]. Other established PCa-related targets, such as Tubulin [57], PI3K [58], mTOR [59] have also been spotted among the compounds-biological target associations. Besides, a number of activity annotations on targets apparently unrelated to PCa, yet potentially relevant to pathology development and progression, or deriving by polypharmacology efforts, have also been observed, which might be of high interest for future drug discovery efforts.

## Conclusions

In this study, we developed a series of machine learning models for the classification of active and inactive compounds towards PC-3 and DU-145 prostate cancer cell lines. The data employed for the development of the models was obtained from ChEMBL, exclusively selecting activity values that derived by colorimetric antiproliferative assays with well-defined and consistent experimental protocols. Two different methods were explored for the sampling of the active and inactive compounds. In particular, the compounds in the datasets were classified according to different activity thresholds, or by excluding progressively increasing percentages of ligands with intermediate activity. Moreover, different combinations of molecular descriptors were investigated as input features, filtering out those with the highest correlation. For each cancer cell line, 9 datasets with 4 different sets of features were subjected to a model development workflow with 10 ML different algorithms, evaluating their prediction performances. The results of these analyses showed that the use of datasets generated through the elimination of intermediate records provided improved prediction performances with respect to those generated by the use of a single activity threshold. Removal of highly correlating features provided minor effects on the overall prediction performances. Of the 10 ML algorithms, SVM, RF and KNN demonstrated the best classification performances on the validation and test sets. Moreover, our analyses allowed to identify a number of compounds in the datasets with activity values that were significantly correlated between PC-3 and DU-145 cancer cells. Afterwards, we simulated real case scenarios by testing the best combination of algorithm, activity threshold, and sets of features, on a series of selected ChEMBL assay excluded from the training set. Remarkably, the models performed well on average with an accuracy above 0.6 in most of the samples. This result inspired us to investigate whether classifiers trained on the combination of the PC-3 and DU-145 datasets could provide better prediction performances with respect to those obtained on single cell lines. Interestingly, the generated PC-3/DU-145 combined models showed excellent precision on the test set. If compared with the individuals PC-3 and DU-145 models, training classification models on compounds of both cell lines led to a marginal improvement in their prediction performances in terms of precision. Finally, we investigated the biological targets associated to the most active compounds reported for the PC-3 and DU-145 cell lines in the curated datasets, around 25% of them having already reported associations to prostate cancer. Besides, a number of targets not directly associated to PCa have also been identified, representing valuable information for future investigation on the mode of action of ligands showing potent antiproliferative activity against PC-3 and DU-145 cell lines.

Altogether, the analyses carried out in this study provided a valuable workflow for the development of ML models able to predict the activity on PC-3 and DU-145 prostate cancer cell lines. The best-performing models can be implemented into fast and cost-effective virtual screening protocols able to identify novel hit candidates to be tested against prostate cancer cells. All the data sets and models discussed in this work is made available as additional files to the research community.

## Methods

### Dataset preparation

Functional assays related to the PC-3 and DU-145 cell lines were firstly obtained from the ChEMBL database [26, 27] (release 29, accessed on February 2022). Then, activity records were filtered to retain only those containing combinations of the keywords ("MTT" OR "SRB"), AND ("48 h" OR "72 h") in the assays description field of the data. A further selection of the assays was carried out manually according to custom parameters to remove duplicates and records without the desired experimental features. The full list of PC-3 and DU-145 cell-based assays can be found in Additional files 1 and 2, respectively. Subsequently, the compounds associated with the selected assays were integrated with cell activity data. Activity records were filtered to retain only values with reported standard relation type corresponding to “ = ”, standard units equal to “nM” and standard type corresponding to “IC50” or “GI50”. The activity values were converted into the logarithmic scale (*i.e.*, pActivity calculated on the −log_10_ of the corresponding IC_50_ or GI_50_). For compounds with multiple activity records were filtered as follows. The range of pActivity were firstly computed, and then compounds with a range of values less than or equal to 1, their average was calculated and used as a reference. Conversely, compounds with a range of pActivity values higher 1 were removed from the dataset. An additional filter was applied to the molecular weight (MW), retaining only compounds between 100 and 600 Da. For each compound 123 molecular descriptors were calculated using RDKit modules implemented in python [60]. Afterwards, the resulting compounds in the PC-3 and DU-145 databases were labelled as active or inactive according to the fixed percentage and the gap percentage methods (Fig. 2). In particular, the pActivities of the compounds were firstly sorted in ascending order. Then, five different classifications were applied to the database by labelling the first 40%, 45%, 50%, 55%, 60% of the compounds as active and the remaining molecules as inactive (Fig. 2), for the fixed percentage method. Besides, additional four different classifications were applied through the exclusion of 5%, 10%, 15%, 20% of records equally distributed around the median of the activity values (Fig. 2) for the gap percentage method. For each of the nine subsamples of data, a procedure was subsequently implemented in order to remove redundant features. In particular, we investigated the matrix of Pearson Correlation Coefficient values calculated by using the numpy python function corr(). Moreover, three additional subsets were obtained by selecting features with a PCC below 0.95 (PCC = 95), 0.85 (PCC = 85) and 0.75 (PCC = 75). Overall, 36 subsets of data were generated for each cancer cell lines by means of this approach.

### Models development and evaluation

The analyses were performed with the *scikit-learn* modules implemented in python [61]. In particular, each dataset was divided into training, validation and test, according to the 50:25:25 ratio using the stratified random sampling to maintain balanced classes. A fourfold cross-validation methodology was applied prior to the models development in order to increase the robustness of the method while maintaining an efficient amount of data for each set. Four stratified subsets of data were generated in this phase, 2 of them being used to train the model, and the other two for the validation and test phases, respectively, for each iteration. Twelve independent iterations were performed for each dataset and the values obtained were averaged in the results. Ten machine learning algorithms were implemented in the development of the models (Table 3). In the first step, models were developed on the training set using the GridSearchCV module implemented in python to tune the hyper-parameters in a fivefold cross-validation procedure. The parameters used with the ML algorithms are reported in the Additional file 10. The F1 scoring function was used for the parameters search. Then, a probability calibration on the classifier was performed by using the validation set. The performances of prediction obtained on the validation set were used to identify the best models. Finally, the model predictions were evaluated on the test set. Six different metrics, *i.e.*, *accuracy (1)*, *precision (2)*, *recall (3), F1 (4), Matthews Correlation Coefficient* (*MCC*) *(5)*, *Area Under the Receiver Operating Characteristic Curve* (*ROC AUC*) were employed to assess models’ performance in the validation and test phases. The evaluation metrics are defined as follows:

1 $$Accuracy= \frac{TP+TN}{TP+TN+FP+FN}$$

2 $$Precision= \frac{TP}{TP + FP}$$

3 $$Recall= \frac{TP}{TP + FN}$$

4 $$F1= \frac{2\times (precision \times recall)}{precision + recall}$$

5 $$MCC= \, \frac{TP\times TN-FP\times FN}{\sqrt{(TP+FP)(TP+FN)(TN+FP)(TN+FN)}}$$

6 $$TNR= \frac{TN}{TN+FP}$$

7 $$FPR= \frac{FP}{TN+FP}$$

8 $$FNR= \frac{FN}{TP+FN}$$

9 $$TPR= \frac{TP}{TP+FN}$$

### Combined PC-3/DU-145 models

The combined PC-3/DU-145 models were trained, validated and testes as follows. The previously curated PC-3 and DU-145 datasets were firstly merged, excluding 587 compounds with activity data on both the cell lines. In particular, 3766 compounds with PC-3 pActivity values and the 1806 ligands with DU-145 pActivity values were combined to develop the PC-3/DU-145 dataset with 5572 molecules. Then, the dataset was split with a ratio of 75:25, to obtain the training and validation sets, respectively. The compounds previously excluded due to their reported antiproliferative activity towards PC-3 and DU-145 cells were used as a test set. A dual classification of the compounds was performed based on the activity data of PC-3 and DU-145 in the test set. In the models development phases, active and inactive compounds were classified according to the FIX 50 and GAP 20 methods, while only non-correlating features, *i.e.*, with PCC value lower than 0.85 (PCC = 85) were retained. The optimization of hyperparameters for the RF, SVM and KNN classifiers was performed on the training set with the GridSearchCV, by setting a fivefold cross validation. The probability of the models’ predictions was calibrated on the validation set. Finally, the prediction performances of the models were evaluated on the test set, for both the PC-3 and DU-145 classes.

### Biological target selection

The analysis of biological targets was focused on the PC-3 and DU-145 active compounds present in the GAP 20 datasets. The compound-target association were retrieved from ChEMBL, retaining only records with standard relation corresponding to “ = ” and standard units equal to “nM”. Moreover, only binding assays on human organism and compounds with reported IC_50_ values lower or equal than 1000 nM on the targets were considered. Compounds as Staurosporine (CHEMBL388978) were excluded from the analyses due to its high binding promiscuity [62]. This approach allowed to detect a total of 213 biological targets. Then, the detected targets were compared with information related to prostate cancer present in the UniProt and TTD databases. In particular, a total of 1069 targets were obtained from UniProt by applying the filter keywords “prostate cancer” and “human” for the organism of origin, and filtering only reviewed results. From TTD, biological targets with associated diseases were extracted and filtered using the keyword “prostate cancer”, obtaining 87 additional targets. Duplicate records emerging from the databases were removed, obtaining a list of 1156 prostate cancer related biological targets (data available as Additional file 5). Finally, the UniProt IDs were mapped with the target ChEMBL IDs associated to the ligands from PC-3 and DU-145 GAP 20 datasets, 47 of the 213 detected biological targets being associated to PCa (Additional file 11: Table S4).

## Supplementary Information

**Additional file 1.** Table with PC-3 ChEMBL assays used to retrieve compounds activities

**Additional file 2.** Table with DU-145 ChEMBL assays used to retrieve compounds activities

**Additional file 3.** PC-3 initial dataset with the 123 RDKit descriptors calculated

**Additional file 4.** DU-145 initial dataset with the 123 RDKit descriptors calculated

**Additional file 5.** Table with UniProt ID associated with prostate cancer from UniProt and TTD

**Additional file 6.** Python script to generate the initial PC-3, DU-145 and PC-3/DU-145 datasets

**Additional file 7.** Python script to perform models training, validation and testing phase

**Additional file 8.** Python script to perform training and testing phase of the combined PC-3/DU-145 models

**Additional file 9.** Python script to retrieve compounds-target relationship

**Additional file 10.** Python script to setup algorithms input parameters for the hyperparameter tuning with *GridSearchCV*

**Additional file 11.** Additional tables and figures.

## Data Availability

The datasets supporting the conclusions of this article are included within the article (and its additional files) and at the following link: https://ligadvisor.unimore.it/downloads.

## Abbreviations

AB: Ada Boost
ADT: Androgen Deprivation Therapy
AR: Androgen Receptor
CART: Decision Tree
CRPC: Castrate-resistant prostate cancer
ET: Extra Trees
FN: False Negative
FNR: False Negative Ratio
FP: False Positive
FPR: False Positive Ratio
GBM: Gradient Boosting
GI_50_: Half maximal growth inhibition concentration
Hsp90: Heat shock protein 90
IC_50_: Half maximal inhibitory concentration
KNN: K-Nearest Neighbor
LDA: Linear Discriminant Analysis
LHRH: Luteinizing hormone-releasing hormone
LR: Logistic Regression
MCC: Matthews Correlation Coefficient
ML: Machine Learning
MoA: Mechanism of Action
MTT: 3-(4,5-Dimethylthiazol-2-yl)-2,5-diphenyltetrazolium bromide
MW: Molecular Weight
NB: Naïve Bayes
PCa: Prostate Cancer
PCC: Pearson Correlation Coefficient
RF: Random Forest
ROC AUC: Area Under the Receiver Operating Characteristic Curve
SVM: Support Vector Machine
SRB: Sulforhodamine B
TBDD: Target-based drug discovery
Tc: Tanimoto Coefficient
TN: True Negative
TNR: True Negative Ratio
TP: True Positive
TPR: True Positive Ratio
TTD: Therapeutic Target Database
