# Supplementary material for: Development of machine learning classifiers to predict compound activity on prostate cancer cell lines
Source: J Cheminform. 2022 Nov 8;14:77. doi: 10.1186/s13321-022-00647-y (PMC9641853; doi:10.1186/s13321-022-00647-y)
Supplement: Supplementary file 11 — Additional file 11. Additional tables and figures. [file 13321_2022_647_MOESM11_ESM.docx]

***Supporting Information***

**Development of Machine Learning Classifiers to Predict Compound Activity on Prostate Cancer Cell Lines**

Davide Bonanni, Luca Pinzi, Giulio Rastelli*

*Department of Life Sciences, University of Modena and Reggio Emilia.*

*Via Campi 103, 41125 Modena, Italy*

**Table of Contents**

**Tables**

| Table S1 | Pag. S3 |
| --- | --- |
| Table S2 | Pag. S4 |
| Table S3 | Pag. S22 |
| Table S4 | Pag. S40 |

**Figures**

| Figure S1 | Pag. S44 |
| --- | --- |
| Figure S2 | Pag. S44 |

**Machine Learning Algorithms explored in the study** Pag. S45

**References** Pag. S47

**Table S1.** Data reported on the distribution of identified similarity values between training and test sets for the respective datasets. The similarity values were calculated for the ECFP4 fingerprints.

| **Cell line** | **Activity threshold** | **Mean Tc** | **Standard deviation** | **Median** | **20° Percentile** | **80° Percentile** | **skewness** |
| --- | --- | --- | --- | --- | --- | --- | --- |
| **PC-3** | GAP 5 | 0.233 | 0.091 | 0.229 | 0.167 | 0.295 | 1.09 |
|  | GAP 10 | 0.233 | 0.092 | 0.229 | 0.167 | 0.296 | 1.02 |
|  | GAP 15 | 0.233 | 0.091 | 0.229 | 0.167 | 0.295 | 1.08 |
|  | GAP 20 | 0.233 | 0.092 | 0.227 | 0.167 | 0.295 | 1.07 |
|  | FIX 40 | 0.234 | 0.091 | 0.229 | 0.167 | 0.295 | 1.13 |
|  | FIX 45 | 0.234 | 0.091 | 0.229 | 0.167 | 0.297 | 1.09 |
|  | FIX 50 | 0.235 | 0.090 | 0.229 | 0.170 | 0.295 | 1.14 |
|  | FIX 55 | 0.234 | 0.091 | 0.229 | 0.167 | 0.295 | 1.11 |
|  | FIX 60 | 0.234 | 0.090 | 0.229 | 0.167 | 0.295 | 1.11 |
|  |  |  |  |  |  |  |  |
| **DU-145** | GAP 5 | 0.237 | 0.095 | 0.229 | 0.170 | 0.297 | 1.53 |
|  | GAP 10 | 0.235 | 0.094 | 0.229 | 0.167 | 0.295 | 1.44 |
|  | GAP 15 | 0.237 | 0.094 | 0.229 | 0.170 | 0.295 | 1.53 |
|  | GAP 20 | 0.232 | 0.095 | 0.226 | 0.167 | 0.293 | 1.46 |
|  | FIX 40 | 0.238 | 0.095 | 0.231 | 0.171 | 0.298 | 1.54 |
|  | FIX 45 | 0.236 | 0.094 | 0.229 | 0.169 | 0.295 | 1.47 |
|  | FIX 50 | 0.238 | 0.095 | 0.231 | 0.170 | 0.298 | 1.51 |
|  | FIX 55 | 0.237 | 0.093 | 0.229 | 0.170 | 0.295 | 1.54 |
|  | FIX 60 | 0.237 | 0.095 | 0.229 | 0.170 | 0.297 | 1.52 |

**Table S2.** Results of the machine learning models developed on data related to the PC-3 cell line.

|  |  |  |  | **Validation set** | | | | | | **Test set** | | | | | | |  |
| --- | --- | --- | --- | --- | --- | --- | --- | --- | --- | --- | --- | --- | --- | --- | --- | --- | --- |
| **Activity threshold** | **PCC** | **Algorithm** | **F1 train** | **F1** | **MCC** | **ROC AUC** | **Accuracy** | **Precision** | **Recall** | | **F1** | **MCC** | **ROC AUC** | **Accuracy** | **Precision** | **Recall** | |
| GAP 5 | 75 | AB | 0.68 ± 0.02 | 0.68 ± 0.03 | 0.36 ± 0.04 | 0.68 ± 0.02 | 0.68 ± 0.02 | 0.68 ± 0.02 | 0.68 ± 0.06 | | 0.68 ± 0.03 | 0.36 ± 0.03 | 0.68 ± 0.02 | 0.68 ± 0.02 | 0.68 ± 0.02 | 0.68 ± 0.06 | |
|  |  | CART | 0.67 ± 0.01 | 0.64 ± 0.02 | 0.29 ± 0.04 | 0.65 ± 0.02 | 0.65 ± 0.02 | 0.65 ± 0.02 | 0.64 ± 0.03 | | 0.64 ± 0.02 | 0.29 ± 0.04 | 0.65 ± 0.02 | 0.65 ± 0.02 | 0.65 ± 0.02 | 0.64 ± 0.04 | |
|  |  | ET | 0.68 ± 0.01 | 0.7 ± 0.01 | 0.39 ± 0.02 | 0.7 ± 0.01 | 0.7 ± 0.01 | 0.69 ± 0.01 | 0.7 ± 0.02 | | 0.7 ± 0.01 | 0.39 ± 0.02 | 0.7 ± 0.01 | 0.7 ± 0.01 | 0.69 ± 0.01 | 0.7 ± 0.02 | |
|  |  | GBM | 0.66 ± 0.01 | 0.66 ± 0.02 | 0.33 ± 0.03 | 0.66 ± 0.02 | 0.66 ± 0.02 | 0.66 ± 0.01 | 0.66 ± 0.02 | | 0.66 ± 0.02 | 0.33 ± 0.03 | 0.66 ± 0.01 | 0.66 ± 0.01 | 0.66 ± 0.02 | 0.66 ± 0.02 | |
|  |  | KNN | 0.74 ± 0.01 | 0.75 ± 0.02 | 0.5 ± 0.04 | 0.75 ± 0.02 | 0.75 ± 0.02 | 0.75 ± 0.02 | 0.75 ± 0.02 | | 0.75 ± 0.02 | 0.5 ± 0.04 | 0.75 ± 0.02 | 0.75 ± 0.02 | 0.75 ± 0.02 | 0.75 ± 0.02 | |
|  |  | LDA | 0.62 ± 0.02 | 0.62 ± 0.02 | 0.25 ± 0.04 | 0.63 ± 0.02 | 0.63 ± 0.02 | 0.63 ± 0.02 | 0.62 ± 0.02 | | 0.62 ± 0.02 | 0.25 ± 0.03 | 0.63 ± 0.02 | 0.63 ± 0.02 | 0.63 ± 0.02 | 0.62 ± 0.02 | |
|  |  | LR | 0.62 ± 0.02 | 0.62 ± 0.01 | 0.26 ± 0.03 | 0.63 ± 0.01 | 0.63 ± 0.02 | 0.63 ± 0.02 | 0.62 ± 0.01 | | 0.63 ± 0.01 | 0.26 ± 0.03 | 0.63 ± 0.02 | 0.63 ± 0.02 | 0.63 ± 0.02 | 0.62 ± 0.02 | |
|  |  | NB | 0.65 ± 0.01 | 0.62 ± 0.02 | 0.19 ± 0.04 | 0.59 ± 0.02 | 0.59 ± 0.02 | 0.58 ± 0.02 | 0.67 ± 0.02 | | 0.62 ± 0.02 | 0.18 ± 0.04 | 0.59 ± 0.02 | 0.59 ± 0.02 | 0.58 ± 0.02 | 0.66 ± 0.02 | |
|  |  | RF | 0.75 ± 0.01 | 0.75 ± 0.02 | 0.5 ± 0.03 | 0.75 ± 0.02 | 0.75 ± 0.02 | 0.76 ± 0.02 | 0.74 ± 0.01 | | 0.75 ± 0.02 | 0.5 ± 0.03 | 0.75 ± 0.02 | 0.75 ± 0.02 | 0.76 ± 0.02 | 0.74 ± 0.02 | |
|  |  | SVM | 0.74 ± 0.02 | 0.75 ± 0.02 | 0.5 ± 0.04 | 0.75 ± 0.02 | 0.75 ± 0.02 | 0.75 ± 0.02 | 0.75 ± 0.02 | | 0.75 ± 0.02 | 0.5 ± 0.04 | 0.75 ± 0.02 | 0.75 ± 0.02 | 0.75 ± 0.02 | 0.75 ± 0.02 | |
|  | 85 | AB | 0.68 ± 0.02 | 0.67 ± 0.02 | 0.32 ± 0.1 | 0.66 ± 0.05 | 0.66 ± 0.05 | 0.66 ± 0.06 | 0.71 ± 0.07 | | 0.67 ± 0.02 | 0.32 ± 0.1 | 0.66 ± 0.05 | 0.66 ± 0.05 | 0.66 ± 0.06 | 0.71 ± 0.07 | |
|  |  | CART | 0.67 ± 0.01 | 0.65 ± 0.02 | 0.3 ± 0.04 | 0.65 ± 0.02 | 0.65 ± 0.02 | 0.65 ± 0.02 | 0.65 ± 0.03 | | 0.65 ± 0.02 | 0.3 ± 0.04 | 0.65 ± 0.02 | 0.65 ± 0.02 | 0.65 ± 0.02 | 0.65 ± 0.03 | |
|  |  | ET | 0.69 ± 0.01 | 0.7 ± 0.02 | 0.41 ± 0.03 | 0.71 ± 0.02 | 0.71 ± 0.02 | 0.7 ± 0.02 | 0.71 ± 0.02 | | 0.7 ± 0.02 | 0.41 ± 0.03 | 0.71 ± 0.02 | 0.71 ± 0.02 | 0.7 ± 0.02 | 0.71 ± 0.02 | |
|  |  | GBM | 0.67 ± 0.01 | 0.68 ± 0.01 | 0.36 ± 0.03 | 0.68 ± 0.01 | 0.68 ± 0.01 | 0.68 ± 0.02 | 0.68 ± 0.02 | | 0.68 ± 0.02 | 0.36 ± 0.03 | 0.68 ± 0.02 | 0.68 ± 0.02 | 0.68 ± 0.02 | 0.69 ± 0.02 | |
|  |  | KNN | 0.75 ± 0.01 | 0.75 ± 0.02 | 0.5 ± 0.03 | 0.75 ± 0.02 | 0.75 ± 0.02 | 0.75 ± 0.02 | 0.75 ± 0.02 | | 0.75 ± 0.02 | 0.5 ± 0.03 | 0.75 ± 0.02 | 0.75 ± 0.02 | 0.75 ± 0.02 | 0.75 ± 0.02 | |
|  |  | LDA | 0.62 ± 0.02 | 0.63 ± 0.02 | 0.27 ± 0.03 | 0.63 ± 0.02 | 0.63 ± 0.02 | 0.63 ± 0.02 | 0.63 ± 0.02 | | 0.63 ± 0.02 | 0.27 ± 0.03 | 0.63 ± 0.02 | 0.63 ± 0.02 | 0.63 ± 0.02 | 0.64 ± 0.02 | |
|  |  | LR | 0.63 ± 0.02 | 0.63 ± 0.02 | 0.27 ± 0.04 | 0.64 ± 0.02 | 0.64 ± 0.02 | 0.64 ± 0.02 | 0.63 ± 0.02 | | 0.63 ± 0.02 | 0.27 ± 0.03 | 0.64 ± 0.02 | 0.64 ± 0.02 | 0.64 ± 0.02 | 0.63 ± 0.02 | |

|  |  |  |  | **Validation set** | | | | | | **Test set** | | | | | |
| --- | --- | --- | --- | --- | --- | --- | --- | --- | --- | --- | --- | --- | --- | --- | --- |
| **Activity threshold** | **PCC** | **Algorithm** | **F1 train** | **F1** | **MCC** | **ROC AUC** | **Accuracy** | **Precision** | **Recall** | **F1** | **MCC** | **ROC AUC** | **Accuracy** | **Precision** | **Recall** |
| GAP 5 | 85 | NB | 0.64 ± 0.0 | 0.61 ± 0.02 | 0.17 ± 0.03 | 0.59 ± 0.02 | 0.58 ± 0.02 | 0.57 ± 0.02 | 0.65 ± 0.03 | 0.61 ± 0.01 | 0.17 ± 0.03 | 0.59 ± 0.02 | 0.59 ± 0.02 | 0.57 ± 0.02 | 0.66 ± 0.02 |
|  |  | RF | 0.75 ± 0.01 | 0.76 ± 0.02 | 0.52 ± 0.03 | 0.76 ± 0.02 | 0.76 ± 0.02 | 0.76 ± 0.02 | 0.75 ± 0.01 | 0.76 ± 0.02 | 0.52 ± 0.03 | 0.76 ± 0.02 | 0.76 ± 0.02 | 0.76 ± 0.02 | 0.75 ± 0.02 |
|  |  | SVM | 0.74 ± 0.01 | 0.75 ± 0.01 | 0.5 ± 0.03 | 0.75 ± 0.02 | 0.75 ± 0.02 | 0.75 ± 0.02 | 0.75 ± 0.02 | 0.75 ± 0.01 | 0.5 ± 0.03 | 0.75 ± 0.02 | 0.75 ± 0.02 | 0.75 ± 0.02 | 0.75 ± 0.02 |
|  | 95 | AB | 0.69 ± 0.01 | 0.7 ± 0.02 | 0.39 ± 0.02 | 0.69 ± 0.01 | 0.69 ± 0.01 | 0.69 ± 0.02 | 0.7 ± 0.04 | 0.7 ± 0.02 | 0.39 ± 0.02 | 0.7 ± 0.01 | 0.7 ± 0.01 | 0.69 ± 0.02 | 0.7 ± 0.04 |
|  |  | CART | 0.67 ± 0.01 | 0.65 ± 0.02 | 0.3 ± 0.03 | 0.65 ± 0.02 | 0.65 ± 0.02 | 0.64 ± 0.02 | 0.66 ± 0.03 | 0.65 ± 0.02 | 0.3 ± 0.03 | 0.65 ± 0.02 | 0.65 ± 0.02 | 0.64 ± 0.02 | 0.66 ± 0.03 |
|  |  | ET | 0.69 ± 0.01 | 0.71 ± 0.02 | 0.41 ± 0.03 | 0.71 ± 0.02 | 0.71 ± 0.02 | 0.7 ± 0.02 | 0.71 ± 0.02 | 0.71 ± 0.02 | 0.41 ± 0.03 | 0.71 ± 0.02 | 0.71 ± 0.02 | 0.7 ± 0.02 | 0.71 ± 0.02 |
|  |  | GBM | 0.67 ± 0.01 | 0.68 ± 0.02 | 0.36 ± 0.03 | 0.68 ± 0.02 | 0.68 ± 0.02 | 0.68 ± 0.02 | 0.68 ± 0.02 | 0.68 ± 0.02 | 0.36 ± 0.03 | 0.68 ± 0.02 | 0.68 ± 0.02 | 0.68 ± 0.02 | 0.68 ± 0.03 |
|  |  | KNN | 0.75 ± 0.01 | 0.75 ± 0.02 | 0.5 ± 0.03 | 0.75 ± 0.02 | 0.75 ± 0.02 | 0.75 ± 0.02 | 0.75 ± 0.02 | 0.75 ± 0.02 | 0.5 ± 0.03 | 0.75 ± 0.02 | 0.75 ± 0.02 | 0.75 ± 0.02 | 0.75 ± 0.02 |
|  |  | LDA | 0.64 ± 0.01 | 0.65 ± 0.01 | 0.29 ± 0.03 | 0.64 ± 0.01 | 0.64 ± 0.01 | 0.64 ± 0.02 | 0.65 ± 0.01 | 0.64 ± 0.01 | 0.29 ± 0.03 | 0.64 ± 0.01 | 0.64 ± 0.01 | 0.64 ± 0.02 | 0.65 ± 0.02 |
|  |  | LR | 0.64 ± 0.02 | 0.64 ± 0.01 | 0.29 ± 0.03 | 0.64 ± 0.02 | 0.64 ± 0.02 | 0.64 ± 0.02 | 0.65 ± 0.01 | 0.64 ± 0.01 | 0.29 ± 0.03 | 0.64 ± 0.01 | 0.64 ± 0.01 | 0.64 ± 0.02 | 0.65 ± 0.02 |
|  |  | NB | 0.62 ± 0.01 | 0.6 ± 0.02 | 0.16 ± 0.03 | 0.58 ± 0.01 | 0.58 ± 0.01 | 0.57 ± 0.01 | 0.64 ± 0.03 | 0.6 ± 0.02 | 0.16 ± 0.03 | 0.58 ± 0.01 | 0.58 ± 0.01 | 0.57 ± 0.01 | 0.64 ± 0.04 |
|  |  | RF | 0.75 ± 0.01 | 0.76 ± 0.02 | 0.52 ± 0.03 | 0.76 ± 0.02 | 0.76 ± 0.02 | 0.76 ± 0.02 | 0.75 ± 0.02 | 0.76 ± 0.02 | 0.52 ± 0.03 | 0.76 ± 0.02 | 0.76 ± 0.02 | 0.76 ± 0.02 | 0.75 ± 0.02 |
|  |  | SVM | 0.74 ± 0.01 | 0.75 ± 0.01 | 0.49 ± 0.03 | 0.74 ± 0.02 | 0.74 ± 0.02 | 0.74 ± 0.02 | 0.75 ± 0.02 | 0.75 ± 0.02 | 0.49 ± 0.03 | 0.74 ± 0.02 | 0.74 ± 0.02 | 0.74 ± 0.03 | 0.75 ± 0.03 |
|  | 100 | AB | 0.69 ± 0.01 | 0.7 ± 0.01 | 0.39 ± 0.02 | 0.69 ± 0.01 | 0.69 ± 0.01 | 0.69 ± 0.02 | 0.7 ± 0.03 | 0.69 ± 0.01 | 0.39 ± 0.02 | 0.69 ± 0.01 | 0.69 ± 0.01 | 0.69 ± 0.02 | 0.7 ± 0.03 |
|  |  | CART | 0.67 ± 0.01 | 0.66 ± 0.01 | 0.24 ± 0.1 | 0.61 ± 0.06 | 0.61 ± 0.06 | 0.6 ± 0.07 | 0.77 ± 0.15 | 0.66 ± 0.01 | 0.23 ± 0.1 | 0.61 ± 0.06 | 0.61 ± 0.06 | 0.6 ± 0.07 | 0.77 ± 0.15 |
|  |  | ET | 0.69 ± 0.01 | 0.7 ± 0.01 | 0.39 ± 0.02 | 0.7 ± 0.01 | 0.7 ± 0.01 | 0.69 ± 0.02 | 0.71 ± 0.01 | 0.7 ± 0.01 | 0.39 ± 0.02 | 0.7 ± 0.01 | 0.7 ± 0.01 | 0.69 ± 0.01 | 0.71 ± 0.02 |
|  |  | GBM | 0.67 ± 0.01 | 0.68 ± 0.02 | 0.36 ± 0.04 | 0.68 ± 0.02 | 0.68 ± 0.02 | 0.68 ± 0.02 | 0.68 ± 0.02 | 0.68 ± 0.02 | 0.36 ± 0.04 | 0.68 ± 0.02 | 0.68 ± 0.02 | 0.68 ± 0.02 | 0.68 ± 0.03 |
|  |  | KNN | 0.74 ± 0.01 | 0.74 ± 0.01 | 0.49 ± 0.02 | 0.74 ± 0.01 | 0.74 ± 0.01 | 0.74 ± 0.02 | 0.75 ± 0.02 | 0.74 ± 0.01 | 0.49 ± 0.02 | 0.74 ± 0.01 | 0.74 ± 0.01 | 0.74 ± 0.02 | 0.75 ± 0.02 |
|  |  | LDA | 0.64 ± 0.01 | 0.66 ± 0.02 | 0.31 ± 0.03 | 0.66 ± 0.02 | 0.66 ± 0.02 | 0.66 ± 0.02 | 0.66 ± 0.02 | 0.66 ± 0.01 | 0.31 ± 0.03 | 0.66 ± 0.02 | 0.66 ± 0.02 | 0.66 ± 0.02 | 0.66 ± 0.02 |
|  |  | LR | 0.65 ± 0.02 | 0.65 ± 0.01 | 0.3 ± 0.03 | 0.65 ± 0.01 | 0.65 ± 0.01 | 0.65 ± 0.02 | 0.65 ± 0.01 | 0.65 ± 0.01 | 0.3 ± 0.03 | 0.65 ± 0.02 | 0.65 ± 0.02 | 0.65 ± 0.02 | 0.65 ± 0.02 |
|  |  | NB | 0.62 ± 0.02 | 0.6 ± 0.02 | 0.15 ± 0.04 | 0.57 ± 0.02 | 0.57 ± 0.02 | 0.56 ± 0.02 | 0.65 ± 0.04 | 0.6 ± 0.02 | 0.14 ± 0.04 | 0.57 ± 0.02 | 0.57 ± 0.02 | 0.56 ± 0.02 | 0.65 ± 0.04 |
|  |  | RF | 0.74 ± 0.01 | 0.75 ± 0.01 | 0.5 ± 0.03 | 0.75 ± 0.01 | 0.75 ± 0.01 | 0.75 ± 0.01 | 0.74 ± 0.01 | 0.75 ± 0.01 | 0.5 ± 0.03 | 0.75 ± 0.01 | 0.75 ± 0.01 | 0.75 ± 0.01 | 0.74 ± 0.02 |

|  |  |  |  | **Validation set** | | | | | | **Test set** | | | | | |  |
| --- | --- | --- | --- | --- | --- | --- | --- | --- | --- | --- | --- | --- | --- | --- | --- | --- |
| **Activity threshold** | **PCC** | **Algorithm** | **F1 train** | **F1** | **MCC** | **ROC AUC** | **Accuracy** | **Precision** | **Recall** | **F1** | **MCC** | **ROC AUC** | **Accuracy** | **Precision** | **Recall** | |
| GAP 5 | 100 | SVM | 0.74 ± 0.01 | 0.74 ± 0.02 | 0.47 ± 0.03 | 0.74 ± 0.02 | 0.74 ± 0.02 | 0.72 ± 0.02 | 0.77 ± 0.02 | 0.74 ± 0.02 | 0.47 ± 0.03 | 0.74 ± 0.02 | 0.74 ± 0.02 | 0.72 ± 0.02 | 0.77 ± 0.02 | |
|  |  |  |  |  |  |  |  |  |  |  |  |  |  |  |  | |
| GAP 10 | 75 | AB | 0.69 ± 0.01 | 0.7 ± 0.01 | 0.38 ± 0.02 | 0.69 ± 0.01 | 0.69 ± 0.01 | 0.68 ± 0.01 | 0.71 ± 0.02 | 0.7 ± 0.01 | 0.38 ± 0.02 | 0.69 ± 0.01 | 0.69 ± 0.01 | 0.68 ± 0.02 | 0.71 ± 0.03 | |
|  |  | CART | 0.68 ± 0.01 | 0.67 ± 0.02 | 0.33 ± 0.03 | 0.67 ± 0.02 | 0.67 ± 0.02 | 0.66 ± 0.02 | 0.67 ± 0.03 | 0.67 ± 0.02 | 0.34 ± 0.03 | 0.67 ± 0.01 | 0.67 ± 0.01 | 0.67 ± 0.02 | 0.67 ± 0.03 | |
|  |  | ET | 0.7 ± 0.01 | 0.7 ± 0.02 | 0.41 ± 0.03 | 0.7 ± 0.02 | 0.7 ± 0.02 | 0.7 ± 0.02 | 0.71 ± 0.01 | 0.7 ± 0.02 | 0.41 ± 0.03 | 0.7 ± 0.02 | 0.7 ± 0.02 | 0.7 ± 0.02 | 0.71 ± 0.02 | |
|  |  | GBM | 0.69 ± 0.01 | 0.68 ± 0.01 | 0.36 ± 0.02 | 0.68 ± 0.01 | 0.68 ± 0.01 | 0.68 ± 0.01 | 0.68 ± 0.01 | 0.68 ± 0.01 | 0.35 ± 0.02 | 0.68 ± 0.01 | 0.68 ± 0.01 | 0.68 ± 0.01 | 0.68 ± 0.02 | |
|  |  | KNN | 0.76 ± 0.01 | 0.77 ± 0.02 | 0.53 ± 0.02 | 0.76 ± 0.01 | 0.76 ± 0.01 | 0.76 ± 0.01 | 0.77 ± 0.03 | 0.77 ± 0.02 | 0.53 ± 0.02 | 0.76 ± 0.01 | 0.76 ± 0.01 | 0.76 ± 0.01 | 0.77 ± 0.03 | |
|  |  | LDA | 0.63 ± 0.01 | 0.63 ± 0.01 | 0.26 ± 0.02 | 0.63 ± 0.01 | 0.63 ± 0.01 | 0.63 ± 0.01 | 0.63 ± 0.01 | 0.63 ± 0.01 | 0.26 ± 0.02 | 0.63 ± 0.01 | 0.63 ± 0.01 | 0.63 ± 0.01 | 0.63 ± 0.02 | |
|  |  | LR | 0.64 ± 0.01 | 0.63 ± 0.01 | 0.26 ± 0.02 | 0.63 ± 0.01 | 0.63 ± 0.01 | 0.63 ± 0.01 | 0.63 ± 0.02 | 0.63 ± 0.01 | 0.26 ± 0.02 | 0.63 ± 0.01 | 0.63 ± 0.01 | 0.63 ± 0.01 | 0.63 ± 0.02 | |
|  |  | NB | 0.65 ± 0.03 | 0.63 ± 0.03 | 0.18 ± 0.04 | 0.59 ± 0.03 | 0.59 ± 0.03 | 0.57 ± 0.03 | 0.7 ± 0.11 | 0.63 ± 0.03 | 0.18 ± 0.04 | 0.59 ± 0.03 | 0.59 ± 0.03 | 0.57 ± 0.03 | 0.7 ± 0.11 | |
|  |  | RF | 0.76 ± 0.0 | 0.76 ± 0.01 | 0.54 ± 0.02 | 0.77 ± 0.01 | 0.77 ± 0.01 | 0.77 ± 0.01 | 0.76 ± 0.01 | 0.77 ± 0.01 | 0.54 ± 0.02 | 0.77 ± 0.01 | 0.77 ± 0.01 | 0.77 ± 0.01 | 0.76 ± 0.02 | |
|  |  | SVM | 0.75 ± 0.01 | 0.77 ± 0.02 | 0.53 ± 0.03 | 0.77 ± 0.02 | 0.77 ± 0.02 | 0.77 ± 0.02 | 0.77 ± 0.02 | 0.77 ± 0.01 | 0.53 ± 0.03 | 0.77 ± 0.01 | 0.77 ± 0.01 | 0.77 ± 0.02 | 0.77 ± 0.02 | |
|  | 85 | AB | 0.7 ± 0.01 | 0.72 ± 0.01 | 0.42 ± 0.02 | 0.71 ± 0.01 | 0.71 ± 0.01 | 0.7 ± 0.01 | 0.72 ± 0.02 | 0.72 ± 0.01 | 0.42 ± 0.02 | 0.71 ± 0.01 | 0.71 ± 0.01 | 0.7 ± 0.01 | 0.73 ± 0.03 | |
|  |  | CART | 0.68 ± 0.01 | 0.66 ± 0.01 | 0.32 ± 0.03 | 0.66 ± 0.02 | 0.66 ± 0.02 | 0.66 ± 0.02 | 0.66 ± 0.02 | 0.66 ± 0.01 | 0.32 ± 0.03 | 0.66 ± 0.02 | 0.66 ± 0.02 | 0.66 ± 0.02 | 0.66 ± 0.02 | |
|  |  | ET | 0.71 ± 0.01 | 0.71 ± 0.01 | 0.42 ± 0.03 | 0.71 ± 0.01 | 0.71 ± 0.01 | 0.71 ± 0.01 | 0.72 ± 0.01 | 0.72 ± 0.01 | 0.43 ± 0.02 | 0.71 ± 0.01 | 0.71 ± 0.01 | 0.71 ± 0.01 | 0.72 ± 0.02 | |
|  |  | GBM | 0.69 ± 0.01 | 0.69 ± 0.01 | 0.38 ± 0.02 | 0.69 ± 0.01 | 0.69 ± 0.01 | 0.68 ± 0.01 | 0.7 ± 0.02 | 0.69 ± 0.01 | 0.38 ± 0.02 | 0.69 ± 0.01 | 0.69 ± 0.01 | 0.69 ± 0.01 | 0.7 ± 0.03 | |
|  |  | KNN | 0.77 ± 0.01 | 0.77 ± 0.01 | 0.53 ± 0.02 | 0.76 ± 0.01 | 0.76 ± 0.01 | 0.76 ± 0.01 | 0.77 ± 0.03 | 0.77 ± 0.01 | 0.53 ± 0.02 | 0.76 ± 0.01 | 0.76 ± 0.01 | 0.76 ± 0.01 | 0.77 ± 0.03 | |
|  |  | LDA | 0.64 ± 0.01 | 0.64 ± 0.01 | 0.27 ± 0.02 | 0.64 ± 0.01 | 0.64 ± 0.01 | 0.64 ± 0.01 | 0.64 ± 0.02 | 0.64 ± 0.01 | 0.27 ± 0.02 | 0.64 ± 0.01 | 0.64 ± 0.01 | 0.64 ± 0.01 | 0.64 ± 0.02 | |
|  |  | LR | 0.64 ± 0.01 | 0.63 ± 0.0 | 0.27 ± 0.01 | 0.64 ± 0.01 | 0.64 ± 0.01 | 0.64 ± 0.01 | 0.63 ± 0.01 | 0.63 ± 0.01 | 0.27 ± 0.02 | 0.64 ± 0.01 | 0.64 ± 0.01 | 0.64 ± 0.01 | 0.63 ± 0.02 | |
|  |  | NB | 0.64 ± 0.02 | 0.62 ± 0.03 | 0.18 ± 0.03 | 0.59 ± 0.02 | 0.59 ± 0.02 | 0.57 ± 0.02 | 0.69 ± 0.09 | 0.62 ± 0.03 | 0.18 ± 0.03 | 0.59 ± 0.02 | 0.59 ± 0.02 | 0.57 ± 0.02 | 0.69 ± 0.09 | |
|  |  | RF | 0.77 ± 0.01 | 0.77 ± 0.01 | 0.54 ± 0.02 | 0.77 ± 0.01 | 0.77 ± 0.01 | 0.77 ± 0.01 | 0.76 ± 0.02 | 0.77 ± 0.02 | 0.54 ± 0.02 | 0.77 ± 0.01 | 0.77 ± 0.01 | 0.77 ± 0.02 | 0.76 ± 0.03 | |

|  |  |  |  | **Validation set** | | | | | | **Test set** | | | | | | |
| --- | --- | --- | --- | --- | --- | --- | --- | --- | --- | --- | --- | --- | --- | --- | --- | --- |
| **Activity threshold** | **PCC** | **Algorithm** | **F1 train** | **F1** | **MCC** | **ROC AUC** | **Accuracy** | **Precision** | **Recall** | **F1** | **MCC** | **ROC AUC** | **Accuracy** | **Precision** | **Recall** |  |
| GAP 10 | 85 | SVM | 0.76 ± 0.01 | 0.77 ± 0.01 | 0.53 ± 0.02 | 0.77 ± 0.01 | 0.77 ± 0.01 | 0.77 ± 0.02 | 0.76 ± 0.02 | 0.77 ± 0.01 | 0.54 ± 0.02 | 0.77 ± 0.01 | 0.77 ± 0.01 | 0.77 ± 0.02 | 0.77 ± 0.02 |  |
|  | 95 | AB | 0.7 ± 0.01 | 0.71 ± 0.01 | 0.42 ± 0.02 | 0.71 ± 0.01 | 0.71 ± 0.01 | 0.7 ± 0.01 | 0.73 ± 0.02 | 0.72 ± 0.01 | 0.42 ± 0.02 | 0.71 ± 0.01 | 0.71 ± 0.01 | 0.7 ± 0.01 | 0.73 ± 0.03 |  |
|  |  | CART | 0.68 ± 0.01 | 0.67 ± 0.02 | 0.33 ± 0.02 | 0.67 ± 0.01 | 0.67 ± 0.01 | 0.66 ± 0.01 | 0.67 ± 0.04 | 0.67 ± 0.02 | 0.33 ± 0.02 | 0.66 ± 0.01 | 0.66 ± 0.01 | 0.66 ± 0.02 | 0.67 ± 0.04 |  |
|  |  | ET | 0.71 ± 0.01 | 0.71 ± 0.02 | 0.42 ± 0.03 | 0.71 ± 0.02 | 0.71 ± 0.02 | 0.71 ± 0.02 | 0.72 ± 0.02 | 0.72 ± 0.02 | 0.43 ± 0.03 | 0.71 ± 0.02 | 0.71 ± 0.02 | 0.71 ± 0.02 | 0.72 ± 0.02 |  |
|  |  | GBM | 0.69 ± 0.01 | 0.69 ± 0.02 | 0.38 ± 0.04 | 0.69 ± 0.02 | 0.69 ± 0.02 | 0.69 ± 0.02 | 0.7 ± 0.02 | 0.69 ± 0.02 | 0.38 ± 0.03 | 0.69 ± 0.02 | 0.69 ± 0.02 | 0.69 ± 0.02 | 0.7 ± 0.04 |  |
|  |  | KNN | 0.76 ± 0.01 | 0.77 ± 0.02 | 0.54 ± 0.02 | 0.77 ± 0.01 | 0.77 ± 0.01 | 0.77 ± 0.01 | 0.78 ± 0.03 | 0.77 ± 0.02 | 0.54 ± 0.02 | 0.77 ± 0.01 | 0.77 ± 0.01 | 0.77 ± 0.01 | 0.78 ± 0.03 |  |
|  |  | LDA | 0.65 ± 0.01 | 0.65 ± 0.01 | 0.3 ± 0.02 | 0.65 ± 0.01 | 0.65 ± 0.01 | 0.65 ± 0.01 | 0.66 ± 0.01 | 0.65 ± 0.01 | 0.3 ± 0.02 | 0.65 ± 0.01 | 0.65 ± 0.01 | 0.65 ± 0.01 | 0.66 ± 0.02 |  |
|  |  | LR | 0.66 ± 0.01 | 0.66 ± 0.01 | 0.31 ± 0.02 | 0.65 ± 0.01 | 0.65 ± 0.01 | 0.65 ± 0.01 | 0.66 ± 0.02 | 0.65 ± 0.01 | 0.31 ± 0.02 | 0.65 ± 0.01 | 0.65 ± 0.01 | 0.65 ± 0.01 | 0.66 ± 0.02 |  |
|  |  | NB | 0.63 ± 0.03 | 0.61 ± 0.03 | 0.17 ± 0.03 | 0.58 ± 0.01 | 0.58 ± 0.01 | 0.57 ± 0.01 | 0.67 ± 0.08 | 0.61 ± 0.03 | 0.17 ± 0.03 | 0.58 ± 0.01 | 0.58 ± 0.01 | 0.57 ± 0.01 | 0.67 ± 0.08 |  |
|  |  | RF | 0.77 ± 0.01 | 0.77 ± 0.01 | 0.54 ± 0.02 | 0.77 ± 0.01 | 0.77 ± 0.01 | 0.78 ± 0.01 | 0.76 ± 0.02 | 0.77 ± 0.01 | 0.55 ± 0.02 | 0.77 ± 0.01 | 0.77 ± 0.01 | 0.78 ± 0.02 | 0.77 ± 0.03 |  |
|  |  | SVM | 0.75 ± 0.01 | 0.76 ± 0.01 | 0.52 ± 0.02 | 0.76 ± 0.01 | 0.76 ± 0.01 | 0.76 ± 0.02 | 0.76 ± 0.02 | 0.76 ± 0.01 | 0.53 ± 0.02 | 0.76 ± 0.01 | 0.76 ± 0.01 | 0.76 ± 0.02 | 0.76 ± 0.02 |  |
|  | 100 | AB | 0.7 ± 0.01 | 0.72 ± 0.01 | 0.42 ± 0.02 | 0.71 ± 0.01 | 0.71 ± 0.01 | 0.7 ± 0.01 | 0.73 ± 0.02 | 0.72 ± 0.01 | 0.42 ± 0.02 | 0.71 ± 0.01 | 0.71 ± 0.01 | 0.7 ± 0.01 | 0.73 ± 0.03 |  |
|  |  | CART | 0.68 ± 0.01 | 0.66 ± 0.02 | 0.33 ± 0.03 | 0.66 ± 0.02 | 0.66 ± 0.02 | 0.66 ± 0.02 | 0.66 ± 0.02 | 0.66 ± 0.02 | 0.33 ± 0.03 | 0.66 ± 0.02 | 0.66 ± 0.02 | 0.66 ± 0.02 | 0.67 ± 0.02 |  |
|  |  | ET | 0.7 ± 0.0 | 0.7 ± 0.02 | 0.4 ± 0.03 | 0.7 ± 0.02 | 0.7 ± 0.02 | 0.7 ± 0.02 | 0.72 ± 0.01 | 0.71 ± 0.02 | 0.4 ± 0.03 | 0.7 ± 0.02 | 0.7 ± 0.02 | 0.7 ± 0.02 | 0.72 ± 0.03 |  |
|  |  | GBM | 0.69 ± 0.01 | 0.69 ± 0.02 | 0.37 ± 0.03 | 0.68 ± 0.02 | 0.68 ± 0.02 | 0.68 ± 0.02 | 0.69 ± 0.02 | 0.68 ± 0.02 | 0.37 ± 0.03 | 0.68 ± 0.02 | 0.68 ± 0.02 | 0.68 ± 0.02 | 0.69 ± 0.03 |  |
|  |  | KNN | 0.76 ± 0.01 | 0.76 ± 0.02 | 0.52 ± 0.03 | 0.76 ± 0.02 | 0.76 ± 0.02 | 0.76 ± 0.01 | 0.77 ± 0.03 | 0.76 ± 0.02 | 0.52 ± 0.03 | 0.76 ± 0.02 | 0.76 ± 0.02 | 0.76 ± 0.01 | 0.77 ± 0.03 |  |
|  |  | LDA | 0.67 ± 0.01 | 0.66 ± 0.01 | 0.32 ± 0.02 | 0.66 ± 0.01 | 0.66 ± 0.01 | 0.66 ± 0.01 | 0.66 ± 0.01 | 0.66 ± 0.01 | 0.32 ± 0.02 | 0.66 ± 0.01 | 0.66 ± 0.01 | 0.66 ± 0.01 | 0.66 ± 0.02 |  |
|  |  | LR | 0.67 ± 0.0 | 0.65 ± 0.01 | 0.31 ± 0.02 | 0.65 ± 0.01 | 0.65 ± 0.01 | 0.65 ± 0.01 | 0.65 ± 0.01 | 0.65 ± 0.01 | 0.31 ± 0.02 | 0.65 ± 0.01 | 0.65 ± 0.01 | 0.65 ± 0.01 | 0.65 ± 0.02 |  |
|  |  | NB | 0.63 ± 0.04 | 0.61 ± 0.04 | 0.15 ± 0.04 | 0.57 ± 0.02 | 0.57 ± 0.02 | 0.56 ± 0.02 | 0.68 ± 0.1 | 0.61 ± 0.04 | 0.15 ± 0.04 | 0.57 ± 0.02 | 0.57 ± 0.02 | 0.56 ± 0.02 | 0.68 ± 0.1 |  |
|  |  | RF | 0.76 ± 0.01 | 0.76 ± 0.01 | 0.52 ± 0.03 | 0.76 ± 0.01 | 0.76 ± 0.01 | 0.76 ± 0.01 | 0.75 ± 0.02 | 0.76 ± 0.02 | 0.52 ± 0.03 | 0.76 ± 0.01 | 0.76 ± 0.01 | 0.77 ± 0.02 | 0.76 ± 0.03 |  |
|  |  | SVM | 0.75 ± 0.01 | 0.76 ± 0.01 | 0.5 ± 0.02 | 0.75 ± 0.01 | 0.75 ± 0.01 | 0.74 ± 0.02 | 0.77 ± 0.02 | 0.75 ± 0.01 | 0.5 ± 0.02 | 0.75 ± 0.01 | 0.75 ± 0.01 | 0.74 ± 0.02 | 0.76 ± 0.03 |  |

|  |  |  |  | **Validation set** | | | | | | **Test set** | | | | | | |
| --- | --- | --- | --- | --- | --- | --- | --- | --- | --- | --- | --- | --- | --- | --- | --- | --- |
| **Activity threshold** | **PCC** | **Algorithm** | **F1 train** | **F1** | **MCC** | **ROC AUC** | **Accuracy** | **Precision** | **Recall** | **F1** | **MCC** | **ROC AUC** | **Accuracy** | **Precision** | **Recall** |  |
| GAP 15 | 75 | AB | 0.7 ± 0.01 | 0.71 ± 0.01 | 0.42 ± 0.02 | 0.71 ± 0.01 | 0.71 ± 0.01 | 0.7 ± 0.02 | 0.72 ± 0.02 | 0.71 ± 0.02 | 0.41 ± 0.03 | 0.7 ± 0.01 | 0.7 ± 0.01 | 0.7 ± 0.02 | 0.72 ± 0.03 |  |
|  |  | CART | 0.69 ± 0.01 | 0.68 ± 0.01 | 0.35 ± 0.03 | 0.68 ± 0.01 | 0.68 ± 0.01 | 0.67 ± 0.02 | 0.68 ± 0.02 | 0.68 ± 0.01 | 0.36 ± 0.03 | 0.68 ± 0.01 | 0.68 ± 0.01 | 0.68 ± 0.02 | 0.69 ± 0.03 |  |
|  |  | ET | 0.7 ± 0.01 | 0.71 ± 0.01 | 0.42 ± 0.02 | 0.71 ± 0.01 | 0.71 ± 0.01 | 0.71 ± 0.01 | 0.71 ± 0.02 | 0.71 ± 0.01 | 0.43 ± 0.02 | 0.71 ± 0.01 | 0.71 ± 0.01 | 0.71 ± 0.02 | 0.71 ± 0.03 |  |
|  |  | GBM | 0.69 ± 0.0 | 0.69 ± 0.01 | 0.38 ± 0.02 | 0.69 ± 0.01 | 0.69 ± 0.01 | 0.69 ± 0.01 | 0.69 ± 0.01 | 0.69 ± 0.01 | 0.38 ± 0.02 | 0.69 ± 0.01 | 0.69 ± 0.01 | 0.69 ± 0.01 | 0.69 ± 0.03 |  |
|  |  | KNN | 0.78 ± 0.0 | 0.78 ± 0.01 | 0.56 ± 0.02 | 0.78 ± 0.01 | 0.78 ± 0.01 | 0.78 ± 0.01 | 0.78 ± 0.02 | 0.78 ± 0.01 | 0.56 ± 0.02 | 0.78 ± 0.01 | 0.78 ± 0.01 | 0.78 ± 0.01 | 0.78 ± 0.02 |  |
|  |  | LDA | 0.63 ± 0.01 | 0.63 ± 0.02 | 0.27 ± 0.03 | 0.63 ± 0.02 | 0.63 ± 0.02 | 0.64 ± 0.01 | 0.63 ± 0.02 | 0.63 ± 0.02 | 0.27 ± 0.03 | 0.63 ± 0.02 | 0.63 ± 0.02 | 0.64 ± 0.01 | 0.63 ± 0.03 |  |
|  |  | LR | 0.64 ± 0.01 | 0.63 ± 0.02 | 0.27 ± 0.02 | 0.63 ± 0.01 | 0.63 ± 0.01 | 0.64 ± 0.01 | 0.62 ± 0.02 | 0.63 ± 0.02 | 0.27 ± 0.03 | 0.64 ± 0.01 | 0.64 ± 0.01 | 0.64 ± 0.01 | 0.63 ± 0.03 |  |
|  |  | NB | 0.66 ± 0.01 | 0.64 ± 0.03 | 0.19 ± 0.05 | 0.59 ± 0.03 | 0.59 ± 0.03 | 0.57 ± 0.03 | 0.73 ± 0.1 | 0.64 ± 0.03 | 0.19 ± 0.04 | 0.59 ± 0.03 | 0.59 ± 0.03 | 0.57 ± 0.03 | 0.73 ± 0.11 |  |
|  |  | RF | 0.78 ± 0.0 | 0.78 ± 0.01 | 0.56 ± 0.02 | 0.78 ± 0.01 | 0.78 ± 0.01 | 0.79 ± 0.01 | 0.77 ± 0.01 | 0.78 ± 0.01 | 0.57 ± 0.02 | 0.78 ± 0.01 | 0.78 ± 0.01 | 0.79 ± 0.01 | 0.77 ± 0.03 |  |
|  |  | SVM | 0.77 ± 0.0 | 0.78 ± 0.01 | 0.57 ± 0.01 | 0.78 ± 0.01 | 0.78 ± 0.01 | 0.79 ± 0.01 | 0.77 ± 0.01 | 0.78 ± 0.01 | 0.57 ± 0.02 | 0.78 ± 0.01 | 0.78 ± 0.01 | 0.79 ± 0.01 | 0.77 ± 0.02 |  |
|  | 85 | AB | 0.71 ± 0.0 | 0.73 ± 0.01 | 0.45 ± 0.02 | 0.73 ± 0.01 | 0.73 ± 0.01 | 0.71 ± 0.01 | 0.76 ± 0.02 | 0.73 ± 0.01 | 0.45 ± 0.02 | 0.72 ± 0.01 | 0.72 ± 0.01 | 0.71 ± 0.02 | 0.76 ± 0.03 |  |
|  |  | CART | 0.69 ± 0.0 | 0.68 ± 0.02 | 0.36 ± 0.03 | 0.68 ± 0.02 | 0.68 ± 0.02 | 0.68 ± 0.02 | 0.67 ± 0.03 | 0.68 ± 0.02 | 0.36 ± 0.03 | 0.68 ± 0.01 | 0.68 ± 0.01 | 0.68 ± 0.02 | 0.67 ± 0.04 |  |
|  |  | ET | 0.71 ± 0.0 | 0.72 ± 0.01 | 0.44 ± 0.03 | 0.72 ± 0.01 | 0.72 ± 0.01 | 0.72 ± 0.02 | 0.73 ± 0.02 | 0.72 ± 0.01 | 0.44 ± 0.03 | 0.72 ± 0.01 | 0.72 ± 0.01 | 0.72 ± 0.02 | 0.73 ± 0.02 |  |
|  |  | GBM | 0.7 ± 0.0 | 0.7 ± 0.01 | 0.39 ± 0.02 | 0.7 ± 0.01 | 0.7 ± 0.01 | 0.7 ± 0.01 | 0.7 ± 0.01 | 0.7 ± 0.01 | 0.4 ± 0.01 | 0.7 ± 0.01 | 0.7 ± 0.01 | 0.7 ± 0.01 | 0.7 ± 0.03 |  |
|  |  | KNN | 0.78 ± 0.0 | 0.78 ± 0.01 | 0.57 ± 0.01 | 0.78 ± 0.01 | 0.78 ± 0.01 | 0.79 ± 0.01 | 0.78 ± 0.01 | 0.78 ± 0.01 | 0.57 ± 0.01 | 0.78 ± 0.01 | 0.78 ± 0.01 | 0.79 ± 0.01 | 0.78 ± 0.01 |  |
|  |  | LDA | 0.64 ± 0.01 | 0.63 ± 0.01 | 0.28 ± 0.02 | 0.64 ± 0.01 | 0.64 ± 0.01 | 0.64 ± 0.01 | 0.63 ± 0.02 | 0.63 ± 0.01 | 0.28 ± 0.02 | 0.64 ± 0.01 | 0.64 ± 0.01 | 0.64 ± 0.01 | 0.63 ± 0.02 |  |
|  |  | LR | 0.64 ± 0.01 | 0.63 ± 0.01 | 0.28 ± 0.02 | 0.64 ± 0.01 | 0.64 ± 0.01 | 0.64 ± 0.01 | 0.63 ± 0.02 | 0.63 ± 0.01 | 0.28 ± 0.02 | 0.64 ± 0.01 | 0.64 ± 0.01 | 0.64 ± 0.01 | 0.63 ± 0.02 |  |
|  |  | NB | 0.65 ± 0.01 | 0.63 ± 0.03 | 0.18 ± 0.06 | 0.58 ± 0.03 | 0.58 ± 0.03 | 0.57 ± 0.03 | 0.72 ± 0.1 | 0.63 ± 0.03 | 0.17 ± 0.05 | 0.58 ± 0.03 | 0.58 ± 0.03 | 0.57 ± 0.03 | 0.72 ± 0.1 |  |
|  |  | RF | 0.78 ± 0.01 | 0.78 ± 0.01 | 0.57 ± 0.02 | 0.79 ± 0.01 | 0.79 ± 0.01 | 0.79 ± 0.01 | 0.78 ± 0.01 | 0.78 ± 0.01 | 0.57 ± 0.01 | 0.79 ± 0.01 | 0.79 ± 0.01 | 0.79 ± 0.01 | 0.78 ± 0.02 |  |
|  |  | SVM | 0.77 ± 0.0 | 0.78 ± 0.01 | 0.57 ± 0.02 | 0.78 ± 0.01 | 0.78 ± 0.01 | 0.79 ± 0.02 | 0.77 ± 0.02 | 0.78 ± 0.01 | 0.57 ± 0.02 | 0.78 ± 0.01 | 0.78 ± 0.01 | 0.79 ± 0.02 | 0.77 ± 0.02 |  |
|  | 95 | AB | 0.71 ± 0.0 | 0.73 ± 0.01 | 0.45 ± 0.02 | 0.72 ± 0.01 | 0.72 ± 0.01 | 0.71 ± 0.01 | 0.75 ± 0.02 | 0.73 ± 0.01 | 0.45 ± 0.03 | 0.72 ± 0.01 | 0.72 ± 0.01 | 0.72 ± 0.02 | 0.75 ± 0.03 |  |

|  |  |  |  | **Validation set** | | | | | | **Test set** | | | | | |  |
| --- | --- | --- | --- | --- | --- | --- | --- | --- | --- | --- | --- | --- | --- | --- | --- | --- |
| **Activity threshold** | **PCC** | **Algorithm** | **F1 train** | **F1** | **MCC** | **ROC AUC** | **Accuracy** | **Precision** | **Recall** | **F1** | **MCC** | **ROC AUC** | **Accuracy** | **Precision** | **Recall** | |
| GAP 15 | 95 | CART | 0.69 ± 0.0 | 0.68 ± 0.01 | 0.36 ± 0.03 | 0.68 ± 0.02 | 0.68 ± 0.02 | 0.67 ± 0.02 | 0.69 ± 0.02 | 0.68 ± 0.02 | 0.35 ± 0.04 | 0.68 ± 0.02 | 0.68 ± 0.02 | 0.67 ± 0.02 | 0.69 ± 0.03 | |
|  |  | ET | 0.71 ± 0.0 | 0.72 ± 0.01 | 0.45 ± 0.02 | 0.72 ± 0.01 | 0.72 ± 0.01 | 0.72 ± 0.01 | 0.73 ± 0.01 | 0.72 ± 0.01 | 0.45 ± 0.03 | 0.72 ± 0.02 | 0.72 ± 0.02 | 0.72 ± 0.02 | 0.73 ± 0.03 | |
|  |  | GBM | 0.7 ± 0.0 | 0.69 ± 0.01 | 0.38 ± 0.02 | 0.69 ± 0.01 | 0.69 ± 0.01 | 0.69 ± 0.01 | 0.7 ± 0.02 | 0.69 ± 0.01 | 0.38 ± 0.02 | 0.69 ± 0.01 | 0.69 ± 0.01 | 0.69 ± 0.02 | 0.7 ± 0.03 | |
|  |  | KNN | 0.78 ± 0.0 | 0.78 ± 0.01 | 0.57 ± 0.02 | 0.78 ± 0.01 | 0.78 ± 0.01 | 0.78 ± 0.01 | 0.78 ± 0.01 | 0.78 ± 0.01 | 0.57 ± 0.02 | 0.78 ± 0.01 | 0.78 ± 0.01 | 0.78 ± 0.01 | 0.78 ± 0.01 | |
|  |  | LDA | 0.65 ± 0.01 | 0.65 ± 0.01 | 0.3 ± 0.02 | 0.65 ± 0.01 | 0.65 ± 0.01 | 0.65 ± 0.01 | 0.65 ± 0.01 | 0.65 ± 0.02 | 0.3 ± 0.02 | 0.65 ± 0.01 | 0.65 ± 0.01 | 0.65 ± 0.01 | 0.65 ± 0.03 | |
|  |  | LR | 0.66 ± 0.0 | 0.65 ± 0.01 | 0.3 ± 0.02 | 0.65 ± 0.01 | 0.65 ± 0.01 | 0.65 ± 0.01 | 0.65 ± 0.02 | 0.65 ± 0.01 | 0.3 ± 0.02 | 0.65 ± 0.01 | 0.65 ± 0.01 | 0.65 ± 0.01 | 0.65 ± 0.02 | |
|  |  | NB | 0.64 ± 0.02 | 0.62 ± 0.03 | 0.16 ± 0.05 | 0.58 ± 0.03 | 0.58 ± 0.03 | 0.57 ± 0.02 | 0.69 ± 0.09 | 0.62 ± 0.03 | 0.16 ± 0.05 | 0.58 ± 0.03 | 0.58 ± 0.03 | 0.57 ± 0.02 | 0.69 ± 0.1 | |
|  |  | RF | 0.78 ± 0.0 | 0.78 ± 0.01 | 0.57 ± 0.01 | 0.79 ± 0.01 | 0.79 ± 0.01 | 0.79 ± 0.01 | 0.78 ± 0.01 | 0.78 ± 0.01 | 0.57 ± 0.01 | 0.79 ± 0.01 | 0.79 ± 0.01 | 0.79 ± 0.01 | 0.78 ± 0.02 | |
|  |  | SVM | 0.77 ± 0.0 | 0.78 ± 0.01 | 0.57 ± 0.02 | 0.78 ± 0.01 | 0.78 ± 0.01 | 0.8 ± 0.01 | 0.76 ± 0.02 | 0.78 ± 0.01 | 0.56 ± 0.01 | 0.78 ± 0.01 | 0.78 ± 0.01 | 0.8 ± 0.02 | 0.76 ± 0.02 | |
|  | 100 | AB | 0.71 ± 0.01 | 0.73 ± 0.01 | 0.45 ± 0.02 | 0.72 ± 0.01 | 0.72 ± 0.01 | 0.71 ± 0.02 | 0.76 ± 0.02 | 0.73 ± 0.02 | 0.45 ± 0.03 | 0.72 ± 0.01 | 0.72 ± 0.01 | 0.71 ± 0.02 | 0.75 ± 0.04 | |
|  |  | CART | 0.68 ± 0.0 | 0.67 ± 0.03 | 0.34 ± 0.06 | 0.67 ± 0.03 | 0.67 ± 0.03 | 0.67 ± 0.03 | 0.66 ± 0.03 | 0.67 ± 0.03 | 0.34 ± 0.06 | 0.67 ± 0.03 | 0.67 ± 0.03 | 0.67 ± 0.03 | 0.66 ± 0.03 | |
|  |  | ET | 0.71 ± 0.01 | 0.71 ± 0.01 | 0.42 ± 0.03 | 0.71 ± 0.01 | 0.71 ± 0.01 | 0.7 ± 0.02 | 0.72 ± 0.02 | 0.71 ± 0.01 | 0.42 ± 0.03 | 0.71 ± 0.01 | 0.71 ± 0.01 | 0.7 ± 0.02 | 0.72 ± 0.03 | |
|  |  | GBM | 0.7 ± 0.01 | 0.69 ± 0.01 | 0.38 ± 0.03 | 0.69 ± 0.01 | 0.69 ± 0.01 | 0.69 ± 0.01 | 0.7 ± 0.02 | 0.69 ± 0.02 | 0.38 ± 0.02 | 0.69 ± 0.01 | 0.69 ± 0.01 | 0.69 ± 0.02 | 0.69 ± 0.04 | |
|  |  | KNN | 0.77 ± 0.01 | 0.77 ± 0.01 | 0.55 ± 0.02 | 0.77 ± 0.01 | 0.77 ± 0.01 | 0.77 ± 0.02 | 0.78 ± 0.02 | 0.77 ± 0.01 | 0.55 ± 0.02 | 0.77 ± 0.01 | 0.77 ± 0.01 | 0.77 ± 0.02 | 0.78 ± 0.02 | |
|  |  | LDA | 0.66 ± 0.01 | 0.66 ± 0.02 | 0.33 ± 0.03 | 0.66 ± 0.01 | 0.66 ± 0.01 | 0.66 ± 0.01 | 0.66 ± 0.02 | 0.66 ± 0.01 | 0.32 ± 0.02 | 0.66 ± 0.01 | 0.66 ± 0.01 | 0.66 ± 0.01 | 0.66 ± 0.02 | |
|  |  | LR | 0.66 ± 0.01 | 0.66 ± 0.02 | 0.31 ± 0.03 | 0.66 ± 0.02 | 0.66 ± 0.02 | 0.66 ± 0.02 | 0.65 ± 0.02 | 0.66 ± 0.02 | 0.31 ± 0.03 | 0.66 ± 0.02 | 0.66 ± 0.02 | 0.66 ± 0.02 | 0.65 ± 0.03 | |
|  |  | NB | 0.63 ± 0.02 | 0.62 ± 0.03 | 0.16 ± 0.04 | 0.58 ± 0.02 | 0.58 ± 0.02 | 0.57 ± 0.02 | 0.69 ± 0.09 | 0.62 ± 0.03 | 0.16 ± 0.04 | 0.58 ± 0.02 | 0.58 ± 0.02 | 0.57 ± 0.02 | 0.69 ± 0.09 | |
|  |  | RF | 0.77 ± 0.0 | 0.78 ± 0.01 | 0.57 ± 0.01 | 0.78 ± 0.01 | 0.78 ± 0.01 | 0.79 ± 0.01 | 0.77 ± 0.02 | 0.78 ± 0.01 | 0.57 ± 0.02 | 0.78 ± 0.01 | 0.78 ± 0.01 | 0.79 ± 0.01 | 0.78 ± 0.02 | |
|  |  | SVM | 0.77 ± 0.01 | 0.76 ± 0.01 | 0.52 ± 0.03 | 0.76 ± 0.01 | 0.76 ± 0.01 | 0.75 ± 0.02 | 0.78 ± 0.02 | 0.77 ± 0.01 | 0.52 ± 0.02 | 0.76 ± 0.01 | 0.76 ± 0.01 | 0.76 ± 0.03 | 0.78 ± 0.02 | |
|  |  |  |  |  |  |  |  |  |  |  |  |  |  |  |  | |
| GAP 20 | 75 | AB | 0.72 ± 0.01 | 0.73 ± 0.01 | 0.45 ± 0.02 | 0.72 ± 0.01 | 0.72 ± 0.01 | 0.71 ± 0.02 | 0.75 ± 0.03 | 0.73 ± 0.01 | 0.45 ± 0.03 | 0.72 ± 0.01 | 0.72 ± 0.01 | 0.71 ± 0.02 | 0.75 ± 0.03 | |

|  |  |  |  | **Validation set** | | | | | | **Test set** | | | | | |
| --- | --- | --- | --- | --- | --- | --- | --- | --- | --- | --- | --- | --- | --- | --- | --- |
| **Activity threshold** | **PCC** | **Algorithm** | **F1 train** | **F1** | **MCC** | **ROC AUC** | **Accuracy** | **Precision** | **Recall** | **F1** | **MCC** | **ROC AUC** | **Accuracy** | **Precision** | **Recall** |
| GAP 20 | 75 | CART | 0.7 ± 0.0 | 0.68 ± 0.02 | 0.36 ± 0.03 | 0.68 ± 0.01 | 0.68 ± 0.01 | 0.68 ± 0.01 | 0.69 ± 0.03 | 0.69 ± 0.02 | 0.37 ± 0.03 | 0.68 ± 0.01 | 0.68 ± 0.01 | 0.68 ± 0.01 | 0.7 ± 0.03 |
|  |  | ET | 0.71 ± 0.01 | 0.73 ± 0.02 | 0.46 ± 0.03 | 0.73 ± 0.02 | 0.73 ± 0.02 | 0.73 ± 0.02 | 0.73 ± 0.02 | 0.73 ± 0.02 | 0.46 ± 0.04 | 0.73 ± 0.02 | 0.73 ± 0.02 | 0.73 ± 0.02 | 0.72 ± 0.03 |
|  |  | GBM | 0.7 ± 0.01 | 0.7 ± 0.01 | 0.41 ± 0.02 | 0.7 ± 0.01 | 0.7 ± 0.01 | 0.7 ± 0.01 | 0.7 ± 0.02 | 0.7 ± 0.01 | 0.41 ± 0.02 | 0.7 ± 0.01 | 0.7 ± 0.01 | 0.7 ± 0.01 | 0.7 ± 0.02 |
|  |  | KNN | 0.78 ± 0.01 | 0.79 ± 0.01 | 0.58 ± 0.02 | 0.79 ± 0.01 | 0.79 ± 0.01 | 0.79 ± 0.01 | 0.79 ± 0.02 | 0.79 ± 0.01 | 0.58 ± 0.02 | 0.79 ± 0.01 | 0.79 ± 0.01 | 0.79 ± 0.01 | 0.79 ± 0.02 |
|  |  | LDA | 0.64 ± 0.01 | 0.64 ± 0.02 | 0.29 ± 0.03 | 0.65 ± 0.02 | 0.65 ± 0.02 | 0.65 ± 0.02 | 0.64 ± 0.02 | 0.64 ± 0.02 | 0.3 ± 0.03 | 0.65 ± 0.02 | 0.65 ± 0.02 | 0.65 ± 0.01 | 0.64 ± 0.04 |
|  |  | LR | 0.65 ± 0.01 | 0.64 ± 0.01 | 0.29 ± 0.03 | 0.64 ± 0.01 | 0.64 ± 0.01 | 0.65 ± 0.02 | 0.64 ± 0.02 | 0.64 ± 0.02 | 0.29 ± 0.03 | 0.64 ± 0.02 | 0.64 ± 0.02 | 0.65 ± 0.02 | 0.64 ± 0.04 |
|  |  | NB | 0.67 ± 0.01 | 0.65 ± 0.02 | 0.2 ± 0.02 | 0.59 ± 0.02 | 0.59 ± 0.02 | 0.57 ± 0.02 | 0.77 ± 0.08 | 0.65 ± 0.02 | 0.2 ± 0.02 | 0.59 ± 0.02 | 0.59 ± 0.02 | 0.57 ± 0.02 | 0.76 ± 0.09 |
|  |  | RF | 0.79 ± 0.01 | 0.79 ± 0.01 | 0.59 ± 0.03 | 0.8 ± 0.01 | 0.8 ± 0.01 | 0.8 ± 0.02 | 0.78 ± 0.01 | 0.79 ± 0.01 | 0.59 ± 0.03 | 0.8 ± 0.01 | 0.8 ± 0.01 | 0.8 ± 0.02 | 0.78 ± 0.02 |
|  |  | SVM | 0.77 ± 0.01 | 0.79 ± 0.02 | 0.58 ± 0.04 | 0.79 ± 0.02 | 0.79 ± 0.02 | 0.8 ± 0.02 | 0.78 ± 0.02 | 0.79 ± 0.02 | 0.58 ± 0.04 | 0.79 ± 0.02 | 0.79 ± 0.02 | 0.8 ± 0.02 | 0.78 ± 0.03 |
|  | 85 | AB | 0.73 ± 0.01 | 0.74 ± 0.01 | 0.46 ± 0.03 | 0.73 ± 0.02 | 0.73 ± 0.02 | 0.72 ± 0.02 | 0.76 ± 0.02 | 0.74 ± 0.01 | 0.46 ± 0.03 | 0.73 ± 0.02 | 0.73 ± 0.02 | 0.72 ± 0.02 | 0.76 ± 0.03 |
|  |  | CART | 0.71 ± 0.01 | 0.7 ± 0.01 | 0.39 ± 0.02 | 0.69 ± 0.01 | 0.69 ± 0.01 | 0.69 ± 0.02 | 0.7 ± 0.02 | 0.69 ± 0.01 | 0.39 ± 0.03 | 0.69 ± 0.01 | 0.69 ± 0.01 | 0.69 ± 0.02 | 0.7 ± 0.02 |
|  |  | ET | 0.72 ± 0.01 | 0.73 ± 0.01 | 0.46 ± 0.03 | 0.73 ± 0.01 | 0.73 ± 0.01 | 0.73 ± 0.02 | 0.73 ± 0.01 | 0.73 ± 0.02 | 0.46 ± 0.03 | 0.73 ± 0.02 | 0.73 ± 0.02 | 0.73 ± 0.02 | 0.73 ± 0.02 |
|  |  | GBM | 0.71 ± 0.01 | 0.71 ± 0.01 | 0.42 ± 0.02 | 0.71 ± 0.01 | 0.71 ± 0.01 | 0.71 ± 0.01 | 0.71 ± 0.01 | 0.71 ± 0.01 | 0.42 ± 0.02 | 0.71 ± 0.01 | 0.71 ± 0.01 | 0.71 ± 0.01 | 0.71 ± 0.02 |
|  |  | KNN | 0.79 ± 0.01 | 0.8 ± 0.01 | 0.59 ± 0.02 | 0.8 ± 0.01 | 0.8 ± 0.01 | 0.79 ± 0.01 | 0.8 ± 0.02 | 0.8 ± 0.01 | 0.59 ± 0.02 | 0.8 ± 0.01 | 0.8 ± 0.01 | 0.79 ± 0.01 | 0.8 ± 0.02 |
|  |  | LDA | 0.65 ± 0.02 | 0.65 ± 0.02 | 0.3 ± 0.03 | 0.65 ± 0.01 | 0.65 ± 0.01 | 0.65 ± 0.02 | 0.64 ± 0.02 | 0.65 ± 0.02 | 0.3 ± 0.03 | 0.65 ± 0.01 | 0.65 ± 0.01 | 0.65 ± 0.02 | 0.64 ± 0.03 |
|  |  | LR | 0.66 ± 0.02 | 0.65 ± 0.01 | 0.32 ± 0.03 | 0.66 ± 0.01 | 0.66 ± 0.01 | 0.66 ± 0.01 | 0.65 ± 0.02 | 0.65 ± 0.02 | 0.31 ± 0.02 | 0.66 ± 0.01 | 0.66 ± 0.01 | 0.66 ± 0.01 | 0.65 ± 0.03 |
|  |  | NB | 0.67 ± 0.01 | 0.65 ± 0.02 | 0.2 ± 0.04 | 0.59 ± 0.02 | 0.59 ± 0.02 | 0.57 ± 0.02 | 0.77 ± 0.08 | 0.65 ± 0.02 | 0.2 ± 0.04 | 0.59 ± 0.02 | 0.59 ± 0.02 | 0.57 ± 0.02 | 0.77 ± 0.08 |
|  |  | RF | 0.79 ± 0.01 | 0.8 ± 0.01 | 0.6 ± 0.02 | 0.8 ± 0.01 | 0.8 ± 0.01 | 0.8 ± 0.02 | 0.79 ± 0.01 | 0.8 ± 0.01 | 0.6 ± 0.02 | 0.8 ± 0.01 | 0.8 ± 0.01 | 0.8 ± 0.01 | 0.79 ± 0.01 |
|  |  | SVM | 0.78 ± 0.01 | 0.78 ± 0.02 | 0.57 ± 0.04 | 0.79 ± 0.02 | 0.79 ± 0.02 | 0.8 ± 0.02 | 0.77 ± 0.02 | 0.78 ± 0.02 | 0.57 ± 0.04 | 0.79 ± 0.02 | 0.79 ± 0.02 | 0.79 ± 0.02 | 0.77 ± 0.03 |
|  | 95 | AB | 0.73 ± 0.01 | 0.74 ± 0.02 | 0.47 ± 0.03 | 0.73 ± 0.02 | 0.73 ± 0.02 | 0.73 ± 0.02 | 0.75 ± 0.02 | 0.74 ± 0.02 | 0.47 ± 0.03 | 0.73 ± 0.02 | 0.73 ± 0.02 | 0.72 ± 0.02 | 0.75 ± 0.03 |
|  |  | CART | 0.7 ± 0.01 | 0.68 ± 0.01 | 0.35 ± 0.03 | 0.68 ± 0.02 | 0.68 ± 0.02 | 0.67 ± 0.03 | 0.69 ± 0.04 | 0.68 ± 0.01 | 0.35 ± 0.03 | 0.68 ± 0.02 | 0.68 ± 0.02 | 0.67 ± 0.03 | 0.69 ± 0.04 |

|  |  |  |  | **Validation set** | | | | | | **Test set** | | | | | | |
| --- | --- | --- | --- | --- | --- | --- | --- | --- | --- | --- | --- | --- | --- | --- | --- | --- |
| **Activity threshold** | **PCC** | **Algorithm** | **F1 train** | **F1** | **MCC** | **ROC AUC** | **Accuracy** | **Precision** | **Recall** | **F1** | **MCC** | **ROC AUC** | **Accuracy** | **Precision** | **Recall** |  |
| GAP 20 | 95 | ET | 0.72 ± 0.01 | 0.72 ± 0.01 | 0.45 ± 0.03 | 0.72 ± 0.01 | 0.72 ± 0.01 | 0.72 ± 0.01 | 0.73 ± 0.01 | 0.72 ± 0.02 | 0.44 ± 0.03 | 0.72 ± 0.01 | 0.72 ± 0.01 | 0.72 ± 0.01 | 0.73 ± 0.02 |  |
|  |  | GBM | 0.71 ± 0.01 | 0.71 ± 0.01 | 0.42 ± 0.02 | 0.71 ± 0.01 | 0.71 ± 0.01 | 0.71 ± 0.01 | 0.71 ± 0.01 | 0.71 ± 0.01 | 0.42 ± 0.02 | 0.71 ± 0.01 | 0.71 ± 0.01 | 0.71 ± 0.01 | 0.71 ± 0.01 |  |
|  |  | KNN | 0.79 ± 0.01 | 0.8 ± 0.01 | 0.59 ± 0.02 | 0.79 ± 0.01 | 0.79 ± 0.01 | 0.79 ± 0.01 | 0.8 ± 0.02 | 0.8 ± 0.01 | 0.59 ± 0.02 | 0.79 ± 0.01 | 0.79 ± 0.01 | 0.79 ± 0.01 | 0.8 ± 0.02 |  |
|  |  | LDA | 0.66 ± 0.01 | 0.66 ± 0.02 | 0.32 ± 0.03 | 0.66 ± 0.01 | 0.66 ± 0.01 | 0.66 ± 0.01 | 0.66 ± 0.02 | 0.66 ± 0.02 | 0.32 ± 0.03 | 0.66 ± 0.01 | 0.66 ± 0.01 | 0.66 ± 0.01 | 0.66 ± 0.03 |  |
|  |  | LR | 0.67 ± 0.01 | 0.66 ± 0.02 | 0.33 ± 0.03 | 0.66 ± 0.02 | 0.66 ± 0.02 | 0.66 ± 0.01 | 0.66 ± 0.02 | 0.66 ± 0.02 | 0.33 ± 0.03 | 0.66 ± 0.02 | 0.66 ± 0.02 | 0.66 ± 0.02 | 0.66 ± 0.03 |  |
|  |  | NB | 0.65 ± 0.02 | 0.64 ± 0.03 | 0.17 ± 0.04 | 0.58 ± 0.02 | 0.58 ± 0.02 | 0.56 ± 0.02 | 0.76 ± 0.1 | 0.64 ± 0.03 | 0.17 ± 0.04 | 0.58 ± 0.02 | 0.58 ± 0.02 | 0.56 ± 0.02 | 0.76 ± 0.1 |  |
|  |  | RF | 0.79 ± 0.01 | 0.79 ± 0.01 | 0.59 ± 0.02 | 0.8 ± 0.01 | 0.8 ± 0.01 | 0.8 ± 0.01 | 0.79 ± 0.01 | 0.79 ± 0.01 | 0.59 ± 0.02 | 0.8 ± 0.01 | 0.8 ± 0.01 | 0.8 ± 0.01 | 0.78 ± 0.02 |  |
|  |  | SVM | 0.77 ± 0.01 | 0.78 ± 0.02 | 0.56 ± 0.04 | 0.78 ± 0.02 | 0.78 ± 0.02 | 0.78 ± 0.03 | 0.78 ± 0.03 | 0.78 ± 0.02 | 0.55 ± 0.04 | 0.78 ± 0.02 | 0.78 ± 0.02 | 0.78 ± 0.03 | 0.78 ± 0.03 |  |
|  | 100 | AB | 0.72 ± 0.01 | 0.74 ± 0.02 | 0.47 ± 0.04 | 0.74 ± 0.02 | 0.74 ± 0.02 | 0.73 ± 0.02 | 0.75 ± 0.02 | 0.74 ± 0.02 | 0.47 ± 0.04 | 0.74 ± 0.02 | 0.74 ± 0.02 | 0.73 ± 0.02 | 0.75 ± 0.03 |  |
|  |  | CART | 0.69 ± 0.0 | 0.69 ± 0.01 | 0.38 ± 0.03 | 0.69 ± 0.01 | 0.69 ± 0.01 | 0.69 ± 0.02 | 0.7 ± 0.02 | 0.69 ± 0.01 | 0.38 ± 0.03 | 0.69 ± 0.01 | 0.69 ± 0.01 | 0.69 ± 0.02 | 0.7 ± 0.02 |  |
|  |  | ET | 0.71 ± 0.01 | 0.72 ± 0.02 | 0.43 ± 0.04 | 0.72 ± 0.02 | 0.72 ± 0.02 | 0.71 ± 0.02 | 0.72 ± 0.02 | 0.72 ± 0.02 | 0.43 ± 0.03 | 0.72 ± 0.02 | 0.72 ± 0.02 | 0.71 ± 0.02 | 0.72 ± 0.02 |  |
|  |  | GBM | 0.71 ± 0.01 | 0.71 ± 0.01 | 0.41 ± 0.02 | 0.71 ± 0.01 | 0.71 ± 0.01 | 0.71 ± 0.02 | 0.71 ± 0.01 | 0.71 ± 0.01 | 0.41 ± 0.02 | 0.71 ± 0.01 | 0.71 ± 0.01 | 0.71 ± 0.02 | 0.71 ± 0.01 |  |
|  |  | KNN | 0.78 ± 0.01 | 0.79 ± 0.01 | 0.57 ± 0.02 | 0.79 ± 0.01 | 0.79 ± 0.01 | 0.78 ± 0.01 | 0.8 ± 0.02 | 0.79 ± 0.01 | 0.57 ± 0.02 | 0.79 ± 0.01 | 0.79 ± 0.01 | 0.78 ± 0.01 | 0.8 ± 0.02 |  |
|  |  | LDA | 0.67 ± 0.01 | 0.68 ± 0.02 | 0.35 ± 0.04 | 0.68 ± 0.02 | 0.68 ± 0.02 | 0.68 ± 0.02 | 0.67 ± 0.02 | 0.68 ± 0.02 | 0.35 ± 0.04 | 0.68 ± 0.02 | 0.68 ± 0.02 | 0.68 ± 0.02 | 0.68 ± 0.02 |  |
|  |  | LR | 0.68 ± 0.01 | 0.67 ± 0.01 | 0.34 ± 0.03 | 0.67 ± 0.01 | 0.67 ± 0.01 | 0.67 ± 0.01 | 0.68 ± 0.02 | 0.67 ± 0.02 | 0.34 ± 0.03 | 0.67 ± 0.02 | 0.67 ± 0.02 | 0.67 ± 0.02 | 0.67 ± 0.03 |  |
|  |  | NB | 0.65 ± 0.02 | 0.64 ± 0.03 | 0.17 ± 0.02 | 0.57 ± 0.01 | 0.57 ± 0.01 | 0.55 ± 0.02 | 0.78 ± 0.11 | 0.64 ± 0.03 | 0.17 ± 0.03 | 0.57 ± 0.01 | 0.57 ± 0.01 | 0.56 ± 0.02 | 0.78 ± 0.11 |  |
|  |  | RF | 0.78 ± 0.01 | 0.79 ± 0.01 | 0.58 ± 0.02 | 0.79 ± 0.01 | 0.79 ± 0.01 | 0.79 ± 0.01 | 0.78 ± 0.02 | 0.79 ± 0.01 | 0.58 ± 0.02 | 0.79 ± 0.01 | 0.79 ± 0.01 | 0.8 ± 0.01 | 0.78 ± 0.02 |  |
|  |  | SVM | 0.77 ± 0.01 | 0.78 ± 0.02 | 0.55 ± 0.03 | 0.78 ± 0.02 | 0.78 ± 0.02 | 0.77 ± 0.02 | 0.78 ± 0.03 | 0.78 ± 0.02 | 0.55 ± 0.03 | 0.78 ± 0.02 | 0.78 ± 0.02 | 0.77 ± 0.02 | 0.78 ± 0.03 |  |
|  |  |  |  |  |  |  |  |  |  |  |  |  |  |  |  |  |
| FIX 40 | 75 | AB | 0.6 ± 0.01 | 0.29 ± 0.15 | 0.2 ± 0.11 | 0.57 ± 0.04 | 0.65 ± 0.03 | 0.66 ± 0.11 | 0.19 ± 0.11 | 0.28 ± 0.15 | 0.2 ± 0.1 | 0.57 ± 0.04 | 0.65 ± 0.03 | 0.67 ± 0.09 | 0.19 ± 0.12 |  |

|  |  |  |  | **Validation set** | | | | | | **Test set** | | | | | |
| --- | --- | --- | --- | --- | --- | --- | --- | --- | --- | --- | --- | --- | --- | --- | --- |
| **Activity threshold** | **PCC** | **Algorithm** | **F1 train** | **F1** | **MCC** | **ROC AUC** | **Accuracy** | **Precision** | **Recall** | **F1** | **MCC** | **ROC AUC** | **Accuracy** | **Precision** | **Recall** |
| FIX 40 | 75 | CART | 0.6 ± 0.01 | 0.52 ± 0.05 | 0.26 ± 0.04 | 0.62 ± 0.02 | 0.66 ± 0.01 | 0.58 ± 0.02 | 0.47 ± 0.08 | 0.52 ± 0.05 | 0.26 ± 0.04 | 0.62 ± 0.02 | 0.66 ± 0.01 | 0.58 ± 0.02 | 0.47 ± 0.08 |
|  |  | ET | 0.63 ± 0.01 | 0.61 ± 0.02 | 0.39 ± 0.02 | 0.69 ± 0.01 | 0.72 ± 0.01 | 0.68 ± 0.01 | 0.55 ± 0.02 | 0.6 ± 0.02 | 0.39 ± 0.03 | 0.68 ± 0.01 | 0.71 ± 0.01 | 0.68 ± 0.03 | 0.55 ± 0.02 |
|  |  | GBM | 0.54 ± 0.01 | 0.55 ± 0.02 | 0.33 ± 0.03 | 0.65 ± 0.02 | 0.69 ± 0.01 | 0.64 ± 0.02 | 0.49 ± 0.03 | 0.55 ± 0.02 | 0.33 ± 0.03 | 0.65 ± 0.01 | 0.69 ± 0.01 | 0.64 ± 0.03 | 0.48 ± 0.02 |
|  |  | KNN | 0.67 ± 0.01 | 0.68 ± 0.01 | 0.46 ± 0.02 | 0.73 ± 0.01 | 0.74 ± 0.01 | 0.68 ± 0.02 | 0.68 ± 0.02 | 0.68 ± 0.01 | 0.46 ± 0.02 | 0.73 ± 0.01 | 0.74 ± 0.01 | 0.68 ± 0.02 | 0.68 ± 0.02 |
|  |  | LDA | 0.47 ± 0.02 | 0.4 ± 0.03 | 0.2 ± 0.03 | 0.58 ± 0.02 | 0.64 ± 0.01 | 0.59 ± 0.03 | 0.31 ± 0.03 | 0.4 ± 0.04 | 0.2 ± 0.03 | 0.58 ± 0.01 | 0.64 ± 0.01 | 0.59 ± 0.03 | 0.3 ± 0.04 |
|  |  | LR | 0.57 ± 0.01 | 0.4 ± 0.04 | 0.19 ± 0.04 | 0.58 ± 0.02 | 0.64 ± 0.01 | 0.58 ± 0.03 | 0.3 ± 0.04 | 0.39 ± 0.03 | 0.19 ± 0.03 | 0.58 ± 0.01 | 0.63 ± 0.01 | 0.58 ± 0.03 | 0.3 ± 0.04 |
|  |  | NB | 0.58 ± 0.01 | 0.08 ± 0.17 | 0.03 ± 0.07 | 0.52 ± 0.03 | 0.61 ± 0.01 | 0.09 ± 0.2 | 0.07 ± 0.15 | 0.07 ± 0.16 | 0.03 ± 0.06 | 0.51 ± 0.03 | 0.6 ± 0.0 | 0.08 ± 0.19 | 0.06 ± 0.14 |
|  |  | RF | 0.66 ± 0.01 | 0.65 ± 0.02 | 0.46 ± 0.02 | 0.72 ± 0.01 | 0.74 ± 0.01 | 0.71 ± 0.02 | 0.6 ± 0.02 | 0.65 ± 0.01 | 0.46 ± 0.02 | 0.72 ± 0.01 | 0.74 ± 0.01 | 0.71 ± 0.02 | 0.6 ± 0.02 |
|  |  | SVM | 0.67 ± 0.01 | 0.63 ± 0.03 | 0.43 ± 0.04 | 0.71 ± 0.02 | 0.73 ± 0.02 | 0.7 ± 0.03 | 0.57 ± 0.03 | 0.63 ± 0.02 | 0.43 ± 0.04 | 0.71 ± 0.02 | 0.73 ± 0.02 | 0.7 ± 0.03 | 0.57 ± 0.03 |
|  | 85 | AB | 0.6 ± 0.01 | 0.25 ± 0.12 | 0.19 ± 0.08 | 0.56 ± 0.04 | 0.64 ± 0.02 | 0.7 ± 0.06 | 0.16 ± 0.09 | 0.25 ± 0.11 | 0.19 ± 0.08 | 0.56 ± 0.04 | 0.64 ± 0.03 | 0.7 ± 0.06 | 0.16 ± 0.08 |
|  |  | CART | 0.6 ± 0.01 | 0.52 ± 0.04 | 0.26 ± 0.04 | 0.62 ± 0.02 | 0.66 ± 0.02 | 0.59 ± 0.03 | 0.47 ± 0.06 | 0.52 ± 0.04 | 0.26 ± 0.04 | 0.62 ± 0.02 | 0.66 ± 0.02 | 0.59 ± 0.04 | 0.47 ± 0.06 |
|  |  | ET | 0.63 ± 0.01 | 0.6 ± 0.01 | 0.39 ± 0.02 | 0.68 ± 0.01 | 0.71 ± 0.01 | 0.67 ± 0.01 | 0.55 ± 0.02 | 0.6 ± 0.01 | 0.39 ± 0.02 | 0.68 ± 0.01 | 0.71 ± 0.01 | 0.67 ± 0.02 | 0.55 ± 0.02 |
|  |  | GBM | 0.55 ± 0.02 | 0.56 ± 0.02 | 0.34 ± 0.03 | 0.66 ± 0.01 | 0.69 ± 0.01 | 0.64 ± 0.02 | 0.5 ± 0.02 | 0.56 ± 0.01 | 0.34 ± 0.02 | 0.66 ± 0.01 | 0.69 ± 0.01 | 0.65 ± 0.02 | 0.5 ± 0.02 |
|  |  | KNN | 0.67 ± 0.01 | 0.68 ± 0.02 | 0.46 ± 0.03 | 0.73 ± 0.02 | 0.74 ± 0.02 | 0.68 ± 0.02 | 0.68 ± 0.03 | 0.68 ± 0.02 | 0.46 ± 0.03 | 0.73 ± 0.02 | 0.74 ± 0.02 | 0.68 ± 0.02 | 0.68 ± 0.03 |
|  |  | LDA | 0.48 ± 0.03 | 0.42 ± 0.02 | 0.21 ± 0.03 | 0.59 ± 0.01 | 0.64 ± 0.01 | 0.6 ± 0.02 | 0.32 ± 0.03 | 0.42 ± 0.03 | 0.21 ± 0.02 | 0.59 ± 0.01 | 0.64 ± 0.01 | 0.6 ± 0.03 | 0.32 ± 0.03 |
|  |  | LR | 0.57 ± 0.01 | 0.4 ± 0.04 | 0.2 ± 0.04 | 0.58 ± 0.02 | 0.64 ± 0.01 | 0.59 ± 0.03 | 0.31 ± 0.04 | 0.4 ± 0.04 | 0.2 ± 0.03 | 0.58 ± 0.02 | 0.64 ± 0.01 | 0.58 ± 0.03 | 0.31 ± 0.04 |
|  |  | NB | 0.58 ± 0.01 | 0.11 ± 0.2 | 0.05 ± 0.08 | 0.52 ± 0.04 | 0.61 ± 0.01 | 0.2 ± 0.28 | 0.1 ± 0.18 | 0.1 ± 0.18 | 0.03 ± 0.05 | 0.51 ± 0.02 | 0.6 ± 0.01 | 0.2 ± 0.32 | 0.09 ± 0.16 |
|  |  | RF | 0.66 ± 0.01 | 0.66 ± 0.02 | 0.48 ± 0.02 | 0.73 ± 0.01 | 0.75 ± 0.01 | 0.73 ± 0.02 | 0.61 ± 0.02 | 0.66 ± 0.01 | 0.47 ± 0.02 | 0.73 ± 0.01 | 0.75 ± 0.01 | 0.73 ± 0.02 | 0.6 ± 0.02 |
|  |  | SVM | 0.67 ± 0.01 | 0.64 ± 0.02 | 0.44 ± 0.03 | 0.71 ± 0.01 | 0.74 ± 0.01 | 0.71 ± 0.02 | 0.58 ± 0.02 | 0.64 ± 0.02 | 0.44 ± 0.02 | 0.71 ± 0.01 | 0.74 ± 0.01 | 0.71 ± 0.02 | 0.58 ± 0.03 |
|  | 95 | AB | 0.61 ± 0.01 | 0.29 ± 0.15 | 0.22 ± 0.1 | 0.57 ± 0.05 | 0.65 ± 0.03 | 0.69 ± 0.08 | 0.2 ± 0.12 | 0.3 ± 0.13 | 0.23 ± 0.08 | 0.57 ± 0.04 | 0.65 ± 0.03 | 0.72 ± 0.05 | 0.2 ± 0.11 |

|  |  |  |  | **Validation set** | | | | | | **Test set** | | | | | | |
| --- | --- | --- | --- | --- | --- | --- | --- | --- | --- | --- | --- | --- | --- | --- | --- | --- |
| **Activity threshold** | **PCC** | **Algorithm** | **F1 train** | **F1** | **MCC** | **ROC AUC** | **Accuracy** | **Precision** | **Recall** | **F1** | **MCC** | **ROC AUC** | **Accuracy** | **Precision** | **Recall** |  |
| FIX 40 | 95 | CART | 0.6 ± 0.0 | 0.53 ± 0.05 | 0.27 ± 0.04 | 0.63 ± 0.02 | 0.66 ± 0.02 | 0.59 ± 0.02 | 0.49 ± 0.07 | 0.53 ± 0.05 | 0.28 ± 0.04 | 0.63 ± 0.02 | 0.66 ± 0.02 | 0.59 ± 0.03 | 0.49 ± 0.07 |  |
|  |  | ET | 0.63 ± 0.0 | 0.6 ± 0.02 | 0.38 ± 0.02 | 0.68 ± 0.01 | 0.71 ± 0.01 | 0.66 ± 0.02 | 0.54 ± 0.02 | 0.6 ± 0.02 | 0.38 ± 0.02 | 0.68 ± 0.01 | 0.71 ± 0.01 | 0.66 ± 0.02 | 0.54 ± 0.03 |  |
|  |  | GBM | 0.55 ± 0.02 | 0.56 ± 0.02 | 0.34 ± 0.03 | 0.66 ± 0.02 | 0.69 ± 0.01 | 0.64 ± 0.02 | 0.5 ± 0.03 | 0.56 ± 0.02 | 0.34 ± 0.03 | 0.66 ± 0.01 | 0.69 ± 0.02 | 0.65 ± 0.03 | 0.5 ± 0.03 |  |
|  |  | KNN | 0.67 ± 0.01 | 0.68 ± 0.02 | 0.47 ± 0.03 | 0.73 ± 0.01 | 0.74 ± 0.01 | 0.68 ± 0.02 | 0.68 ± 0.03 | 0.68 ± 0.02 | 0.47 ± 0.03 | 0.73 ± 0.01 | 0.74 ± 0.01 | 0.68 ± 0.02 | 0.68 ± 0.03 |  |
|  |  | LDA | 0.51 ± 0.02 | 0.45 ± 0.02 | 0.23 ± 0.02 | 0.6 ± 0.01 | 0.65 ± 0.01 | 0.6 ± 0.02 | 0.36 ± 0.02 | 0.44 ± 0.03 | 0.23 ± 0.02 | 0.6 ± 0.01 | 0.65 ± 0.01 | 0.6 ± 0.02 | 0.35 ± 0.03 |  |
|  |  | LR | 0.58 ± 0.0 | 0.44 ± 0.02 | 0.22 ± 0.02 | 0.6 ± 0.01 | 0.65 ± 0.01 | 0.6 ± 0.02 | 0.34 ± 0.02 | 0.43 ± 0.03 | 0.22 ± 0.03 | 0.59 ± 0.01 | 0.64 ± 0.01 | 0.6 ± 0.02 | 0.34 ± 0.04 |  |
|  |  | NB | 0.57 ± 0.02 | 0.08 ± 0.19 | 0.04 ± 0.08 | 0.52 ± 0.04 | 0.61 ± 0.01 | 0.09 ± 0.2 | 0.08 ± 0.18 | 0.08 ± 0.17 | 0.02 ± 0.04 | 0.51 ± 0.02 | 0.6 ± 0.01 | 0.08 ± 0.18 | 0.07 ± 0.16 |  |
|  |  | RF | 0.66 ± 0.01 | 0.66 ± 0.02 | 0.47 ± 0.03 | 0.73 ± 0.01 | 0.75 ± 0.01 | 0.72 ± 0.02 | 0.61 ± 0.02 | 0.66 ± 0.01 | 0.47 ± 0.02 | 0.72 ± 0.01 | 0.75 ± 0.01 | 0.72 ± 0.02 | 0.61 ± 0.02 |  |
|  |  | SVM | 0.67 ± 0.01 | 0.63 ± 0.02 | 0.42 ± 0.03 | 0.7 ± 0.01 | 0.73 ± 0.01 | 0.69 ± 0.02 | 0.58 ± 0.02 | 0.63 ± 0.02 | 0.43 ± 0.02 | 0.71 ± 0.01 | 0.73 ± 0.01 | 0.7 ± 0.02 | 0.58 ± 0.03 |  |
|  | 100 | AB | 0.61 ± 0.01 | 0.39 ± 0.16 | 0.27 ± 0.1 | 0.6 ± 0.05 | 0.67 ± 0.03 | 0.69 ± 0.05 | 0.29 ± 0.14 | 0.4 ± 0.15 | 0.28 ± 0.08 | 0.61 ± 0.05 | 0.67 ± 0.03 | 0.7 ± 0.06 | 0.3 ± 0.14 |  |
|  |  | CART | 0.6 ± 0.01 | 0.48 ± 0.08 | 0.22 ± 0.06 | 0.61 ± 0.03 | 0.64 ± 0.02 | 0.56 ± 0.02 | 0.43 ± 0.1 | 0.48 ± 0.09 | 0.22 ± 0.06 | 0.61 ± 0.04 | 0.64 ± 0.02 | 0.56 ± 0.03 | 0.43 ± 0.11 |  |
|  |  | ET | 0.62 ± 0.01 | 0.59 ± 0.01 | 0.37 ± 0.02 | 0.68 ± 0.01 | 0.7 ± 0.01 | 0.66 ± 0.01 | 0.53 ± 0.01 | 0.59 ± 0.01 | 0.37 ± 0.02 | 0.68 ± 0.01 | 0.71 ± 0.01 | 0.67 ± 0.02 | 0.53 ± 0.03 |  |
|  |  | GBM | 0.55 ± 0.02 | 0.56 ± 0.03 | 0.33 ± 0.04 | 0.65 ± 0.02 | 0.69 ± 0.02 | 0.64 ± 0.02 | 0.49 ± 0.03 | 0.56 ± 0.03 | 0.33 ± 0.04 | 0.66 ± 0.02 | 0.69 ± 0.02 | 0.64 ± 0.04 | 0.49 ± 0.04 |  |
|  |  | KNN | 0.67 ± 0.01 | 0.67 ± 0.02 | 0.44 ± 0.03 | 0.72 ± 0.02 | 0.73 ± 0.01 | 0.66 ± 0.02 | 0.67 ± 0.03 | 0.67 ± 0.02 | 0.44 ± 0.03 | 0.72 ± 0.02 | 0.73 ± 0.01 | 0.66 ± 0.02 | 0.67 ± 0.03 |  |
|  |  | LDA | 0.52 ± 0.02 | 0.46 ± 0.03 | 0.24 ± 0.03 | 0.61 ± 0.01 | 0.66 ± 0.01 | 0.61 ± 0.02 | 0.37 ± 0.03 | 0.46 ± 0.03 | 0.24 ± 0.02 | 0.6 ± 0.01 | 0.65 ± 0.01 | 0.6 ± 0.02 | 0.37 ± 0.04 |  |
|  |  | LR | 0.59 ± 0.01 | 0.44 ± 0.02 | 0.23 ± 0.02 | 0.6 ± 0.01 | 0.65 ± 0.01 | 0.6 ± 0.02 | 0.35 ± 0.02 | 0.44 ± 0.04 | 0.22 ± 0.03 | 0.6 ± 0.01 | 0.65 ± 0.01 | 0.6 ± 0.02 | 0.35 ± 0.04 |  |
|  |  | NB | 0.57 ± 0.02 | 0.08 ± 0.18 | 0.04 ± 0.08 | 0.52 ± 0.04 | 0.61 ± 0.01 | 0.09 ± 0.2 | 0.08 ± 0.17 | 0.08 ± 0.17 | 0.02 ± 0.05 | 0.51 ± 0.02 | 0.6 ± 0.0 | 0.08 ± 0.18 | 0.07 ± 0.16 |  |
|  |  | RF | 0.66 ± 0.01 | 0.66 ± 0.02 | 0.46 ± 0.02 | 0.72 ± 0.01 | 0.75 ± 0.01 | 0.72 ± 0.02 | 0.6 ± 0.02 | 0.65 ± 0.01 | 0.46 ± 0.02 | 0.72 ± 0.01 | 0.75 ± 0.01 | 0.72 ± 0.02 | 0.6 ± 0.02 |  |
|  |  | SVM | 0.67 ± 0.01 | 0.62 ± 0.02 | 0.42 ± 0.03 | 0.7 ± 0.02 | 0.73 ± 0.01 | 0.69 ± 0.02 | 0.57 ± 0.03 | 0.63 ± 0.02 | 0.42 ± 0.03 | 0.7 ± 0.01 | 0.73 ± 0.01 | 0.69 ± 0.03 | 0.57 ± 0.04 |  |
|  |  |  |  |  |  |  |  |  |  |  |  |  |  |  |  |  |
| FIX 45 | 75 | AB | 0.63 ± 0.01 | 0.44 ± 0.1 | 0.28 ± 0.05 | 0.61 ± 0.03 | 0.64 ± 0.03 | 0.72 ± 0.02 | 0.33 ± 0.1 | 0.44 ± 0.1 | 0.27 ± 0.05 | 0.61 ± 0.03 | 0.64 ± 0.03 | 0.72 ± 0.04 | 0.33 ± 0.1 |  |
|  |  | CART | 0.63 ± 0.0 | 0.59 ± 0.03 | 0.27 ± 0.05 | 0.63 ± 0.02 | 0.64 ± 0.02 | 0.61 ± 0.03 | 0.57 ± 0.04 | 0.59 ± 0.03 | 0.27 ± 0.05 | 0.63 ± 0.02 | 0.64 ± 0.02 | 0.61 ± 0.03 | 0.57 ± 0.04 |  |

|  |  |  |  | **Validation set** | | | | | | **Test set** | | | | | |
| --- | --- | --- | --- | --- | --- | --- | --- | --- | --- | --- | --- | --- | --- | --- | --- |
| **Activity threshold** | **PCC** | **Algorithm** | **F1 train** | **F1** | **MCC** | **ROC AUC** | **Accuracy** | **Precision** | **Recall** | **F1** | **MCC** | **ROC AUC** | **Accuracy** | **Precision** | **Recall** |
| FIX 45 | 75 | ET | 0.65 ± 0.01 | 0.64 ± 0.02 | 0.36 ± 0.03 | 0.68 ± 0.01 | 0.69 ± 0.01 | 0.67 ± 0.02 | 0.61 ± 0.02 | 0.64 ± 0.02 | 0.36 ± 0.03 | 0.68 ± 0.01 | 0.69 ± 0.01 | 0.67 ± 0.02 | 0.61 ± 0.02 |
|  |  | GBM | 0.6 ± 0.02 | 0.61 ± 0.02 | 0.33 ± 0.03 | 0.66 ± 0.02 | 0.67 ± 0.02 | 0.65 ± 0.02 | 0.57 ± 0.02 | 0.61 ± 0.02 | 0.32 ± 0.03 | 0.66 ± 0.02 | 0.67 ± 0.02 | 0.65 ± 0.02 | 0.57 ± 0.02 |
|  |  | KNN | 0.7 ± 0.01 | 0.71 ± 0.02 | 0.47 ± 0.04 | 0.74 ± 0.02 | 0.74 ± 0.02 | 0.7 ± 0.02 | 0.72 ± 0.02 | 0.71 ± 0.02 | 0.47 ± 0.04 | 0.74 ± 0.02 | 0.74 ± 0.02 | 0.7 ± 0.02 | 0.72 ± 0.02 |
|  |  | LDA | 0.54 ± 0.01 | 0.51 ± 0.03 | 0.22 ± 0.04 | 0.6 ± 0.02 | 0.62 ± 0.02 | 0.6 ± 0.02 | 0.44 ± 0.04 | 0.51 ± 0.02 | 0.22 ± 0.03 | 0.6 ± 0.01 | 0.62 ± 0.02 | 0.61 ± 0.03 | 0.44 ± 0.03 |
|  |  | LR | 0.59 ± 0.01 | 0.51 ± 0.03 | 0.22 ± 0.03 | 0.6 ± 0.02 | 0.62 ± 0.01 | 0.6 ± 0.02 | 0.45 ± 0.04 | 0.51 ± 0.02 | 0.22 ± 0.02 | 0.6 ± 0.01 | 0.62 ± 0.01 | 0.61 ± 0.02 | 0.44 ± 0.03 |
|  |  | NB | 0.6 ± 0.02 | 0.48 ± 0.15 | 0.14 ± 0.06 | 0.57 ± 0.03 | 0.58 ± 0.02 | 0.49 ± 0.15 | 0.46 ± 0.15 | 0.47 ± 0.14 | 0.13 ± 0.05 | 0.56 ± 0.03 | 0.58 ± 0.02 | 0.49 ± 0.15 | 0.46 ± 0.14 |
|  |  | RF | 0.7 ± 0.01 | 0.7 ± 0.01 | 0.48 ± 0.02 | 0.74 ± 0.01 | 0.74 ± 0.01 | 0.73 ± 0.02 | 0.67 ± 0.01 | 0.7 ± 0.01 | 0.47 ± 0.02 | 0.73 ± 0.01 | 0.74 ± 0.01 | 0.73 ± 0.02 | 0.67 ± 0.01 |
|  |  | SVM | 0.7 ± 0.01 | 0.69 ± 0.01 | 0.45 ± 0.02 | 0.72 ± 0.01 | 0.73 ± 0.01 | 0.71 ± 0.01 | 0.67 ± 0.02 | 0.69 ± 0.01 | 0.45 ± 0.02 | 0.72 ± 0.01 | 0.73 ± 0.01 | 0.71 ± 0.01 | 0.67 ± 0.02 |
|  | 85 | AB | 0.64 ± 0.01 | 0.5 ± 0.07 | 0.3 ± 0.05 | 0.63 ± 0.03 | 0.66 ± 0.02 | 0.7 ± 0.02 | 0.4 ± 0.09 | 0.51 ± 0.06 | 0.31 ± 0.02 | 0.63 ± 0.02 | 0.66 ± 0.01 | 0.72 ± 0.03 | 0.4 ± 0.07 |
|  |  | CART | 0.63 ± 0.01 | 0.6 ± 0.02 | 0.29 ± 0.03 | 0.64 ± 0.02 | 0.65 ± 0.01 | 0.62 ± 0.02 | 0.58 ± 0.02 | 0.6 ± 0.02 | 0.29 ± 0.03 | 0.64 ± 0.02 | 0.65 ± 0.01 | 0.62 ± 0.02 | 0.58 ± 0.02 |
|  |  | ET | 0.65 ± 0.01 | 0.65 ± 0.02 | 0.38 ± 0.03 | 0.69 ± 0.02 | 0.7 ± 0.01 | 0.67 ± 0.02 | 0.62 ± 0.02 | 0.65 ± 0.02 | 0.38 ± 0.03 | 0.69 ± 0.01 | 0.7 ± 0.01 | 0.68 ± 0.02 | 0.62 ± 0.02 |
|  |  | GBM | 0.6 ± 0.02 | 0.61 ± 0.02 | 0.32 ± 0.03 | 0.66 ± 0.02 | 0.67 ± 0.02 | 0.64 ± 0.02 | 0.58 ± 0.02 | 0.61 ± 0.02 | 0.32 ± 0.03 | 0.66 ± 0.02 | 0.67 ± 0.02 | 0.64 ± 0.02 | 0.58 ± 0.02 |
|  |  | KNN | 0.7 ± 0.01 | 0.71 ± 0.02 | 0.47 ± 0.03 | 0.74 ± 0.02 | 0.74 ± 0.02 | 0.71 ± 0.02 | 0.72 ± 0.02 | 0.71 ± 0.02 | 0.47 ± 0.03 | 0.74 ± 0.02 | 0.74 ± 0.02 | 0.71 ± 0.02 | 0.72 ± 0.02 |
|  |  | LDA | 0.55 ± 0.01 | 0.52 ± 0.03 | 0.23 ± 0.04 | 0.61 ± 0.02 | 0.62 ± 0.02 | 0.61 ± 0.02 | 0.45 ± 0.04 | 0.52 ± 0.02 | 0.23 ± 0.03 | 0.61 ± 0.01 | 0.62 ± 0.02 | 0.61 ± 0.03 | 0.45 ± 0.03 |
|  |  | LR | 0.6 ± 0.01 | 0.52 ± 0.03 | 0.23 ± 0.03 | 0.61 ± 0.02 | 0.62 ± 0.02 | 0.61 ± 0.02 | 0.45 ± 0.04 | 0.52 ± 0.01 | 0.22 ± 0.03 | 0.6 ± 0.01 | 0.62 ± 0.01 | 0.61 ± 0.03 | 0.45 ± 0.02 |
|  |  | NB | 0.6 ± 0.02 | 0.43 ± 0.19 | 0.13 ± 0.06 | 0.56 ± 0.03 | 0.58 ± 0.02 | 0.45 ± 0.2 | 0.42 ± 0.19 | 0.43 ± 0.19 | 0.12 ± 0.06 | 0.56 ± 0.03 | 0.58 ± 0.02 | 0.45 ± 0.2 | 0.42 ± 0.19 |
|  |  | RF | 0.7 ± 0.01 | 0.71 ± 0.01 | 0.49 ± 0.02 | 0.74 ± 0.01 | 0.75 ± 0.01 | 0.74 ± 0.02 | 0.68 ± 0.01 | 0.71 ± 0.01 | 0.49 ± 0.02 | 0.74 ± 0.01 | 0.75 ± 0.01 | 0.74 ± 0.02 | 0.68 ± 0.01 |
|  |  | SVM | 0.7 ± 0.0 | 0.69 ± 0.01 | 0.44 ± 0.03 | 0.72 ± 0.01 | 0.73 ± 0.01 | 0.71 ± 0.02 | 0.67 ± 0.02 | 0.69 ± 0.02 | 0.44 ± 0.03 | 0.72 ± 0.02 | 0.72 ± 0.02 | 0.7 ± 0.02 | 0.67 ± 0.02 |
|  | 95 | AB | 0.64 ± 0.01 | 0.5 ± 0.08 | 0.31 ± 0.04 | 0.63 ± 0.03 | 0.66 ± 0.02 | 0.72 ± 0.02 | 0.4 ± 0.11 | 0.51 ± 0.08 | 0.32 ± 0.04 | 0.64 ± 0.03 | 0.66 ± 0.02 | 0.72 ± 0.03 | 0.4 ± 0.1 |
|  |  | CART | 0.63 ± 0.01 | 0.59 ± 0.01 | 0.28 ± 0.02 | 0.64 ± 0.01 | 0.65 ± 0.01 | 0.61 ± 0.02 | 0.57 ± 0.02 | 0.59 ± 0.01 | 0.28 ± 0.02 | 0.64 ± 0.01 | 0.65 ± 0.01 | 0.61 ± 0.02 | 0.57 ± 0.02 |
|  |  | ET | 0.66 ± 0.01 | 0.65 ± 0.02 | 0.38 ± 0.04 | 0.69 ± 0.02 | 0.7 ± 0.02 | 0.67 ± 0.02 | 0.62 ± 0.02 | 0.65 ± 0.02 | 0.38 ± 0.03 | 0.69 ± 0.02 | 0.7 ± 0.02 | 0.68 ± 0.02 | 0.62 ± 0.02 |

|  |  |  |  | **Validation set** | | | | | | **Test set** | | | | | | |
| --- | --- | --- | --- | --- | --- | --- | --- | --- | --- | --- | --- | --- | --- | --- | --- | --- |
| **Activity threshold** | **PCC** | **Algorithm** | **F1 train** | **F1** | **MCC** | **ROC AUC** | **Accuracy** | **Precision** | **Recall** | **F1** | **MCC** | **ROC AUC** | **Accuracy** | **Precision** | **Recall** |  |
| FIX 45 | 95 | GBM | 0.61 ± 0.02 | 0.61 ± 0.02 | 0.33 ± 0.03 | 0.66 ± 0.02 | 0.67 ± 0.02 | 0.65 ± 0.02 | 0.58 ± 0.02 | 0.61 ± 0.01 | 0.33 ± 0.03 | 0.66 ± 0.01 | 0.67 ± 0.01 | 0.65 ± 0.02 | 0.58 ± 0.02 |  |
|  |  | KNN | 0.71 ± 0.01 | 0.71 ± 0.02 | 0.47 ± 0.04 | 0.74 ± 0.02 | 0.74 ± 0.02 | 0.71 ± 0.02 | 0.72 ± 0.02 | 0.71 ± 0.02 | 0.47 ± 0.04 | 0.74 ± 0.02 | 0.74 ± 0.02 | 0.71 ± 0.02 | 0.72 ± 0.02 |  |
|  |  | LDA | 0.57 ± 0.01 | 0.55 ± 0.02 | 0.25 ± 0.03 | 0.62 ± 0.02 | 0.64 ± 0.01 | 0.62 ± 0.02 | 0.5 ± 0.03 | 0.55 ± 0.01 | 0.25 ± 0.03 | 0.62 ± 0.01 | 0.63 ± 0.01 | 0.62 ± 0.02 | 0.49 ± 0.02 |  |
|  |  | LR | 0.61 ± 0.01 | 0.54 ± 0.03 | 0.25 ± 0.04 | 0.62 ± 0.02 | 0.63 ± 0.02 | 0.61 ± 0.02 | 0.49 ± 0.04 | 0.54 ± 0.02 | 0.24 ± 0.03 | 0.62 ± 0.02 | 0.63 ± 0.02 | 0.61 ± 0.03 | 0.48 ± 0.02 |  |
|  |  | NB | 0.58 ± 0.03 | 0.44 ± 0.2 | 0.13 ± 0.07 | 0.56 ± 0.03 | 0.58 ± 0.02 | 0.45 ± 0.2 | 0.43 ± 0.2 | 0.44 ± 0.2 | 0.13 ± 0.07 | 0.56 ± 0.03 | 0.58 ± 0.02 | 0.45 ± 0.2 | 0.43 ± 0.2 |  |
|  |  | RF | 0.7 ± 0.01 | 0.7 ± 0.02 | 0.48 ± 0.03 | 0.74 ± 0.01 | 0.74 ± 0.01 | 0.73 ± 0.02 | 0.68 ± 0.02 | 0.7 ± 0.02 | 0.48 ± 0.03 | 0.74 ± 0.01 | 0.74 ± 0.01 | 0.73 ± 0.02 | 0.68 ± 0.01 |  |
|  |  | SVM | 0.7 ± 0.0 | 0.68 ± 0.01 | 0.44 ± 0.02 | 0.72 ± 0.01 | 0.72 ± 0.01 | 0.7 ± 0.01 | 0.66 ± 0.02 | 0.68 ± 0.01 | 0.44 ± 0.02 | 0.72 ± 0.01 | 0.72 ± 0.01 | 0.7 ± 0.02 | 0.66 ± 0.02 |  |
|  | 100 | AB | 0.65 ± 0.01 | 0.54 ± 0.06 | 0.34 ± 0.04 | 0.65 ± 0.03 | 0.67 ± 0.02 | 0.72 ± 0.02 | 0.44 ± 0.09 | 0.55 ± 0.06 | 0.34 ± 0.03 | 0.65 ± 0.02 | 0.67 ± 0.02 | 0.72 ± 0.03 | 0.45 ± 0.08 |  |
|  |  | CART | 0.63 ± 0.01 | 0.51 ± 0.2 | 0.24 ± 0.11 | 0.62 ± 0.05 | 0.63 ± 0.04 | 0.58 ± 0.07 | 0.5 ± 0.21 | 0.51 ± 0.2 | 0.24 ± 0.11 | 0.62 ± 0.05 | 0.63 ± 0.04 | 0.58 ± 0.07 | 0.5 ± 0.21 |  |
|  |  | ET | 0.65 ± 0.0 | 0.64 ± 0.02 | 0.37 ± 0.03 | 0.68 ± 0.02 | 0.69 ± 0.02 | 0.66 ± 0.02 | 0.62 ± 0.02 | 0.64 ± 0.02 | 0.37 ± 0.03 | 0.68 ± 0.02 | 0.69 ± 0.01 | 0.66 ± 0.02 | 0.62 ± 0.02 |  |
|  |  | GBM | 0.61 ± 0.02 | 0.62 ± 0.02 | 0.34 ± 0.03 | 0.66 ± 0.02 | 0.67 ± 0.02 | 0.65 ± 0.02 | 0.58 ± 0.02 | 0.61 ± 0.02 | 0.33 ± 0.03 | 0.66 ± 0.02 | 0.67 ± 0.02 | 0.65 ± 0.02 | 0.58 ± 0.02 |  |
|  |  | KNN | 0.7 ± 0.01 | 0.7 ± 0.02 | 0.46 ± 0.03 | 0.73 ± 0.02 | 0.73 ± 0.02 | 0.7 ± 0.02 | 0.71 ± 0.01 | 0.7 ± 0.02 | 0.46 ± 0.03 | 0.73 ± 0.02 | 0.73 ± 0.02 | 0.7 ± 0.02 | 0.71 ± 0.01 |  |
|  |  | LDA | 0.58 ± 0.01 | 0.56 ± 0.02 | 0.27 ± 0.03 | 0.63 ± 0.01 | 0.64 ± 0.01 | 0.62 ± 0.01 | 0.51 ± 0.03 | 0.56 ± 0.01 | 0.27 ± 0.02 | 0.63 ± 0.01 | 0.64 ± 0.01 | 0.62 ± 0.02 | 0.51 ± 0.02 |  |
|  |  | LR | 0.61 ± 0.01 | 0.55 ± 0.02 | 0.26 ± 0.03 | 0.62 ± 0.02 | 0.64 ± 0.02 | 0.62 ± 0.02 | 0.5 ± 0.03 | 0.55 ± 0.02 | 0.26 ± 0.03 | 0.62 ± 0.01 | 0.64 ± 0.01 | 0.62 ± 0.02 | 0.5 ± 0.02 |  |
|  |  | NB | 0.59 ± 0.02 | 0.44 ± 0.2 | 0.12 ± 0.06 | 0.56 ± 0.03 | 0.57 ± 0.02 | 0.44 ± 0.2 | 0.44 ± 0.2 | 0.44 ± 0.2 | 0.12 ± 0.06 | 0.56 ± 0.03 | 0.57 ± 0.02 | 0.44 ± 0.2 | 0.44 ± 0.2 |  |
|  |  | RF | 0.69 ± 0.01 | 0.7 ± 0.02 | 0.46 ± 0.03 | 0.73 ± 0.02 | 0.74 ± 0.02 | 0.72 ± 0.02 | 0.67 ± 0.02 | 0.7 ± 0.02 | 0.46 ± 0.04 | 0.73 ± 0.02 | 0.74 ± 0.02 | 0.72 ± 0.03 | 0.67 ± 0.01 |  |
|  |  | SVM | 0.7 ± 0.0 | 0.68 ± 0.01 | 0.43 ± 0.02 | 0.71 ± 0.01 | 0.72 ± 0.01 | 0.7 ± 0.01 | 0.66 ± 0.02 | 0.68 ± 0.01 | 0.43 ± 0.02 | 0.71 ± 0.01 | 0.72 ± 0.01 | 0.7 ± 0.01 | 0.66 ± 0.02 |  |
|  |  |  |  |  |  |  |  |  |  |  |  |  |  |  |  |  |
| FIX 50 | 75 | AB | 0.67 ± 0.01 | 0.68 ± 0.02 | 0.35 ± 0.02 | 0.67 ± 0.01 | 0.67 ± 0.01 | 0.67 ± 0.02 | 0.69 ± 0.06 | 0.68 ± 0.02 | 0.35 ± 0.02 | 0.68 ± 0.01 | 0.68 ± 0.01 | 0.67 ± 0.03 | 0.7 ± 0.06 |  |
|  |  | CART | 0.67 ± 0.01 | 0.65 ± 0.01 | 0.3 ± 0.02 | 0.65 ± 0.01 | 0.65 ± 0.01 | 0.64 ± 0.02 | 0.66 ± 0.04 | 0.65 ± 0.01 | 0.3 ± 0.03 | 0.65 ± 0.01 | 0.65 ± 0.01 | 0.65 ± 0.02 | 0.66 ± 0.04 |  |
|  |  | ET | 0.68 ± 0.01 | 0.69 ± 0.01 | 0.39 ± 0.02 | 0.69 ± 0.01 | 0.7 ± 0.01 | 0.69 ± 0.01 | 0.69 ± 0.02 | 0.69 ± 0.01 | 0.39 ± 0.01 | 0.69 ± 0.01 | 0.69 ± 0.01 | 0.69 ± 0.0 | 0.69 ± 0.02 |  |

|  |  |  |  | **Validation set** | | | | | | **Test set** | | | | | |
| --- | --- | --- | --- | --- | --- | --- | --- | --- | --- | --- | --- | --- | --- | --- | --- |
| **Activity threshold** | **PCC** | **Algorithm** | **F1 train** | **F1** | **MCC** | **ROC AUC** | **Accuracy** | **Precision** | **Recall** | **F1** | **MCC** | **ROC AUC** | **Accuracy** | **Precision** | **Recall** |
| FIX 50 | 75 | GBM | 0.66 ± 0.01 | 0.66 ± 0.01 | 0.31 ± 0.03 | 0.66 ± 0.01 | 0.66 ± 0.01 | 0.65 ± 0.01 | 0.66 ± 0.01 | 0.65 ± 0.01 | 0.31 ± 0.03 | 0.66 ± 0.01 | 0.66 ± 0.01 | 0.65 ± 0.02 | 0.66 ± 0.02 |
|  |  | KNN | 0.73 ± 0.01 | 0.74 ± 0.01 | 0.49 ± 0.02 | 0.74 ± 0.01 | 0.74 ± 0.01 | 0.74 ± 0.01 | 0.75 ± 0.02 | 0.74 ± 0.01 | 0.49 ± 0.02 | 0.74 ± 0.01 | 0.74 ± 0.01 | 0.74 ± 0.01 | 0.75 ± 0.02 |
|  |  | LDA | 0.61 ± 0.0 | 0.62 ± 0.01 | 0.24 ± 0.02 | 0.62 ± 0.01 | 0.62 ± 0.01 | 0.62 ± 0.01 | 0.62 ± 0.01 | 0.62 ± 0.02 | 0.24 ± 0.02 | 0.62 ± 0.01 | 0.62 ± 0.01 | 0.62 ± 0.01 | 0.62 ± 0.04 |
|  |  | LR | 0.62 ± 0.0 | 0.62 ± 0.01 | 0.25 ± 0.02 | 0.62 ± 0.01 | 0.62 ± 0.01 | 0.62 ± 0.01 | 0.62 ± 0.01 | 0.62 ± 0.01 | 0.24 ± 0.02 | 0.62 ± 0.01 | 0.62 ± 0.01 | 0.62 ± 0.01 | 0.62 ± 0.03 |
|  |  | NB | 0.65 ± 0.01 | 0.62 ± 0.02 | 0.17 ± 0.03 | 0.58 ± 0.02 | 0.58 ± 0.02 | 0.57 ± 0.02 | 0.68 ± 0.08 | 0.62 ± 0.02 | 0.17 ± 0.02 | 0.58 ± 0.02 | 0.58 ± 0.02 | 0.57 ± 0.02 | 0.68 ± 0.08 |
|  |  | RF | 0.74 ± 0.01 | 0.75 ± 0.02 | 0.51 ± 0.03 | 0.75 ± 0.01 | 0.75 ± 0.01 | 0.76 ± 0.02 | 0.74 ± 0.02 | 0.75 ± 0.01 | 0.5 ± 0.03 | 0.75 ± 0.01 | 0.75 ± 0.01 | 0.76 ± 0.02 | 0.74 ± 0.02 |
|  |  | SVM | 0.73 ± 0.01 | 0.74 ± 0.01 | 0.49 ± 0.03 | 0.74 ± 0.02 | 0.74 ± 0.02 | 0.74 ± 0.02 | 0.74 ± 0.02 | 0.74 ± 0.01 | 0.49 ± 0.03 | 0.74 ± 0.01 | 0.74 ± 0.01 | 0.74 ± 0.02 | 0.74 ± 0.02 |
|  | 85 | AB | 0.68 ± 0.01 | 0.69 ± 0.01 | 0.38 ± 0.02 | 0.69 ± 0.01 | 0.69 ± 0.01 | 0.68 ± 0.02 | 0.7 ± 0.04 | 0.69 ± 0.01 | 0.38 ± 0.02 | 0.69 ± 0.01 | 0.69 ± 0.01 | 0.68 ± 0.02 | 0.7 ± 0.04 |
|  |  | CART | 0.66 ± 0.0 | 0.64 ± 0.02 | 0.28 ± 0.02 | 0.64 ± 0.01 | 0.64 ± 0.01 | 0.64 ± 0.01 | 0.65 ± 0.03 | 0.65 ± 0.02 | 0.29 ± 0.02 | 0.64 ± 0.01 | 0.64 ± 0.01 | 0.64 ± 0.02 | 0.66 ± 0.04 |
|  |  | ET | 0.68 ± 0.0 | 0.7 ± 0.01 | 0.4 ± 0.02 | 0.7 ± 0.01 | 0.7 ± 0.01 | 0.7 ± 0.01 | 0.7 ± 0.02 | 0.7 ± 0.01 | 0.4 ± 0.02 | 0.7 ± 0.01 | 0.7 ± 0.01 | 0.7 ± 0.01 | 0.7 ± 0.02 |
|  |  | GBM | 0.67 ± 0.01 | 0.66 ± 0.02 | 0.33 ± 0.03 | 0.66 ± 0.01 | 0.66 ± 0.01 | 0.66 ± 0.01 | 0.66 ± 0.02 | 0.66 ± 0.02 | 0.33 ± 0.03 | 0.66 ± 0.02 | 0.66 ± 0.02 | 0.66 ± 0.02 | 0.67 ± 0.02 |
|  |  | KNN | 0.73 ± 0.01 | 0.75 ± 0.01 | 0.49 ± 0.02 | 0.74 ± 0.01 | 0.74 ± 0.01 | 0.74 ± 0.01 | 0.75 ± 0.02 | 0.75 ± 0.01 | 0.49 ± 0.02 | 0.74 ± 0.01 | 0.74 ± 0.01 | 0.74 ± 0.01 | 0.75 ± 0.02 |
|  |  | LDA | 0.62 ± 0.0 | 0.62 ± 0.01 | 0.24 ± 0.02 | 0.62 ± 0.01 | 0.62 ± 0.01 | 0.62 ± 0.01 | 0.62 ± 0.01 | 0.62 ± 0.01 | 0.24 ± 0.01 | 0.62 ± 0.01 | 0.62 ± 0.01 | 0.62 ± 0.01 | 0.62 ± 0.03 |
|  |  | LR | 0.62 ± 0.0 | 0.62 ± 0.01 | 0.26 ± 0.02 | 0.63 ± 0.01 | 0.63 ± 0.01 | 0.63 ± 0.01 | 0.62 ± 0.01 | 0.62 ± 0.01 | 0.25 ± 0.02 | 0.63 ± 0.01 | 0.63 ± 0.01 | 0.63 ± 0.01 | 0.62 ± 0.02 |
|  |  | NB | 0.65 ± 0.01 | 0.61 ± 0.02 | 0.16 ± 0.03 | 0.57 ± 0.02 | 0.57 ± 0.02 | 0.56 ± 0.02 | 0.69 ± 0.09 | 0.61 ± 0.03 | 0.16 ± 0.03 | 0.57 ± 0.02 | 0.57 ± 0.02 | 0.56 ± 0.02 | 0.69 ± 0.1 |
|  |  | RF | 0.74 ± 0.01 | 0.75 ± 0.01 | 0.5 ± 0.03 | 0.75 ± 0.01 | 0.75 ± 0.01 | 0.76 ± 0.02 | 0.74 ± 0.02 | 0.75 ± 0.01 | 0.5 ± 0.02 | 0.75 ± 0.01 | 0.75 ± 0.01 | 0.76 ± 0.02 | 0.74 ± 0.01 |
|  |  | SVM | 0.73 ± 0.01 | 0.74 ± 0.02 | 0.49 ± 0.03 | 0.74 ± 0.02 | 0.74 ± 0.02 | 0.74 ± 0.02 | 0.74 ± 0.02 | 0.75 ± 0.01 | 0.49 ± 0.03 | 0.75 ± 0.01 | 0.75 ± 0.01 | 0.75 ± 0.02 | 0.75 ± 0.02 |
|  | 95 | AB | 0.68 ± 0.01 | 0.69 ± 0.02 | 0.36 ± 0.03 | 0.68 ± 0.02 | 0.68 ± 0.02 | 0.67 ± 0.02 | 0.7 ± 0.03 | 0.69 ± 0.02 | 0.36 ± 0.04 | 0.68 ± 0.02 | 0.68 ± 0.02 | 0.68 ± 0.02 | 0.7 ± 0.03 |
|  |  | CART | 0.67 ± 0.0 | 0.64 ± 0.02 | 0.28 ± 0.03 | 0.64 ± 0.02 | 0.64 ± 0.02 | 0.64 ± 0.02 | 0.63 ± 0.03 | 0.64 ± 0.02 | 0.28 ± 0.03 | 0.64 ± 0.02 | 0.64 ± 0.02 | 0.64 ± 0.02 | 0.63 ± 0.04 |
|  |  | ET | 0.68 ± 0.0 | 0.7 ± 0.01 | 0.41 ± 0.02 | 0.7 ± 0.01 | 0.7 ± 0.01 | 0.7 ± 0.01 | 0.71 ± 0.02 | 0.7 ± 0.01 | 0.41 ± 0.02 | 0.7 ± 0.01 | 0.7 ± 0.01 | 0.7 ± 0.01 | 0.71 ± 0.02 |
|  |  | GBM | 0.67 ± 0.01 | 0.67 ± 0.01 | 0.34 ± 0.02 | 0.67 ± 0.01 | 0.67 ± 0.01 | 0.67 ± 0.01 | 0.67 ± 0.02 | 0.67 ± 0.01 | 0.34 ± 0.02 | 0.67 ± 0.01 | 0.67 ± 0.01 | 0.67 ± 0.01 | 0.66 ± 0.02 |

|  |  |  |  | **Validation set** | | | | | | **Test set** | | | | | | |
| --- | --- | --- | --- | --- | --- | --- | --- | --- | --- | --- | --- | --- | --- | --- | --- | --- |
| **Activity threshold** | **PCC** | **Algorithm** | **F1 train** | **F1** | **MCC** | **ROC AUC** | **Accuracy** | **Precision** | **Recall** | **F1** | **MCC** | **ROC AUC** | **Accuracy** | **Precision** | **Recall** |  |
|  | 95 | KNN | 0.73 ± 0.01 | 0.75 ± 0.01 | 0.49 ± 0.02 | 0.75 ± 0.01 | 0.75 ± 0.01 | 0.74 ± 0.02 | 0.76 ± 0.01 | 0.75 ± 0.01 | 0.49 ± 0.02 | 0.75 ± 0.01 | 0.75 ± 0.01 | 0.74 ± 0.02 | 0.76 ± 0.01 |  |
|  |  | LDA | 0.63 ± 0.0 | 0.64 ± 0.01 | 0.26 ± 0.02 | 0.63 ± 0.01 | 0.63 ± 0.01 | 0.63 ± 0.01 | 0.64 ± 0.01 | 0.63 ± 0.01 | 0.27 ± 0.02 | 0.63 ± 0.01 | 0.63 ± 0.01 | 0.63 ± 0.01 | 0.64 ± 0.02 |  |
|  |  | LR | 0.64 ± 0.0 | 0.63 ± 0.01 | 0.26 ± 0.02 | 0.63 ± 0.01 | 0.63 ± 0.01 | 0.63 ± 0.01 | 0.64 ± 0.01 | 0.63 ± 0.01 | 0.26 ± 0.01 | 0.63 ± 0.01 | 0.63 ± 0.01 | 0.63 ± 0.01 | 0.64 ± 0.02 |  |
|  |  | NB | 0.64 ± 0.02 | 0.62 ± 0.02 | 0.16 ± 0.03 | 0.57 ± 0.02 | 0.57 ± 0.02 | 0.56 ± 0.02 | 0.69 ± 0.11 | 0.61 ± 0.03 | 0.15 ± 0.03 | 0.57 ± 0.02 | 0.57 ± 0.02 | 0.56 ± 0.02 | 0.69 ± 0.11 |  |
|  |  | RF | 0.74 ± 0.01 | 0.75 ± 0.01 | 0.51 ± 0.02 | 0.75 ± 0.01 | 0.75 ± 0.01 | 0.76 ± 0.01 | 0.74 ± 0.02 | 0.75 ± 0.01 | 0.51 ± 0.03 | 0.75 ± 0.01 | 0.75 ± 0.01 | 0.76 ± 0.02 | 0.74 ± 0.02 |  |
|  |  | SVM | 0.73 ± 0.01 | 0.74 ± 0.01 | 0.48 ± 0.03 | 0.74 ± 0.02 | 0.74 ± 0.02 | 0.73 ± 0.03 | 0.75 ± 0.03 | 0.74 ± 0.01 | 0.48 ± 0.03 | 0.74 ± 0.01 | 0.74 ± 0.01 | 0.73 ± 0.03 | 0.75 ± 0.03 |  |
|  | 100 | AB | 0.67 ± 0.01 | 0.69 ± 0.02 | 0.38 ± 0.03 | 0.69 ± 0.02 | 0.69 ± 0.02 | 0.68 ± 0.02 | 0.71 ± 0.03 | 0.69 ± 0.02 | 0.38 ± 0.03 | 0.69 ± 0.01 | 0.69 ± 0.01 | 0.68 ± 0.02 | 0.71 ± 0.04 |  |
|  |  | CART | 0.66 ± 0.01 | 0.65 ± 0.02 | 0.2 ± 0.1 | 0.59 ± 0.06 | 0.59 ± 0.06 | 0.58 ± 0.07 | 0.78 ± 0.16 | 0.65 ± 0.02 | 0.2 ± 0.1 | 0.59 ± 0.06 | 0.59 ± 0.06 | 0.58 ± 0.07 | 0.78 ± 0.16 |  |
|  |  | ET | 0.68 ± 0.01 | 0.69 ± 0.01 | 0.38 ± 0.02 | 0.69 ± 0.01 | 0.69 ± 0.01 | 0.69 ± 0.01 | 0.7 ± 0.01 | 0.69 ± 0.01 | 0.38 ± 0.02 | 0.69 ± 0.01 | 0.69 ± 0.01 | 0.68 ± 0.01 | 0.7 ± 0.02 |  |
|  |  | GBM | 0.67 ± 0.01 | 0.66 ± 0.01 | 0.33 ± 0.03 | 0.66 ± 0.01 | 0.66 ± 0.01 | 0.66 ± 0.02 | 0.67 ± 0.02 | 0.66 ± 0.01 | 0.33 ± 0.03 | 0.66 ± 0.02 | 0.66 ± 0.02 | 0.66 ± 0.02 | 0.67 ± 0.02 |  |
|  |  | KNN | 0.73 ± 0.01 | 0.74 ± 0.01 | 0.48 ± 0.02 | 0.74 ± 0.01 | 0.74 ± 0.01 | 0.73 ± 0.02 | 0.75 ± 0.02 | 0.74 ± 0.01 | 0.48 ± 0.02 | 0.74 ± 0.01 | 0.74 ± 0.01 | 0.73 ± 0.02 | 0.75 ± 0.02 |  |
|  |  | LDA | 0.64 ± 0.01 | 0.64 ± 0.01 | 0.29 ± 0.02 | 0.64 ± 0.01 | 0.64 ± 0.01 | 0.64 ± 0.01 | 0.65 ± 0.01 | 0.65 ± 0.01 | 0.29 ± 0.02 | 0.65 ± 0.01 | 0.65 ± 0.01 | 0.64 ± 0.01 | 0.65 ± 0.02 |  |
|  |  | LR | 0.64 ± 0.0 | 0.64 ± 0.01 | 0.28 ± 0.02 | 0.64 ± 0.01 | 0.64 ± 0.01 | 0.64 ± 0.01 | 0.64 ± 0.01 | 0.64 ± 0.01 | 0.28 ± 0.02 | 0.64 ± 0.01 | 0.64 ± 0.01 | 0.64 ± 0.01 | 0.64 ± 0.02 |  |
|  |  | NB | 0.64 ± 0.02 | 0.61 ± 0.02 | 0.14 ± 0.02 | 0.57 ± 0.01 | 0.57 ± 0.01 | 0.55 ± 0.01 | 0.7 ± 0.09 | 0.61 ± 0.03 | 0.14 ± 0.02 | 0.57 ± 0.01 | 0.57 ± 0.01 | 0.55 ± 0.01 | 0.7 ± 0.09 |  |
|  |  | RF | 0.74 ± 0.01 | 0.75 ± 0.01 | 0.5 ± 0.02 | 0.75 ± 0.01 | 0.75 ± 0.01 | 0.75 ± 0.02 | 0.74 ± 0.01 | 0.74 ± 0.01 | 0.5 ± 0.03 | 0.75 ± 0.01 | 0.75 ± 0.01 | 0.75 ± 0.02 | 0.74 ± 0.01 |  |
|  |  | SVM | 0.73 ± 0.0 | 0.74 ± 0.01 | 0.46 ± 0.03 | 0.73 ± 0.01 | 0.73 ± 0.01 | 0.72 ± 0.02 | 0.76 ± 0.02 | 0.74 ± 0.01 | 0.46 ± 0.02 | 0.73 ± 0.01 | 0.73 ± 0.01 | 0.72 ± 0.02 | 0.76 ± 0.03 |  |
|  |  |  |  |  |  |  |  |  |  |  |  |  |  |  |  |  |
| FIX 55 | 75 | AB | 0.72 ± 0.0 | 0.72 ± 0.02 | 0.26 ± 0.05 | 0.61 ± 0.03 | 0.64 ± 0.02 | 0.63 ± 0.02 | 0.85 ± 0.07 | 0.72 ± 0.02 | 0.26 ± 0.05 | 0.61 ± 0.03 | 0.63 ± 0.02 | 0.62 ± 0.03 | 0.85 ± 0.07 |  |
|  |  | CART | 0.69 ± 0.01 | 0.69 ± 0.01 | 0.29 ± 0.03 | 0.64 ± 0.01 | 0.65 ± 0.01 | 0.67 ± 0.01 | 0.7 ± 0.01 | 0.69 ± 0.01 | 0.29 ± 0.03 | 0.64 ± 0.01 | 0.65 ± 0.01 | 0.67 ± 0.01 | 0.7 ± 0.01 |  |
|  |  | ET | 0.71 ± 0.0 | 0.74 ± 0.02 | 0.39 ± 0.05 | 0.69 ± 0.02 | 0.7 ± 0.02 | 0.71 ± 0.02 | 0.76 ± 0.02 | 0.74 ± 0.02 | 0.39 ± 0.04 | 0.69 ± 0.02 | 0.7 ± 0.02 | 0.71 ± 0.02 | 0.76 ± 0.02 |  |
|  |  | GBM | 0.72 ± 0.0 | 0.71 ± 0.02 | 0.25 ± 0.13 | 0.62 ± 0.06 | 0.64 ± 0.05 | 0.65 ± 0.05 | 0.79 ± 0.09 | 0.71 ± 0.02 | 0.25 ± 0.12 | 0.62 ± 0.06 | 0.64 ± 0.05 | 0.65 ± 0.05 | 0.79 ± 0.1 |  |

|  |  |  |  | **Validation set** | | | | | | **Test set** | | | | | |
| --- | --- | --- | --- | --- | --- | --- | --- | --- | --- | --- | --- | --- | --- | --- | --- |
| **Activity threshold** | **PCC** | **Algorithm** | **F1 train** | **F1** | **MCC** | **ROC AUC** | **Accuracy** | **Precision** | **Recall** | **F1** | **MCC** | **ROC AUC** | **Accuracy** | **Precision** | **Recall** |
| FIX 55 | 75 | KNN | 0.76 ± 0.01 | 0.76 ± 0.01 | 0.47 ± 0.03 | 0.73 ± 0.01 | 0.74 ± 0.02 | 0.76 ± 0.01 | 0.77 ± 0.02 | 0.76 ± 0.01 | 0.47 ± 0.03 | 0.73 ± 0.01 | 0.74 ± 0.02 | 0.76 ± 0.01 | 0.77 ± 0.02 |
|  |  | LDA | 0.68 ± 0.01 | 0.69 ± 0.01 | 0.22 ± 0.02 | 0.6 ± 0.01 | 0.62 ± 0.01 | 0.63 ± 0.01 | 0.76 ± 0.02 | 0.69 ± 0.01 | 0.22 ± 0.02 | 0.6 ± 0.01 | 0.62 ± 0.01 | 0.63 ± 0.01 | 0.77 ± 0.02 |
|  |  | LR | 0.64 ± 0.01 | 0.69 ± 0.0 | 0.23 ± 0.02 | 0.61 ± 0.01 | 0.62 ± 0.01 | 0.63 ± 0.01 | 0.76 ± 0.02 | 0.69 ± 0.01 | 0.23 ± 0.02 | 0.61 ± 0.01 | 0.63 ± 0.01 | 0.63 ± 0.01 | 0.77 ± 0.02 |
|  |  | NB | 0.68 ± 0.02 | 0.68 ± 0.01 | 0.14 ± 0.07 | 0.56 ± 0.03 | 0.59 ± 0.02 | 0.6 ± 0.02 | 0.82 ± 0.08 | 0.69 ± 0.02 | 0.15 ± 0.08 | 0.57 ± 0.03 | 0.59 ± 0.03 | 0.6 ± 0.02 | 0.82 ± 0.09 |
|  |  | RF | 0.78 ± 0.01 | 0.78 ± 0.01 | 0.5 ± 0.03 | 0.75 ± 0.01 | 0.75 ± 0.01 | 0.76 ± 0.01 | 0.79 ± 0.02 | 0.78 ± 0.01 | 0.49 ± 0.02 | 0.74 ± 0.01 | 0.75 ± 0.01 | 0.76 ± 0.01 | 0.79 ± 0.02 |
|  |  | SVM | 0.77 ± 0.01 | 0.78 ± 0.01 | 0.49 ± 0.02 | 0.74 ± 0.01 | 0.75 ± 0.01 | 0.75 ± 0.01 | 0.81 ± 0.01 | 0.78 ± 0.01 | 0.48 ± 0.02 | 0.74 ± 0.01 | 0.74 ± 0.01 | 0.75 ± 0.01 | 0.81 ± 0.02 |
|  | 85 | AB | 0.72 ± 0.01 | 0.73 ± 0.02 | 0.29 ± 0.06 | 0.62 ± 0.03 | 0.65 ± 0.03 | 0.63 ± 0.02 | 0.87 ± 0.06 | 0.73 ± 0.02 | 0.28 ± 0.06 | 0.62 ± 0.03 | 0.64 ± 0.03 | 0.63 ± 0.02 | 0.87 ± 0.07 |
|  |  | CART | 0.69 ± 0.01 | 0.68 ± 0.02 | 0.26 ± 0.05 | 0.62 ± 0.03 | 0.64 ± 0.02 | 0.66 ± 0.04 | 0.73 ± 0.08 | 0.68 ± 0.02 | 0.26 ± 0.05 | 0.62 ± 0.03 | 0.64 ± 0.02 | 0.66 ± 0.04 | 0.73 ± 0.08 |
|  |  | ET | 0.72 ± 0.01 | 0.74 ± 0.02 | 0.4 ± 0.04 | 0.7 ± 0.02 | 0.7 ± 0.02 | 0.72 ± 0.02 | 0.77 ± 0.02 | 0.74 ± 0.02 | 0.4 ± 0.04 | 0.7 ± 0.02 | 0.7 ± 0.02 | 0.72 ± 0.02 | 0.77 ± 0.02 |
|  |  | GBM | 0.72 ± 0.0 | 0.72 ± 0.02 | 0.33 ± 0.05 | 0.66 ± 0.02 | 0.67 ± 0.02 | 0.68 ± 0.02 | 0.75 ± 0.02 | 0.71 ± 0.02 | 0.32 ± 0.04 | 0.66 ± 0.02 | 0.67 ± 0.02 | 0.68 ± 0.02 | 0.75 ± 0.04 |
|  |  | KNN | 0.76 ± 0.01 | 0.77 ± 0.01 | 0.48 ± 0.03 | 0.74 ± 0.01 | 0.74 ± 0.01 | 0.76 ± 0.01 | 0.78 ± 0.02 | 0.77 ± 0.01 | 0.48 ± 0.03 | 0.74 ± 0.01 | 0.74 ± 0.01 | 0.76 ± 0.01 | 0.78 ± 0.02 |
|  |  | LDA | 0.68 ± 0.01 | 0.69 ± 0.01 | 0.22 ± 0.03 | 0.61 ± 0.01 | 0.62 ± 0.01 | 0.63 ± 0.01 | 0.76 ± 0.01 | 0.69 ± 0.01 | 0.23 ± 0.02 | 0.61 ± 0.01 | 0.62 ± 0.01 | 0.63 ± 0.01 | 0.76 ± 0.02 |
|  |  | LR | 0.64 ± 0.01 | 0.69 ± 0.01 | 0.22 ± 0.03 | 0.61 ± 0.01 | 0.62 ± 0.01 | 0.63 ± 0.01 | 0.76 ± 0.02 | 0.69 ± 0.01 | 0.22 ± 0.02 | 0.6 ± 0.01 | 0.62 ± 0.01 | 0.63 ± 0.01 | 0.76 ± 0.02 |
|  |  | NB | 0.68 ± 0.02 | 0.69 ± 0.01 | 0.15 ± 0.06 | 0.57 ± 0.03 | 0.59 ± 0.02 | 0.6 ± 0.02 | 0.82 ± 0.08 | 0.69 ± 0.02 | 0.16 ± 0.07 | 0.57 ± 0.03 | 0.59 ± 0.03 | 0.6 ± 0.02 | 0.82 ± 0.08 |
|  |  | RF | 0.78 ± 0.01 | 0.78 ± 0.01 | 0.49 ± 0.03 | 0.74 ± 0.01 | 0.75 ± 0.01 | 0.76 ± 0.01 | 0.79 ± 0.01 | 0.78 ± 0.01 | 0.49 ± 0.03 | 0.75 ± 0.01 | 0.75 ± 0.01 | 0.76 ± 0.01 | 0.79 ± 0.02 |
|  |  | SVM | 0.77 ± 0.01 | 0.78 ± 0.01 | 0.48 ± 0.02 | 0.74 ± 0.01 | 0.75 ± 0.01 | 0.74 ± 0.01 | 0.82 ± 0.02 | 0.78 ± 0.01 | 0.48 ± 0.03 | 0.74 ± 0.01 | 0.75 ± 0.01 | 0.74 ± 0.01 | 0.82 ± 0.02 |
|  | 95 | AB | 0.72 ± 0.01 | 0.73 ± 0.02 | 0.3 ± 0.06 | 0.64 ± 0.03 | 0.66 ± 0.03 | 0.65 ± 0.03 | 0.84 ± 0.04 | 0.73 ± 0.02 | 0.31 ± 0.06 | 0.64 ± 0.03 | 0.66 ± 0.03 | 0.65 ± 0.03 | 0.84 ± 0.05 |
|  |  | CART | 0.69 ± 0.0 | 0.69 ± 0.02 | 0.25 ± 0.06 | 0.62 ± 0.03 | 0.63 ± 0.03 | 0.64 ± 0.03 | 0.75 ± 0.06 | 0.69 ± 0.02 | 0.25 ± 0.06 | 0.62 ± 0.03 | 0.63 ± 0.03 | 0.64 ± 0.03 | 0.75 ± 0.06 |
|  |  | ET | 0.72 ± 0.01 | 0.74 ± 0.01 | 0.41 ± 0.03 | 0.7 ± 0.02 | 0.71 ± 0.02 | 0.72 ± 0.01 | 0.78 ± 0.01 | 0.74 ± 0.01 | 0.41 ± 0.03 | 0.7 ± 0.01 | 0.71 ± 0.01 | 0.72 ± 0.01 | 0.78 ± 0.03 |
|  |  | GBM | 0.72 ± 0.0 | 0.71 ± 0.02 | 0.33 ± 0.04 | 0.66 ± 0.02 | 0.67 ± 0.02 | 0.68 ± 0.02 | 0.75 ± 0.01 | 0.71 ± 0.02 | 0.32 ± 0.03 | 0.66 ± 0.02 | 0.67 ± 0.02 | 0.68 ± 0.01 | 0.75 ± 0.04 |
|  |  | KNN | 0.76 ± 0.01 | 0.77 ± 0.01 | 0.48 ± 0.02 | 0.74 ± 0.01 | 0.74 ± 0.01 | 0.76 ± 0.01 | 0.77 ± 0.02 | 0.77 ± 0.01 | 0.48 ± 0.02 | 0.74 ± 0.01 | 0.74 ± 0.01 | 0.76 ± 0.01 | 0.77 ± 0.02 |

|  |  |  |  | **Validation set** | | | | | | **Test set** | | | | | | |
| --- | --- | --- | --- | --- | --- | --- | --- | --- | --- | --- | --- | --- | --- | --- | --- | --- |
| **Activity threshold** | **PCC** | **Algorithm** | **F1 train** | **F1** | **MCC** | **ROC AUC** | **Accuracy** | **Precision** | **Recall** | **F1** | **MCC** | **ROC AUC** | **Accuracy** | **Precision** | **Recall** |  |
| FIX 55 | 95 | LDA | 0.69 ± 0.0 | 0.7 ± 0.0 | 0.26 ± 0.02 | 0.62 ± 0.01 | 0.64 ± 0.01 | 0.64 ± 0.01 | 0.76 ± 0.01 | 0.7 ± 0.01 | 0.26 ± 0.02 | 0.62 ± 0.01 | 0.64 ± 0.01 | 0.64 ± 0.01 | 0.76 ± 0.02 |  |
|  |  | LR | 0.66 ± 0.01 | 0.69 ± 0.0 | 0.25 ± 0.02 | 0.62 ± 0.01 | 0.63 ± 0.01 | 0.64 ± 0.01 | 0.76 ± 0.01 | 0.7 ± 0.01 | 0.25 ± 0.02 | 0.62 ± 0.01 | 0.63 ± 0.01 | 0.64 ± 0.01 | 0.76 ± 0.02 |  |
|  |  | NB | 0.66 ± 0.03 | 0.68 ± 0.02 | 0.15 ± 0.05 | 0.56 ± 0.03 | 0.59 ± 0.02 | 0.6 ± 0.02 | 0.8 ± 0.08 | 0.68 ± 0.02 | 0.14 ± 0.04 | 0.56 ± 0.03 | 0.58 ± 0.02 | 0.59 ± 0.02 | 0.79 ± 0.09 |  |
|  |  | RF | 0.78 ± 0.01 | 0.78 ± 0.01 | 0.49 ± 0.03 | 0.74 ± 0.01 | 0.75 ± 0.01 | 0.76 ± 0.01 | 0.79 ± 0.01 | 0.78 ± 0.01 | 0.49 ± 0.02 | 0.74 ± 0.01 | 0.75 ± 0.01 | 0.76 ± 0.01 | 0.79 ± 0.03 |  |
|  |  | SVM | 0.77 ± 0.01 | 0.78 ± 0.01 | 0.48 ± 0.02 | 0.73 ± 0.01 | 0.74 ± 0.01 | 0.74 ± 0.01 | 0.83 ± 0.02 | 0.78 ± 0.01 | 0.48 ± 0.02 | 0.73 ± 0.01 | 0.74 ± 0.01 | 0.74 ± 0.01 | 0.83 ± 0.02 |  |
|  | 100 | AB | 0.72 ± 0.01 | 0.73 ± 0.02 | 0.31 ± 0.06 | 0.64 ± 0.03 | 0.66 ± 0.03 | 0.65 ± 0.03 | 0.82 ± 0.05 | 0.73 ± 0.02 | 0.31 ± 0.05 | 0.64 ± 0.03 | 0.66 ± 0.03 | 0.65 ± 0.03 | 0.82 ± 0.06 |  |
|  |  | CART | 0.7 ± 0.01 | 0.7 ± 0.01 | 0.1 ± 0.02 | 0.53 ± 0.01 | 0.57 ± 0.01 | 0.57 ± 0.01 | 0.92 ± 0.05 | 0.7 ± 0.01 | 0.1 ± 0.02 | 0.53 ± 0.01 | 0.57 ± 0.01 | 0.57 ± 0.01 | 0.92 ± 0.05 |  |
|  |  | ET | 0.71 ± 0.0 | 0.74 ± 0.01 | 0.38 ± 0.03 | 0.69 ± 0.01 | 0.7 ± 0.01 | 0.7 ± 0.01 | 0.78 ± 0.01 | 0.74 ± 0.01 | 0.38 ± 0.03 | 0.68 ± 0.01 | 0.69 ± 0.01 | 0.7 ± 0.01 | 0.78 ± 0.02 |  |
|  |  | GBM | 0.72 ± 0.01 | 0.71 ± 0.02 | 0.32 ± 0.04 | 0.66 ± 0.02 | 0.67 ± 0.02 | 0.68 ± 0.02 | 0.75 ± 0.02 | 0.71 ± 0.02 | 0.33 ± 0.04 | 0.66 ± 0.02 | 0.67 ± 0.02 | 0.68 ± 0.01 | 0.75 ± 0.04 |  |
|  |  | KNN | 0.76 ± 0.01 | 0.76 ± 0.01 | 0.46 ± 0.03 | 0.73 ± 0.02 | 0.73 ± 0.01 | 0.75 ± 0.01 | 0.77 ± 0.02 | 0.76 ± 0.01 | 0.46 ± 0.03 | 0.73 ± 0.02 | 0.73 ± 0.01 | 0.75 ± 0.01 | 0.77 ± 0.02 |  |
|  |  | LDA | 0.69 ± 0.0 | 0.7 ± 0.01 | 0.28 ± 0.02 | 0.63 ± 0.01 | 0.65 ± 0.01 | 0.65 ± 0.01 | 0.76 ± 0.01 | 0.7 ± 0.01 | 0.28 ± 0.02 | 0.63 ± 0.01 | 0.64 ± 0.01 | 0.65 ± 0.01 | 0.75 ± 0.02 |  |
|  |  | LR | 0.66 ± 0.0 | 0.7 ± 0.01 | 0.26 ± 0.03 | 0.62 ± 0.01 | 0.64 ± 0.01 | 0.65 ± 0.01 | 0.76 ± 0.01 | 0.7 ± 0.01 | 0.26 ± 0.02 | 0.62 ± 0.01 | 0.64 ± 0.01 | 0.65 ± 0.01 | 0.75 ± 0.02 |  |
|  |  | NB | 0.66 ± 0.03 | 0.68 ± 0.02 | 0.14 ± 0.02 | 0.56 ± 0.02 | 0.58 ± 0.01 | 0.59 ± 0.01 | 0.8 ± 0.08 | 0.68 ± 0.02 | 0.14 ± 0.03 | 0.56 ± 0.02 | 0.58 ± 0.01 | 0.59 ± 0.01 | 0.8 ± 0.08 |  |
|  |  | RF | 0.77 ± 0.01 | 0.78 ± 0.01 | 0.49 ± 0.02 | 0.74 ± 0.01 | 0.75 ± 0.01 | 0.76 ± 0.01 | 0.79 ± 0.01 | 0.77 ± 0.01 | 0.48 ± 0.02 | 0.74 ± 0.01 | 0.74 ± 0.01 | 0.76 ± 0.01 | 0.79 ± 0.03 |  |
|  |  | SVM | 0.77 ± 0.01 | 0.78 ± 0.01 | 0.47 ± 0.04 | 0.73 ± 0.02 | 0.74 ± 0.02 | 0.74 ± 0.02 | 0.81 ± 0.02 | 0.77 ± 0.01 | 0.47 ± 0.04 | 0.73 ± 0.02 | 0.74 ± 0.02 | 0.74 ± 0.02 | 0.81 ± 0.02 |  |
|  |  |  |  |  |  |  |  |  |  |  |  |  |  |  |  |  |
| FIX 60 | 75 | AB | 0.75 ± 0.0 | 0.75 ± 0.01 | 0.21 ± 0.06 | 0.58 ± 0.03 | 0.64 ± 0.02 | 0.65 ± 0.02 | 0.88 ± 0.02 | 0.74 ± 0.02 | 0.2 ± 0.06 | 0.58 ± 0.03 | 0.64 ± 0.02 | 0.65 ± 0.02 | 0.88 ± 0.04 |  |
|  |  | CART | 0.73 ± 0.0 | 0.72 ± 0.01 | 0.28 ± 0.02 | 0.64 ± 0.01 | 0.66 ± 0.01 | 0.71 ± 0.01 | 0.73 ± 0.03 | 0.72 ± 0.01 | 0.28 ± 0.02 | 0.64 ± 0.01 | 0.66 ± 0.01 | 0.71 ± 0.01 | 0.73 ± 0.03 |  |
|  |  | ET | 0.74 ± 0.01 | 0.76 ± 0.01 | 0.36 ± 0.03 | 0.67 ± 0.02 | 0.7 ± 0.01 | 0.72 ± 0.01 | 0.81 ± 0.01 | 0.76 ± 0.01 | 0.36 ± 0.02 | 0.67 ± 0.01 | 0.7 ± 0.01 | 0.72 ± 0.01 | 0.81 ± 0.02 |  |
|  |  | GBM | 0.76 ± 0.0 | 0.75 ± 0.01 | 0.27 ± 0.07 | 0.62 ± 0.04 | 0.66 ± 0.03 | 0.68 ± 0.03 | 0.83 ± 0.02 | 0.75 ± 0.01 | 0.26 ± 0.06 | 0.62 ± 0.03 | 0.66 ± 0.02 | 0.68 ± 0.02 | 0.83 ± 0.03 |  |
|  |  | KNN | 0.78 ± 0.0 | 0.79 ± 0.01 | 0.45 ± 0.02 | 0.72 ± 0.01 | 0.74 ± 0.01 | 0.78 ± 0.01 | 0.8 ± 0.02 | 0.79 ± 0.01 | 0.45 ± 0.02 | 0.72 ± 0.01 | 0.74 ± 0.01 | 0.78 ± 0.01 | 0.8 ± 0.02 |  |

|  |  |  |  | **Validation set** | | | | | | **Test set** | | | | | |
| --- | --- | --- | --- | --- | --- | --- | --- | --- | --- | --- | --- | --- | --- | --- | --- |
| **Activity threshold** | **PCC** | **Algorithm** | **F1 train** | **F1** | **MCC** | **ROC AUC** | **Accuracy** | **Precision** | **Recall** | **F1** | **MCC** | **ROC AUC** | **Accuracy** | **Precision** | **Recall** |
| FIX 60 | 75 | LDA | 0.72 ± 0.01 | 0.73 ± 0.01 | 0.17 ± 0.04 | 0.57 ± 0.02 | 0.63 ± 0.02 | 0.64 ± 0.01 | 0.86 ± 0.01 | 0.73 ± 0.01 | 0.17 ± 0.04 | 0.57 ± 0.02 | 0.63 ± 0.01 | 0.64 ± 0.01 | 0.86 ± 0.02 |
|  |  | LR | 0.66 ± 0.01 | 0.73 ± 0.01 | 0.16 ± 0.05 | 0.57 ± 0.02 | 0.63 ± 0.02 | 0.64 ± 0.02 | 0.86 ± 0.02 | 0.73 ± 0.01 | 0.16 ± 0.04 | 0.57 ± 0.02 | 0.62 ± 0.02 | 0.64 ± 0.01 | 0.86 ± 0.02 |
|  |  | NB | 0.73 ± 0.02 | 0.74 ± 0.0 | 0.16 ± 0.06 | 0.56 ± 0.03 | 0.63 ± 0.02 | 0.64 ± 0.02 | 0.9 ± 0.04 | 0.74 ± 0.0 | 0.16 ± 0.05 | 0.56 ± 0.02 | 0.63 ± 0.01 | 0.64 ± 0.02 | 0.9 ± 0.04 |
|  |  | RF | 0.8 ± 0.0 | 0.8 ± 0.01 | 0.46 ± 0.02 | 0.73 ± 0.01 | 0.75 ± 0.01 | 0.77 ± 0.01 | 0.83 ± 0.01 | 0.8 ± 0.01 | 0.47 ± 0.02 | 0.73 ± 0.01 | 0.75 ± 0.01 | 0.77 ± 0.01 | 0.83 ± 0.01 |
|  |  | SVM | 0.79 ± 0.01 | 0.8 ± 0.01 | 0.44 ± 0.03 | 0.71 ± 0.02 | 0.74 ± 0.02 | 0.75 ± 0.02 | 0.85 ± 0.01 | 0.8 ± 0.01 | 0.44 ± 0.03 | 0.71 ± 0.02 | 0.74 ± 0.01 | 0.75 ± 0.02 | 0.85 ± 0.02 |
|  | 85 | AB | 0.75 ± 0.0 | 0.76 ± 0.01 | 0.24 ± 0.07 | 0.59 ± 0.03 | 0.65 ± 0.02 | 0.66 ± 0.02 | 0.89 ± 0.04 | 0.76 ± 0.02 | 0.24 ± 0.07 | 0.59 ± 0.03 | 0.65 ± 0.02 | 0.66 ± 0.02 | 0.89 ± 0.04 |
|  |  | CART | 0.73 ± 0.0 | 0.73 ± 0.01 | 0.27 ± 0.07 | 0.63 ± 0.04 | 0.66 ± 0.02 | 0.7 ± 0.04 | 0.76 ± 0.08 | 0.72 ± 0.01 | 0.27 ± 0.07 | 0.63 ± 0.04 | 0.66 ± 0.02 | 0.7 ± 0.04 | 0.76 ± 0.08 |
|  |  | ET | 0.74 ± 0.01 | 0.77 ± 0.01 | 0.37 ± 0.03 | 0.68 ± 0.01 | 0.71 ± 0.01 | 0.73 ± 0.01 | 0.82 ± 0.01 | 0.77 ± 0.01 | 0.37 ± 0.03 | 0.68 ± 0.01 | 0.7 ± 0.01 | 0.72 ± 0.01 | 0.82 ± 0.02 |
|  |  | GBM | 0.76 ± 0.0 | 0.75 ± 0.01 | 0.28 ± 0.06 | 0.63 ± 0.03 | 0.67 ± 0.02 | 0.69 ± 0.03 | 0.83 ± 0.02 | 0.75 ± 0.02 | 0.28 ± 0.06 | 0.63 ± 0.03 | 0.67 ± 0.02 | 0.69 ± 0.02 | 0.83 ± 0.03 |
|  |  | KNN | 0.79 ± 0.0 | 0.79 ± 0.01 | 0.46 ± 0.02 | 0.72 ± 0.01 | 0.74 ± 0.01 | 0.78 ± 0.01 | 0.8 ± 0.01 | 0.79 ± 0.01 | 0.46 ± 0.02 | 0.72 ± 0.01 | 0.74 ± 0.01 | 0.78 ± 0.01 | 0.8 ± 0.01 |
|  |  | LDA | 0.73 ± 0.0 | 0.74 ± 0.01 | 0.19 ± 0.04 | 0.58 ± 0.02 | 0.64 ± 0.02 | 0.65 ± 0.01 | 0.86 ± 0.01 | 0.74 ± 0.01 | 0.19 ± 0.03 | 0.58 ± 0.01 | 0.64 ± 0.01 | 0.65 ± 0.01 | 0.86 ± 0.03 |
|  |  | LR | 0.67 ± 0.01 | 0.74 ± 0.01 | 0.18 ± 0.04 | 0.57 ± 0.02 | 0.63 ± 0.02 | 0.65 ± 0.02 | 0.86 ± 0.02 | 0.74 ± 0.01 | 0.18 ± 0.03 | 0.57 ± 0.01 | 0.63 ± 0.01 | 0.65 ± 0.01 | 0.86 ± 0.03 |
|  |  | NB | 0.72 ± 0.02 | 0.74 ± 0.0 | 0.18 ± 0.04 | 0.57 ± 0.02 | 0.63 ± 0.01 | 0.64 ± 0.01 | 0.89 ± 0.04 | 0.74 ± 0.01 | 0.17 ± 0.03 | 0.56 ± 0.02 | 0.63 ± 0.01 | 0.64 ± 0.01 | 0.89 ± 0.04 |
|  |  | RF | 0.8 ± 0.0 | 0.8 ± 0.0 | 0.47 ± 0.01 | 0.73 ± 0.01 | 0.75 ± 0.01 | 0.77 ± 0.01 | 0.83 ± 0.01 | 0.8 ± 0.0 | 0.47 ± 0.01 | 0.73 ± 0.01 | 0.75 ± 0.01 | 0.77 ± 0.01 | 0.83 ± 0.01 |
|  |  | SVM | 0.79 ± 0.01 | 0.8 ± 0.01 | 0.44 ± 0.04 | 0.71 ± 0.02 | 0.74 ± 0.02 | 0.74 ± 0.02 | 0.86 ± 0.01 | 0.8 ± 0.01 | 0.44 ± 0.04 | 0.71 ± 0.02 | 0.74 ± 0.02 | 0.74 ± 0.02 | 0.86 ± 0.02 |
|  | 95 | AB | 0.76 ± 0.0 | 0.76 ± 0.02 | 0.24 ± 0.08 | 0.6 ± 0.03 | 0.66 ± 0.03 | 0.66 ± 0.02 | 0.9 ± 0.02 | 0.76 ± 0.02 | 0.24 ± 0.08 | 0.6 ± 0.03 | 0.66 ± 0.03 | 0.66 ± 0.02 | 0.9 ± 0.03 |
|  |  | CART | 0.72 ± 0.0 | 0.72 ± 0.01 | 0.29 ± 0.03 | 0.64 ± 0.02 | 0.66 ± 0.01 | 0.71 ± 0.01 | 0.74 ± 0.02 | 0.72 ± 0.01 | 0.29 ± 0.03 | 0.64 ± 0.02 | 0.66 ± 0.01 | 0.71 ± 0.01 | 0.74 ± 0.02 |
|  |  | ET | 0.74 ± 0.0 | 0.77 ± 0.01 | 0.38 ± 0.02 | 0.68 ± 0.01 | 0.71 ± 0.01 | 0.73 ± 0.01 | 0.82 ± 0.01 | 0.77 ± 0.01 | 0.38 ± 0.02 | 0.68 ± 0.01 | 0.71 ± 0.01 | 0.73 ± 0.01 | 0.82 ± 0.02 |
|  |  | GBM | 0.76 ± 0.0 | 0.74 ± 0.01 | 0.24 ± 0.08 | 0.61 ± 0.04 | 0.65 ± 0.03 | 0.67 ± 0.03 | 0.84 ± 0.04 | 0.74 ± 0.02 | 0.24 ± 0.08 | 0.6 ± 0.04 | 0.65 ± 0.03 | 0.67 ± 0.03 | 0.84 ± 0.04 |
|  |  | KNN | 0.79 ± 0.01 | 0.79 ± 0.01 | 0.45 ± 0.02 | 0.72 ± 0.01 | 0.74 ± 0.01 | 0.77 ± 0.01 | 0.8 ± 0.02 | 0.79 ± 0.01 | 0.45 ± 0.02 | 0.72 ± 0.01 | 0.74 ± 0.01 | 0.77 ± 0.01 | 0.8 ± 0.02 |
|  |  | LDA | 0.73 ± 0.01 | 0.75 ± 0.01 | 0.24 ± 0.03 | 0.6 ± 0.02 | 0.65 ± 0.01 | 0.67 ± 0.01 | 0.85 ± 0.02 | 0.75 ± 0.01 | 0.24 ± 0.03 | 0.61 ± 0.01 | 0.65 ± 0.01 | 0.67 ± 0.01 | 0.85 ± 0.02 |

|  |  |  |  | **Validation set** | | | | | | **Test set** | | | | | |
| --- | --- | --- | --- | --- | --- | --- | --- | --- | --- | --- | --- | --- | --- | --- | --- |
| **Activity threshold** | **PCC** | **Algorithm** | **F1 train** | **F1** | **MCC** | **ROC AUC** | **Accuracy** | **Precision** | **Recall** | **F1** | **MCC** | **ROC AUC** | **Accuracy** | **Precision** | **Recall** |
| FIX 60 | 95 | LR | 0.68 ± 0.01 | 0.74 ± 0.01 | 0.23 ± 0.05 | 0.6 ± 0.02 | 0.65 ± 0.02 | 0.66 ± 0.02 | 0.85 ± 0.02 | 0.74 ± 0.01 | 0.22 ± 0.05 | 0.6 ± 0.02 | 0.64 ± 0.02 | 0.66 ± 0.02 | 0.85 ± 0.03 |
|  |  | NB | 0.7 ± 0.04 | 0.74 ± 0.01 | 0.16 ± 0.03 | 0.56 ± 0.02 | 0.62 ± 0.01 | 0.64 ± 0.01 | 0.88 ± 0.05 | 0.74 ± 0.01 | 0.16 ± 0.03 | 0.56 ± 0.02 | 0.62 ± 0.01 | 0.64 ± 0.01 | 0.88 ± 0.05 |
|  |  | RF | 0.81 ± 0.01 | 0.8 ± 0.0 | 0.47 ± 0.01 | 0.73 ± 0.01 | 0.75 ± 0.01 | 0.77 ± 0.0 | 0.83 ± 0.01 | 0.8 ± 0.0 | 0.47 ± 0.01 | 0.73 ± 0.01 | 0.75 ± 0.0 | 0.77 ± 0.01 | 0.83 ± 0.01 |
|  |  | SVM | 0.79 ± 0.01 | 0.8 ± 0.01 | 0.44 ± 0.04 | 0.71 ± 0.02 | 0.74 ± 0.02 | 0.75 ± 0.02 | 0.86 ± 0.02 | 0.8 ± 0.01 | 0.44 ± 0.04 | 0.71 ± 0.02 | 0.74 ± 0.02 | 0.75 ± 0.02 | 0.86 ± 0.02 |
|  | 100 | AB | 0.76 ± 0.0 | 0.75 ± 0.01 | 0.23 ± 0.06 | 0.6 ± 0.03 | 0.65 ± 0.02 | 0.66 ± 0.02 | 0.87 ± 0.02 | 0.75 ± 0.02 | 0.23 ± 0.06 | 0.6 ± 0.03 | 0.65 ± 0.02 | 0.66 ± 0.02 | 0.87 ± 0.03 |
|  |  | CART | 0.73 ± 0.01 | 0.74 ± 0.01 | 0.16 ± 0.06 | 0.56 ± 0.04 | 0.62 ± 0.02 | 0.64 ± 0.04 | 0.89 ± 0.09 | 0.74 ± 0.01 | 0.14 ± 0.08 | 0.55 ± 0.04 | 0.62 ± 0.02 | 0.64 ± 0.04 | 0.89 ± 0.09 |
|  |  | ET | 0.73 ± 0.01 | 0.77 ± 0.01 | 0.35 ± 0.02 | 0.66 ± 0.01 | 0.7 ± 0.01 | 0.71 ± 0.01 | 0.83 ± 0.01 | 0.77 ± 0.01 | 0.35 ± 0.02 | 0.66 ± 0.01 | 0.7 ± 0.01 | 0.71 ± 0.01 | 0.83 ± 0.02 |
|  |  | GBM | 0.76 ± 0.0 | 0.75 ± 0.01 | 0.26 ± 0.06 | 0.62 ± 0.03 | 0.66 ± 0.02 | 0.68 ± 0.02 | 0.84 ± 0.03 | 0.75 ± 0.01 | 0.27 ± 0.06 | 0.62 ± 0.03 | 0.66 ± 0.02 | 0.68 ± 0.02 | 0.84 ± 0.03 |
|  |  | KNN | 0.78 ± 0.0 | 0.78 ± 0.01 | 0.44 ± 0.02 | 0.72 ± 0.01 | 0.74 ± 0.01 | 0.77 ± 0.01 | 0.79 ± 0.02 | 0.78 ± 0.01 | 0.44 ± 0.02 | 0.72 ± 0.01 | 0.74 ± 0.01 | 0.77 ± 0.01 | 0.79 ± 0.02 |
|  |  | LDA | 0.74 ± 0.0 | 0.75 ± 0.01 | 0.25 ± 0.02 | 0.61 ± 0.01 | 0.66 ± 0.01 | 0.67 ± 0.01 | 0.85 ± 0.02 | 0.75 ± 0.01 | 0.25 ± 0.02 | 0.61 ± 0.01 | 0.66 ± 0.01 | 0.67 ± 0.01 | 0.85 ± 0.01 |
|  |  | LR | 0.69 ± 0.01 | 0.75 ± 0.01 | 0.24 ± 0.01 | 0.6 ± 0.01 | 0.65 ± 0.0 | 0.66 ± 0.01 | 0.85 ± 0.02 | 0.75 ± 0.0 | 0.24 ± 0.02 | 0.6 ± 0.01 | 0.65 ± 0.0 | 0.66 ± 0.01 | 0.85 ± 0.02 |
|  |  | NB | 0.69 ± 0.04 | 0.74 ± 0.01 | 0.11 ± 0.08 | 0.54 ± 0.03 | 0.62 ± 0.01 | 0.63 ± 0.02 | 0.91 ± 0.07 | 0.74 ± 0.01 | 0.11 ± 0.08 | 0.54 ± 0.03 | 0.62 ± 0.01 | 0.63 ± 0.02 | 0.91 ± 0.07 |
|  |  | RF | 0.8 ± 0.0 | 0.8 ± 0.01 | 0.46 ± 0.02 | 0.72 ± 0.01 | 0.74 ± 0.01 | 0.76 ± 0.01 | 0.83 ± 0.01 | 0.8 ± 0.01 | 0.46 ± 0.02 | 0.72 ± 0.01 | 0.74 ± 0.01 | 0.76 ± 0.01 | 0.83 ± 0.01 |
|  |  | SVM | 0.79 ± 0.01 | 0.8 ± 0.01 | 0.44 ± 0.04 | 0.71 ± 0.02 | 0.74 ± 0.02 | 0.75 ± 0.02 | 0.85 ± 0.02 | 0.8 ± 0.01 | 0.44 ± 0.04 | 0.71 ± 0.02 | 0.74 ± 0.02 | 0.75 ± 0.02 | 0.85 ± 0.03 |

**Table S3.** Results of the machine learning models developed on data related to the DU-145 cell line.

|  |  |  |  | **Validation set** | | | | | | **Test set** | | | | | |
| --- | --- | --- | --- | --- | --- | --- | --- | --- | --- | --- | --- | --- | --- | --- | --- |
| **Activity threshold** | **PCC** | **Algorithm** | **F1 train** | **F1** | **MCC** | **ROC AUC** | **Accuracy** | **Precision** | **Recall** | **F1** | **MCC** | **ROC AUC** | **Accuracy** | **Precision** | **Recall** |
| GAP 5 | 75 | AB | 0.71 ± 0.02 | 0.72 ± 0.02 | 0.43 ± 0.04 | 0.71 ± 0.02 | 0.71 ± 0.02 | 0.7 ± 0.02 | 0.74 ± 0.04 | 0.72 ± 0.03 | 0.43 ± 0.04 | 0.71 ± 0.02 | 0.71 ± 0.02 | 0.7 ± 0.02 | 0.74 ± 0.06 |
|  |  | CART | 0.7 ± 0.01 | 0.68 ± 0.04 | 0.37 ± 0.07 | 0.68 ± 0.03 | 0.68 ± 0.03 | 0.68 ± 0.03 | 0.68 ± 0.06 | 0.68 ± 0.04 | 0.37 ± 0.07 | 0.68 ± 0.03 | 0.68 ± 0.03 | 0.68 ± 0.03 | 0.68 ± 0.07 |
|  |  | ET | 0.71 ± 0.02 | 0.72 ± 0.04 | 0.43 ± 0.08 | 0.72 ± 0.04 | 0.72 ± 0.04 | 0.71 ± 0.04 | 0.73 ± 0.04 | 0.72 ± 0.04 | 0.43 ± 0.08 | 0.72 ± 0.04 | 0.72 ± 0.04 | 0.71 ± 0.03 | 0.72 ± 0.06 |
|  |  | GBM | 0.73 ± 0.01 | 0.72 ± 0.04 | 0.45 ± 0.06 | 0.73 ± 0.03 | 0.73 ± 0.03 | 0.72 ± 0.03 | 0.72 ± 0.04 | 0.72 ± 0.04 | 0.44 ± 0.06 | 0.72 ± 0.03 | 0.72 ± 0.03 | 0.72 ± 0.03 | 0.72 ± 0.05 |
|  |  | KNN | 0.75 ± 0.01 | 0.75 ± 0.03 | 0.49 ± 0.06 | 0.75 ± 0.03 | 0.75 ± 0.03 | 0.74 ± 0.03 | 0.75 ± 0.04 | 0.75 ± 0.03 | 0.49 ± 0.06 | 0.75 ± 0.03 | 0.75 ± 0.03 | 0.74 ± 0.03 | 0.75 ± 0.04 |
|  |  | LDA | 0.66 ± 0.02 | 0.66 ± 0.03 | 0.32 ± 0.05 | 0.66 ± 0.02 | 0.66 ± 0.02 | 0.66 ± 0.02 | 0.67 ± 0.04 | 0.66 ± 0.03 | 0.33 ± 0.06 | 0.66 ± 0.03 | 0.66 ± 0.03 | 0.66 ± 0.03 | 0.67 ± 0.04 |
|  |  | LR | 0.67 ± 0.02 | 0.66 ± 0.03 | 0.31 ± 0.06 | 0.65 ± 0.03 | 0.65 ± 0.03 | 0.64 ± 0.03 | 0.68 ± 0.04 | 0.66 ± 0.03 | 0.31 ± 0.06 | 0.66 ± 0.03 | 0.66 ± 0.03 | 0.65 ± 0.03 | 0.68 ± 0.05 |
|  |  | NB | 0.68 ± 0.01 | 0.66 ± 0.03 | 0.23 ± 0.07 | 0.6 ± 0.04 | 0.6 ± 0.04 | 0.58 ± 0.04 | 0.77 ± 0.07 | 0.66 ± 0.03 | 0.23 ± 0.06 | 0.6 ± 0.04 | 0.6 ± 0.04 | 0.58 ± 0.03 | 0.77 ± 0.08 |
|  |  | RF | 0.77 ± 0.02 | 0.77 ± 0.03 | 0.53 ± 0.05 | 0.77 ± 0.03 | 0.77 ± 0.03 | 0.76 ± 0.02 | 0.77 ± 0.04 | 0.76 ± 0.03 | 0.53 ± 0.04 | 0.77 ± 0.02 | 0.77 ± 0.02 | 0.76 ± 0.03 | 0.77 ± 0.04 |
|  |  | SVM | 0.76 ± 0.02 | 0.75 ± 0.03 | 0.49 ± 0.06 | 0.75 ± 0.03 | 0.75 ± 0.03 | 0.73 ± 0.03 | 0.78 ± 0.03 | 0.75 ± 0.02 | 0.49 ± 0.05 | 0.74 ± 0.02 | 0.74 ± 0.02 | 0.73 ± 0.03 | 0.78 ± 0.04 |
|  | 85 | AB | 0.71 ± 0.02 | 0.72 ± 0.03 | 0.44 ± 0.06 | 0.72 ± 0.03 | 0.72 ± 0.03 | 0.7 ± 0.03 | 0.75 ± 0.04 | 0.72 ± 0.03 | 0.43 ± 0.06 | 0.72 ± 0.03 | 0.72 ± 0.03 | 0.7 ± 0.03 | 0.74 ± 0.05 |
|  |  | CART | 0.71 ± 0.01 | 0.67 ± 0.03 | 0.32 ± 0.08 | 0.66 ± 0.05 | 0.65 ± 0.05 | 0.65 ± 0.06 | 0.72 ± 0.12 | 0.67 ± 0.03 | 0.32 ± 0.07 | 0.65 ± 0.05 | 0.65 ± 0.05 | 0.65 ± 0.06 | 0.72 ± 0.12 |
|  |  | ET | 0.71 ± 0.01 | 0.7 ± 0.04 | 0.41 ± 0.07 | 0.7 ± 0.04 | 0.7 ± 0.03 | 0.7 ± 0.03 | 0.71 ± 0.05 | 0.7 ± 0.04 | 0.41 ± 0.07 | 0.7 ± 0.03 | 0.7 ± 0.03 | 0.7 ± 0.03 | 0.71 ± 0.06 |
|  |  | GBM | 0.74 ± 0.01 | 0.72 ± 0.03 | 0.45 ± 0.06 | 0.72 ± 0.03 | 0.72 ± 0.03 | 0.72 ± 0.03 | 0.72 ± 0.04 | 0.72 ± 0.04 | 0.45 ± 0.06 | 0.72 ± 0.03 | 0.72 ± 0.03 | 0.73 ± 0.04 | 0.72 ± 0.05 |
|  |  | KNN | 0.75 ± 0.01 | 0.75 ± 0.02 | 0.49 ± 0.05 | 0.75 ± 0.02 | 0.75 ± 0.02 | 0.74 ± 0.02 | 0.75 ± 0.03 | 0.75 ± 0.02 | 0.49 ± 0.05 | 0.75 ± 0.02 | 0.75 ± 0.02 | 0.74 ± 0.02 | 0.75 ± 0.03 |
|  |  | LDA | 0.66 ± 0.01 | 0.68 ± 0.02 | 0.35 ± 0.05 | 0.67 ± 0.02 | 0.67 ± 0.02 | 0.67 ± 0.02 | 0.68 ± 0.03 | 0.67 ± 0.02 | 0.34 ± 0.05 | 0.67 ± 0.02 | 0.67 ± 0.02 | 0.66 ± 0.02 | 0.68 ± 0.03 |
|  |  | LR | 0.67 ± 0.02 | 0.66 ± 0.02 | 0.32 ± 0.04 | 0.66 ± 0.02 | 0.66 ± 0.02 | 0.65 ± 0.02 | 0.68 ± 0.03 | 0.66 ± 0.03 | 0.31 ± 0.05 | 0.66 ± 0.02 | 0.66 ± 0.02 | 0.65 ± 0.02 | 0.68 ± 0.04 |
|  |  | NB | 0.68 ± 0.01 | 0.66 ± 0.04 | 0.2 ± 0.08 | 0.59 ± 0.04 | 0.59 ± 0.04 | 0.56 ± 0.04 | 0.8 ± 0.09 | 0.66 ± 0.04 | 0.21 ± 0.08 | 0.59 ± 0.04 | 0.59 ± 0.04 | 0.56 ± 0.04 | 0.81 ± 0.1 |
|  |  | RF | 0.77 ± 0.02 | 0.77 ± 0.03 | 0.54 ± 0.06 | 0.77 ± 0.03 | 0.77 ± 0.03 | 0.77 ± 0.03 | 0.77 ± 0.03 | 0.77 ± 0.03 | 0.54 ± 0.06 | 0.77 ± 0.03 | 0.77 ± 0.03 | 0.77 ± 0.04 | 0.76 ± 0.04 |
|  |  | SVM | 0.76 ± 0.01 | 0.75 ± 0.02 | 0.49 ± 0.05 | 0.75 ± 0.03 | 0.74 ± 0.03 | 0.72 ± 0.02 | 0.79 ± 0.03 | 0.75 ± 0.02 | 0.49 ± 0.05 | 0.74 ± 0.02 | 0.74 ± 0.02 | 0.72 ± 0.02 | 0.79 ± 0.04 |
|  | 95 | AB | 0.71 ± 0.02 | 0.72 ± 0.03 | 0.42 ± 0.05 | 0.71 ± 0.02 | 0.71 ± 0.02 | 0.7 ± 0.02 | 0.73 ± 0.04 | 0.71 ± 0.03 | 0.41 ± 0.05 | 0.71 ± 0.03 | 0.71 ± 0.03 | 0.7 ± 0.02 | 0.73 ± 0.06 |

|  |  |  |  | **Validation set** | | | | | | **Test set** | | | | | |
| --- | --- | --- | --- | --- | --- | --- | --- | --- | --- | --- | --- | --- | --- | --- | --- |
| **Activity threshold** | **PCC** | **Algorithm** | **F1 train** | **F1** | **MCC** | **ROC AUC** | **Accuracy** | **Precision** | **Recall** | **F1** | **MCC** | **ROC AUC** | **Accuracy** | **Precision** | **Recall** |
| GAP 5 | 95 | CART | 0.71 ± 0.02 | 0.68 ± 0.03 | 0.35 ± 0.06 | 0.68 ± 0.03 | 0.68 ± 0.03 | 0.67 ± 0.04 | 0.7 ± 0.04 | 0.68 ± 0.02 | 0.35 ± 0.05 | 0.68 ± 0.02 | 0.68 ± 0.02 | 0.67 ± 0.04 | 0.7 ± 0.04 |
|  |  | ET | 0.71 ± 0.01 | 0.71 ± 0.04 | 0.42 ± 0.08 | 0.71 ± 0.04 | 0.71 ± 0.04 | 0.71 ± 0.03 | 0.71 ± 0.05 | 0.7 ± 0.04 | 0.41 ± 0.07 | 0.7 ± 0.04 | 0.7 ± 0.04 | 0.7 ± 0.03 | 0.7 ± 0.06 |
|  |  | GBM | 0.74 ± 0.01 | 0.72 ± 0.03 | 0.44 ± 0.06 | 0.72 ± 0.03 | 0.72 ± 0.03 | 0.71 ± 0.02 | 0.72 ± 0.04 | 0.71 ± 0.03 | 0.43 ± 0.05 | 0.71 ± 0.03 | 0.71 ± 0.03 | 0.71 ± 0.04 | 0.71 ± 0.05 |
|  |  | KNN | 0.75 ± 0.01 | 0.75 ± 0.03 | 0.5 ± 0.05 | 0.75 ± 0.03 | 0.75 ± 0.03 | 0.74 ± 0.03 | 0.76 ± 0.04 | 0.75 ± 0.03 | 0.5 ± 0.05 | 0.75 ± 0.03 | 0.75 ± 0.03 | 0.74 ± 0.03 | 0.76 ± 0.04 |
|  |  | LDA | 0.68 ± 0.01 | 0.69 ± 0.03 | 0.38 ± 0.05 | 0.69 ± 0.03 | 0.69 ± 0.03 | 0.68 ± 0.03 | 0.7 ± 0.03 | 0.7 ± 0.03 | 0.39 ± 0.06 | 0.69 ± 0.03 | 0.69 ± 0.03 | 0.69 ± 0.03 | 0.7 ± 0.04 |
|  |  | LR | 0.68 ± 0.01 | 0.68 ± 0.02 | 0.36 ± 0.05 | 0.68 ± 0.03 | 0.68 ± 0.03 | 0.67 ± 0.02 | 0.7 ± 0.03 | 0.68 ± 0.03 | 0.35 ± 0.05 | 0.68 ± 0.03 | 0.68 ± 0.03 | 0.67 ± 0.03 | 0.69 ± 0.04 |
|  |  | NB | 0.68 ± 0.01 | 0.66 ± 0.03 | 0.23 ± 0.08 | 0.61 ± 0.04 | 0.61 ± 0.04 | 0.58 ± 0.03 | 0.76 ± 0.04 | 0.66 ± 0.04 | 0.24 ± 0.08 | 0.61 ± 0.04 | 0.61 ± 0.04 | 0.58 ± 0.03 | 0.77 ± 0.06 |
|  |  | RF | 0.77 ± 0.02 | 0.77 ± 0.02 | 0.54 ± 0.05 | 0.77 ± 0.02 | 0.77 ± 0.02 | 0.76 ± 0.02 | 0.77 ± 0.03 | 0.77 ± 0.02 | 0.54 ± 0.04 | 0.77 ± 0.02 | 0.77 ± 0.02 | 0.77 ± 0.03 | 0.77 ± 0.04 |
|  |  | SVM | 0.76 ± 0.02 | 0.75 ± 0.03 | 0.49 ± 0.07 | 0.74 ± 0.03 | 0.74 ± 0.03 | 0.72 ± 0.03 | 0.8 ± 0.04 | 0.75 ± 0.03 | 0.48 ± 0.06 | 0.74 ± 0.03 | 0.74 ± 0.03 | 0.72 ± 0.03 | 0.8 ± 0.06 |
|  | 100 | AB | 0.72 ± 0.02 | 0.71 ± 0.03 | 0.42 ± 0.05 | 0.71 ± 0.03 | 0.71 ± 0.03 | 0.7 ± 0.02 | 0.73 ± 0.04 | 0.71 ± 0.03 | 0.42 ± 0.05 | 0.71 ± 0.03 | 0.71 ± 0.03 | 0.7 ± 0.03 | 0.72 ± 0.06 |
|  |  | CART | 0.7 ± 0.02 | 0.68 ± 0.02 | 0.36 ± 0.04 | 0.68 ± 0.02 | 0.68 ± 0.02 | 0.68 ± 0.02 | 0.67 ± 0.04 | 0.68 ± 0.03 | 0.36 ± 0.04 | 0.68 ± 0.02 | 0.68 ± 0.02 | 0.68 ± 0.02 | 0.67 ± 0.05 |
|  |  | ET | 0.71 ± 0.01 | 0.72 ± 0.03 | 0.43 ± 0.06 | 0.72 ± 0.03 | 0.72 ± 0.03 | 0.71 ± 0.03 | 0.72 ± 0.04 | 0.71 ± 0.04 | 0.43 ± 0.08 | 0.71 ± 0.04 | 0.71 ± 0.04 | 0.71 ± 0.04 | 0.72 ± 0.06 |
|  |  | GBM | 0.74 ± 0.02 | 0.73 ± 0.03 | 0.46 ± 0.05 | 0.73 ± 0.03 | 0.73 ± 0.03 | 0.73 ± 0.02 | 0.73 ± 0.04 | 0.72 ± 0.03 | 0.45 ± 0.05 | 0.73 ± 0.03 | 0.73 ± 0.03 | 0.73 ± 0.04 | 0.72 ± 0.05 |
|  |  | KNN | 0.75 ± 0.01 | 0.75 ± 0.02 | 0.5 ± 0.04 | 0.75 ± 0.02 | 0.75 ± 0.02 | 0.74 ± 0.03 | 0.75 ± 0.02 | 0.75 ± 0.02 | 0.5 ± 0.04 | 0.75 ± 0.02 | 0.75 ± 0.02 | 0.74 ± 0.03 | 0.75 ± 0.02 |
|  |  | LDA | 0.68 ± 0.02 | 0.69 ± 0.02 | 0.38 ± 0.04 | 0.69 ± 0.02 | 0.69 ± 0.02 | 0.68 ± 0.02 | 0.7 ± 0.03 | 0.69 ± 0.02 | 0.37 ± 0.04 | 0.69 ± 0.02 | 0.69 ± 0.02 | 0.68 ± 0.02 | 0.7 ± 0.04 |
|  |  | LR | 0.68 ± 0.02 | 0.69 ± 0.02 | 0.38 ± 0.05 | 0.69 ± 0.02 | 0.69 ± 0.02 | 0.68 ± 0.02 | 0.7 ± 0.03 | 0.69 ± 0.03 | 0.37 ± 0.05 | 0.68 ± 0.02 | 0.68 ± 0.02 | 0.68 ± 0.03 | 0.7 ± 0.04 |
|  |  | NB | 0.67 ± 0.01 | 0.65 ± 0.04 | 0.2 ± 0.07 | 0.59 ± 0.04 | 0.59 ± 0.04 | 0.56 ± 0.03 | 0.78 ± 0.07 | 0.65 ± 0.04 | 0.2 ± 0.07 | 0.59 ± 0.03 | 0.59 ± 0.03 | 0.56 ± 0.02 | 0.78 ± 0.08 |
|  |  | RF | 0.77 ± 0.01 | 0.76 ± 0.02 | 0.53 ± 0.04 | 0.76 ± 0.02 | 0.76 ± 0.02 | 0.76 ± 0.02 | 0.77 ± 0.03 | 0.76 ± 0.02 | 0.53 ± 0.04 | 0.76 ± 0.02 | 0.76 ± 0.02 | 0.76 ± 0.03 | 0.77 ± 0.03 |
|  |  | SVM | 0.75 ± 0.01 | 0.76 ± 0.03 | 0.49 ± 0.06 | 0.74 ± 0.03 | 0.74 ± 0.03 | 0.72 ± 0.03 | 0.8 ± 0.03 | 0.75 ± 0.03 | 0.48 ± 0.06 | 0.74 ± 0.03 | 0.74 ± 0.03 | 0.72 ± 0.03 | 0.8 ± 0.04 |
|  |  |  |  |  |  |  |  |  |  |  |  |  |  |  |  |
| GAP 10 | 75 | AB | 0.72 ± 0.01 | 0.74 ± 0.02 | 0.46 ± 0.04 | 0.73 ± 0.02 | 0.73 ± 0.02 | 0.72 ± 0.03 | 0.76 ± 0.03 | 0.73 ± 0.02 | 0.46 ± 0.04 | 0.73 ± 0.02 | 0.73 ± 0.02 | 0.72 ± 0.03 | 0.75 ± 0.05 |

|  |  |  |  | **Validation set** | | | | | | **Test set** | | | | | |
| --- | --- | --- | --- | --- | --- | --- | --- | --- | --- | --- | --- | --- | --- | --- | --- |
| **Activity threshold** | **PCC** | **Algorithm** | **F1 train** | **F1** | **MCC** | **ROC AUC** | **Accuracy** | **Precision** | **Recall** | **F1** | **MCC** | **ROC AUC** | **Accuracy** | **Precision** | **Recall** |
| GAP 10 | 75 | CART | 0.72 ± 0.02 | 0.69 ± 0.03 | 0.38 ± 0.06 | 0.69 ± 0.03 | 0.69 ± 0.03 | 0.69 ± 0.03 | 0.69 ± 0.04 | 0.68 ± 0.03 | 0.38 ± 0.05 | 0.69 ± 0.03 | 0.69 ± 0.03 | 0.69 ± 0.03 | 0.68 ± 0.04 |
|  |  | ET | 0.7 ± 0.01 | 0.72 ± 0.02 | 0.44 ± 0.04 | 0.72 ± 0.02 | 0.72 ± 0.02 | 0.71 ± 0.02 | 0.73 ± 0.02 | 0.72 ± 0.02 | 0.44 ± 0.04 | 0.72 ± 0.02 | 0.72 ± 0.02 | 0.72 ± 0.03 | 0.73 ± 0.03 |
|  |  | GBM | 0.74 ± 0.01 | 0.74 ± 0.02 | 0.48 ± 0.03 | 0.74 ± 0.02 | 0.74 ± 0.02 | 0.73 ± 0.02 | 0.75 ± 0.02 | 0.74 ± 0.02 | 0.48 ± 0.03 | 0.74 ± 0.01 | 0.74 ± 0.01 | 0.73 ± 0.02 | 0.75 ± 0.04 |
|  |  | KNN | 0.76 ± 0.02 | 0.77 ± 0.02 | 0.54 ± 0.04 | 0.77 ± 0.02 | 0.77 ± 0.02 | 0.76 ± 0.02 | 0.79 ± 0.04 | 0.77 ± 0.02 | 0.54 ± 0.04 | 0.77 ± 0.02 | 0.77 ± 0.02 | 0.76 ± 0.02 | 0.79 ± 0.04 |
|  |  | LDA | 0.69 ± 0.01 | 0.71 ± 0.02 | 0.41 ± 0.03 | 0.7 ± 0.02 | 0.7 ± 0.02 | 0.7 ± 0.02 | 0.72 ± 0.03 | 0.7 ± 0.02 | 0.4 ± 0.04 | 0.7 ± 0.02 | 0.7 ± 0.02 | 0.69 ± 0.02 | 0.72 ± 0.02 |
|  |  | LR | 0.7 ± 0.01 | 0.7 ± 0.01 | 0.39 ± 0.02 | 0.69 ± 0.01 | 0.7 ± 0.01 | 0.68 ± 0.01 | 0.71 ± 0.02 | 0.69 ± 0.02 | 0.38 ± 0.03 | 0.69 ± 0.02 | 0.69 ± 0.02 | 0.68 ± 0.02 | 0.71 ± 0.03 |
|  |  | NB | 0.67 ± 0.01 | 0.66 ± 0.02 | 0.22 ± 0.04 | 0.6 ± 0.02 | 0.6 ± 0.02 | 0.57 ± 0.01 | 0.79 ± 0.05 | 0.66 ± 0.02 | 0.22 ± 0.03 | 0.6 ± 0.02 | 0.6 ± 0.02 | 0.57 ± 0.01 | 0.79 ± 0.05 |
|  |  | RF | 0.78 ± 0.01 | 0.78 ± 0.02 | 0.56 ± 0.03 | 0.78 ± 0.01 | 0.78 ± 0.01 | 0.77 ± 0.01 | 0.79 ± 0.03 | 0.78 ± 0.02 | 0.55 ± 0.03 | 0.78 ± 0.01 | 0.78 ± 0.01 | 0.77 ± 0.02 | 0.79 ± 0.04 |
|  |  | SVM | 0.76 ± 0.0 | 0.77 ± 0.02 | 0.52 ± 0.04 | 0.76 ± 0.02 | 0.76 ± 0.02 | 0.73 ± 0.02 | 0.81 ± 0.02 | 0.77 ± 0.02 | 0.52 ± 0.04 | 0.76 ± 0.02 | 0.76 ± 0.02 | 0.73 ± 0.02 | 0.82 ± 0.03 |
|  | 85 | AB | 0.72 ± 0.01 | 0.72 ± 0.02 | 0.44 ± 0.03 | 0.72 ± 0.01 | 0.72 ± 0.01 | 0.71 ± 0.02 | 0.73 ± 0.04 | 0.72 ± 0.03 | 0.44 ± 0.04 | 0.72 ± 0.02 | 0.72 ± 0.02 | 0.71 ± 0.02 | 0.73 ± 0.07 |
|  |  | CART | 0.71 ± 0.01 | 0.67 ± 0.02 | 0.33 ± 0.04 | 0.67 ± 0.02 | 0.67 ± 0.02 | 0.66 ± 0.02 | 0.68 ± 0.04 | 0.67 ± 0.02 | 0.33 ± 0.04 | 0.66 ± 0.02 | 0.66 ± 0.02 | 0.66 ± 0.03 | 0.68 ± 0.04 |
|  |  | ET | 0.71 ± 0.01 | 0.72 ± 0.02 | 0.44 ± 0.04 | 0.72 ± 0.02 | 0.72 ± 0.02 | 0.71 ± 0.02 | 0.73 ± 0.02 | 0.72 ± 0.02 | 0.44 ± 0.04 | 0.72 ± 0.02 | 0.72 ± 0.02 | 0.72 ± 0.03 | 0.73 ± 0.03 |
|  |  | GBM | 0.73 ± 0.01 | 0.73 ± 0.01 | 0.45 ± 0.03 | 0.73 ± 0.01 | 0.73 ± 0.01 | 0.72 ± 0.01 | 0.73 ± 0.02 | 0.72 ± 0.02 | 0.45 ± 0.03 | 0.72 ± 0.01 | 0.72 ± 0.01 | 0.72 ± 0.02 | 0.73 ± 0.04 |
|  |  | KNN | 0.77 ± 0.01 | 0.77 ± 0.02 | 0.54 ± 0.05 | 0.77 ± 0.02 | 0.77 ± 0.02 | 0.76 ± 0.03 | 0.79 ± 0.03 | 0.77 ± 0.02 | 0.54 ± 0.05 | 0.77 ± 0.02 | 0.77 ± 0.02 | 0.76 ± 0.03 | 0.79 ± 0.03 |
|  |  | LDA | 0.66 ± 0.02 | 0.68 ± 0.02 | 0.35 ± 0.04 | 0.68 ± 0.02 | 0.68 ± 0.02 | 0.67 ± 0.02 | 0.69 ± 0.02 | 0.68 ± 0.02 | 0.35 ± 0.04 | 0.68 ± 0.02 | 0.68 ± 0.02 | 0.67 ± 0.03 | 0.69 ± 0.03 |
|  |  | LR | 0.68 ± 0.02 | 0.67 ± 0.02 | 0.33 ± 0.04 | 0.66 ± 0.02 | 0.66 ± 0.02 | 0.66 ± 0.02 | 0.68 ± 0.03 | 0.67 ± 0.02 | 0.33 ± 0.03 | 0.66 ± 0.02 | 0.66 ± 0.02 | 0.66 ± 0.02 | 0.68 ± 0.05 |
|  |  | NB | 0.67 ± 0.02 | 0.67 ± 0.02 | 0.25 ± 0.06 | 0.62 ± 0.04 | 0.62 ± 0.04 | 0.59 ± 0.03 | 0.78 ± 0.08 | 0.67 ± 0.02 | 0.26 ± 0.06 | 0.62 ± 0.04 | 0.62 ± 0.04 | 0.59 ± 0.04 | 0.78 ± 0.09 |
|  |  | RF | 0.78 ± 0.01 | 0.78 ± 0.01 | 0.55 ± 0.02 | 0.78 ± 0.01 | 0.78 ± 0.01 | 0.77 ± 0.02 | 0.78 ± 0.02 | 0.78 ± 0.01 | 0.55 ± 0.02 | 0.78 ± 0.01 | 0.78 ± 0.01 | 0.77 ± 0.02 | 0.78 ± 0.03 |
|  |  | SVM | 0.77 ± 0.01 | 0.77 ± 0.02 | 0.53 ± 0.03 | 0.76 ± 0.02 | 0.76 ± 0.02 | 0.74 ± 0.02 | 0.81 ± 0.02 | 0.77 ± 0.02 | 0.53 ± 0.03 | 0.76 ± 0.02 | 0.76 ± 0.02 | 0.74 ± 0.02 | 0.81 ± 0.03 |
|  | 95 | AB | 0.72 ± 0.02 | 0.74 ± 0.02 | 0.46 ± 0.05 | 0.73 ± 0.02 | 0.73 ± 0.02 | 0.71 ± 0.02 | 0.76 ± 0.03 | 0.74 ± 0.03 | 0.46 ± 0.04 | 0.73 ± 0.02 | 0.73 ± 0.02 | 0.71 ± 0.02 | 0.77 ± 0.05 |
|  |  | CART | 0.72 ± 0.01 | 0.68 ± 0.02 | 0.35 ± 0.04 | 0.67 ± 0.02 | 0.67 ± 0.02 | 0.66 ± 0.02 | 0.69 ± 0.03 | 0.68 ± 0.02 | 0.35 ± 0.03 | 0.67 ± 0.02 | 0.67 ± 0.02 | 0.67 ± 0.02 | 0.69 ± 0.05 |

|  |  |  |  | **Validation set** | | | | | | **Test set** | | | | | |
| --- | --- | --- | --- | --- | --- | --- | --- | --- | --- | --- | --- | --- | --- | --- | --- |
| **Activity threshold** | **PCC** | **Algorithm** | **F1 train** | **F1** | **MCC** | **ROC AUC** | **Accuracy** | **Precision** | **Recall** | **F1** | **MCC** | **ROC AUC** | **Accuracy** | **Precision** | **Recall** |
| GAP 10 | 95 | ET | 0.7 ± 0.01 | 0.72 ± 0.02 | 0.45 ± 0.04 | 0.72 ± 0.02 | 0.72 ± 0.02 | 0.72 ± 0.02 | 0.72 ± 0.02 | 0.72 ± 0.01 | 0.44 ± 0.03 | 0.72 ± 0.02 | 0.72 ± 0.02 | 0.72 ± 0.03 | 0.73 ± 0.02 |
|  |  | GBM | 0.75 ± 0.01 | 0.75 ± 0.02 | 0.49 ± 0.03 | 0.74 ± 0.02 | 0.74 ± 0.02 | 0.74 ± 0.02 | 0.75 ± 0.02 | 0.75 ± 0.02 | 0.49 ± 0.03 | 0.75 ± 0.02 | 0.75 ± 0.02 | 0.74 ± 0.02 | 0.75 ± 0.03 |
|  |  | KNN | 0.77 ± 0.01 | 0.78 ± 0.02 | 0.56 ± 0.04 | 0.78 ± 0.02 | 0.78 ± 0.02 | 0.77 ± 0.02 | 0.8 ± 0.04 | 0.78 ± 0.02 | 0.56 ± 0.04 | 0.78 ± 0.02 | 0.78 ± 0.02 | 0.77 ± 0.02 | 0.8 ± 0.04 |
|  |  | LDA | 0.66 ± 0.02 | 0.69 ± 0.02 | 0.37 ± 0.04 | 0.68 ± 0.02 | 0.68 ± 0.02 | 0.68 ± 0.02 | 0.7 ± 0.02 | 0.69 ± 0.02 | 0.37 ± 0.04 | 0.68 ± 0.02 | 0.68 ± 0.02 | 0.68 ± 0.02 | 0.7 ± 0.03 |
|  |  | LR | 0.68 ± 0.02 | 0.68 ± 0.02 | 0.36 ± 0.04 | 0.68 ± 0.02 | 0.68 ± 0.02 | 0.67 ± 0.03 | 0.7 ± 0.02 | 0.68 ± 0.02 | 0.35 ± 0.04 | 0.67 ± 0.02 | 0.67 ± 0.02 | 0.67 ± 0.03 | 0.7 ± 0.04 |
|  |  | NB | 0.67 ± 0.01 | 0.67 ± 0.02 | 0.23 ± 0.05 | 0.61 ± 0.03 | 0.6 ± 0.03 | 0.58 ± 0.03 | 0.8 ± 0.07 | 0.67 ± 0.02 | 0.23 ± 0.05 | 0.61 ± 0.03 | 0.6 ± 0.03 | 0.58 ± 0.03 | 0.8 ± 0.07 |
|  |  | RF | 0.78 ± 0.01 | 0.78 ± 0.02 | 0.57 ± 0.03 | 0.78 ± 0.02 | 0.78 ± 0.02 | 0.78 ± 0.02 | 0.79 ± 0.02 | 0.78 ± 0.02 | 0.57 ± 0.03 | 0.78 ± 0.02 | 0.78 ± 0.02 | 0.78 ± 0.02 | 0.79 ± 0.03 |
|  |  | SVM | 0.76 ± 0.01 | 0.78 ± 0.01 | 0.53 ± 0.03 | 0.76 ± 0.02 | 0.76 ± 0.02 | 0.74 ± 0.02 | 0.82 ± 0.02 | 0.78 ± 0.02 | 0.54 ± 0.04 | 0.77 ± 0.02 | 0.77 ± 0.02 | 0.74 ± 0.03 | 0.82 ± 0.03 |
|  | 100 | AB | 0.72 ± 0.01 | 0.74 ± 0.03 | 0.46 ± 0.06 | 0.73 ± 0.03 | 0.73 ± 0.03 | 0.72 ± 0.03 | 0.76 ± 0.02 | 0.74 ± 0.03 | 0.46 ± 0.05 | 0.73 ± 0.02 | 0.73 ± 0.02 | 0.72 ± 0.02 | 0.75 ± 0.04 |
|  |  | CART | 0.72 ± 0.01 | 0.71 ± 0.03 | 0.41 ± 0.05 | 0.7 ± 0.02 | 0.7 ± 0.02 | 0.69 ± 0.03 | 0.73 ± 0.06 | 0.71 ± 0.03 | 0.41 ± 0.05 | 0.7 ± 0.02 | 0.7 ± 0.02 | 0.69 ± 0.03 | 0.73 ± 0.06 |
|  |  | ET | 0.7 ± 0.02 | 0.72 ± 0.01 | 0.43 ± 0.03 | 0.72 ± 0.02 | 0.72 ± 0.02 | 0.71 ± 0.02 | 0.72 ± 0.02 | 0.72 ± 0.02 | 0.43 ± 0.03 | 0.72 ± 0.02 | 0.72 ± 0.02 | 0.71 ± 0.02 | 0.72 ± 0.03 |
|  |  | GBM | 0.74 ± 0.01 | 0.74 ± 0.02 | 0.48 ± 0.04 | 0.74 ± 0.02 | 0.74 ± 0.02 | 0.73 ± 0.02 | 0.75 ± 0.03 | 0.74 ± 0.02 | 0.48 ± 0.04 | 0.74 ± 0.02 | 0.74 ± 0.02 | 0.74 ± 0.02 | 0.74 ± 0.03 |
|  |  | KNN | 0.77 ± 0.02 | 0.78 ± 0.02 | 0.56 ± 0.03 | 0.78 ± 0.02 | 0.78 ± 0.02 | 0.77 ± 0.02 | 0.79 ± 0.03 | 0.78 ± 0.02 | 0.56 ± 0.03 | 0.78 ± 0.02 | 0.78 ± 0.02 | 0.77 ± 0.02 | 0.79 ± 0.03 |
|  |  | LDA | 0.69 ± 0.01 | 0.7 ± 0.02 | 0.39 ± 0.04 | 0.7 ± 0.02 | 0.7 ± 0.02 | 0.69 ± 0.02 | 0.71 ± 0.02 | 0.69 ± 0.01 | 0.38 ± 0.03 | 0.69 ± 0.02 | 0.69 ± 0.02 | 0.68 ± 0.02 | 0.7 ± 0.03 |
|  |  | LR | 0.69 ± 0.01 | 0.7 ± 0.02 | 0.38 ± 0.05 | 0.69 ± 0.03 | 0.69 ± 0.03 | 0.68 ± 0.03 | 0.71 ± 0.03 | 0.69 ± 0.02 | 0.37 ± 0.04 | 0.69 ± 0.02 | 0.69 ± 0.02 | 0.68 ± 0.03 | 0.71 ± 0.03 |
|  |  | NB | 0.67 ± 0.01 | 0.67 ± 0.02 | 0.25 ± 0.04 | 0.62 ± 0.02 | 0.62 ± 0.02 | 0.58 ± 0.02 | 0.78 ± 0.04 | 0.67 ± 0.02 | 0.25 ± 0.04 | 0.62 ± 0.02 | 0.62 ± 0.02 | 0.59 ± 0.01 | 0.78 ± 0.04 |
|  |  | RF | 0.78 ± 0.01 | 0.79 ± 0.02 | 0.57 ± 0.03 | 0.79 ± 0.02 | 0.79 ± 0.02 | 0.78 ± 0.01 | 0.8 ± 0.03 | 0.79 ± 0.02 | 0.57 ± 0.04 | 0.79 ± 0.02 | 0.79 ± 0.02 | 0.78 ± 0.02 | 0.8 ± 0.04 |
|  |  | SVM | 0.76 ± 0.01 | 0.77 ± 0.02 | 0.53 ± 0.03 | 0.76 ± 0.02 | 0.76 ± 0.02 | 0.73 ± 0.02 | 0.82 ± 0.03 | 0.77 ± 0.02 | 0.53 ± 0.04 | 0.76 ± 0.02 | 0.76 ± 0.02 | 0.74 ± 0.03 | 0.82 ± 0.04 |
|  |  |  |  |  |  |  |  |  |  |  |  |  |  |  |  |
| GAP 15 | 75 | AB | 0.73 ± 0.02 | 0.74 ± 0.01 | 0.47 ± 0.03 | 0.73 ± 0.02 | 0.73 ± 0.02 | 0.72 ± 0.02 | 0.76 ± 0.02 | 0.74 ± 0.01 | 0.46 ± 0.02 | 0.73 ± 0.01 | 0.73 ± 0.01 | 0.72 ± 0.02 | 0.76 ± 0.03 |
|  |  | CART | 0.72 ± 0.01 | 0.68 ± 0.03 | 0.35 ± 0.06 | 0.67 ± 0.03 | 0.67 ± 0.03 | 0.66 ± 0.03 | 0.71 ± 0.07 | 0.68 ± 0.03 | 0.35 ± 0.06 | 0.67 ± 0.03 | 0.67 ± 0.03 | 0.66 ± 0.03 | 0.71 ± 0.07 |
|  |  |  |  | **Validation set** | | | | | | **Test set** | | | | | |
| **Activity threshold** | **PCC** | **Algorithm** | **F1 train** | **F1** | **MCC** | **ROC AUC** | **Accuracy** | **Precision** | **Recall** | **F1** | **MCC** | **ROC AUC** | **Accuracy** | **Precision** | **Recall** |
| GAP 15 | 75 | ET | 0.73 ± 0.0 | 0.72 ± 0.01 | 0.72 ± 0.02 | 0.43 ± 0.04 | 0.72 ± 0.02 | 0.72 ± 0.02 | 0.71 ± 0.02 | 0.72 ± 0.03 | 0.72 ± 0.02 | 0.43 ± 0.04 | 0.72 ± 0.02 | 0.72 ± 0.02 | 0.71 ± 0.02 |
|  |  | GBM | 0.72 ± 0.01 | 0.75 ± 0.01 | 0.74 ± 0.02 | 0.49 ± 0.03 | 0.74 ± 0.02 | 0.74 ± 0.02 | 0.74 ± 0.02 | 0.75 ± 0.02 | 0.74 ± 0.01 | 0.48 ± 0.04 | 0.74 ± 0.02 | 0.74 ± 0.02 | 0.74 ± 0.04 |
|  |  | KNN | 0.79 ± 0.01 | 0.78 ± 0.02 | 0.78 ± 0.03 | 0.56 ± 0.04 | 0.78 ± 0.02 | 0.78 ± 0.02 | 0.77 ± 0.02 | 0.79 ± 0.04 | 0.78 ± 0.03 | 0.56 ± 0.04 | 0.78 ± 0.02 | 0.78 ± 0.02 | 0.77 ± 0.02 |
|  |  | LDA | 0.68 ± 0.01 | 0.68 ± 0.02 | 0.68 ± 0.02 | 0.35 ± 0.04 | 0.68 ± 0.02 | 0.68 ± 0.02 | 0.67 ± 0.02 | 0.68 ± 0.03 | 0.68 ± 0.02 | 0.35 ± 0.03 | 0.68 ± 0.02 | 0.68 ± 0.02 | 0.67 ± 0.02 |
|  |  | LR | 0.68 ± 0.01 | 0.69 ± 0.01 | 0.67 ± 0.02 | 0.33 ± 0.04 | 0.66 ± 0.02 | 0.66 ± 0.02 | 0.66 ± 0.02 | 0.68 ± 0.02 | 0.67 ± 0.02 | 0.33 ± 0.03 | 0.66 ± 0.02 | 0.66 ± 0.02 | 0.66 ± 0.03 |
|  |  | NB | 0.69 ± 0.01 | 0.68 ± 0.01 | 0.66 ± 0.02 | 0.22 ± 0.08 | 0.6 ± 0.04 | 0.6 ± 0.04 | 0.58 ± 0.04 | 0.77 ± 0.09 | 0.66 ± 0.02 | 0.22 ± 0.08 | 0.6 ± 0.04 | 0.6 ± 0.04 | 0.58 ± 0.04 |
|  |  | RF | 0.79 ± 0.01 | 0.79 ± 0.01 | 0.78 ± 0.02 | 0.56 ± 0.04 | 0.78 ± 0.02 | 0.78 ± 0.02 | 0.78 ± 0.02 | 0.78 ± 0.02 | 0.78 ± 0.02 | 0.56 ± 0.04 | 0.78 ± 0.02 | 0.78 ± 0.02 | 0.78 ± 0.03 |
|  |  | SVM | 0.79 ± 0.01 | 0.78 ± 0.01 | 0.78 ± 0.02 | 0.54 ± 0.04 | 0.77 ± 0.02 | 0.77 ± 0.02 | 0.75 ± 0.03 | 0.8 ± 0.04 | 0.77 ± 0.02 | 0.54 ± 0.05 | 0.77 ± 0.02 | 0.77 ± 0.02 | 0.75 ± 0.03 |
|  | 85 | AB | 0.73 ± 0.01 | 0.74 ± 0.01 | 0.75 ± 0.02 | 0.5 ± 0.03 | 0.75 ± 0.02 | 0.75 ± 0.02 | 0.73 ± 0.02 | 0.78 ± 0.02 | 0.75 ± 0.02 | 0.5 ± 0.03 | 0.75 ± 0.02 | 0.75 ± 0.02 | 0.73 ± 0.03 |
|  |  | CART | 0.71 ± 0.01 | 0.72 ± 0.01 | 0.7 ± 0.04 | 0.4 ± 0.07 | 0.7 ± 0.04 | 0.7 ± 0.03 | 0.7 ± 0.04 | 0.71 ± 0.04 | 0.7 ± 0.03 | 0.4 ± 0.07 | 0.7 ± 0.04 | 0.7 ± 0.04 | 0.7 ± 0.04 |
|  |  | ET | 0.74 ± 0.01 | 0.72 ± 0.01 | 0.72 ± 0.02 | 0.43 ± 0.04 | 0.72 ± 0.02 | 0.72 ± 0.02 | 0.71 ± 0.02 | 0.72 ± 0.02 | 0.72 ± 0.02 | 0.44 ± 0.04 | 0.72 ± 0.02 | 0.72 ± 0.02 | 0.72 ± 0.02 |
|  |  | GBM | 0.74 ± 0.01 | 0.75 ± 0.01 | 0.74 ± 0.02 | 0.49 ± 0.04 | 0.74 ± 0.02 | 0.74 ± 0.02 | 0.73 ± 0.02 | 0.76 ± 0.02 | 0.75 ± 0.02 | 0.49 ± 0.04 | 0.75 ± 0.02 | 0.75 ± 0.02 | 0.74 ± 0.03 |
|  |  | KNN | 0.79 ± 0.01 | 0.78 ± 0.02 | 0.78 ± 0.02 | 0.55 ± 0.04 | 0.77 ± 0.02 | 0.77 ± 0.02 | 0.76 ± 0.02 | 0.79 ± 0.04 | 0.78 ± 0.02 | 0.55 ± 0.04 | 0.77 ± 0.02 | 0.77 ± 0.02 | 0.76 ± 0.02 |
|  |  | LDA | 0.67 ± 0.02 | 0.68 ± 0.01 | 0.69 ± 0.02 | 0.38 ± 0.02 | 0.69 ± 0.01 | 0.69 ± 0.01 | 0.68 ± 0.01 | 0.69 ± 0.02 | 0.68 ± 0.02 | 0.36 ± 0.02 | 0.68 ± 0.01 | 0.68 ± 0.01 | 0.68 ± 0.02 |
|  |  | LR | 0.68 ± 0.01 | 0.69 ± 0.01 | 0.67 ± 0.02 | 0.34 ± 0.04 | 0.67 ± 0.02 | 0.67 ± 0.02 | 0.66 ± 0.02 | 0.68 ± 0.02 | 0.67 ± 0.02 | 0.33 ± 0.04 | 0.66 ± 0.02 | 0.66 ± 0.02 | 0.66 ± 0.03 |
|  |  | NB | 0.68 ± 0.02 | 0.68 ± 0.01 | 0.66 ± 0.01 | 0.23 ± 0.04 | 0.61 ± 0.02 | 0.61 ± 0.02 | 0.58 ± 0.03 | 0.78 ± 0.07 | 0.66 ± 0.01 | 0.24 ± 0.04 | 0.61 ± 0.03 | 0.61 ± 0.03 | 0.58 ± 0.03 |
|  |  | RF | 0.8 ± 0.01 | 0.79 ± 0.01 | 0.78 ± 0.02 | 0.56 ± 0.05 | 0.78 ± 0.02 | 0.78 ± 0.02 | 0.78 ± 0.03 | 0.78 ± 0.03 | 0.78 ± 0.02 | 0.56 ± 0.04 | 0.78 ± 0.02 | 0.78 ± 0.02 | 0.78 ± 0.03 |
|  |  | SVM | 0.79 ± 0.01 | 0.78 ± 0.01 | 0.77 ± 0.02 | 0.54 ± 0.04 | 0.77 ± 0.02 | 0.77 ± 0.02 | 0.76 ± 0.02 | 0.79 ± 0.04 | 0.78 ± 0.02 | 0.54 ± 0.04 | 0.77 ± 0.02 | 0.77 ± 0.02 | 0.76 ± 0.03 |
|  | 95 | AB | 0.73 ± 0.01 | 0.74 ± 0.01 | 0.74 ± 0.02 | 0.46 ± 0.05 | 0.73 ± 0.02 | 0.73 ± 0.02 | 0.71 ± 0.03 | 0.76 ± 0.02 | 0.74 ± 0.02 | 0.46 ± 0.04 | 0.73 ± 0.02 | 0.73 ± 0.02 | 0.71 ± 0.03 |
|  |  | CART | 0.71 ± 0.01 | 0.73 ± 0.01 | 0.69 ± 0.03 | 0.38 ± 0.06 | 0.69 ± 0.03 | 0.69 ± 0.03 | 0.68 ± 0.04 | 0.7 ± 0.06 | 0.69 ± 0.03 | 0.38 ± 0.07 | 0.69 ± 0.03 | 0.69 ± 0.03 | 0.69 ± 0.05 |
|  |  | ET | 0.74 ± 0.0 | 0.72 ± 0.01 | 0.72 ± 0.02 | 0.43 ± 0.04 | 0.72 ± 0.02 | 0.72 ± 0.02 | 0.71 ± 0.02 | 0.72 ± 0.02 | 0.71 ± 0.02 | 0.43 ± 0.05 | 0.72 ± 0.02 | 0.72 ± 0.02 | 0.71 ± 0.03 |
|  |  | GBM | 0.74 ± 0.01 | 0.76 ± 0.01 | 0.76 ± 0.02 | 0.52 ± 0.04 | 0.76 ± 0.02 | 0.76 ± 0.02 | 0.75 ± 0.02 | 0.78 ± 0.03 | 0.76 ± 0.02 | 0.51 ± 0.04 | 0.76 ± 0.02 | 0.76 ± 0.02 | 0.75 ± 0.03 |
|  |  |  |  | **Validation set** | | | | | | **Test set** | | | | | |
| **Activity threshold** | **PCC** | **Algorithm** | **F1 train** | **F1** | **MCC** | **ROC AUC** | **Accuracy** | **Precision** | **Recall** | **F1** | **MCC** | **ROC AUC** | **Accuracy** | **Precision** | **Recall** |
| GAP 15 | 95 | KNN | 0.78 ± 0.02 | 0.78 ± 0.02 | 0.56 ± 0.03 | 0.78 ± 0.02 | 0.78 ± 0.02 | 0.77 ± 0.02 | 0.8 ± 0.03 | 0.78 ± 0.02 | 0.56 ± 0.03 | 0.78 ± 0.02 | 0.78 ± 0.02 | 0.77 ± 0.02 | 0.8 ± 0.03 |
|  |  | LDA | 0.69 ± 0.01 | 0.71 ± 0.02 | 0.41 ± 0.03 | 0.7 ± 0.02 | 0.7 ± 0.02 | 0.69 ± 0.01 | 0.72 ± 0.03 | 0.71 ± 0.01 | 0.41 ± 0.02 | 0.7 ± 0.01 | 0.7 ± 0.01 | 0.7 ± 0.01 | 0.72 ± 0.02 |
|  |  | LR | 0.7 ± 0.01 | 0.7 ± 0.02 | 0.4 ± 0.04 | 0.7 ± 0.02 | 0.7 ± 0.02 | 0.69 ± 0.02 | 0.72 ± 0.03 | 0.7 ± 0.02 | 0.39 ± 0.03 | 0.7 ± 0.02 | 0.7 ± 0.02 | 0.69 ± 0.02 | 0.71 ± 0.04 |
|  |  | NB | 0.68 ± 0.01 | 0.66 ± 0.01 | 0.24 ± 0.02 | 0.61 ± 0.01 | 0.61 ± 0.01 | 0.58 ± 0.01 | 0.77 ± 0.04 | 0.66 ± 0.01 | 0.24 ± 0.03 | 0.61 ± 0.02 | 0.61 ± 0.02 | 0.58 ± 0.01 | 0.78 ± 0.04 |
|  |  | RF | 0.8 ± 0.02 | 0.78 ± 0.03 | 0.57 ± 0.05 | 0.78 ± 0.03 | 0.78 ± 0.03 | 0.78 ± 0.03 | 0.78 ± 0.03 | 0.78 ± 0.02 | 0.56 ± 0.04 | 0.78 ± 0.02 | 0.78 ± 0.02 | 0.78 ± 0.03 | 0.78 ± 0.03 |
|  |  | SVM | 0.78 ± 0.02 | 0.77 ± 0.03 | 0.54 ± 0.04 | 0.77 ± 0.02 | 0.77 ± 0.02 | 0.76 ± 0.03 | 0.79 ± 0.06 | 0.77 ± 0.03 | 0.54 ± 0.04 | 0.77 ± 0.02 | 0.77 ± 0.02 | 0.76 ± 0.03 | 0.79 ± 0.07 |
|  | 100 | AB | 0.74 ± 0.01 | 0.74 ± 0.02 | 0.47 ± 0.04 | 0.73 ± 0.02 | 0.73 ± 0.02 | 0.72 ± 0.03 | 0.77 ± 0.02 | 0.74 ± 0.02 | 0.46 ± 0.04 | 0.73 ± 0.02 | 0.73 ± 0.02 | 0.72 ± 0.03 | 0.77 ± 0.04 |
|  |  | CART | 0.72 ± 0.01 | 0.68 ± 0.04 | 0.37 ± 0.07 | 0.68 ± 0.04 | 0.68 ± 0.04 | 0.68 ± 0.04 | 0.7 ± 0.06 | 0.69 ± 0.04 | 0.37 ± 0.07 | 0.68 ± 0.04 | 0.68 ± 0.04 | 0.68 ± 0.04 | 0.7 ± 0.06 |
|  |  | ET | 0.72 ± 0.01 | 0.72 ± 0.02 | 0.44 ± 0.04 | 0.72 ± 0.02 | 0.72 ± 0.02 | 0.72 ± 0.03 | 0.72 ± 0.02 | 0.72 ± 0.02 | 0.44 ± 0.04 | 0.72 ± 0.02 | 0.72 ± 0.02 | 0.72 ± 0.03 | 0.72 ± 0.03 |
|  |  | GBM | 0.76 ± 0.01 | 0.76 ± 0.02 | 0.51 ± 0.03 | 0.75 ± 0.02 | 0.75 ± 0.02 | 0.75 ± 0.02 | 0.77 ± 0.02 | 0.75 ± 0.02 | 0.5 ± 0.03 | 0.75 ± 0.02 | 0.75 ± 0.02 | 0.74 ± 0.02 | 0.76 ± 0.03 |
|  |  | KNN | 0.78 ± 0.02 | 0.78 ± 0.02 | 0.55 ± 0.03 | 0.78 ± 0.02 | 0.78 ± 0.02 | 0.76 ± 0.01 | 0.8 ± 0.03 | 0.78 ± 0.02 | 0.55 ± 0.03 | 0.78 ± 0.02 | 0.78 ± 0.02 | 0.76 ± 0.01 | 0.8 ± 0.03 |
|  |  | LDA | 0.7 ± 0.02 | 0.7 ± 0.02 | 0.4 ± 0.05 | 0.7 ± 0.02 | 0.7 ± 0.02 | 0.7 ± 0.02 | 0.71 ± 0.03 | 0.7 ± 0.02 | 0.41 ± 0.04 | 0.7 ± 0.02 | 0.7 ± 0.02 | 0.7 ± 0.02 | 0.71 ± 0.03 |
|  |  | LR | 0.7 ± 0.01 | 0.7 ± 0.02 | 0.39 ± 0.04 | 0.7 ± 0.02 | 0.7 ± 0.02 | 0.69 ± 0.02 | 0.72 ± 0.02 | 0.7 ± 0.01 | 0.4 ± 0.03 | 0.7 ± 0.02 | 0.7 ± 0.02 | 0.69 ± 0.03 | 0.72 ± 0.03 |
|  |  | NB | 0.67 ± 0.01 | 0.67 ± 0.01 | 0.21 ± 0.04 | 0.59 ± 0.02 | 0.59 ± 0.02 | 0.56 ± 0.02 | 0.82 ± 0.05 | 0.66 ± 0.02 | 0.21 ± 0.04 | 0.59 ± 0.02 | 0.59 ± 0.02 | 0.56 ± 0.02 | 0.81 ± 0.06 |
|  |  | RF | 0.79 ± 0.01 | 0.78 ± 0.02 | 0.57 ± 0.05 | 0.78 ± 0.02 | 0.78 ± 0.02 | 0.78 ± 0.03 | 0.79 ± 0.03 | 0.78 ± 0.02 | 0.56 ± 0.05 | 0.78 ± 0.03 | 0.78 ± 0.03 | 0.78 ± 0.04 | 0.79 ± 0.03 |
|  |  | SVM | 0.78 ± 0.02 | 0.77 ± 0.03 | 0.53 ± 0.05 | 0.76 ± 0.02 | 0.76 ± 0.02 | 0.75 ± 0.02 | 0.78 ± 0.04 | 0.77 ± 0.03 | 0.53 ± 0.06 | 0.76 ± 0.03 | 0.76 ± 0.03 | 0.75 ± 0.03 | 0.78 ± 0.05 |
|  |  |  |  |  |  |  |  |  |  |  |  |  |  |  |  |
| GAP 20 | 75 | AB | 0.74 ± 0.02 | 0.76 ± 0.02 | 0.51 ± 0.04 | 0.75 ± 0.02 | 0.75 ± 0.02 | 0.73 ± 0.02 | 0.79 ± 0.03 | 0.76 ± 0.02 | 0.51 ± 0.04 | 0.75 ± 0.02 | 0.75 ± 0.02 | 0.74 ± 0.02 | 0.79 ± 0.04 |
|  |  | CART | 0.73 ± 0.0 | 0.7 ± 0.02 | 0.38 ± 0.04 | 0.69 ± 0.02 | 0.69 ± 0.02 | 0.68 ± 0.03 | 0.72 ± 0.05 | 0.7 ± 0.02 | 0.38 ± 0.04 | 0.69 ± 0.02 | 0.69 ± 0.02 | 0.68 ± 0.03 | 0.72 ± 0.05 |
|  |  | ET | 0.72 ± 0.01 | 0.73 ± 0.02 | 0.47 ± 0.05 | 0.73 ± 0.02 | 0.73 ± 0.02 | 0.73 ± 0.02 | 0.74 ± 0.03 | 0.74 ± 0.03 | 0.48 ± 0.06 | 0.74 ± 0.03 | 0.74 ± 0.03 | 0.73 ± 0.02 | 0.74 ± 0.06 |
|  |  | GBM | 0.75 ± 0.02 | 0.76 ± 0.03 | 0.51 ± 0.06 | 0.76 ± 0.03 | 0.76 ± 0.03 | 0.75 ± 0.03 | 0.77 ± 0.03 | 0.76 ± 0.03 | 0.51 ± 0.05 | 0.76 ± 0.03 | 0.76 ± 0.03 | 0.75 ± 0.03 | 0.77 ± 0.04 |

|  |  |  |  | **Validation set** | | | | | | **Test set** | | | | | |
| --- | --- | --- | --- | --- | --- | --- | --- | --- | --- | --- | --- | --- | --- | --- | --- |
| **Activity threshold** | **PCC** | **Algorithm** | **F1 train** | **F1** | **MCC** | **ROC AUC** | **Accuracy** | **Precision** | **Recall** | **F1** | **MCC** | **ROC AUC** | **Accuracy** | **Precision** | **Recall** |
| GAP 20 | 75 | KNN | 0.78 ± 0.01 | 0.8 ± 0.01 | 0.59 ± 0.03 | 0.79 ± 0.01 | 0.79 ± 0.01 | 0.78 ± 0.02 | 0.81 ± 0.03 | 0.8 ± 0.01 | 0.59 ± 0.03 | 0.79 ± 0.01 | 0.79 ± 0.01 | 0.78 ± 0.02 | 0.81 ± 0.03 |
|  |  | LDA | 0.69 ± 0.02 | 0.7 ± 0.02 | 0.39 ± 0.03 | 0.69 ± 0.02 | 0.69 ± 0.02 | 0.68 ± 0.02 | 0.72 ± 0.02 | 0.7 ± 0.02 | 0.39 ± 0.03 | 0.69 ± 0.01 | 0.69 ± 0.01 | 0.68 ± 0.02 | 0.72 ± 0.04 |
|  |  | LR | 0.69 ± 0.02 | 0.58 ± 0.26 | 0.32 ± 0.15 | 0.66 ± 0.07 | 0.66 ± 0.07 | 0.56 ± 0.25 | 0.6 ± 0.27 | 0.58 ± 0.26 | 0.32 ± 0.14 | 0.66 ± 0.07 | 0.66 ± 0.07 | 0.56 ± 0.25 | 0.6 ± 0.27 |
|  |  | NB | 0.68 ± 0.01 | 0.68 ± 0.02 | 0.31 ± 0.05 | 0.65 ± 0.02 | 0.65 ± 0.02 | 0.62 ± 0.02 | 0.77 ± 0.04 | 0.69 ± 0.03 | 0.31 ± 0.06 | 0.65 ± 0.03 | 0.65 ± 0.03 | 0.62 ± 0.03 | 0.78 ± 0.05 |
|  |  | RF | 0.79 ± 0.01 | 0.8 ± 0.01 | 0.59 ± 0.03 | 0.8 ± 0.01 | 0.8 ± 0.02 | 0.79 ± 0.02 | 0.8 ± 0.02 | 0.79 ± 0.01 | 0.59 ± 0.03 | 0.8 ± 0.01 | 0.8 ± 0.01 | 0.79 ± 0.02 | 0.8 ± 0.02 |
|  |  | SVM | 0.78 ± 0.01 | 0.79 ± 0.03 | 0.56 ± 0.06 | 0.78 ± 0.03 | 0.78 ± 0.03 | 0.76 ± 0.03 | 0.83 ± 0.04 | 0.79 ± 0.03 | 0.56 ± 0.06 | 0.78 ± 0.03 | 0.78 ± 0.03 | 0.76 ± 0.03 | 0.82 ± 0.05 |
|  | 85 | AB | 0.76 ± 0.01 | 0.77 ± 0.02 | 0.52 ± 0.04 | 0.76 ± 0.02 | 0.76 ± 0.02 | 0.74 ± 0.03 | 0.8 ± 0.04 | 0.77 ± 0.02 | 0.52 ± 0.04 | 0.76 ± 0.02 | 0.76 ± 0.02 | 0.74 ± 0.03 | 0.8 ± 0.04 |
|  |  | CART | 0.72 ± 0.01 | 0.7 ± 0.03 | 0.41 ± 0.05 | 0.7 ± 0.02 | 0.7 ± 0.02 | 0.7 ± 0.03 | 0.71 ± 0.04 | 0.7 ± 0.02 | 0.41 ± 0.04 | 0.7 ± 0.02 | 0.7 ± 0.02 | 0.7 ± 0.03 | 0.71 ± 0.04 |
|  |  | ET | 0.71 ± 0.01 | 0.73 ± 0.03 | 0.46 ± 0.05 | 0.73 ± 0.02 | 0.73 ± 0.02 | 0.73 ± 0.03 | 0.73 ± 0.03 | 0.73 ± 0.02 | 0.46 ± 0.04 | 0.73 ± 0.02 | 0.73 ± 0.02 | 0.72 ± 0.03 | 0.73 ± 0.05 |
|  |  | GBM | 0.76 ± 0.01 | 0.77 ± 0.02 | 0.53 ± 0.04 | 0.77 ± 0.02 | 0.77 ± 0.02 | 0.76 ± 0.02 | 0.78 ± 0.02 | 0.77 ± 0.02 | 0.53 ± 0.04 | 0.76 ± 0.02 | 0.76 ± 0.02 | 0.76 ± 0.02 | 0.78 ± 0.03 |
|  |  | KNN | 0.79 ± 0.01 | 0.8 ± 0.02 | 0.6 ± 0.03 | 0.8 ± 0.02 | 0.8 ± 0.02 | 0.79 ± 0.02 | 0.81 ± 0.02 | 0.8 ± 0.02 | 0.6 ± 0.03 | 0.8 ± 0.02 | 0.8 ± 0.02 | 0.79 ± 0.02 | 0.81 ± 0.02 |
|  |  | LDA | 0.7 ± 0.01 | 0.71 ± 0.02 | 0.41 ± 0.04 | 0.71 ± 0.02 | 0.71 ± 0.02 | 0.7 ± 0.02 | 0.73 ± 0.03 | 0.71 ± 0.02 | 0.41 ± 0.03 | 0.71 ± 0.02 | 0.71 ± 0.02 | 0.7 ± 0.02 | 0.73 ± 0.04 |
|  |  | LR | 0.7 ± 0.02 | 0.71 ± 0.02 | 0.41 ± 0.04 | 0.71 ± 0.02 | 0.71 ± 0.02 | 0.69 ± 0.02 | 0.73 ± 0.03 | 0.72 ± 0.02 | 0.42 ± 0.04 | 0.71 ± 0.02 | 0.71 ± 0.02 | 0.7 ± 0.02 | 0.74 ± 0.04 |
|  |  | NB | 0.68 ± 0.01 | 0.68 ± 0.02 | 0.29 ± 0.04 | 0.64 ± 0.02 | 0.64 ± 0.02 | 0.6 ± 0.02 | 0.8 ± 0.04 | 0.68 ± 0.02 | 0.29 ± 0.04 | 0.64 ± 0.02 | 0.63 ± 0.02 | 0.6 ± 0.02 | 0.79 ± 0.06 |
|  |  | RF | 0.79 ± 0.02 | 0.8 ± 0.02 | 0.59 ± 0.05 | 0.8 ± 0.02 | 0.8 ± 0.02 | 0.8 ± 0.03 | 0.8 ± 0.02 | 0.8 ± 0.02 | 0.6 ± 0.04 | 0.8 ± 0.02 | 0.8 ± 0.02 | 0.8 ± 0.02 | 0.8 ± 0.03 |
|  |  | SVM | 0.78 ± 0.01 | 0.79 ± 0.03 | 0.57 ± 0.07 | 0.79 ± 0.03 | 0.79 ± 0.03 | 0.77 ± 0.04 | 0.81 ± 0.04 | 0.79 ± 0.03 | 0.57 ± 0.06 | 0.78 ± 0.03 | 0.78 ± 0.03 | 0.77 ± 0.03 | 0.81 ± 0.05 |
|  | 95 | AB | 0.76 ± 0.01 | 0.77 ± 0.02 | 0.53 ± 0.05 | 0.77 ± 0.02 | 0.77 ± 0.02 | 0.75 ± 0.03 | 0.79 ± 0.02 | 0.77 ± 0.02 | 0.53 ± 0.04 | 0.76 ± 0.02 | 0.76 ± 0.02 | 0.75 ± 0.02 | 0.79 ± 0.02 |
|  |  | CART | 0.73 ± 0.01 | 0.69 ± 0.03 | 0.39 ± 0.06 | 0.69 ± 0.03 | 0.69 ± 0.03 | 0.7 ± 0.03 | 0.68 ± 0.04 | 0.69 ± 0.03 | 0.39 ± 0.06 | 0.69 ± 0.03 | 0.69 ± 0.03 | 0.7 ± 0.03 | 0.68 ± 0.04 |
|  |  | ET | 0.71 ± 0.01 | 0.73 ± 0.02 | 0.46 ± 0.04 | 0.73 ± 0.02 | 0.73 ± 0.02 | 0.73 ± 0.02 | 0.73 ± 0.03 | 0.73 ± 0.03 | 0.46 ± 0.05 | 0.73 ± 0.02 | 0.73 ± 0.02 | 0.72 ± 0.03 | 0.73 ± 0.05 |
|  |  | GBM | 0.76 ± 0.01 | 0.77 ± 0.02 | 0.54 ± 0.05 | 0.77 ± 0.02 | 0.77 ± 0.02 | 0.76 ± 0.03 | 0.77 ± 0.02 | 0.77 ± 0.03 | 0.54 ± 0.05 | 0.77 ± 0.02 | 0.77 ± 0.02 | 0.76 ± 0.03 | 0.77 ± 0.03 |
|  |  | KNN | 0.79 ± 0.01 | 0.8 ± 0.01 | 0.61 ± 0.03 | 0.8 ± 0.01 | 0.8 ± 0.01 | 0.8 ± 0.02 | 0.81 ± 0.02 | 0.8 ± 0.01 | 0.61 ± 0.03 | 0.8 ± 0.01 | 0.8 ± 0.01 | 0.8 ± 0.02 | 0.81 ± 0.02 |
|  |  | LDA | 0.7 ± 0.01 | 0.72 ± 0.02 | 0.44 ± 0.04 | 0.72 ± 0.02 | 0.72 ± 0.02 | 0.7 ± 0.02 | 0.74 ± 0.02 | 0.72 ± 0.02 | 0.44 ± 0.03 | 0.72 ± 0.02 | 0.72 ± 0.02 | 0.71 ± 0.02 | 0.74 ± 0.04 |

|  |  |  |  | **Validation set** | | | | | | **Test set** | | | | | |
| --- | --- | --- | --- | --- | --- | --- | --- | --- | --- | --- | --- | --- | --- | --- | --- |
| **Activity threshold** | **PCC** | **Algorithm** | **F1 train** | **F1** | **MCC** | **ROC AUC** | **Accuracy** | **Precision** | **Recall** | **F1** | **MCC** | **ROC AUC** | **Accuracy** | **Precision** | **Recall** |
| GAP 20 | 95 | LR | 0.7 ± 0.01 | 0.72 ± 0.01 | 0.43 ± 0.03 | 0.72 ± 0.02 | 0.71 ± 0.02 | 0.7 ± 0.02 | 0.75 ± 0.02 | 0.72 ± 0.01 | 0.43 ± 0.03 | 0.72 ± 0.01 | 0.72 ± 0.01 | 0.7 ± 0.02 | 0.75 ± 0.03 |
|  |  | NB | 0.68 ± 0.01 | 0.69 ± 0.02 | 0.29 ± 0.05 | 0.64 ± 0.02 | 0.64 ± 0.02 | 0.6 ± 0.02 | 0.81 ± 0.04 | 0.69 ± 0.02 | 0.29 ± 0.06 | 0.64 ± 0.02 | 0.63 ± 0.02 | 0.6 ± 0.02 | 0.81 ± 0.05 |
|  |  | RF | 0.79 ± 0.01 | 0.8 ± 0.02 | 0.6 ± 0.04 | 0.8 ± 0.02 | 0.8 ± 0.02 | 0.8 ± 0.02 | 0.8 ± 0.03 | 0.8 ± 0.02 | 0.6 ± 0.04 | 0.8 ± 0.02 | 0.8 ± 0.02 | 0.8 ± 0.02 | 0.8 ± 0.03 |
|  |  | SVM | 0.78 ± 0.01 | 0.78 ± 0.03 | 0.55 ± 0.05 | 0.78 ± 0.03 | 0.78 ± 0.03 | 0.76 ± 0.03 | 0.8 ± 0.03 | 0.78 ± 0.02 | 0.55 ± 0.05 | 0.78 ± 0.02 | 0.78 ± 0.02 | 0.76 ± 0.02 | 0.81 ± 0.03 |
|  | 100 | AB | 0.75 ± 0.02 | 0.78 ± 0.02 | 0.54 ± 0.04 | 0.77 ± 0.02 | 0.77 ± 0.02 | 0.75 ± 0.02 | 0.8 ± 0.02 | 0.77 ± 0.02 | 0.53 ± 0.04 | 0.77 ± 0.02 | 0.77 ± 0.02 | 0.75 ± 0.02 | 0.8 ± 0.03 |
|  |  | CART | 0.73 ± 0.01 | 0.7 ± 0.05 | 0.39 ± 0.1 | 0.7 ± 0.05 | 0.7 ± 0.05 | 0.69 ± 0.05 | 0.71 ± 0.06 | 0.69 ± 0.06 | 0.39 ± 0.1 | 0.69 ± 0.05 | 0.69 ± 0.05 | 0.69 ± 0.05 | 0.7 ± 0.09 |
|  |  | ET | 0.72 ± 0.01 | 0.74 ± 0.02 | 0.47 ± 0.04 | 0.74 ± 0.02 | 0.74 ± 0.02 | 0.73 ± 0.02 | 0.74 ± 0.03 | 0.73 ± 0.03 | 0.47 ± 0.05 | 0.73 ± 0.02 | 0.73 ± 0.02 | 0.73 ± 0.02 | 0.74 ± 0.06 |
|  |  | GBM | 0.76 ± 0.01 | 0.77 ± 0.02 | 0.54 ± 0.05 | 0.77 ± 0.02 | 0.77 ± 0.02 | 0.76 ± 0.03 | 0.78 ± 0.03 | 0.77 ± 0.02 | 0.54 ± 0.05 | 0.77 ± 0.02 | 0.77 ± 0.02 | 0.76 ± 0.02 | 0.78 ± 0.03 |
|  |  | KNN | 0.78 ± 0.01 | 0.79 ± 0.02 | 0.59 ± 0.04 | 0.79 ± 0.02 | 0.79 ± 0.02 | 0.78 ± 0.02 | 0.81 ± 0.04 | 0.79 ± 0.02 | 0.59 ± 0.04 | 0.79 ± 0.02 | 0.79 ± 0.02 | 0.78 ± 0.02 | 0.81 ± 0.04 |
|  |  | LDA | 0.72 ± 0.01 | 0.73 ± 0.02 | 0.44 ± 0.03 | 0.72 ± 0.02 | 0.72 ± 0.02 | 0.71 ± 0.02 | 0.75 ± 0.02 | 0.72 ± 0.02 | 0.44 ± 0.04 | 0.72 ± 0.02 | 0.72 ± 0.02 | 0.71 ± 0.03 | 0.74 ± 0.03 |
|  |  | LR | 0.72 ± 0.01 | 0.73 ± 0.02 | 0.44 ± 0.04 | 0.72 ± 0.02 | 0.72 ± 0.02 | 0.71 ± 0.02 | 0.75 ± 0.02 | 0.73 ± 0.02 | 0.44 ± 0.05 | 0.72 ± 0.02 | 0.72 ± 0.02 | 0.71 ± 0.03 | 0.75 ± 0.04 |
|  |  | NB | 0.68 ± 0.01 | 0.68 ± 0.03 | 0.27 ± 0.06 | 0.63 ± 0.02 | 0.63 ± 0.02 | 0.59 ± 0.02 | 0.8 ± 0.05 | 0.68 ± 0.03 | 0.28 ± 0.05 | 0.63 ± 0.02 | 0.63 ± 0.02 | 0.59 ± 0.02 | 0.8 ± 0.06 |
|  |  | RF | 0.79 ± 0.01 | 0.8 ± 0.02 | 0.6 ± 0.04 | 0.8 ± 0.02 | 0.8 ± 0.02 | 0.8 ± 0.02 | 0.8 ± 0.02 | 0.8 ± 0.02 | 0.6 ± 0.05 | 0.8 ± 0.02 | 0.8 ± 0.02 | 0.8 ± 0.03 | 0.8 ± 0.04 |
|  |  | SVM | 0.78 ± 0.02 | 0.77 ± 0.02 | 0.53 ± 0.04 | 0.77 ± 0.02 | 0.77 ± 0.02 | 0.75 ± 0.02 | 0.8 ± 0.02 | 0.78 ± 0.02 | 0.54 ± 0.04 | 0.77 ± 0.02 | 0.77 ± 0.02 | 0.75 ± 0.02 | 0.8 ± 0.03 |
|  |  |  |  |  |  |  |  |  |  |  |  |  |  |  |  |
| FIX 40 | 75 | AB | 0.63 ± 0.03 | 0.55 ± 0.09 | 0.36 ± 0.07 | 0.66 ± 0.04 | 0.7 ± 0.03 | 0.69 ± 0.04 | 0.46 ± 0.11 | 0.55 ± 0.08 | 0.37 ± 0.07 | 0.67 ± 0.04 | 0.71 ± 0.03 | 0.7 ± 0.05 | 0.46 ± 0.11 |
|  |  | CART | 0.64 ± 0.01 | 0.57 ± 0.06 | 0.32 ± 0.06 | 0.65 ± 0.03 | 0.68 ± 0.03 | 0.61 ± 0.04 | 0.54 ± 0.08 | 0.56 ± 0.04 | 0.31 ± 0.06 | 0.65 ± 0.03 | 0.68 ± 0.03 | 0.61 ± 0.06 | 0.53 ± 0.07 |
|  |  | ET | 0.65 ± 0.03 | 0.6 ± 0.06 | 0.38 ± 0.08 | 0.68 ± 0.04 | 0.71 ± 0.03 | 0.66 ± 0.04 | 0.56 ± 0.08 | 0.6 ± 0.06 | 0.37 ± 0.08 | 0.68 ± 0.04 | 0.71 ± 0.04 | 0.65 ± 0.05 | 0.55 ± 0.07 |
|  |  | GBM | 0.64 ± 0.03 | 0.63 ± 0.04 | 0.43 ± 0.04 | 0.71 ± 0.02 | 0.73 ± 0.02 | 0.69 ± 0.02 | 0.58 ± 0.05 | 0.63 ± 0.04 | 0.42 ± 0.05 | 0.7 ± 0.02 | 0.73 ± 0.02 | 0.69 ± 0.03 | 0.58 ± 0.05 |
|  |  | KNN | 0.7 ± 0.02 | 0.68 ± 0.03 | 0.48 ± 0.04 | 0.74 ± 0.02 | 0.75 ± 0.02 | 0.68 ± 0.02 | 0.69 ± 0.04 | 0.68 ± 0.03 | 0.48 ± 0.04 | 0.74 ± 0.02 | 0.75 ± 0.02 | 0.68 ± 0.02 | 0.69 ± 0.04 |
|  |  | LDA | 0.56 ± 0.04 | 0.48 ± 0.09 | 0.26 ± 0.08 | 0.62 ± 0.04 | 0.66 ± 0.03 | 0.61 ± 0.05 | 0.4 ± 0.11 | 0.48 ± 0.06 | 0.26 ± 0.06 | 0.62 ± 0.03 | 0.66 ± 0.03 | 0.61 ± 0.06 | 0.4 ± 0.07 |
|  |  |  |  | **Validation set** | | | | | | **Test set** | | | | | |
| **Activity threshold** | **PCC** | **Algorithm** | **F1 train** | **F1** | **MCC** | **ROC AUC** | **Accuracy** | **Precision** | **Recall** | **F1** | **MCC** | **ROC AUC** | **Accuracy** | **Precision** | **Recall** |
| FIX 40 | 75 | LR | 0.62 ± 0.03 | 0.46 ± 0.09 | 0.23 ± 0.07 | 0.6 ± 0.04 | 0.65 ± 0.03 | 0.59 ± 0.04 | 0.38 ± 0.1 | 0.45 ± 0.08 | 0.23 ± 0.06 | 0.6 ± 0.03 | 0.64 ± 0.03 | 0.59 ± 0.06 | 0.38 ± 0.09 |
|  |  | NB | 0.59 ± 0.03 | 0.25 ± 0.26 | 0.12 ± 0.14 | 0.56 ± 0.07 | 0.63 ± 0.03 | 0.29 ± 0.29 | 0.22 ± 0.24 | 0.24 ± 0.24 | 0.1 ± 0.11 | 0.55 ± 0.05 | 0.62 ± 0.03 | 0.28 ± 0.28 | 0.21 ± 0.22 |
|  |  | RF | 0.69 ± 0.03 | 0.66 ± 0.04 | 0.47 ± 0.06 | 0.73 ± 0.03 | 0.75 ± 0.03 | 0.72 ± 0.04 | 0.62 ± 0.05 | 0.66 ± 0.04 | 0.47 ± 0.07 | 0.73 ± 0.03 | 0.75 ± 0.03 | 0.72 ± 0.04 | 0.62 ± 0.05 |
|  |  | SVM | 0.7 ± 0.02 | 0.66 ± 0.04 | 0.45 ± 0.04 | 0.72 ± 0.03 | 0.74 ± 0.02 | 0.7 ± 0.02 | 0.62 ± 0.06 | 0.65 ± 0.04 | 0.45 ± 0.04 | 0.72 ± 0.03 | 0.74 ± 0.02 | 0.7 ± 0.02 | 0.62 ± 0.08 |
|  | 85 | AB | 0.65 ± 0.03 | 0.56 ± 0.08 | 0.37 ± 0.07 | 0.67 ± 0.04 | 0.71 ± 0.03 | 0.68 ± 0.03 | 0.49 ± 0.1 | 0.56 ± 0.08 | 0.38 ± 0.06 | 0.67 ± 0.04 | 0.71 ± 0.03 | 0.7 ± 0.04 | 0.48 ± 0.11 |
|  |  | CART | 0.65 ± 0.02 | 0.56 ± 0.05 | 0.32 ± 0.06 | 0.65 ± 0.03 | 0.68 ± 0.03 | 0.62 ± 0.04 | 0.52 ± 0.08 | 0.57 ± 0.04 | 0.33 ± 0.06 | 0.66 ± 0.03 | 0.68 ± 0.03 | 0.62 ± 0.05 | 0.53 ± 0.07 |
|  |  | ET | 0.64 ± 0.03 | 0.6 ± 0.07 | 0.38 ± 0.09 | 0.68 ± 0.05 | 0.71 ± 0.04 | 0.66 ± 0.04 | 0.55 ± 0.09 | 0.59 ± 0.06 | 0.36 ± 0.08 | 0.68 ± 0.04 | 0.7 ± 0.04 | 0.65 ± 0.05 | 0.54 ± 0.08 |
|  |  | GBM | 0.65 ± 0.04 | 0.63 ± 0.04 | 0.42 ± 0.06 | 0.7 ± 0.03 | 0.73 ± 0.03 | 0.68 ± 0.03 | 0.58 ± 0.06 | 0.63 ± 0.04 | 0.42 ± 0.06 | 0.7 ± 0.03 | 0.73 ± 0.03 | 0.69 ± 0.04 | 0.58 ± 0.05 |
|  |  | KNN | 0.7 ± 0.03 | 0.7 ± 0.03 | 0.5 ± 0.04 | 0.75 ± 0.02 | 0.76 ± 0.02 | 0.69 ± 0.02 | 0.7 ± 0.04 | 0.7 ± 0.03 | 0.5 ± 0.04 | 0.75 ± 0.02 | 0.76 ± 0.02 | 0.69 ± 0.02 | 0.7 ± 0.04 |
|  |  | LDA | 0.58 ± 0.03 | 0.51 ± 0.07 | 0.29 ± 0.06 | 0.63 ± 0.03 | 0.67 ± 0.02 | 0.62 ± 0.04 | 0.44 ± 0.08 | 0.5 ± 0.05 | 0.27 ± 0.06 | 0.62 ± 0.03 | 0.66 ± 0.03 | 0.62 ± 0.05 | 0.43 ± 0.06 |
|  |  | LR | 0.63 ± 0.02 | 0.48 ± 0.09 | 0.26 ± 0.08 | 0.62 ± 0.04 | 0.66 ± 0.03 | 0.6 ± 0.05 | 0.42 ± 0.11 | 0.49 ± 0.04 | 0.26 ± 0.05 | 0.62 ± 0.02 | 0.66 ± 0.02 | 0.61 ± 0.04 | 0.42 ± 0.05 |
|  |  | NB | 0.6 ± 0.02 | 0.23 ± 0.27 | 0.11 ± 0.14 | 0.56 ± 0.07 | 0.62 ± 0.03 | 0.24 ± 0.28 | 0.22 ± 0.27 | 0.21 ± 0.25 | 0.08 ± 0.09 | 0.54 ± 0.05 | 0.61 ± 0.01 | 0.21 ± 0.25 | 0.21 ± 0.25 |
|  |  | RF | 0.69 ± 0.03 | 0.68 ± 0.04 | 0.5 ± 0.05 | 0.74 ± 0.03 | 0.76 ± 0.02 | 0.73 ± 0.03 | 0.64 ± 0.05 | 0.68 ± 0.04 | 0.5 ± 0.05 | 0.74 ± 0.03 | 0.76 ± 0.02 | 0.73 ± 0.03 | 0.63 ± 0.05 |
|  |  | SVM | 0.7 ± 0.02 | 0.66 ± 0.03 | 0.46 ± 0.05 | 0.73 ± 0.02 | 0.74 ± 0.02 | 0.7 ± 0.03 | 0.63 ± 0.05 | 0.66 ± 0.03 | 0.46 ± 0.04 | 0.72 ± 0.02 | 0.74 ± 0.02 | 0.7 ± 0.03 | 0.63 ± 0.05 |
|  | 95 | AB | 0.64 ± 0.03 | 0.57 ± 0.06 | 0.38 ± 0.07 | 0.67 ± 0.04 | 0.71 ± 0.03 | 0.69 ± 0.04 | 0.49 ± 0.07 | 0.56 ± 0.05 | 0.37 ± 0.06 | 0.67 ± 0.03 | 0.71 ± 0.02 | 0.7 ± 0.05 | 0.47 ± 0.07 |
|  |  | CART | 0.65 ± 0.02 | 0.62 ± 0.03 | 0.38 ± 0.04 | 0.69 ± 0.02 | 0.7 ± 0.02 | 0.63 ± 0.02 | 0.6 ± 0.06 | 0.62 ± 0.03 | 0.38 ± 0.04 | 0.69 ± 0.02 | 0.7 ± 0.02 | 0.63 ± 0.02 | 0.61 ± 0.06 |
|  |  | ET | 0.64 ± 0.03 | 0.6 ± 0.07 | 0.37 ± 0.08 | 0.68 ± 0.04 | 0.71 ± 0.04 | 0.65 ± 0.04 | 0.55 ± 0.08 | 0.6 ± 0.06 | 0.37 ± 0.08 | 0.68 ± 0.04 | 0.71 ± 0.03 | 0.66 ± 0.05 | 0.55 ± 0.07 |
|  |  | GBM | 0.64 ± 0.04 | 0.63 ± 0.04 | 0.42 ± 0.06 | 0.7 ± 0.03 | 0.73 ± 0.02 | 0.69 ± 0.03 | 0.58 ± 0.04 | 0.63 ± 0.04 | 0.43 ± 0.05 | 0.71 ± 0.03 | 0.73 ± 0.02 | 0.69 ± 0.04 | 0.58 ± 0.04 |
|  |  | KNN | 0.7 ± 0.02 | 0.7 ± 0.03 | 0.5 ± 0.05 | 0.75 ± 0.02 | 0.76 ± 0.02 | 0.69 ± 0.02 | 0.71 ± 0.04 | 0.7 ± 0.03 | 0.5 ± 0.05 | 0.75 ± 0.02 | 0.76 ± 0.02 | 0.69 ± 0.02 | 0.71 ± 0.04 |
|  |  | LDA | 0.6 ± 0.03 | 0.55 ± 0.07 | 0.34 ± 0.08 | 0.66 ± 0.04 | 0.69 ± 0.03 | 0.65 ± 0.04 | 0.48 ± 0.09 | 0.55 ± 0.06 | 0.33 ± 0.07 | 0.65 ± 0.04 | 0.69 ± 0.03 | 0.64 ± 0.04 | 0.49 ± 0.07 |
|  |  | LR | 0.65 ± 0.02 | 0.55 ± 0.06 | 0.33 ± 0.05 | 0.65 ± 0.03 | 0.69 ± 0.02 | 0.64 ± 0.02 | 0.48 ± 0.07 | 0.55 ± 0.04 | 0.32 ± 0.05 | 0.65 ± 0.02 | 0.69 ± 0.02 | 0.64 ± 0.03 | 0.48 ± 0.06 |
|  |  | NB | 0.6 ± 0.02 | 0.23 ± 0.28 | 0.12 ± 0.14 | 0.56 ± 0.07 | 0.62 ± 0.03 | 0.24 ± 0.28 | 0.24 ± 0.29 | 0.22 ± 0.26 | 0.08 ± 0.1 | 0.54 ± 0.05 | 0.61 ± 0.02 | 0.22 ± 0.26 | 0.22 ± 0.26 |

|  |  |  |  | **Validation set** | | | | | | **Test set** | | | | | |
| --- | --- | --- | --- | --- | --- | --- | --- | --- | --- | --- | --- | --- | --- | --- | --- |
| **Activity threshold** | **PCC** | **Algorithm** | **F1 train** | **F1** | **MCC** | **ROC AUC** | **Accuracy** | **Precision** | **Recall** | **F1** | **MCC** | **ROC AUC** | **Accuracy** | **Precision** | **Recall** |
| FIX 40 | 95 | RF | 0.7 ± 0.03 | 0.68 ± 0.04 | 0.5 ± 0.05 | 0.74 ± 0.03 | 0.76 ± 0.02 | 0.73 ± 0.03 | 0.64 ± 0.04 | 0.68 ± 0.04 | 0.49 ± 0.05 | 0.74 ± 0.03 | 0.76 ± 0.02 | 0.73 ± 0.03 | 0.63 ± 0.04 |
|  |  | SVM | 0.71 ± 0.02 | 0.66 ± 0.03 | 0.45 ± 0.04 | 0.72 ± 0.02 | 0.74 ± 0.02 | 0.69 ± 0.02 | 0.63 ± 0.04 | 0.66 ± 0.03 | 0.45 ± 0.04 | 0.72 ± 0.02 | 0.74 ± 0.02 | 0.69 ± 0.03 | 0.64 ± 0.04 |
|  | 100 | AB | 0.64 ± 0.03 | 0.55 ± 0.13 | 0.36 ± 0.09 | 0.66 ± 0.05 | 0.7 ± 0.04 | 0.67 ± 0.03 | 0.48 ± 0.14 | 0.56 ± 0.14 | 0.37 ± 0.09 | 0.67 ± 0.06 | 0.71 ± 0.04 | 0.69 ± 0.03 | 0.5 ± 0.15 |
|  |  | CART | 0.64 ± 0.02 | 0.54 ± 0.06 | 0.29 ± 0.05 | 0.64 ± 0.03 | 0.67 ± 0.02 | 0.6 ± 0.02 | 0.5 ± 0.08 | 0.54 ± 0.04 | 0.29 ± 0.04 | 0.64 ± 0.02 | 0.67 ± 0.02 | 0.6 ± 0.02 | 0.5 ± 0.06 |
|  |  | ET | 0.64 ± 0.03 | 0.6 ± 0.06 | 0.38 ± 0.08 | 0.68 ± 0.04 | 0.71 ± 0.03 | 0.66 ± 0.04 | 0.55 ± 0.08 | 0.6 ± 0.05 | 0.38 ± 0.08 | 0.68 ± 0.04 | 0.71 ± 0.04 | 0.66 ± 0.06 | 0.55 ± 0.06 |
|  |  | GBM | 0.65 ± 0.04 | 0.62 ± 0.05 | 0.42 ± 0.06 | 0.7 ± 0.03 | 0.73 ± 0.02 | 0.68 ± 0.03 | 0.57 ± 0.06 | 0.62 ± 0.04 | 0.41 ± 0.06 | 0.7 ± 0.03 | 0.72 ± 0.03 | 0.68 ± 0.04 | 0.57 ± 0.05 |
|  |  | KNN | 0.69 ± 0.02 | 0.69 ± 0.03 | 0.49 ± 0.05 | 0.74 ± 0.02 | 0.75 ± 0.02 | 0.68 ± 0.02 | 0.7 ± 0.04 | 0.69 ± 0.03 | 0.49 ± 0.05 | 0.74 ± 0.02 | 0.75 ± 0.02 | 0.68 ± 0.02 | 0.7 ± 0.04 |
|  |  | LDA | 0.61 ± 0.02 | 0.55 ± 0.06 | 0.34 ± 0.06 | 0.66 ± 0.03 | 0.69 ± 0.02 | 0.66 ± 0.03 | 0.48 ± 0.07 | 0.55 ± 0.04 | 0.34 ± 0.05 | 0.66 ± 0.02 | 0.69 ± 0.02 | 0.65 ± 0.03 | 0.48 ± 0.06 |
|  |  | LR | 0.65 ± 0.02 | 0.55 ± 0.07 | 0.33 ± 0.07 | 0.65 ± 0.04 | 0.69 ± 0.03 | 0.65 ± 0.04 | 0.48 ± 0.08 | 0.55 ± 0.05 | 0.33 ± 0.06 | 0.65 ± 0.03 | 0.69 ± 0.03 | 0.65 ± 0.04 | 0.48 ± 0.06 |
|  |  | NB | 0.6 ± 0.02 | 0.08 ± 0.19 | 0.04 ± 0.08 | 0.52 ± 0.04 | 0.61 ± 0.01 | 0.09 ± 0.2 | 0.08 ± 0.18 | 0.08 ± 0.19 | 0.03 ± 0.08 | 0.52 ± 0.04 | 0.61 ± 0.01 | 0.09 ± 0.2 | 0.08 ± 0.19 |
|  |  | RF | 0.69 ± 0.03 | 0.68 ± 0.05 | 0.5 ± 0.07 | 0.74 ± 0.04 | 0.76 ± 0.03 | 0.73 ± 0.04 | 0.64 ± 0.06 | 0.68 ± 0.05 | 0.5 ± 0.07 | 0.74 ± 0.04 | 0.76 ± 0.03 | 0.73 ± 0.04 | 0.64 ± 0.06 |
|  |  | SVM | 0.7 ± 0.02 | 0.66 ± 0.04 | 0.46 ± 0.05 | 0.72 ± 0.03 | 0.74 ± 0.02 | 0.69 ± 0.02 | 0.63 ± 0.05 | 0.66 ± 0.03 | 0.45 ± 0.05 | 0.72 ± 0.02 | 0.74 ± 0.02 | 0.69 ± 0.04 | 0.63 ± 0.04 |
|  |  |  |  |  |  |  |  |  |  |  |  |  |  |  |  |
| FIX 45 | 75 | AB | 0.67 ± 0.01 | 0.65 ± 0.03 | 0.41 ± 0.04 | 0.7 ± 0.02 | 0.71 ± 0.02 | 0.7 ± 0.03 | 0.62 ± 0.05 | 0.65 ± 0.03 | 0.41 ± 0.04 | 0.7 ± 0.02 | 0.71 ± 0.02 | 0.7 ± 0.03 | 0.62 ± 0.05 |
|  |  | CART | 0.67 ± 0.02 | 0.64 ± 0.03 | 0.36 ± 0.05 | 0.68 ± 0.02 | 0.68 ± 0.03 | 0.66 ± 0.04 | 0.62 ± 0.05 | 0.63 ± 0.02 | 0.36 ± 0.05 | 0.68 ± 0.02 | 0.68 ± 0.03 | 0.66 ± 0.04 | 0.61 ± 0.04 |
|  |  | ET | 0.67 ± 0.02 | 0.67 ± 0.03 | 0.41 ± 0.04 | 0.7 ± 0.02 | 0.71 ± 0.02 | 0.68 ± 0.02 | 0.65 ± 0.04 | 0.67 ± 0.02 | 0.41 ± 0.03 | 0.7 ± 0.02 | 0.71 ± 0.02 | 0.69 ± 0.02 | 0.65 ± 0.04 |
|  |  | GBM | 0.69 ± 0.01 | 0.68 ± 0.02 | 0.43 ± 0.04 | 0.72 ± 0.02 | 0.72 ± 0.02 | 0.7 ± 0.03 | 0.66 ± 0.03 | 0.68 ± 0.03 | 0.44 ± 0.05 | 0.72 ± 0.02 | 0.72 ± 0.02 | 0.7 ± 0.03 | 0.67 ± 0.04 |
|  |  | KNN | 0.72 ± 0.02 | 0.73 ± 0.02 | 0.51 ± 0.04 | 0.76 ± 0.02 | 0.76 ± 0.02 | 0.72 ± 0.02 | 0.75 ± 0.04 | 0.73 ± 0.02 | 0.51 ± 0.04 | 0.76 ± 0.02 | 0.76 ± 0.02 | 0.72 ± 0.02 | 0.75 ± 0.04 |
|  |  | LDA | 0.62 ± 0.01 | 0.6 ± 0.03 | 0.31 ± 0.04 | 0.65 ± 0.02 | 0.66 ± 0.02 | 0.64 ± 0.02 | 0.57 ± 0.04 | 0.6 ± 0.03 | 0.31 ± 0.04 | 0.65 ± 0.02 | 0.66 ± 0.02 | 0.64 ± 0.02 | 0.56 ± 0.04 |
|  |  | LR | 0.64 ± 0.01 | 0.6 ± 0.03 | 0.31 ± 0.04 | 0.65 ± 0.02 | 0.66 ± 0.02 | 0.64 ± 0.02 | 0.57 ± 0.04 | 0.6 ± 0.03 | 0.31 ± 0.04 | 0.65 ± 0.02 | 0.66 ± 0.02 | 0.64 ± 0.02 | 0.57 ± 0.04 |
|  |  | NB | 0.64 ± 0.01 | 0.61 ± 0.03 | 0.25 ± 0.03 | 0.62 ± 0.02 | 0.62 ± 0.01 | 0.56 ± 0.02 | 0.67 ± 0.09 | 0.61 ± 0.04 | 0.25 ± 0.04 | 0.62 ± 0.02 | 0.62 ± 0.02 | 0.56 ± 0.02 | 0.67 ± 0.09 |
|  |  |  |  | **Validation set** | | | | | | **Test set** | | | | | |
| **Activity threshold** | **PCC** | **Algorithm** | **F1 train** | **F1** | **MCC** | **ROC AUC** | **Accuracy** | **Precision** | **Recall** | **F1** | **MCC** | **ROC AUC** | **Accuracy** | **Precision** | **Recall** |
| FIX 45 | 75 | RF | 0.73 ± 0.01 | 0.74 ± 0.03 | 0.54 ± 0.05 | 0.77 ± 0.02 | 0.77 ± 0.02 | 0.75 ± 0.03 | 0.73 ± 0.03 | 0.74 ± 0.03 | 0.54 ± 0.05 | 0.77 ± 0.02 | 0.77 ± 0.02 | 0.76 ± 0.03 | 0.73 ± 0.04 |
|  |  | SVM | 0.72 ± 0.02 | 0.67 ± 0.2 | 0.46 ± 0.15 | 0.73 ± 0.07 | 0.74 ± 0.06 | 0.67 ± 0.2 | 0.66 ± 0.2 | 0.66 ± 0.2 | 0.46 ± 0.15 | 0.73 ± 0.07 | 0.74 ± 0.06 | 0.67 ± 0.2 | 0.66 ± 0.2 |
|  | 85 | AB | 0.67 ± 0.01 | 0.67 ± 0.02 | 0.42 ± 0.03 | 0.71 ± 0.02 | 0.71 ± 0.02 | 0.69 ± 0.02 | 0.65 ± 0.03 | 0.66 ± 0.03 | 0.42 ± 0.04 | 0.7 ± 0.02 | 0.71 ± 0.02 | 0.69 ± 0.02 | 0.64 ± 0.06 |
|  |  | CART | 0.68 ± 0.01 | 0.62 ± 0.03 | 0.33 ± 0.05 | 0.66 ± 0.03 | 0.67 ± 0.03 | 0.64 ± 0.04 | 0.62 ± 0.06 | 0.62 ± 0.04 | 0.32 ± 0.07 | 0.66 ± 0.03 | 0.66 ± 0.04 | 0.63 ± 0.05 | 0.62 ± 0.07 |
|  |  | ET | 0.68 ± 0.01 | 0.67 ± 0.02 | 0.4 ± 0.03 | 0.7 ± 0.02 | 0.71 ± 0.01 | 0.68 ± 0.02 | 0.66 ± 0.03 | 0.67 ± 0.02 | 0.41 ± 0.03 | 0.7 ± 0.02 | 0.71 ± 0.01 | 0.68 ± 0.02 | 0.66 ± 0.03 |
|  |  | GBM | 0.69 ± 0.01 | 0.69 ± 0.02 | 0.46 ± 0.03 | 0.73 ± 0.02 | 0.73 ± 0.02 | 0.71 ± 0.02 | 0.67 ± 0.03 | 0.69 ± 0.03 | 0.46 ± 0.04 | 0.73 ± 0.02 | 0.74 ± 0.02 | 0.72 ± 0.03 | 0.68 ± 0.05 |
|  |  | KNN | 0.72 ± 0.01 | 0.74 ± 0.02 | 0.52 ± 0.04 | 0.76 ± 0.02 | 0.76 ± 0.02 | 0.73 ± 0.02 | 0.75 ± 0.04 | 0.74 ± 0.02 | 0.52 ± 0.04 | 0.76 ± 0.02 | 0.76 ± 0.02 | 0.73 ± 0.02 | 0.75 ± 0.04 |
|  |  | LDA | 0.62 ± 0.03 | 0.61 ± 0.02 | 0.32 ± 0.03 | 0.66 ± 0.02 | 0.67 ± 0.01 | 0.64 ± 0.02 | 0.58 ± 0.04 | 0.61 ± 0.02 | 0.32 ± 0.03 | 0.66 ± 0.02 | 0.66 ± 0.02 | 0.64 ± 0.02 | 0.58 ± 0.04 |
|  |  | LR | 0.64 ± 0.02 | 0.6 ± 0.03 | 0.31 ± 0.04 | 0.65 ± 0.02 | 0.66 ± 0.02 | 0.63 ± 0.02 | 0.58 ± 0.04 | 0.6 ± 0.03 | 0.31 ± 0.03 | 0.65 ± 0.02 | 0.66 ± 0.02 | 0.63 ± 0.02 | 0.57 ± 0.04 |
|  |  | NB | 0.63 ± 0.0 | 0.51 ± 0.23 | 0.21 ± 0.1 | 0.6 ± 0.05 | 0.6 ± 0.03 | 0.46 ± 0.21 | 0.58 ± 0.27 | 0.51 ± 0.23 | 0.2 ± 0.1 | 0.6 ± 0.05 | 0.6 ± 0.03 | 0.46 ± 0.21 | 0.57 ± 0.26 |
|  |  | RF | 0.74 ± 0.01 | 0.75 ± 0.02 | 0.56 ± 0.04 | 0.78 ± 0.02 | 0.78 ± 0.02 | 0.77 ± 0.02 | 0.74 ± 0.02 | 0.75 ± 0.03 | 0.56 ± 0.05 | 0.78 ± 0.02 | 0.78 ± 0.02 | 0.76 ± 0.03 | 0.74 ± 0.04 |
|  |  | SVM | 0.72 ± 0.02 | 0.71 ± 0.03 | 0.48 ± 0.02 | 0.74 ± 0.02 | 0.74 ± 0.01 | 0.72 ± 0.05 | 0.7 ± 0.07 | 0.7 ± 0.03 | 0.48 ± 0.03 | 0.74 ± 0.02 | 0.74 ± 0.01 | 0.72 ± 0.04 | 0.7 ± 0.08 |
|  | 95 | AB | 0.68 ± 0.01 | 0.65 ± 0.04 | 0.4 ± 0.04 | 0.7 ± 0.02 | 0.7 ± 0.02 | 0.69 ± 0.02 | 0.62 ± 0.07 | 0.65 ± 0.04 | 0.41 ± 0.04 | 0.7 ± 0.02 | 0.71 ± 0.02 | 0.7 ± 0.02 | 0.62 ± 0.08 |
|  |  | CART | 0.67 ± 0.02 | 0.58 ± 0.14 | 0.32 ± 0.08 | 0.66 ± 0.05 | 0.66 ± 0.04 | 0.65 ± 0.04 | 0.56 ± 0.18 | 0.58 ± 0.14 | 0.32 ± 0.08 | 0.66 ± 0.05 | 0.67 ± 0.04 | 0.66 ± 0.04 | 0.56 ± 0.18 |
|  |  | ET | 0.66 ± 0.01 | 0.66 ± 0.02 | 0.4 ± 0.03 | 0.7 ± 0.02 | 0.7 ± 0.02 | 0.68 ± 0.02 | 0.65 ± 0.03 | 0.66 ± 0.02 | 0.4 ± 0.03 | 0.7 ± 0.02 | 0.71 ± 0.02 | 0.68 ± 0.02 | 0.65 ± 0.03 |
|  |  | GBM | 0.69 ± 0.01 | 0.7 ± 0.02 | 0.46 ± 0.04 | 0.73 ± 0.02 | 0.74 ± 0.02 | 0.72 ± 0.02 | 0.68 ± 0.03 | 0.7 ± 0.02 | 0.47 ± 0.04 | 0.73 ± 0.02 | 0.74 ± 0.02 | 0.72 ± 0.02 | 0.68 ± 0.04 |
|  |  | KNN | 0.72 ± 0.01 | 0.74 ± 0.03 | 0.52 ± 0.04 | 0.76 ± 0.02 | 0.76 ± 0.02 | 0.72 ± 0.02 | 0.75 ± 0.04 | 0.74 ± 0.03 | 0.52 ± 0.04 | 0.76 ± 0.02 | 0.76 ± 0.02 | 0.72 ± 0.02 | 0.75 ± 0.04 |
|  |  | LDA | 0.64 ± 0.02 | 0.64 ± 0.03 | 0.37 ± 0.04 | 0.68 ± 0.02 | 0.69 ± 0.02 | 0.66 ± 0.02 | 0.61 ± 0.03 | 0.64 ± 0.02 | 0.37 ± 0.04 | 0.68 ± 0.02 | 0.69 ± 0.02 | 0.66 ± 0.03 | 0.62 ± 0.03 |
|  |  | LR | 0.66 ± 0.02 | 0.64 ± 0.03 | 0.36 ± 0.04 | 0.68 ± 0.02 | 0.69 ± 0.02 | 0.66 ± 0.02 | 0.61 ± 0.04 | 0.63 ± 0.02 | 0.36 ± 0.04 | 0.68 ± 0.02 | 0.68 ± 0.02 | 0.66 ± 0.02 | 0.61 ± 0.03 |
|  |  | NB | 0.63 ± 0.0 | 0.46 ± 0.27 | 0.18 ± 0.11 | 0.59 ± 0.06 | 0.6 ± 0.03 | 0.42 ± 0.24 | 0.53 ± 0.31 | 0.46 ± 0.27 | 0.18 ± 0.11 | 0.59 ± 0.06 | 0.6 ± 0.03 | 0.41 ± 0.24 | 0.52 ± 0.31 |
|  |  | RF | 0.73 ± 0.02 | 0.75 ± 0.02 | 0.55 ± 0.04 | 0.77 ± 0.02 | 0.78 ± 0.02 | 0.76 ± 0.03 | 0.74 ± 0.02 | 0.74 ± 0.03 | 0.55 ± 0.04 | 0.77 ± 0.02 | 0.78 ± 0.02 | 0.76 ± 0.03 | 0.74 ± 0.04 |
|  |  | SVM | 0.72 ± 0.01 | 0.72 ± 0.03 | 0.49 ± 0.03 | 0.74 ± 0.02 | 0.75 ± 0.02 | 0.73 ± 0.04 | 0.71 ± 0.06 | 0.7 ± 0.04 | 0.48 ± 0.04 | 0.74 ± 0.02 | 0.74 ± 0.02 | 0.72 ± 0.03 | 0.69 ± 0.08 |

|  |  |  |  | **Validation set** | | | | | | **Test set** | | | | | |
| --- | --- | --- | --- | --- | --- | --- | --- | --- | --- | --- | --- | --- | --- | --- | --- |
| **Activity threshold** | **PCC** | **Algorithm** | **F1 train** | **F1** | **MCC** | **ROC AUC** | **Accuracy** | **Precision** | **Recall** | **F1** | **MCC** | **ROC AUC** | **Accuracy** | **Precision** | **Recall** |
| FIX 45 | 100 | AB | 0.68 ± 0.01 | 0.66 ± 0.02 | 0.4 ± 0.03 | 0.7 ± 0.02 | 0.7 ± 0.02 | 0.68 ± 0.02 | 0.63 ± 0.03 | 0.66 ± 0.03 | 0.4 ± 0.04 | 0.7 ± 0.02 | 0.71 ± 0.02 | 0.69 ± 0.02 | 0.64 ± 0.05 |
|  |  | CART | 0.67 ± 0.01 | 0.63 ± 0.04 | 0.34 ± 0.06 | 0.67 ± 0.03 | 0.68 ± 0.03 | 0.64 ± 0.03 | 0.62 ± 0.05 | 0.64 ± 0.03 | 0.35 ± 0.05 | 0.67 ± 0.03 | 0.68 ± 0.02 | 0.64 ± 0.03 | 0.63 ± 0.05 |
|  |  | ET | 0.68 ± 0.01 | 0.67 ± 0.02 | 0.42 ± 0.04 | 0.71 ± 0.02 | 0.71 ± 0.02 | 0.69 ± 0.02 | 0.66 ± 0.02 | 0.68 ± 0.02 | 0.42 ± 0.03 | 0.71 ± 0.02 | 0.72 ± 0.02 | 0.69 ± 0.02 | 0.66 ± 0.03 |
|  |  | GBM | 0.69 ± 0.01 | 0.7 ± 0.03 | 0.47 ± 0.04 | 0.73 ± 0.02 | 0.74 ± 0.02 | 0.72 ± 0.02 | 0.68 ± 0.03 | 0.7 ± 0.02 | 0.47 ± 0.04 | 0.74 ± 0.02 | 0.74 ± 0.02 | 0.72 ± 0.02 | 0.68 ± 0.04 |
|  |  | KNN | 0.71 ± 0.02 | 0.73 ± 0.02 | 0.5 ± 0.04 | 0.75 ± 0.02 | 0.75 ± 0.02 | 0.72 ± 0.02 | 0.74 ± 0.04 | 0.73 ± 0.02 | 0.5 ± 0.04 | 0.75 ± 0.02 | 0.75 ± 0.02 | 0.72 ± 0.02 | 0.74 ± 0.04 |
|  |  | LDA | 0.63 ± 0.02 | 0.62 ± 0.05 | 0.36 ± 0.07 | 0.68 ± 0.04 | 0.68 ± 0.04 | 0.67 ± 0.04 | 0.59 ± 0.05 | 0.63 ± 0.04 | 0.36 ± 0.07 | 0.68 ± 0.03 | 0.68 ± 0.03 | 0.67 ± 0.04 | 0.59 ± 0.04 |
|  |  | LR | 0.66 ± 0.01 | 0.63 ± 0.03 | 0.36 ± 0.04 | 0.68 ± 0.02 | 0.69 ± 0.02 | 0.66 ± 0.02 | 0.6 ± 0.04 | 0.63 ± 0.02 | 0.36 ± 0.04 | 0.68 ± 0.02 | 0.69 ± 0.02 | 0.66 ± 0.03 | 0.6 ± 0.03 |
|  |  | NB | 0.63 ± 0.0 | 0.46 ± 0.27 | 0.18 ± 0.1 | 0.59 ± 0.05 | 0.59 ± 0.03 | 0.41 ± 0.24 | 0.55 ± 0.32 | 0.46 ± 0.26 | 0.16 ± 0.1 | 0.58 ± 0.05 | 0.58 ± 0.03 | 0.4 ± 0.23 | 0.54 ± 0.32 |
|  |  | RF | 0.73 ± 0.02 | 0.74 ± 0.02 | 0.54 ± 0.04 | 0.77 ± 0.02 | 0.77 ± 0.02 | 0.75 ± 0.03 | 0.73 ± 0.02 | 0.74 ± 0.03 | 0.54 ± 0.05 | 0.77 ± 0.02 | 0.77 ± 0.02 | 0.75 ± 0.03 | 0.73 ± 0.04 |
|  |  | SVM | 0.72 ± 0.01 | 0.71 ± 0.01 | 0.47 ± 0.02 | 0.74 ± 0.01 | 0.74 ± 0.01 | 0.7 ± 0.03 | 0.72 ± 0.03 | 0.71 ± 0.01 | 0.48 ± 0.02 | 0.74 ± 0.01 | 0.74 ± 0.01 | 0.71 ± 0.03 | 0.72 ± 0.04 |
|  |  |  |  |  |  |  |  |  |  |  |  |  |  |  |  |
| FIX 50 | 75 | AB | 0.71 ± 0.01 | 0.7 ± 0.03 | 0.4 ± 0.05 | 0.7 ± 0.02 | 0.7 ± 0.02 | 0.69 ± 0.02 | 0.72 ± 0.04 | 0.7 ± 0.03 | 0.4 ± 0.05 | 0.7 ± 0.03 | 0.7 ± 0.03 | 0.69 ± 0.04 | 0.72 ± 0.07 |
|  |  | CART | 0.7 ± 0.02 | 0.65 ± 0.02 | 0.31 ± 0.04 | 0.65 ± 0.02 | 0.66 ± 0.02 | 0.65 ± 0.03 | 0.65 ± 0.04 | 0.65 ± 0.03 | 0.31 ± 0.05 | 0.66 ± 0.02 | 0.66 ± 0.02 | 0.65 ± 0.03 | 0.65 ± 0.05 |
|  |  | ET | 0.7 ± 0.02 | 0.7 ± 0.02 | 0.4 ± 0.05 | 0.7 ± 0.02 | 0.7 ± 0.02 | 0.7 ± 0.02 | 0.71 ± 0.03 | 0.7 ± 0.03 | 0.4 ± 0.05 | 0.7 ± 0.02 | 0.7 ± 0.02 | 0.69 ± 0.02 | 0.71 ± 0.04 |
|  |  | GBM | 0.72 ± 0.01 | 0.71 ± 0.02 | 0.42 ± 0.03 | 0.71 ± 0.02 | 0.71 ± 0.02 | 0.7 ± 0.02 | 0.71 ± 0.02 | 0.71 ± 0.02 | 0.42 ± 0.03 | 0.71 ± 0.02 | 0.71 ± 0.02 | 0.7 ± 0.02 | 0.71 ± 0.04 |
|  |  | KNN | 0.74 ± 0.02 | 0.73 ± 0.03 | 0.46 ± 0.06 | 0.73 ± 0.03 | 0.73 ± 0.03 | 0.72 ± 0.03 | 0.74 ± 0.03 | 0.73 ± 0.03 | 0.46 ± 0.06 | 0.73 ± 0.03 | 0.73 ± 0.03 | 0.72 ± 0.03 | 0.74 ± 0.03 |
|  |  | LDA | 0.66 ± 0.01 | 0.66 ± 0.02 | 0.31 ± 0.04 | 0.66 ± 0.02 | 0.66 ± 0.02 | 0.65 ± 0.02 | 0.66 ± 0.02 | 0.65 ± 0.02 | 0.31 ± 0.04 | 0.65 ± 0.02 | 0.65 ± 0.02 | 0.65 ± 0.03 | 0.66 ± 0.05 |
|  |  | LR | 0.67 ± 0.02 | 0.66 ± 0.02 | 0.32 ± 0.04 | 0.66 ± 0.02 | 0.66 ± 0.02 | 0.65 ± 0.02 | 0.68 ± 0.02 | 0.66 ± 0.02 | 0.31 ± 0.04 | 0.66 ± 0.02 | 0.66 ± 0.02 | 0.65 ± 0.03 | 0.67 ± 0.05 |
|  |  | NB | 0.66 ± 0.02 | 0.65 ± 0.02 | 0.22 ± 0.04 | 0.6 ± 0.03 | 0.6 ± 0.03 | 0.58 ± 0.04 | 0.74 ± 0.11 | 0.65 ± 0.03 | 0.23 ± 0.04 | 0.61 ± 0.03 | 0.61 ± 0.03 | 0.59 ± 0.04 | 0.74 ± 0.12 |
|  |  | RF | 0.76 ± 0.01 | 0.74 ± 0.01 | 0.49 ± 0.02 | 0.74 ± 0.01 | 0.74 ± 0.01 | 0.74 ± 0.01 | 0.75 ± 0.02 | 0.74 ± 0.02 | 0.49 ± 0.03 | 0.74 ± 0.01 | 0.74 ± 0.01 | 0.74 ± 0.02 | 0.75 ± 0.04 |
|  |  | SVM | 0.75 ± 0.01 | 0.73 ± 0.02 | 0.46 ± 0.05 | 0.73 ± 0.02 | 0.73 ± 0.02 | 0.72 ± 0.03 | 0.75 ± 0.04 | 0.73 ± 0.02 | 0.46 ± 0.04 | 0.73 ± 0.02 | 0.73 ± 0.02 | 0.72 ± 0.03 | 0.75 ± 0.04 |
|  |  |  |  | **Validation set** | | | | | | **Test set** | | | | | |
| **Activity threshold** | **PCC** | **Algorithm** | **F1 train** | **F1** | **MCC** | **ROC AUC** | **Accuracy** | **Precision** | **Recall** | **F1** | **MCC** | **ROC AUC** | **Accuracy** | **Precision** | **Recall** |
| FIX 50 | 85 | AB | 0.71 ± 0.01 | 0.71 ± 0.02 | 0.4 ± 0.05 | 0.7 ± 0.02 | 0.7 ± 0.02 | 0.69 ± 0.03 | 0.72 ± 0.04 | 0.7 ± 0.03 | 0.4 ± 0.05 | 0.7 ± 0.03 | 0.7 ± 0.03 | 0.69 ± 0.03 | 0.72 ± 0.06 |
|  |  | CART | 0.7 ± 0.01 | 0.67 ± 0.03 | 0.32 ± 0.05 | 0.66 ± 0.02 | 0.66 ± 0.02 | 0.65 ± 0.02 | 0.68 ± 0.05 | 0.66 ± 0.03 | 0.32 ± 0.05 | 0.66 ± 0.02 | 0.66 ± 0.02 | 0.65 ± 0.03 | 0.68 ± 0.07 |
|  |  | ET | 0.7 ± 0.02 | 0.7 ± 0.02 | 0.4 ± 0.04 | 0.7 ± 0.02 | 0.7 ± 0.02 | 0.7 ± 0.02 | 0.71 ± 0.02 | 0.7 ± 0.02 | 0.4 ± 0.04 | 0.7 ± 0.02 | 0.7 ± 0.02 | 0.69 ± 0.02 | 0.71 ± 0.04 |
|  |  | GBM | 0.72 ± 0.01 | 0.72 ± 0.02 | 0.43 ± 0.04 | 0.71 ± 0.02 | 0.71 ± 0.02 | 0.71 ± 0.02 | 0.72 ± 0.03 | 0.72 ± 0.02 | 0.43 ± 0.03 | 0.72 ± 0.02 | 0.72 ± 0.02 | 0.71 ± 0.02 | 0.72 ± 0.05 |
|  |  | KNN | 0.75 ± 0.02 | 0.74 ± 0.03 | 0.47 ± 0.06 | 0.74 ± 0.03 | 0.74 ± 0.03 | 0.73 ± 0.03 | 0.74 ± 0.03 | 0.74 ± 0.03 | 0.47 ± 0.06 | 0.74 ± 0.03 | 0.74 ± 0.03 | 0.73 ± 0.03 | 0.74 ± 0.03 |
|  |  | LDA | 0.66 ± 0.02 | 0.66 ± 0.01 | 0.33 ± 0.03 | 0.66 ± 0.02 | 0.66 ± 0.02 | 0.66 ± 0.02 | 0.67 ± 0.02 | 0.66 ± 0.02 | 0.33 ± 0.04 | 0.66 ± 0.02 | 0.66 ± 0.02 | 0.66 ± 0.03 | 0.67 ± 0.04 |
|  |  | LR | 0.67 ± 0.01 | 0.66 ± 0.02 | 0.32 ± 0.03 | 0.66 ± 0.02 | 0.66 ± 0.02 | 0.66 ± 0.02 | 0.68 ± 0.02 | 0.66 ± 0.02 | 0.32 ± 0.04 | 0.66 ± 0.02 | 0.66 ± 0.02 | 0.65 ± 0.03 | 0.67 ± 0.05 |
|  |  | NB | 0.66 ± 0.01 | 0.65 ± 0.02 | 0.23 ± 0.04 | 0.6 ± 0.03 | 0.6 ± 0.03 | 0.58 ± 0.03 | 0.75 ± 0.1 | 0.64 ± 0.03 | 0.21 ± 0.03 | 0.6 ± 0.02 | 0.6 ± 0.02 | 0.58 ± 0.03 | 0.74 ± 0.11 |
|  |  | RF | 0.76 ± 0.01 | 0.75 ± 0.02 | 0.5 ± 0.04 | 0.75 ± 0.02 | 0.75 ± 0.02 | 0.74 ± 0.02 | 0.76 ± 0.02 | 0.75 ± 0.02 | 0.5 ± 0.04 | 0.75 ± 0.02 | 0.75 ± 0.02 | 0.75 ± 0.02 | 0.76 ± 0.04 |
|  |  | SVM | 0.75 ± 0.01 | 0.72 ± 0.02 | 0.44 ± 0.04 | 0.72 ± 0.02 | 0.72 ± 0.02 | 0.7 ± 0.02 | 0.76 ± 0.02 | 0.72 ± 0.02 | 0.43 ± 0.03 | 0.72 ± 0.02 | 0.71 ± 0.02 | 0.7 ± 0.02 | 0.75 ± 0.04 |
|  | 95 | AB | 0.7 ± 0.01 | 0.7 ± 0.02 | 0.39 ± 0.04 | 0.7 ± 0.02 | 0.69 ± 0.02 | 0.68 ± 0.03 | 0.74 ± 0.05 | 0.7 ± 0.03 | 0.39 ± 0.04 | 0.69 ± 0.02 | 0.69 ± 0.02 | 0.68 ± 0.03 | 0.73 ± 0.07 |
|  |  | CART | 0.7 ± 0.0 | 0.68 ± 0.04 | 0.35 ± 0.07 | 0.68 ± 0.03 | 0.68 ± 0.03 | 0.67 ± 0.03 | 0.68 ± 0.06 | 0.67 ± 0.05 | 0.35 ± 0.08 | 0.67 ± 0.04 | 0.67 ± 0.04 | 0.67 ± 0.04 | 0.68 ± 0.07 |
|  |  | ET | 0.7 ± 0.01 | 0.7 ± 0.02 | 0.4 ± 0.04 | 0.7 ± 0.02 | 0.7 ± 0.02 | 0.7 ± 0.02 | 0.71 ± 0.03 | 0.7 ± 0.02 | 0.4 ± 0.04 | 0.7 ± 0.02 | 0.7 ± 0.02 | 0.7 ± 0.02 | 0.7 ± 0.04 |
|  |  | GBM | 0.72 ± 0.01 | 0.71 ± 0.02 | 0.42 ± 0.04 | 0.71 ± 0.02 | 0.71 ± 0.02 | 0.7 ± 0.02 | 0.72 ± 0.02 | 0.71 ± 0.02 | 0.43 ± 0.03 | 0.71 ± 0.02 | 0.71 ± 0.02 | 0.71 ± 0.02 | 0.72 ± 0.05 |
|  |  | KNN | 0.75 ± 0.02 | 0.74 ± 0.02 | 0.47 ± 0.05 | 0.74 ± 0.02 | 0.74 ± 0.02 | 0.73 ± 0.03 | 0.75 ± 0.03 | 0.74 ± 0.02 | 0.47 ± 0.05 | 0.74 ± 0.02 | 0.74 ± 0.02 | 0.73 ± 0.03 | 0.75 ± 0.03 |
|  |  | LDA | 0.67 ± 0.01 | 0.68 ± 0.02 | 0.35 ± 0.04 | 0.68 ± 0.02 | 0.68 ± 0.02 | 0.67 ± 0.02 | 0.68 ± 0.03 | 0.67 ± 0.03 | 0.35 ± 0.05 | 0.67 ± 0.02 | 0.67 ± 0.02 | 0.67 ± 0.02 | 0.68 ± 0.04 |
|  |  | LR | 0.69 ± 0.01 | 0.68 ± 0.02 | 0.36 ± 0.04 | 0.68 ± 0.02 | 0.68 ± 0.02 | 0.67 ± 0.02 | 0.69 ± 0.02 | 0.68 ± 0.02 | 0.35 ± 0.04 | 0.68 ± 0.02 | 0.68 ± 0.02 | 0.67 ± 0.02 | 0.69 ± 0.04 |
|  |  | NB | 0.66 ± 0.01 | 0.65 ± 0.02 | 0.21 ± 0.04 | 0.6 ± 0.02 | 0.6 ± 0.02 | 0.57 ± 0.02 | 0.75 ± 0.09 | 0.65 ± 0.03 | 0.21 ± 0.04 | 0.6 ± 0.02 | 0.6 ± 0.02 | 0.57 ± 0.02 | 0.75 ± 0.09 |
|  |  | RF | 0.76 ± 0.01 | 0.75 ± 0.02 | 0.49 ± 0.03 | 0.75 ± 0.02 | 0.75 ± 0.02 | 0.74 ± 0.02 | 0.76 ± 0.02 | 0.74 ± 0.02 | 0.49 ± 0.03 | 0.74 ± 0.02 | 0.74 ± 0.02 | 0.74 ± 0.02 | 0.75 ± 0.03 |
|  |  | SVM | 0.75 ± 0.01 | 0.73 ± 0.02 | 0.44 ± 0.04 | 0.72 ± 0.02 | 0.72 ± 0.02 | 0.7 ± 0.02 | 0.75 ± 0.03 | 0.73 ± 0.02 | 0.44 ± 0.04 | 0.72 ± 0.02 | 0.72 ± 0.02 | 0.7 ± 0.02 | 0.76 ± 0.03 |
|  | 100 | AB | 0.7 ± 0.01 | 0.71 ± 0.02 | 0.42 ± 0.05 | 0.71 ± 0.02 | 0.71 ± 0.02 | 0.69 ± 0.03 | 0.74 ± 0.03 | 0.71 ± 0.02 | 0.41 ± 0.05 | 0.7 ± 0.03 | 0.7 ± 0.03 | 0.69 ± 0.04 | 0.74 ± 0.04 |
|  |  | CART | 0.7 ± 0.01 | 0.66 ± 0.04 | 0.32 ± 0.07 | 0.66 ± 0.03 | 0.66 ± 0.03 | 0.66 ± 0.03 | 0.66 ± 0.05 | 0.66 ± 0.04 | 0.32 ± 0.07 | 0.66 ± 0.03 | 0.66 ± 0.03 | 0.66 ± 0.04 | 0.66 ± 0.06 |

|  |  |  |  | **Validation set** | | | | | | **Test set** | | | | | |
| --- | --- | --- | --- | --- | --- | --- | --- | --- | --- | --- | --- | --- | --- | --- | --- |
| **Activity threshold** | **PCC** | **Algorithm** | **F1 train** | **F1** | **MCC** | **ROC AUC** | **Accuracy** | **Precision** | **Recall** | **F1** | **MCC** | **ROC AUC** | **Accuracy** | **Precision** | **Recall** |
| FIX 50 | 100 | ET | 0.7 ± 0.02 | 0.71 ± 0.01 | 0.41 ± 0.03 | 0.71 ± 0.01 | 0.71 ± 0.01 | 0.7 ± 0.01 | 0.72 ± 0.02 | 0.7 ± 0.02 | 0.41 ± 0.03 | 0.7 ± 0.02 | 0.7 ± 0.02 | 0.7 ± 0.02 | 0.71 ± 0.04 |
|  |  | GBM | 0.72 ± 0.01 | 0.71 ± 0.01 | 0.42 ± 0.03 | 0.71 ± 0.02 | 0.71 ± 0.02 | 0.7 ± 0.02 | 0.71 ± 0.02 | 0.7 ± 0.02 | 0.4 ± 0.03 | 0.7 ± 0.02 | 0.7 ± 0.02 | 0.7 ± 0.02 | 0.7 ± 0.04 |
|  |  | KNN | 0.74 ± 0.02 | 0.73 ± 0.02 | 0.45 ± 0.05 | 0.72 ± 0.03 | 0.72 ± 0.03 | 0.71 ± 0.03 | 0.75 ± 0.04 | 0.73 ± 0.02 | 0.45 ± 0.05 | 0.72 ± 0.03 | 0.72 ± 0.03 | 0.71 ± 0.03 | 0.75 ± 0.04 |
|  |  | LDA | 0.67 ± 0.03 | 0.68 ± 0.02 | 0.36 ± 0.04 | 0.68 ± 0.02 | 0.68 ± 0.02 | 0.68 ± 0.02 | 0.69 ± 0.03 | 0.68 ± 0.02 | 0.36 ± 0.04 | 0.68 ± 0.02 | 0.68 ± 0.02 | 0.68 ± 0.02 | 0.68 ± 0.03 |
|  |  | LR | 0.69 ± 0.02 | 0.68 ± 0.02 | 0.35 ± 0.04 | 0.68 ± 0.02 | 0.68 ± 0.02 | 0.67 ± 0.02 | 0.69 ± 0.03 | 0.68 ± 0.02 | 0.36 ± 0.04 | 0.68 ± 0.02 | 0.68 ± 0.02 | 0.67 ± 0.02 | 0.69 ± 0.03 |
|  |  | NB | 0.66 ± 0.02 | 0.64 ± 0.03 | 0.18 ± 0.04 | 0.58 ± 0.02 | 0.58 ± 0.02 | 0.56 ± 0.02 | 0.76 ± 0.1 | 0.64 ± 0.03 | 0.18 ± 0.03 | 0.58 ± 0.02 | 0.58 ± 0.02 | 0.56 ± 0.02 | 0.76 ± 0.1 |
|  |  | RF | 0.75 ± 0.01 | 0.75 ± 0.02 | 0.5 ± 0.03 | 0.75 ± 0.01 | 0.75 ± 0.01 | 0.74 ± 0.01 | 0.76 ± 0.02 | 0.75 ± 0.01 | 0.49 ± 0.02 | 0.75 ± 0.01 | 0.75 ± 0.01 | 0.74 ± 0.02 | 0.75 ± 0.03 |
|  |  | SVM | 0.75 ± 0.02 | 0.72 ± 0.02 | 0.44 ± 0.04 | 0.72 ± 0.02 | 0.72 ± 0.02 | 0.71 ± 0.03 | 0.75 ± 0.06 | 0.72 ± 0.02 | 0.44 ± 0.04 | 0.72 ± 0.02 | 0.72 ± 0.02 | 0.71 ± 0.03 | 0.74 ± 0.06 |
|  |  |  |  |  |  |  |  |  |  |  |  |  |  |  |  |
| FIX 55 | 75 | AB | 0.74 ± 0.01 | 0.73 ± 0.02 | 0.36 ± 0.04 | 0.67 ± 0.02 | 0.68 ± 0.02 | 0.68 ± 0.02 | 0.79 ± 0.02 | 0.73 ± 0.02 | 0.37 ± 0.04 | 0.68 ± 0.02 | 0.69 ± 0.02 | 0.69 ± 0.02 | 0.79 ± 0.04 |
|  |  | CART | 0.71 ± 0.01 | 0.69 ± 0.03 | 0.28 ± 0.06 | 0.64 ± 0.03 | 0.65 ± 0.03 | 0.67 ± 0.04 | 0.71 ± 0.05 | 0.68 ± 0.03 | 0.28 ± 0.06 | 0.64 ± 0.03 | 0.64 ± 0.03 | 0.67 ± 0.03 | 0.7 ± 0.06 |
|  |  | ET | 0.72 ± 0.02 | 0.73 ± 0.02 | 0.37 ± 0.04 | 0.68 ± 0.02 | 0.69 ± 0.02 | 0.7 ± 0.02 | 0.77 ± 0.02 | 0.74 ± 0.02 | 0.38 ± 0.04 | 0.69 ± 0.02 | 0.7 ± 0.02 | 0.7 ± 0.02 | 0.78 ± 0.02 |
|  |  | GBM | 0.76 ± 0.01 | 0.74 ± 0.02 | 0.41 ± 0.04 | 0.7 ± 0.02 | 0.71 ± 0.02 | 0.72 ± 0.02 | 0.77 ± 0.02 | 0.74 ± 0.02 | 0.41 ± 0.04 | 0.7 ± 0.02 | 0.71 ± 0.02 | 0.72 ± 0.02 | 0.78 ± 0.04 |
|  |  | KNN | 0.76 ± 0.02 | 0.75 ± 0.02 | 0.45 ± 0.04 | 0.73 ± 0.02 | 0.73 ± 0.02 | 0.75 ± 0.02 | 0.76 ± 0.03 | 0.75 ± 0.02 | 0.45 ± 0.04 | 0.73 ± 0.02 | 0.73 ± 0.02 | 0.75 ± 0.02 | 0.76 ± 0.03 |
|  |  | LDA | 0.7 ± 0.02 | 0.72 ± 0.02 | 0.32 ± 0.04 | 0.66 ± 0.02 | 0.67 ± 0.02 | 0.67 ± 0.02 | 0.77 ± 0.02 | 0.71 ± 0.02 | 0.32 ± 0.04 | 0.66 ± 0.02 | 0.67 ± 0.02 | 0.67 ± 0.02 | 0.76 ± 0.04 |
|  |  | LR | 0.69 ± 0.02 | 0.7 ± 0.02 | 0.29 ± 0.05 | 0.64 ± 0.02 | 0.65 ± 0.02 | 0.66 ± 0.02 | 0.76 ± 0.03 | 0.71 ± 0.02 | 0.3 ± 0.04 | 0.65 ± 0.02 | 0.66 ± 0.02 | 0.66 ± 0.02 | 0.77 ± 0.05 |
|  |  | NB | 0.71 ± 0.01 | 0.7 ± 0.01 | 0.21 ± 0.04 | 0.59 ± 0.03 | 0.62 ± 0.02 | 0.61 ± 0.02 | 0.82 ± 0.06 | 0.7 ± 0.01 | 0.21 ± 0.04 | 0.59 ± 0.03 | 0.61 ± 0.02 | 0.61 ± 0.03 | 0.82 ± 0.06 |
|  |  | RF | 0.79 ± 0.01 | 0.77 ± 0.02 | 0.48 ± 0.04 | 0.74 ± 0.02 | 0.74 ± 0.02 | 0.75 ± 0.02 | 0.8 ± 0.02 | 0.77 ± 0.02 | 0.48 ± 0.04 | 0.74 ± 0.02 | 0.74 ± 0.02 | 0.75 ± 0.02 | 0.8 ± 0.03 |
|  |  | SVM | 0.77 ± 0.01 | 0.75 ± 0.02 | 0.4 ± 0.05 | 0.69 ± 0.03 | 0.7 ± 0.02 | 0.69 ± 0.03 | 0.82 ± 0.05 | 0.75 ± 0.02 | 0.41 ± 0.06 | 0.69 ± 0.03 | 0.71 ± 0.03 | 0.7 ± 0.03 | 0.83 ± 0.05 |
|  | 85 | AB | 0.74 ± 0.01 | 0.74 ± 0.01 | 0.38 ± 0.04 | 0.68 ± 0.02 | 0.7 ± 0.02 | 0.69 ± 0.02 | 0.8 ± 0.02 | 0.74 ± 0.02 | 0.37 ± 0.05 | 0.68 ± 0.02 | 0.69 ± 0.02 | 0.69 ± 0.02 | 0.79 ± 0.04 |
|  |  | CART | 0.72 ± 0.01 | 0.7 ± 0.02 | 0.33 ± 0.03 | 0.66 ± 0.02 | 0.67 ± 0.02 | 0.69 ± 0.02 | 0.72 ± 0.04 | 0.7 ± 0.02 | 0.33 ± 0.04 | 0.66 ± 0.02 | 0.67 ± 0.02 | 0.69 ± 0.02 | 0.72 ± 0.05 |
|  |  |  |  | **Validation set** | | | | | | **Test set** | | | | | |
| **Activity threshold** | **PCC** | **Algorithm** | **F1 train** | **F1** | **MCC** | **ROC AUC** | **Accuracy** | **Precision** | **Recall** | **F1** | **MCC** | **ROC AUC** | **Accuracy** | **Precision** | **Recall** |
| FIX 55 | 85 | ET | 0.72 ± 0.02 | 0.73 ± 0.02 | 0.37 ± 0.04 | 0.68 ± 0.02 | 0.69 ± 0.02 | 0.69 ± 0.02 | 0.78 ± 0.02 | 0.74 ± 0.02 | 0.38 ± 0.04 | 0.68 ± 0.02 | 0.69 ± 0.02 | 0.7 ± 0.02 | 0.78 ± 0.02 |
|  |  | GBM | 0.76 ± 0.02 | 0.74 ± 0.02 | 0.39 ± 0.04 | 0.69 ± 0.02 | 0.7 ± 0.02 | 0.71 ± 0.02 | 0.77 ± 0.02 | 0.74 ± 0.02 | 0.4 ± 0.03 | 0.7 ± 0.02 | 0.7 ± 0.02 | 0.71 ± 0.02 | 0.77 ± 0.03 |
|  |  | KNN | 0.76 ± 0.02 | 0.75 ± 0.02 | 0.45 ± 0.06 | 0.72 ± 0.03 | 0.73 ± 0.03 | 0.75 ± 0.03 | 0.76 ± 0.02 | 0.75 ± 0.02 | 0.45 ± 0.06 | 0.72 ± 0.03 | 0.73 ± 0.03 | 0.75 ± 0.03 | 0.76 ± 0.02 |
|  |  | LDA | 0.7 ± 0.02 | 0.72 ± 0.02 | 0.34 ± 0.04 | 0.66 ± 0.02 | 0.68 ± 0.02 | 0.68 ± 0.02 | 0.78 ± 0.02 | 0.72 ± 0.02 | 0.34 ± 0.04 | 0.66 ± 0.02 | 0.67 ± 0.02 | 0.68 ± 0.02 | 0.77 ± 0.04 |
|  |  | LR | 0.7 ± 0.02 | 0.72 ± 0.02 | 0.32 ± 0.05 | 0.65 ± 0.02 | 0.66 ± 0.02 | 0.66 ± 0.02 | 0.78 ± 0.01 | 0.71 ± 0.02 | 0.31 ± 0.05 | 0.65 ± 0.02 | 0.66 ± 0.02 | 0.66 ± 0.02 | 0.77 ± 0.04 |
|  |  | NB | 0.7 ± 0.02 | 0.7 ± 0.02 | 0.2 ± 0.06 | 0.59 ± 0.03 | 0.61 ± 0.03 | 0.6 ± 0.02 | 0.84 ± 0.05 | 0.7 ± 0.02 | 0.2 ± 0.05 | 0.58 ± 0.03 | 0.61 ± 0.02 | 0.6 ± 0.02 | 0.84 ± 0.05 |
|  |  | RF | 0.78 ± 0.01 | 0.78 ± 0.02 | 0.49 ± 0.04 | 0.74 ± 0.02 | 0.75 ± 0.02 | 0.75 ± 0.02 | 0.8 ± 0.02 | 0.78 ± 0.02 | 0.49 ± 0.04 | 0.74 ± 0.02 | 0.75 ± 0.02 | 0.75 ± 0.02 | 0.8 ± 0.03 |
|  |  | SVM | 0.77 ± 0.01 | 0.76 ± 0.02 | 0.42 ± 0.05 | 0.7 ± 0.03 | 0.71 ± 0.03 | 0.69 ± 0.03 | 0.85 ± 0.03 | 0.76 ± 0.02 | 0.41 ± 0.05 | 0.69 ± 0.03 | 0.71 ± 0.03 | 0.69 ± 0.03 | 0.84 ± 0.03 |
|  | 95 | AB | 0.74 ± 0.01 | 0.73 ± 0.02 | 0.36 ± 0.06 | 0.67 ± 0.03 | 0.68 ± 0.03 | 0.68 ± 0.03 | 0.8 ± 0.02 | 0.74 ± 0.03 | 0.37 ± 0.06 | 0.68 ± 0.03 | 0.69 ± 0.03 | 0.68 ± 0.02 | 0.8 ± 0.04 |
|  |  | CART | 0.72 ± 0.01 | 0.7 ± 0.03 | 0.29 ± 0.06 | 0.64 ± 0.03 | 0.65 ± 0.03 | 0.67 ± 0.04 | 0.73 ± 0.08 | 0.69 ± 0.04 | 0.29 ± 0.07 | 0.64 ± 0.03 | 0.65 ± 0.03 | 0.66 ± 0.04 | 0.73 ± 0.1 |
|  |  | ET | 0.72 ± 0.02 | 0.73 ± 0.02 | 0.37 ± 0.05 | 0.68 ± 0.02 | 0.69 ± 0.02 | 0.69 ± 0.02 | 0.77 ± 0.02 | 0.73 ± 0.02 | 0.37 ± 0.05 | 0.68 ± 0.02 | 0.69 ± 0.02 | 0.69 ± 0.02 | 0.78 ± 0.03 |
|  |  | GBM | 0.76 ± 0.01 | 0.74 ± 0.02 | 0.4 ± 0.06 | 0.7 ± 0.03 | 0.71 ± 0.03 | 0.71 ± 0.03 | 0.78 ± 0.02 | 0.74 ± 0.03 | 0.4 ± 0.07 | 0.7 ± 0.03 | 0.7 ± 0.03 | 0.71 ± 0.03 | 0.78 ± 0.04 |
|  |  | KNN | 0.76 ± 0.02 | 0.75 ± 0.03 | 0.45 ± 0.06 | 0.73 ± 0.03 | 0.73 ± 0.03 | 0.75 ± 0.03 | 0.76 ± 0.03 | 0.75 ± 0.03 | 0.45 ± 0.06 | 0.73 ± 0.03 | 0.73 ± 0.03 | 0.75 ± 0.03 | 0.76 ± 0.03 |
|  |  | LDA | 0.72 ± 0.02 | 0.73 ± 0.02 | 0.36 ± 0.06 | 0.68 ± 0.03 | 0.69 ± 0.03 | 0.69 ± 0.03 | 0.78 ± 0.02 | 0.73 ± 0.03 | 0.36 ± 0.06 | 0.68 ± 0.03 | 0.68 ± 0.03 | 0.69 ± 0.02 | 0.78 ± 0.03 |
|  |  | LR | 0.71 ± 0.03 | 0.73 ± 0.02 | 0.36 ± 0.06 | 0.67 ± 0.03 | 0.68 ± 0.03 | 0.69 ± 0.02 | 0.77 ± 0.03 | 0.72 ± 0.03 | 0.35 ± 0.06 | 0.67 ± 0.03 | 0.68 ± 0.03 | 0.68 ± 0.02 | 0.77 ± 0.04 |
|  |  | NB | 0.7 ± 0.02 | 0.7 ± 0.02 | 0.21 ± 0.06 | 0.59 ± 0.03 | 0.61 ± 0.02 | 0.61 ± 0.03 | 0.83 ± 0.06 | 0.7 ± 0.02 | 0.2 ± 0.05 | 0.59 ± 0.03 | 0.61 ± 0.02 | 0.61 ± 0.02 | 0.82 ± 0.06 |
|  |  | RF | 0.78 ± 0.01 | 0.78 ± 0.02 | 0.49 ± 0.04 | 0.74 ± 0.02 | 0.75 ± 0.02 | 0.75 ± 0.02 | 0.8 ± 0.01 | 0.78 ± 0.02 | 0.49 ± 0.04 | 0.74 ± 0.02 | 0.75 ± 0.02 | 0.75 ± 0.02 | 0.8 ± 0.03 |
|  |  | SVM | 0.77 ± 0.01 | 0.77 ± 0.02 | 0.44 ± 0.05 | 0.71 ± 0.03 | 0.72 ± 0.03 | 0.71 ± 0.03 | 0.84 ± 0.04 | 0.76 ± 0.02 | 0.42 ± 0.05 | 0.7 ± 0.03 | 0.71 ± 0.02 | 0.7 ± 0.03 | 0.84 ± 0.04 |
|  | 100 | AB | 0.74 ± 0.01 | 0.74 ± 0.02 | 0.35 ± 0.09 | 0.66 ± 0.05 | 0.68 ± 0.04 | 0.67 ± 0.05 | 0.83 ± 0.04 | 0.74 ± 0.03 | 0.35 ± 0.08 | 0.66 ± 0.04 | 0.68 ± 0.04 | 0.67 ± 0.04 | 0.83 ± 0.05 |
|  |  | CART | 0.72 ± 0.01 | 0.7 ± 0.03 | 0.3 ± 0.08 | 0.64 ± 0.04 | 0.65 ± 0.04 | 0.67 ± 0.05 | 0.75 ± 0.09 | 0.7 ± 0.02 | 0.3 ± 0.07 | 0.64 ± 0.04 | 0.65 ± 0.03 | 0.67 ± 0.05 | 0.75 ± 0.08 |
|  |  | ET | 0.72 ± 0.02 | 0.74 ± 0.02 | 0.38 ± 0.04 | 0.69 ± 0.02 | 0.7 ± 0.02 | 0.7 ± 0.02 | 0.77 ± 0.02 | 0.73 ± 0.02 | 0.38 ± 0.04 | 0.69 ± 0.02 | 0.69 ± 0.02 | 0.7 ± 0.02 | 0.77 ± 0.03 |
|  |  | GBM | 0.76 ± 0.01 | 0.75 ± 0.02 | 0.42 ± 0.04 | 0.71 ± 0.02 | 0.71 ± 0.02 | 0.72 ± 0.02 | 0.78 ± 0.02 | 0.74 ± 0.02 | 0.42 ± 0.04 | 0.7 ± 0.02 | 0.71 ± 0.02 | 0.72 ± 0.02 | 0.77 ± 0.03 |
|  |  |  |  | **Validation set** | | | | | | **Test set** | | | | | |
| **Activity threshold** | **PCC** | **Algorithm** | **F1 train** | **F1** | **MCC** | **ROC AUC** | **Accuracy** | **Precision** | **Recall** | **F1** | **MCC** | **ROC AUC** | **Accuracy** | **Precision** | **Recall** |
| FIX 55 | 100 | KNN | 0.76 ± 0.02 | 0.75 ± 0.02 | 0.44 ± 0.05 | 0.72 ± 0.02 | 0.72 ± 0.02 | 0.74 ± 0.03 | 0.77 ± 0.02 | 0.75 ± 0.02 | 0.44 ± 0.05 | 0.72 ± 0.02 | 0.72 ± 0.02 | 0.74 ± 0.03 | 0.77 ± 0.02 |
|  |  | LDA | 0.71 ± 0.02 | 0.72 ± 0.02 | 0.35 ± 0.05 | 0.67 ± 0.02 | 0.68 ± 0.02 | 0.68 ± 0.02 | 0.77 ± 0.03 | 0.72 ± 0.02 | 0.35 ± 0.05 | 0.67 ± 0.02 | 0.68 ± 0.02 | 0.68 ± 0.02 | 0.77 ± 0.03 |
|  |  | LR | 0.71 ± 0.02 | 0.72 ± 0.02 | 0.33 ± 0.06 | 0.66 ± 0.03 | 0.67 ± 0.03 | 0.68 ± 0.03 | 0.77 ± 0.03 | 0.72 ± 0.02 | 0.33 ± 0.06 | 0.66 ± 0.03 | 0.67 ± 0.03 | 0.68 ± 0.02 | 0.77 ± 0.04 |
|  |  | NB | 0.7 ± 0.03 | 0.7 ± 0.02 | 0.21 ± 0.05 | 0.59 ± 0.03 | 0.61 ± 0.02 | 0.61 ± 0.02 | 0.83 ± 0.07 | 0.7 ± 0.03 | 0.21 ± 0.06 | 0.59 ± 0.03 | 0.61 ± 0.02 | 0.61 ± 0.02 | 0.83 ± 0.07 |
|  |  | RF | 0.78 ± 0.01 | 0.77 ± 0.01 | 0.48 ± 0.04 | 0.74 ± 0.02 | 0.74 ± 0.02 | 0.75 ± 0.02 | 0.8 ± 0.01 | 0.77 ± 0.02 | 0.48 ± 0.04 | 0.74 ± 0.02 | 0.74 ± 0.02 | 0.75 ± 0.02 | 0.8 ± 0.02 |
|  |  | SVM | 0.76 ± 0.01 | 0.76 ± 0.02 | 0.42 ± 0.06 | 0.7 ± 0.03 | 0.71 ± 0.03 | 0.7 ± 0.03 | 0.83 ± 0.04 | 0.76 ± 0.03 | 0.42 ± 0.06 | 0.7 ± 0.03 | 0.71 ± 0.03 | 0.7 ± 0.03 | 0.83 ± 0.05 |
|  |  |  |  |  |  |  |  |  |  |  |  |  |  |  |  |
| FIX 60 | 75 | AB | 0.77 ± 0.01 | 0.78 ± 0.02 | 0.37 ± 0.06 | 0.67 ± 0.03 | 0.71 ± 0.03 | 0.71 ± 0.02 | 0.86 ± 0.03 | 0.77 ± 0.02 | 0.36 ± 0.06 | 0.66 ± 0.03 | 0.7 ± 0.03 | 0.71 ± 0.03 | 0.86 ± 0.02 |
|  |  | CART | 0.74 ± 0.01 | 0.74 ± 0.02 | 0.32 ± 0.05 | 0.65 ± 0.03 | 0.68 ± 0.02 | 0.71 ± 0.02 | 0.78 ± 0.05 | 0.74 ± 0.02 | 0.32 ± 0.05 | 0.65 ± 0.03 | 0.68 ± 0.02 | 0.71 ± 0.03 | 0.78 ± 0.05 |
|  |  | ET | 0.75 ± 0.01 | 0.78 ± 0.01 | 0.39 ± 0.04 | 0.68 ± 0.02 | 0.72 ± 0.02 | 0.72 ± 0.02 | 0.84 ± 0.02 | 0.78 ± 0.01 | 0.4 ± 0.04 | 0.69 ± 0.02 | 0.72 ± 0.02 | 0.73 ± 0.02 | 0.85 ± 0.02 |
|  |  | GBM | 0.79 ± 0.01 | 0.78 ± 0.01 | 0.41 ± 0.05 | 0.7 ± 0.03 | 0.72 ± 0.02 | 0.74 ± 0.02 | 0.84 ± 0.02 | 0.78 ± 0.02 | 0.41 ± 0.05 | 0.7 ± 0.03 | 0.72 ± 0.02 | 0.74 ± 0.02 | 0.84 ± 0.03 |
|  |  | KNN | 0.79 ± 0.01 | 0.8 ± 0.02 | 0.5 ± 0.04 | 0.75 ± 0.02 | 0.76 ± 0.02 | 0.79 ± 0.02 | 0.82 ± 0.03 | 0.8 ± 0.02 | 0.5 ± 0.04 | 0.75 ± 0.02 | 0.76 ± 0.02 | 0.79 ± 0.02 | 0.82 ± 0.03 |
|  |  | LDA | 0.75 ± 0.01 | 0.76 ± 0.01 | 0.33 ± 0.04 | 0.65 ± 0.02 | 0.69 ± 0.02 | 0.7 ± 0.02 | 0.85 ± 0.02 | 0.76 ± 0.02 | 0.33 ± 0.04 | 0.65 ± 0.02 | 0.69 ± 0.02 | 0.7 ± 0.01 | 0.85 ± 0.03 |
|  |  | LR | 0.72 ± 0.01 | 0.76 ± 0.01 | 0.27 ± 0.12 | 0.62 ± 0.06 | 0.67 ± 0.04 | 0.67 ± 0.04 | 0.88 ± 0.06 | 0.76 ± 0.02 | 0.27 ± 0.13 | 0.62 ± 0.06 | 0.67 ± 0.04 | 0.68 ± 0.04 | 0.88 ± 0.06 |
|  |  | NB | 0.74 ± 0.01 | 0.75 ± 0.01 | 0.23 ± 0.06 | 0.6 ± 0.03 | 0.65 ± 0.02 | 0.66 ± 0.02 | 0.88 ± 0.03 | 0.75 ± 0.01 | 0.23 ± 0.06 | 0.59 ± 0.03 | 0.65 ± 0.02 | 0.65 ± 0.02 | 0.88 ± 0.03 |
|  |  | RF | 0.82 ± 0.01 | 0.82 ± 0.01 | 0.52 ± 0.04 | 0.75 ± 0.02 | 0.77 ± 0.02 | 0.78 ± 0.02 | 0.86 ± 0.02 | 0.82 ± 0.01 | 0.52 ± 0.03 | 0.75 ± 0.02 | 0.77 ± 0.01 | 0.78 ± 0.01 | 0.86 ± 0.02 |
|  |  | SVM | 0.81 ± 0.01 | 0.81 ± 0.02 | 0.49 ± 0.04 | 0.73 ± 0.02 | 0.76 ± 0.02 | 0.76 ± 0.02 | 0.87 ± 0.03 | 0.81 ± 0.02 | 0.48 ± 0.05 | 0.73 ± 0.02 | 0.76 ± 0.02 | 0.76 ± 0.02 | 0.87 ± 0.02 |
|  | 85 | AB | 0.78 ± 0.01 | 0.78 ± 0.02 | 0.39 ± 0.06 | 0.68 ± 0.03 | 0.72 ± 0.02 | 0.72 ± 0.02 | 0.86 ± 0.02 | 0.78 ± 0.02 | 0.39 ± 0.05 | 0.68 ± 0.02 | 0.71 ± 0.02 | 0.72 ± 0.02 | 0.86 ± 0.02 |
|  |  | CART | 0.76 ± 0.01 | 0.76 ± 0.02 | 0.34 ± 0.08 | 0.66 ± 0.04 | 0.69 ± 0.03 | 0.71 ± 0.04 | 0.82 ± 0.04 | 0.76 ± 0.02 | 0.34 ± 0.08 | 0.66 ± 0.04 | 0.69 ± 0.03 | 0.71 ± 0.03 | 0.82 ± 0.04 |
|  |  | ET | 0.75 ± 0.01 | 0.78 ± 0.01 | 0.4 ± 0.04 | 0.69 ± 0.02 | 0.72 ± 0.02 | 0.73 ± 0.02 | 0.85 ± 0.01 | 0.78 ± 0.02 | 0.4 ± 0.04 | 0.69 ± 0.02 | 0.72 ± 0.02 | 0.73 ± 0.01 | 0.85 ± 0.03 |
|  |  | GBM | 0.79 ± 0.01 | 0.8 ± 0.02 | 0.45 ± 0.04 | 0.72 ± 0.02 | 0.74 ± 0.02 | 0.76 ± 0.02 | 0.84 ± 0.02 | 0.8 ± 0.01 | 0.46 ± 0.04 | 0.72 ± 0.02 | 0.74 ± 0.02 | 0.76 ± 0.02 | 0.84 ± 0.02 |

|  |  |  |  | **Validation set** | | | | | | **Test set** | | | | | |
| --- | --- | --- | --- | --- | --- | --- | --- | --- | --- | --- | --- | --- | --- | --- | --- |
| **Activity threshold** | **PCC** | **Algorithm** | **F1 train** | **F1** | **MCC** | **ROC AUC** | **Accuracy** | **Precision** | **Recall** | **F1** | **MCC** | **ROC AUC** | **Accuracy** | **Precision** | **Recall** |
| FIX 60 | 85 | KNN | 0.79 ± 0.01 | 0.8 ± 0.02 | 0.5 ± 0.06 | 0.75 ± 0.03 | 0.76 ± 0.03 | 0.79 ± 0.03 | 0.82 ± 0.03 | 0.8 ± 0.02 | 0.5 ± 0.06 | 0.75 ± 0.03 | 0.76 ± 0.03 | 0.79 ± 0.03 | 0.82 ± 0.03 |
|  |  | LDA | 0.75 ± 0.01 | 0.76 ± 0.01 | 0.34 ± 0.03 | 0.66 ± 0.02 | 0.69 ± 0.02 | 0.7 ± 0.01 | 0.84 ± 0.02 | 0.76 ± 0.01 | 0.34 ± 0.03 | 0.66 ± 0.02 | 0.69 ± 0.02 | 0.7 ± 0.01 | 0.84 ± 0.02 |
|  |  | LR | 0.73 ± 0.01 | 0.76 ± 0.01 | 0.26 ± 0.12 | 0.62 ± 0.06 | 0.66 ± 0.04 | 0.67 ± 0.04 | 0.87 ± 0.06 | 0.76 ± 0.02 | 0.26 ± 0.12 | 0.62 ± 0.06 | 0.66 ± 0.04 | 0.67 ± 0.04 | 0.87 ± 0.06 |
|  |  | NB | 0.75 ± 0.01 | 0.74 ± 0.02 | 0.22 ± 0.08 | 0.59 ± 0.04 | 0.64 ± 0.03 | 0.65 ± 0.03 | 0.86 ± 0.03 | 0.75 ± 0.02 | 0.22 ± 0.08 | 0.59 ± 0.04 | 0.65 ± 0.03 | 0.66 ± 0.03 | 0.87 ± 0.03 |
|  |  | RF | 0.82 ± 0.01 | 0.82 ± 0.01 | 0.53 ± 0.03 | 0.76 ± 0.02 | 0.78 ± 0.02 | 0.79 ± 0.02 | 0.86 ± 0.02 | 0.82 ± 0.01 | 0.53 ± 0.03 | 0.76 ± 0.02 | 0.78 ± 0.02 | 0.79 ± 0.02 | 0.86 ± 0.02 |
|  |  | SVM | 0.81 ± 0.01 | 0.82 ± 0.01 | 0.51 ± 0.04 | 0.74 ± 0.02 | 0.77 ± 0.02 | 0.77 ± 0.02 | 0.88 ± 0.02 | 0.82 ± 0.01 | 0.5 ± 0.04 | 0.74 ± 0.02 | 0.76 ± 0.02 | 0.77 ± 0.02 | 0.88 ± 0.02 |
|  | 95 | AB | 0.78 ± 0.01 | 0.79 ± 0.02 | 0.41 ± 0.05 | 0.69 ± 0.03 | 0.72 ± 0.02 | 0.72 ± 0.02 | 0.87 ± 0.02 | 0.79 ± 0.02 | 0.4 ± 0.06 | 0.69 ± 0.03 | 0.72 ± 0.02 | 0.72 ± 0.02 | 0.86 ± 0.02 |
|  |  | CART | 0.75 ± 0.01 | 0.74 ± 0.02 | 0.32 ± 0.08 | 0.65 ± 0.04 | 0.68 ± 0.03 | 0.71 ± 0.04 | 0.79 ± 0.04 | 0.74 ± 0.02 | 0.32 ± 0.08 | 0.65 ± 0.04 | 0.68 ± 0.03 | 0.71 ± 0.04 | 0.79 ± 0.04 |
|  |  | ET | 0.75 ± 0.02 | 0.78 ± 0.01 | 0.4 ± 0.04 | 0.69 ± 0.02 | 0.72 ± 0.02 | 0.73 ± 0.02 | 0.84 ± 0.01 | 0.78 ± 0.02 | 0.4 ± 0.04 | 0.69 ± 0.02 | 0.72 ± 0.02 | 0.73 ± 0.02 | 0.84 ± 0.02 |
|  |  | GBM | 0.79 ± 0.01 | 0.79 ± 0.02 | 0.44 ± 0.05 | 0.71 ± 0.02 | 0.74 ± 0.02 | 0.75 ± 0.02 | 0.84 ± 0.02 | 0.79 ± 0.02 | 0.44 ± 0.05 | 0.71 ± 0.03 | 0.74 ± 0.02 | 0.75 ± 0.02 | 0.84 ± 0.02 |
|  |  | KNN | 0.8 ± 0.01 | 0.81 ± 0.02 | 0.52 ± 0.04 | 0.76 ± 0.02 | 0.77 ± 0.02 | 0.8 ± 0.02 | 0.82 ± 0.02 | 0.81 ± 0.02 | 0.52 ± 0.04 | 0.76 ± 0.02 | 0.77 ± 0.02 | 0.8 ± 0.02 | 0.82 ± 0.02 |
|  |  | LDA | 0.75 ± 0.01 | 0.77 ± 0.02 | 0.36 ± 0.05 | 0.67 ± 0.02 | 0.7 ± 0.02 | 0.71 ± 0.02 | 0.84 ± 0.02 | 0.77 ± 0.02 | 0.37 ± 0.05 | 0.67 ± 0.02 | 0.7 ± 0.02 | 0.71 ± 0.02 | 0.84 ± 0.03 |
|  |  | LR | 0.73 ± 0.01 | 0.77 ± 0.02 | 0.29 ± 0.14 | 0.64 ± 0.07 | 0.68 ± 0.04 | 0.69 ± 0.05 | 0.87 ± 0.06 | 0.77 ± 0.02 | 0.3 ± 0.14 | 0.64 ± 0.07 | 0.68 ± 0.04 | 0.69 ± 0.04 | 0.87 ± 0.06 |
|  |  | NB | 0.74 ± 0.01 | 0.74 ± 0.01 | 0.2 ± 0.06 | 0.58 ± 0.03 | 0.64 ± 0.02 | 0.65 ± 0.02 | 0.87 ± 0.02 | 0.74 ± 0.01 | 0.21 ± 0.05 | 0.58 ± 0.02 | 0.64 ± 0.02 | 0.65 ± 0.02 | 0.87 ± 0.03 |
|  |  | RF | 0.82 ± 0.01 | 0.82 ± 0.02 | 0.51 ± 0.04 | 0.75 ± 0.02 | 0.77 ± 0.02 | 0.78 ± 0.02 | 0.86 ± 0.02 | 0.82 ± 0.02 | 0.51 ± 0.04 | 0.75 ± 0.02 | 0.77 ± 0.02 | 0.78 ± 0.02 | 0.85 ± 0.02 |
|  |  | SVM | 0.81 ± 0.01 | 0.81 ± 0.01 | 0.49 ± 0.04 | 0.73 ± 0.02 | 0.76 ± 0.02 | 0.76 ± 0.02 | 0.88 ± 0.02 | 0.81 ± 0.01 | 0.49 ± 0.04 | 0.73 ± 0.02 | 0.76 ± 0.02 | 0.76 ± 0.02 | 0.88 ± 0.02 |
|  | 100 | AB | 0.78 ± 0.01 | 0.79 ± 0.02 | 0.41 ± 0.06 | 0.69 ± 0.03 | 0.72 ± 0.03 | 0.72 ± 0.02 | 0.87 ± 0.02 | 0.79 ± 0.02 | 0.4 ± 0.06 | 0.68 ± 0.03 | 0.72 ± 0.02 | 0.72 ± 0.02 | 0.86 ± 0.02 |
|  |  | CART | 0.75 ± 0.02 | 0.76 ± 0.02 | 0.38 ± 0.04 | 0.68 ± 0.02 | 0.7 ± 0.02 | 0.74 ± 0.02 | 0.78 ± 0.02 | 0.76 ± 0.02 | 0.38 ± 0.04 | 0.68 ± 0.02 | 0.7 ± 0.02 | 0.74 ± 0.02 | 0.78 ± 0.02 |
|  |  | ET | 0.75 ± 0.01 | 0.78 ± 0.02 | 0.39 ± 0.06 | 0.68 ± 0.03 | 0.71 ± 0.03 | 0.73 ± 0.02 | 0.84 ± 0.02 | 0.78 ± 0.02 | 0.39 ± 0.05 | 0.69 ± 0.02 | 0.72 ± 0.02 | 0.73 ± 0.02 | 0.84 ± 0.02 |
|  |  | GBM | 0.8 ± 0.01 | 0.79 ± 0.02 | 0.43 ± 0.04 | 0.71 ± 0.02 | 0.73 ± 0.02 | 0.74 ± 0.02 | 0.84 ± 0.02 | 0.79 ± 0.02 | 0.43 ± 0.04 | 0.7 ± 0.02 | 0.73 ± 0.02 | 0.74 ± 0.02 | 0.83 ± 0.03 |
|  |  | KNN | 0.79 ± 0.01 | 0.8 ± 0.01 | 0.49 ± 0.04 | 0.74 ± 0.02 | 0.76 ± 0.02 | 0.78 ± 0.02 | 0.83 ± 0.02 | 0.8 ± 0.01 | 0.49 ± 0.04 | 0.74 ± 0.02 | 0.76 ± 0.02 | 0.78 ± 0.02 | 0.83 ± 0.02 |
|  |  | LDA | 0.75 ± 0.01 | 0.77 ± 0.02 | 0.36 ± 0.04 | 0.67 ± 0.02 | 0.7 ± 0.02 | 0.71 ± 0.01 | 0.85 ± 0.03 | 0.77 ± 0.02 | 0.37 ± 0.04 | 0.67 ± 0.02 | 0.7 ± 0.02 | 0.71 ± 0.01 | 0.85 ± 0.03 |

|  |  |  |  | **Validation set** | | | | | | **Test set** | | | | | |
| --- | --- | --- | --- | --- | --- | --- | --- | --- | --- | --- | --- | --- | --- | --- | --- |
| **Activity threshold** | **PCC** | **Algorithm** | **F1 train** | **F1** | **MCC** | **ROC AUC** | **Accuracy** | **Precision** | **Recall** | **F1** | **MCC** | **ROC AUC** | **Accuracy** | **Precision** | **Recall** |
| FIX 60 | 100 | LR | 0.73 ± 0.01 | 0.76 ± 0.01 | 0.28 ± 0.13 | 0.63 ± 0.06 | 0.67 ± 0.04 | 0.68 ± 0.04 | 0.87 ± 0.06 | 0.76 ± 0.02 | 0.28 ± 0.14 | 0.63 ± 0.06 | 0.68 ± 0.04 | 0.68 ± 0.04 | 0.87 ± 0.06 |
|  |  | NB | 0.74 ± 0.01 | 0.74 ± 0.01 | 0.2 ± 0.05 | 0.58 ± 0.02 | 0.64 ± 0.02 | 0.65 ± 0.02 | 0.87 ± 0.02 | 0.74 ± 0.01 | 0.21 ± 0.04 | 0.58 ± 0.02 | 0.64 ± 0.02 | 0.65 ± 0.01 | 0.88 ± 0.02 |
|  |  | RF | 0.82 ± 0.01 | 0.81 ± 0.02 | 0.5 ± 0.04 | 0.74 ± 0.02 | 0.76 ± 0.02 | 0.78 ± 0.02 | 0.85 ± 0.02 | 0.81 ± 0.02 | 0.5 ± 0.04 | 0.74 ± 0.02 | 0.76 ± 0.02 | 0.78 ± 0.02 | 0.85 ± 0.02 |
|  |  | SVM | 0.8 ± 0.01 | 0.81 ± 0.01 | 0.47 ± 0.04 | 0.72 ± 0.02 | 0.75 ± 0.02 | 0.74 ± 0.02 | 0.88 ± 0.02 | 0.81 ± 0.02 | 0.47 ± 0.04 | 0.72 ± 0.02 | 0.75 ± 0.02 | 0.75 ± 0.02 | 0.88 ± 0.02 |

**Table S4**. Molecular targets identified by activity annotations of the ligands in the curated PC-3 and DU-145 datasets. The number of active ligands per target (IC_50_ ≤ 1000 nM) is also reported, while targets with associated effects on PCa development and progression, according to the UniProt and TTD data, are highlighted in bold.

| **Target ChEMBL ID** | **Target name** | ***N* PC-3 compounds** | ***N* DU-145 compounds** |
| --- | --- | --- | --- |
| CHEMBL1865 | Histone deacetylase 6 | 74 | 2 |
| **CHEMBL325** | **Histone deacetylase 1** | 71 | 3 |
| CHEMBL2093865 | Histone deacetylase | 40 | 1 |
| CHEMBL1937 | Histone deacetylase 2 | 39 | 3 |
| CHEMBL3192 | Histone deacetylase 8 | 27 | 1 |
| CHEMBL2971 | Tyrosine-protein kinase JAK2 | 27 |  |
| CHEMBL3717 | Hepatocyte growth factor receptor | 24 |  |
| **CHEMBL2842** | **Serine/threonine-protein kinase mTOR** | 21 |  |
| CHEMBL279 | Vascular endothelial growth factor receptor 2 | 19 |  |
| CHEMBL1829 | Histone deacetylase 3 | 18 | 3 |
| CHEMBL2111429 | Histone deacetylase (HDAC1 and HDAC2) | 17 | 1 |
| CHEMBL203 | Epidermal growth factor receptor erbB1 | 16 | 3 |
| **CHEMBL1977** | **Vitamin D receptor** | 14 |  |
| CHEMBL2095182 | Tubulin | 10 | 5 |
| CHEMBL2111367 | PI3-kinase p110-alpha/p85-alpha | 10 |  |
| CHEMBL3038469 | CDK2/Cyclin A | 7 | 4 |
| CHEMBL2111363 | Histone deacetylase 3/Nuclear receptor corepressor 2 (HDAC3/NCoR2) | 7 | 2 |
| **CHEMBL1913** | **Platelet-derived growth factor receptor beta** | 6 | 1 |
| **CHEMBL287** | **Sigma opioid receptor** | 6 | 5 |
| **CHEMBL3267** | **PI3-kinase p110-gamma subunit** | 6 |  |
| CHEMBL5103 | Histone deacetylase 10 | 6 | 2 |
| **CHEMBL4005** | **PI3-kinase p110-alpha subunit** | 6 |  |
| CHEMBL3553 | Tyrosine-protein kinase TYK2 | 5 |  |
| CHEMBL2111389 | CDK9/cyclin T1 | 5 | 4 |
| CHEMBL202 | Dihydrofolate reductase | 5 |  |
| **Target ChEMBL ID** | **Target name** | ***N* PC-3 compounds** | ***N* DU-145 compounds** |
| CHEMBL2973 | Rho-associated protein kinase 2 | 5 |  |
| CHEMBL1907600 | Cyclin-dependent kinase 5/CDK5 activator 1 | 5 | 4 |
| **CHEMBL1936** | **Stem cell growth factor receptor** | 4 | 1 |
| **CHEMBL262** | **Glycogen synthase kinase-3 beta** | 4 | 1 |
| CHEMBL299 | Protein kinase C alpha | 4 |  |
| CHEMBL3310 | Histone deacetylase 11 | 4 | 3 |
| CHEMBL2148 | Tyrosine-protein kinase JAK3 | 4 | 1 |
| CHEMBL1974 | Tyrosine-protein kinase receptor FLT3 | 4 |  |
| CHEMBL2835 | Tyrosine-protein kinase JAK1 | 4 |  |
| **CHEMBL3130** | **PI3-kinase p110-delta subunit** | 3 |  |
| CHEMBL4699 | Isoprenylcysteine carboxyl methyltransferase | 3 |  |
| CHEMBL260 | MAP kinase p38 alpha | 3 |  |
| CHEMBL3038510 | PI3K p110 beta/p85 alpha | 3 |  |
| **CHEMBL4282** | **Serine/threonine-protein kinase AKT** | 3 |  |
| CHEMBL4040 | MAP kinase ERK2 | 3 |  |
| CHEMBL3385 | MAP kinase ERK1 | 3 |  |
| CHEMBL390 | PC-3 | 3 | 1 |
| CHEMBL308 | Cyclin-dependent kinase 1 | 3 | 2 |
| CHEMBL3650 | Fibroblast growth factor receptor 1 | 3 | 1 |
| CHEMBL4105838 | DCN1-like protein 1 | 3 |  |
| CHEMBL6136 | Lysine-specific histone demethylase 1 | 3 | 2 |
| **CHEMBL1955** | **Vascular endothelial growth factor receptor 3** | 3 |  |
| CHEMBL1868 | Vascular endothelial growth factor receptor 1 | 3 |  |
| **CHEMBL1844** | **Macrophage colony stimulating factor receptor** | 3 | 1 |
| CHEMBL1915 | Tubulin beta-1 chain | 3 | 1 |
| **Target ChEMBL ID** | **Target name** | ***N* PC-3 compounds** | ***N* DU-145 compounds** |
| CHEMBL2111288 | Cyclin-dependent kinase 7/ cyclin H | 2 | 3 |
| **CHEMBL2597** | **Tubulin beta-3 chain** | 2 | 2 |
| CHEMBL2094255 | DNA topoisomerase II | 2 | 1 |
| CHEMBL4523988 | Class 1 histone deacetylase | 2 | 1 |
| CHEMBL4261 | Hypoxia-inducible factor 1 alpha | 2 | 1 |
| **CHEMBL2916** | **Telomerase reverse transcriptase** | 2 |  |
| CHEMBL301 | Cyclin-dependent kinase 2 | 2 | 2 |
| CHEMBL3038484 | Histone deacetylase 3/NCoR1 | 2 | 2 |
| CHEMBL1781 | DNA topoisomerase I | 2 | 2 |
| **CHEMBL1163125** | **Bromodomain-containing protein 4** | 2 | 1 |
| CHEMBL3920 | Protein kinase C theta | 2 |  |
| **CHEMBL3145** | **PI3-kinase p110-beta subunit** | 2 |  |
| **CHEMBL331** | **Cyclin-dependent kinase 4** | 2 | 2 |
| CHEMBL1862 | Tyrosine-protein kinase ABL | 2 | 1 |
| CHEMBL3524 | Histone deacetylase 4 | 2 | 1 |
| CHEMBL2508 | Cyclin-dependent kinase 6 | 2 | 2 |
| CHEMBL4302 | P-glycoprotein 1 | 2 | 2 |
| CHEMBL3430881 | PI3-kinase subunit gamma/Phosphoinositide 3-kinase regulatory subunit 5 | 2 |  |
| CHEMBL2095188 | Glycogen synthase kinase-3 | 2 |  |
| CHEMBL2111432 | PI3-kinase p110-delta/p85-alpha | 2 |  |
| CHEMBL240 | HERG | 2 | 2 |
| CHEMBL2378 | Dual specificity phosphatase Cdc25C | 2 |  |
| CHEMBL2345 | Ribosomal protein S6 kinase alpha 3 | 2 | 1 |
| **CHEMBL5427** | **Homeodomain-interacting protein kinase 1** | 2 |  |
| CHEMBL5719 | Cell division protein kinase 8 | 1 |  |
| CHEMBL4071 | Cathepsin G | 1 |  |
| CHEMBL5543 | Dual specificity tyrosine-phosphorylation-regulated kinase 1B | 1 |  |
| CHEMBL4036 | Cyclin-dependent kinase 5 | 1 | 1 |
| CHEMBL5554 | Phosphatidylinositol-4-phosphate 3-kinase C2 domain-containing beta polypeptide | 1 |  |
| **Target ChEMBL ID** | **Target name** | ***N* PC-3 compounds** | ***N* DU-145 compounds** |
| CHEMBL402 | HMG-CoA reductase | 1 |  |
| CHEMBL1806 | DNA topoisomerase II alpha | 1 | 1 |
| CHEMBL5620 | Proteasome subunit beta type-8 | 1 |  |
| **CHEMBL1824** | **Receptor protein-tyrosine kinase erbB-2** | 1 | 1 |
| CHEMBL5749 | Mitogen-activated protein kinase kinase kinase kinase 1 | 1 |  |
| **CHEMBL3906** | **Ribosomal protein S6 kinase alpha 2** | 1 |  |
| CHEMBL3831201 | 20S proteasome | 1 |  |
| CHEMBL3775 | Dual specificity phosphatase Cdc25A | 1 |  |
| CHEMBL5963 | Proteinase-activated receptor 2 | 1 | 1 |
| **CHEMBL5976** | **Heat shock protein beta-1** | 1 | 1 |
| CHEMBL6002 | Cell division cycle 2-like protein kinase 6 | 1 |  |
| CHEMBL3617 | G1/S-specific cyclin E1 | 1 |  |
| CHEMBL1744525 | Nicotinamide phosphoribosyltransferase | 1 |  |
| CHEMBL4804 | Dual specificity phosphatase Cdc25B | 1 |  |
| CHEMBL4106152 | CDK2/Cyclin O | 1 | 1 |
| CHEMBL1169596 | Serine/threonine-protein kinase A-Raf | 1 |  |
| CHEMBL5122 | Discoidin domain-containing receptor 2 | 1 | 1 |
| CHEMBL4662 | Proteasome Macropain subunit MB1 | 1 |  |
| **CHEMBL4577** | **Homeodomain-interacting protein kinase 3** | 1 |  |
| CHEMBL4576 | Homeodomain-interacting protein kinase 2 | 1 |  |
| CHEMBL4575 | Dual-specificity tyrosine-phosphorylation regulated kinase 3 | 1 |  |
| CHEMBL5145 | Serine/threonine-protein kinase B-raf | 1 | 1 |
| CHEMBL4501 | Ribosomal protein S6 kinase 1 | 1 |  |
| **CHEMBL4462** | **NAD-dependent deacetylase sirtuin 2** | 1 |  |
| CHEMBL4376 | Dual-specificity tyrosine-phosphorylation regulated kinase 2 | 1 |  |
| **CHEMBL3522** | **Cytochrome P450 17A1** | 1 |  |
| CHEMBL1293199 | Serine/threonine-protein kinase VRK1 | 1 |  |
| CHEMBL5147 | Ephrin type-B receptor 4 | 1 |  |
| **Target ChEMBL ID** | **Target name** | ***N* PC-3 compounds** | ***N* DU-145 compounds** |
| CHEMBL4237 | Ribosomal protein S6 kinase alpha 5 | 1 |  |
| CHEMBL4224 | Dual specificty protein kinase CLK1 | 1 |  |
| CHEMBL4208 | Proteasome component C5 | 1 |  |
| CHEMBL4204 | MAP kinase signal-integrating kinase 2 | 1 |  |
| CHEMBL4147 | CaM kinase II alpha | 1 |  |
| CHEMBL4145 | Histone deacetylase 9 | 1 | 1 |
| CHEMBL5285 | Mitogen-activated protein kinase kinase kinase 5 | 1 |  |
| CHEMBL4128 | Tyrosine-protein kinase TIE-2 | 1 |  |
| CHEMBL1907602 | Cyclin-dependent kinase 1/cyclin B1 | 1 | 3 |
| CHEMBL3492 | Proteasome Macropain subunit | 1 |  |
| CHEMBL2793 | Casein kinase I alpha | 1 |  |
| CHEMBL2716 | Histone deacetylase 7 | 1 | 1 |
| **CHEMBL267** | **Tyrosine-protein kinase SRC** | 1 | 1 |
| CHEMBL2664 | Adenosylhomocysteinase | 1 |  |
| CHEMBL2094128 | Cyclin-dependent kinase 2/cyclin A | 1 | 2 |
| CHEMBL2582 | Cyclin A2 | 1 |  |
| CHEMBL258 | Tyrosine-protein kinase LCK | 1 | 1 |
| CHEMBL2563 | Histone deacetylase 5 | 1 | 1 |
| CHEMBL2422 | G1/S-specific cyclin D3 | 1 |  |
| **CHEMBL242** | **Estrogen receptor beta** | 1 |  |
| CHEMBL2412 | G2/mitotic-specific cyclin B1 | 1 |  |
| CHEMBL2409 | Epoxide hydratase | 1 |  |
| CHEMBL2364701 | 26S proteasome | 1 |  |
| CHEMBL2363049 | Epidermal growth factor receptor | 1 |  |
| CHEMBL230 | Cyclooxygenase-2 | 1 | 2 |
| CHEMBL2292 | Dual-specificity tyrosine-phosphorylation regulated kinase 1A | 1 |  |
| CHEMBL2221345 | Hypoxia inducible factors; HIF-1-alpha, HIF-2-alpha | 1 |  |
| CHEMBL221 | Cyclooxygenase-1 | 1 | 1 |
| CHEMBL220 | Acetylcholinesterase | 1 |  |
| **CHEMBL2185** | **Serine/threonine-protein kinase Aurora-B** | 1 |  |
| CHEMBL215 | Arachidonate 5-lipoxygenase | 1 | 1 |
| **Target ChEMBL ID** | **Target name** | ***N* PC-3 compounds** | ***N* DU-145 compounds** |
| CHEMBL2094258 | Nuclear factor NF-kappa-B complex | 1 |  |
| CHEMBL2111455 | CDK6/cyclin D1 | 1 | 2 |
| CHEMBL2111448 | CDK6/cyclin D3 | 1 | 2 |
| CHEMBL2094127 | Cyclin-dependent kinase 1/cyclin B | 1 | 1 |
| CHEMBL2093867 | Protein kinase C (PKC) | 1 |  |
| **CHEMBL340** | **Cytochrome P450 3A4** | 1 |  |
| **CHEMBL206** | **Estrogen receptor alpha** | 1 |  |
| **CHEMBL3396** | **DNA topoisomerase II beta** | 1 | 1 |
| CHEMBL1906 | Serine/threonine-protein kinase RAF | 1 | 3 |
| CHEMBL3317334 | Proteasome subunit beta type-10 | 1 |  |
| CHEMBL1907601 | Cyclin-dependent kinase 4/cyclin D1 | 1 | 2 |
| CHEMBL2108 | Cyclin T1 | 1 |  |
| CHEMBL1907605 | Cyclin-dependent kinase 2/cyclin E1 | 1 | 2 |
| CHEMBL3234 | Tyrosine-protein kinase HCK | 1 |  |
| CHEMBL1907611 | Tumour suppressor p53/oncoprotein Mdm2 | 1 |  |
| CHEMBL3116 | Cyclin-dependent kinase 9 | 1 | 3 |
| CHEMBL1944495 | Proteasome subunit beta type-9 | 1 |  |
| CHEMBL1954 | Ribonucleoside-diphosphate reductase M2 chain | 1 |  |
| CHEMBL3038483 | Histone deacetylase 1/3/5/8 | 1 | 1 |
| CHEMBL3038475 | CDK9/Cyclin K | 1 | 1 |
| CHEMBL3038474 | CDK8/Cyclin C | 1 |  |
| CHEMBL3038472 | CDK4/Cyclin D3 | 1 | 2 |
| CHEMBL3038468 | CDK1/Cyclin E | 1 | 1 |
| CHEMBL3038467 | CDK1/Cyclin A | 1 | 1 |
| CHEMBL3009 | Receptor protein-tyrosine kinase erbB-4 | 1 |  |
| **CHEMBL2007** | **Platelet-derived growth factor receptor alpha** | 1 | 1 |
| **CHEMBL2034** | **Glucocorticoid receptor** | 1 |  |
| **CHEMBL2041** | **Tyrosine-protein kinase receptor RET** | 1 | 1 |
| CHEMBL1075167 | Homeodomain-interacting protein kinase 4 | 1 |  |
| CHEMBL2850 | Glycogen synthase kinase-3 alpha | 1 |  |
| **Target ChEMBL ID** | **Target name** | ***N* PC-3 compounds** | ***N* DU-145 compounds** |
| CHEMBL2850 | Glycogen synthase kinase-3 alpha | 1 |  |
| **CHEMBL1075163** | **Serine/threonine-protein kinase haspin** | 1 |  |
| CHEMBL4641 | Voltage-gated T-type calcium channel alpha-1G subunit |  | 7 |
| CHEMBL2002 | Inosine-5'-monophosphate dehydrogenase 2 |  | 5 |
| **CHEMBL3880** | **Heat shock protein HSP 90-alpha** |  | 4 |
| CHEMBL1075138 | Tyrosyl-DNA phosphodiesterase 1 |  | 2 |
| CHEMBL5319 | Epithelial discoidin domain-containing receptor 1 |  | 2 |
| CHEMBL3055 | Cyclin-dependent kinase 7 |  | 1 |
| CHEMBL3622 | Cytochrome P450 2C19 |  | 1 |
| CHEMBL3301386 | CDK6/cyclin D2 |  | 1 |
| **CHEMBL3290** | **Ephrin type-B receptor 2** |  | 1 |
| CHEMBL1075132 | Heat shock protein 75 kDa, mitochondrial |  | 1 |
| CHEMBL4367 | Tyrosine-protein kinase TXK |  | 1 |
| CHEMBL5251 | Tyrosine-protein kinase BTK |  | 1 |
| **CHEMBL5014** | **Serine/threonine-protein kinase RIPK2** |  | 1 |
| **CHEMBL4685** | **Indoleamine 2,3-dioxygenase** |  | 1 |
| **CHEMBL4601** | **Tyrosine-protein kinase BRK** |  | 1 |
| **CHEMBL4303** | **Heat shock protein HSP 90-beta** |  | 1 |
| CHEMBL3836 | LIM domain kinase 1 |  | 1 |
| CHEMBL4246 | Tyrosine-protein kinase TEC |  | 1 |
| **CHEMBL4026** | **Signal transducer and activator of transcription 3** |  | 1 |
| CHEMBL3905 | Tyrosine-protein kinase Lyn |  | 1 |
| CHEMBL3885549 | Cyclin-T1/Cyclin-dependent-like kinase 5 |  | 1 |
| CHEMBL3984 | Tyrosine- and threonine-specific cdc2-inhibitory kinase |  | 1 |
| CHEMBL2094115 | MAP kinase p38 |  | 1 |
| **Target ChEMBL ID** | **Target name** | ***N* PC-3 compounds** | ***N* DU-145 compounds** |
| CHEMBL2073 | Tyrosine-protein kinase YES |  | 1 |
| CHEMBL2068 | Ephrin type-A receptor 2 |  | 1 |
| **CHEMBL1856** | **Steroid 5-alpha-reductase 2** |  | 1 |
| CHEMBL1841 | Tyrosine-protein kinase FYN |  | 1 |
| CHEMBL1833 | Serotonin 2b (5-HT2b) receptor |  | 1 |
| CHEMBL1822 | Inosine-5'-monophosphate dehydrogenase 1 |  | 1 |
| **CHEMBL1787** | **Steroid 5-alpha-reductase 1** |  | 1 |
| CHEMBL1075323 | Endoplasmin |  | 1 |
| CHEMBL2094126 | Cyclin-dependent kinase 2/cyclin E |  | 1 |
| CHEMBL2095165 | Heat shock protein HSP90 |  | 1 |
| **CHEMBL3024** | **Serine/threonine-protein kinase PLK1** |  | 1 |
| CHEMBL2959 | Tyrosine-protein kinase ITK/TSK |  | 1 |
| **CHEMBL2845** | **Protein phosphatase 2C beta** |  | 1 |
| CHEMBL2634 | Tyrosine-protein kinase CSK |  | 1 |
| CHEMBL2331044 | Myelin transcription factor 1 |  | 1 |
| CHEMBL2169736 | Tyrosyl-DNA phosphodiesterase 2 |  | 1 |
| **CHEMBL2146346** | **Neurogenic locus notch homolog protein 1** |  | 1 |
| CHEMBL2140 | Tryptophan 2,3-dioxygenase |  | 1 |
| CHEMBL2096618 | Bcr/Abl fusion protein |  | 1 |
| CHEMBL2095942 | Cyclin-dependent kinase 4/cyclin D |  | 1 |
| CHEMBL614818 | HEK293 |  | 1 |

**
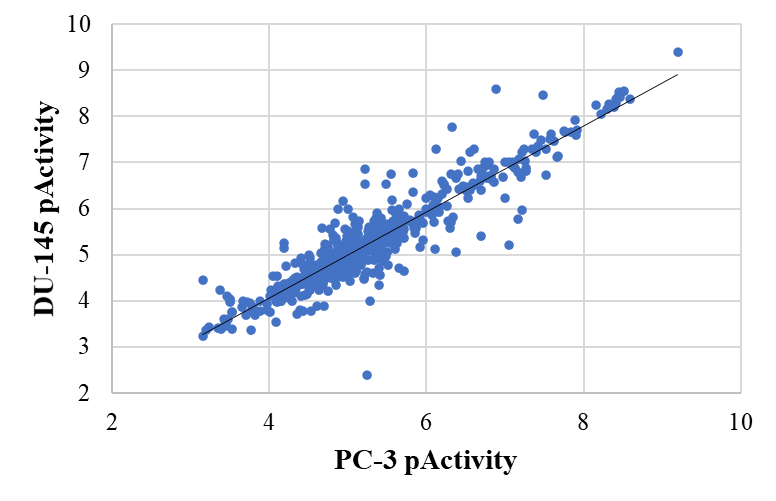
**

**Figure S1.** Correlation plot of the pActivities related to the 587 compounds common to the PC-3 and DU-145 datasets.


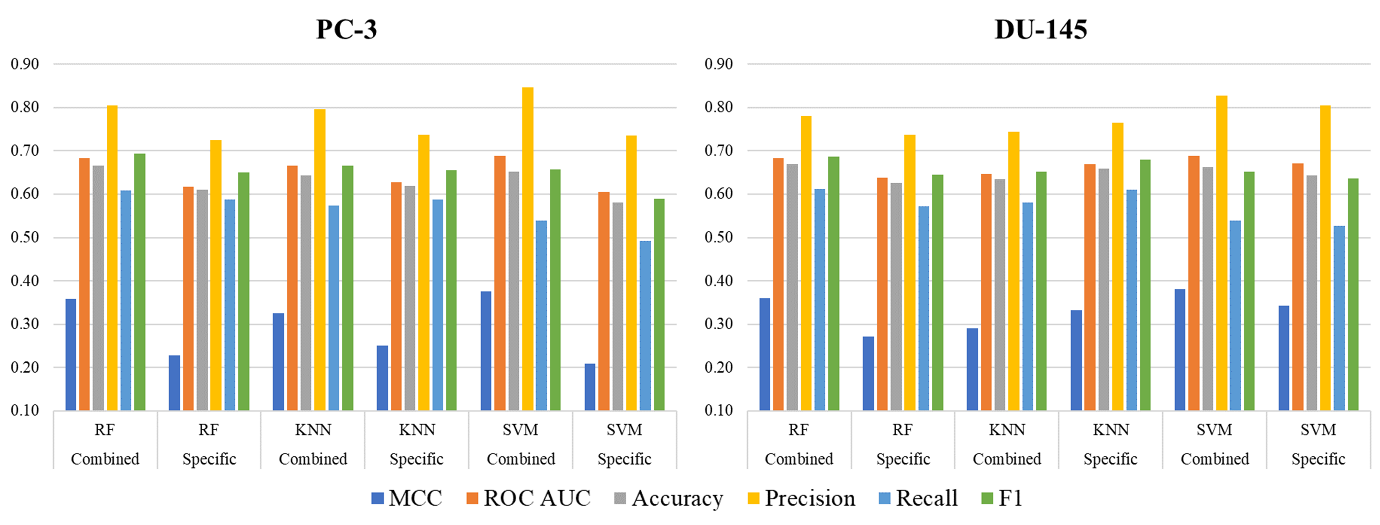


**Figure S2.** Prediction performance obtained on the test set for the combined PC-3/DU-145 models, and the PC-3 and DU-145 specific models. The results related to the predictions made on the PC-3 and DU-145 activity values using the RF, KNN and SVM algorithms. All the models were trained and validated on the GAP 20, PCC=95 datasets.

**Machine Learning Algorithms explored in the study**

In this study, ten machine learning (ML) algorithms, implemented in the scikit-learn python library (https://scikit-learn.org/stable/supervised_learning.html#supervised-learning) were applied for the development of the models. The ones explored belong to different classes of algorithms, which have been extensively used and reviewed in various drug discovery contexts, over the past decades [1-5]. Logistic Regression (LR) and Linear Discriminant Analysis (LDA) can be classified among supervised, regression, linear models in which the target value should derive by a linear combination of features. In particular, Logistic Regression can be used for both regression and classification tasks; this algorithm can require a dependent variable to be binary and the assignment to the classes is made on the probability of data to be classified on one of them, modeled by using a logistic function. The “*penalty*”, “*C*”, “*solver*” and “*max_iter*” hyperparameters were optimized in this study in search of the best-performing model. The Linear Regression algorithm has been previously used with success in drug discovery, for the identification, *e.g.*, of novel inhibitors of putrescine uptake in *Trypanosoma cruzi* [4]. While Linear Discriminant Analysis tries to find a combination of features separating two classes of objects. In particular, an object is assigned by LDA to one of the classes as a mean of probability of belonging to it divided by the probability of not being assigned to it, as calculated by a discriminant function. The “*solver*” hyperparameter was optimized in the study in search of the best-performing model. An example of the LDA algorithm in drug discovery is reported in reference 6.

Besides, K-Nearest Neighbor (KNN) can be listed within the supervised, classification, instance-based learning class of algorithms in which the information related to the training set is memorized and used to make predictions. KNN is a non-parametric classifier algorithm that makes predictions on the membership to a class of an object according to its similarity degree with respect to a set of references. The “*leaf_size*”, “*n_neighbors*” and “*p*” hyperparameters were subjected to optimization in the study.

Different types of the so-called “*classification trees*” algorithms, *i.e*., Decision Tree (Classification and Regression Trees – CART), Random Forest (RF) and Extra Trees (ET), were also explored during the development of the models. They are considered supervised non-linear ML methods. In all these types of algorithms, the classification of objects is performed according to the application of a series of decisions. In particular, Decision Tree is a non-parametric classification method, which can assign an object to different classes according to a set of rules it learns from labelled data. The rules are developed through the use of features and thresholds at each node yielding the largest information gain. DT has been extensively used in different tasks of drug discovery and also reviewed in the last years [7]. The “*max_features*”, “*max_depth*”, “*min_samples_split*”, “*min_samples_leaf*” and “*criterion*” hyperparameters were optimized in this study. Random Forest is classified among the ensemble methods. This algorithm is based on the application of a series of decision trees to various sub-samples of data (the data is bootstrapped), averaging their results to improve the overall prediction performances. RF classifiers have been as well extensively explored for different drug discovery tasks, as target fishing and ligand design [1,3,8,9]. In this study, the optimization of “*n_estimators*”, “*max_features*”, “*max_depth*”, “*min_samples_split*” and “*min_samples_leaf*” hyperparameters was performed for the development of the RF models. Very similar to RF, Extra Trees performs classification tasks by applying several decision trees to sub-samples of data. However, in this ensemble algorithm, randomness was obtained from the random splits performed on of the entire dataset. The “*n_estimators*”, “*max_features*”, “*min_samples_leaf*” and “*min_samples_split*” hyperparameters were optimized for the ET algorithm in the study.

Other algorithms in the context of supervised ensemble ML methods explored in this study are the Ada Boost (AB) and Gradient Boosting (GBM), both of them performing boosting modeling. In particular, Ada Boost can perform classification of objects through the combination of multiple, simple learners, each with low prediction performances (also known as “weak learners”). Multiple iterations are performed during the modelling process, at each of them different weights are assigned the single weak learners according to the misclassified samples, to the obtainment of good model convergency. For AB, the “*n_estimators*” and “*learning_rate*” hyperparameters were explored during the development of the models. Similarly to AB, Gradient Boosting generates multiple weak learners and combine them to get an overall improved performance. In this case, the worst performing weak learners are identified according to a gradient, rather than by the presence of high weights as in AB. Herein the “*loss*”, “*learning_rate*”, “*n_estimators*”, “*max_depth*”, “*min_samples_split*”, “*min_samples_leaf*”, “*max_features*” and “*subsample*” hyperparameters were optimized for the GB algorithm.

As KNN, Gaussian Naïve Bayes (NB) and Support Vector Machine (SVM) can be listed within the supervised, classification, instance-based learning class of algorithms. Support Vector Machine relies on the use of a high-dimensional geometrical shape, namely hyperplane, which allows the efficient separation of objects in two classes. Support vector machine is one among the most widely applied ML algorithms in tasks related to the identification and design of drugs [1,3, 10, 11]. In this study, we performed optimization of the “*gamma*”, “*C*”, and “*degree*” hyperparameters for SVM.

The Gaussian Naïve Bayes algorithm can perform classification applying probabilistic statistics based on Bayes’ theorem. In this study, the “*var_smoothing*” hyperparameter was optimized in the search of the best fitting model. Naïve Bayes algorithms have been previously used in several drug discovery tasks as for example in the search of effective drug combinations for ligands design [12].

**References**

1. Patel L, Shukla T, Huang X, et al (2020) Machine Learning Methods in Drug Discovery. Molecules 25(22);5277. <https://doi.org/390/molecules25225277>.
2. Lavecchia A. (2015) Machine-learning approaches in drug discovery: methods and applications. Drug Discov Today 20(3);318-31. <https://doi.org/10.1016/j.drudis.2014.10.012>.
3. Talevi A, Morales JF, Hather G, et al (2020) CPT Pharmacometrics Syst Pharmacol 9(3);129-142. <https://doi.org/10.1002/psp4.12491>.
4. Alberca LN, Sbaraglini ML, Morales JF, et al (2018) Cascade Ligand- and Structure-Based Virtual Screening to Identify New Trypanocidal Compounds Inhibiting Putrescine Uptake. Front Cell Infect Microbiol. 8;173. <https://doi.org/10.3389/fcimb.2018.00173>.
5. Tinivella A, Pinzi L, Rastelli G (2021) Prediction of activity and selectivity profiles of human Carbonic Anhydrase inhibitors using machine learning classification models. J Cheminform. 13(1);18. <https://doi.org/10.1186/s13321-021-00499-y>.
6. Talevi A, Cravero MS, Castro EA (2007) Discovery of anticonvulsant activity of abietic acid through application of linear discriminant analysis. Bioorg Med Chem Lett. 17(6);1684-1690. <https://doi.org/10.1016/j.bmcl.2006>.
7. Blower PE, Cross KP (2006) Decision tree methods in pharmaceutical research. Curr Top Med Chem 6(1);31-39. <https://doi.org/10.2174/156802606775193301>.
8. Lind AP, Anderson PC (2019) Predicting drug activity against cancer cells by random forest models based on minimal genomic information and chemical properties. PLoS ONE 14(7): e0219774. <https://doi.org/10.1371/journal.pone.0219774>.
9. Lee K, Lee M, Kim D (2017) Utilizing random Forest QSAR models with optimized parameters for target identification and its application to target-fishing server. BMC Bioinformatics 18 (Suppl 16), 567. <https://doi.org/10.1186/s12859-017-1960-x>.
10. Heikamp K, Bajorath J. (2014) Support vector machines for drug discovery. Expert Opin Drug Discovery 9(1);93-104. https://doi.org/10.1517/17460441.2014.866943.
11. Maltarollo VG, Kronenberger T, Espinoza GZ (2019) Advances with support vector machines for novel drug discovery. Expert Opin Drug Discov. 14(1):23-33. <https://doi.org/10.1080/17460441.2019.1549033>.
12. Bai LY, Dai H, Xu Q, (2018) Prediction of Effective Drug Combinations by an Improved Naïve Bayesian Algorithm. Int J Mol Sci. 19(2);467. https://doi.org/10.3390/ijms19020467.
